# Gold(I)‐Catalyzed Nucleophilic Allylation of Azinium Ions with Allylboronates

**DOI:** 10.1002/anie.202202305

**Published:** 2022-03-25

**Authors:** Luke O'Brien, Stephen P. Argent, Kristaps Ermanis, Hon Wai Lam

**Affiliations:** ^1^ The GlaxoSmithKline Carbon Neutral Laboratories for Sustainable Chemistry University of Nottingham Jubilee Campus, Triumph Road Nottingham NG7 2TU UK; ^2^ School of Chemistry University of Nottingham University Park Nottingham NG7 2RD UK

**Keywords:** Allylation, Allylboron, Azinium Ions, Catalysis, Gold

## Abstract

Gold(I)‐catalyzed nucleophilic allylations of pyridinium and quinolinium ions with various allyl pinacolboronates are reported. The reactions are completely selective with respect to the site of the azinium ion that is attacked, to give various functionalized 1,4‐dihydropyridines and 1,4‐dihydroquinolines. Evidence suggests that the reactions proceed through nucleophilic allylgold(I) intermediates formed by transmetalation from allylboronates. Density functional theory (DFT) calculations provided mechanistic insight.

## Graphical abstract

Gold(I)‐catalyzed nucleophilic allylations of pyridinium and quinolinium ions with allylboronates are reported. Transmetalation of the allylboronate with gold produces nucleophilic allylgold(I) species that add to the 4‐position of the azinium ion with complete regioselectivity to give functionalized 1,4‐dihydropyridines and 1,4‐dihydroquinolines. Density functional theory (DFT) calculations provided mechanistic insight.

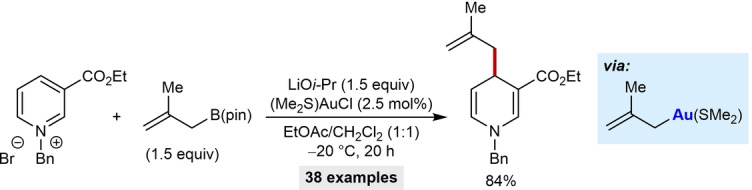

## Introduction

Since the turn of the century, the application of homogeneous gold catalysis in organic synthesis has grown significantly.[^1^, ^2^] The ability of gold complexes to act as powerful carbophilic Lewis acids for carbon‐carbon multiple bonds, as well as to achieve other modes of substrate activation, has led to the development of myriad new synthetic methods.[^1^, ^2^] Allylation reactions are important transformations that have been subject to gold catalysis.^[2]^ Gold‐catalyzed allylic substitutions involving nucleophilic additions to electrophilic allylating agents have been developed extensively.[^2^, ^3^] Gold‐catalyzed rearrangements that result in overall allylation are also well‐known.^[2]^ In contrast, gold‐mediated or gold‐catalyzed allylations involving the addition of nucleophilic allylating agents to electrophiles are comparatively underdeveloped.[^2^, ^4^] Given the many unique features of gold catalysts, addressing this deficiency could provide valuable new synthetic methods.

**Scheme 1:**
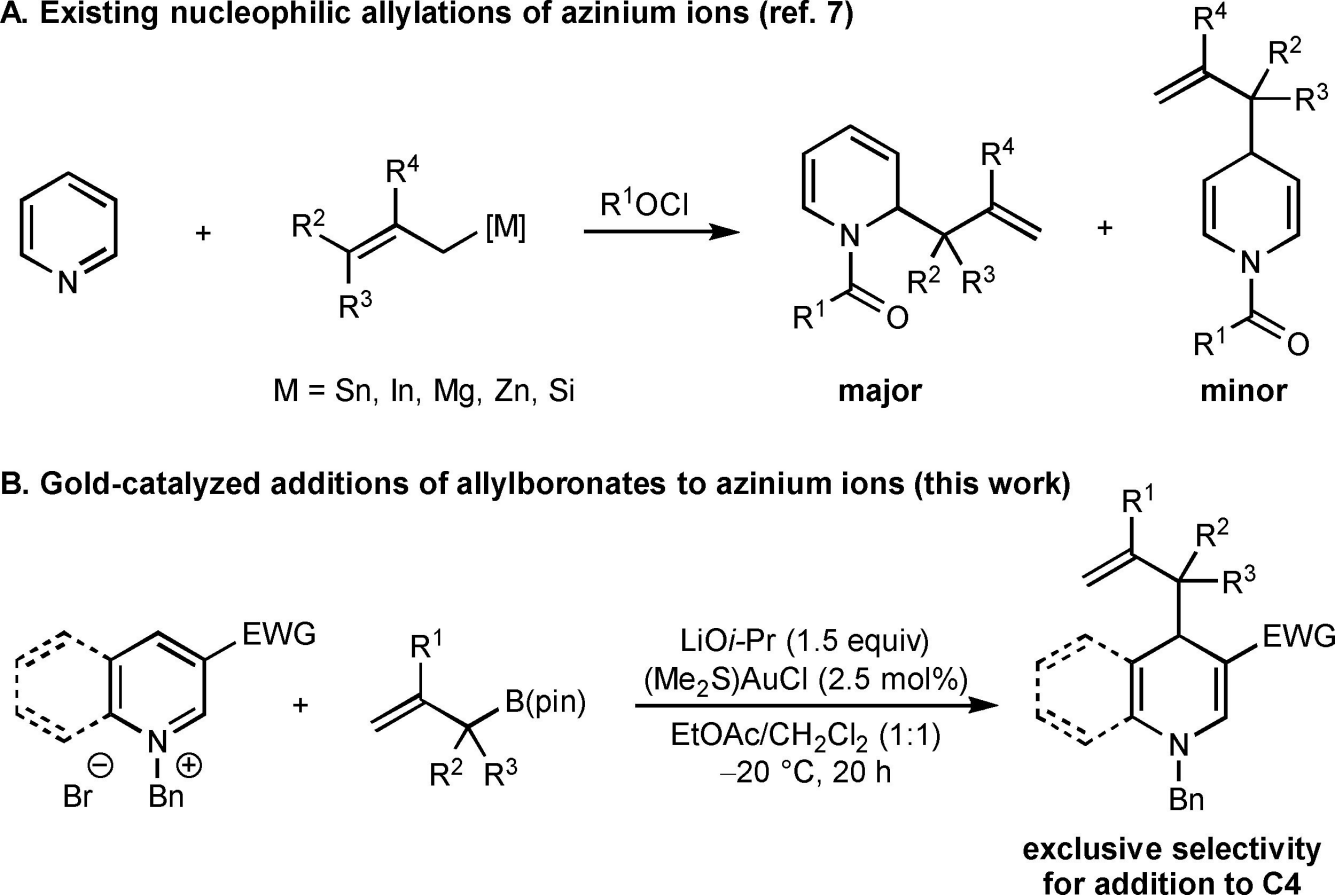
Nucleophilic allylations of azinium ions. pin=pinacolato.

Our research into gold‐catalyzed nucleophilic allylations arose when we became interested in nucleophilic additions to azines or their corresponding azinium ions.[^5^, ^6^] These are powerful reactions to access partially saturated nitrogen heterocycles, which are valuable chemical building blocks.[^5^, ^6^] Nucleophilic allylations of in situ‐generated *N*‐acylazinium ions have also been explored,^[7]^ typically using allyltin,[^7a^, ^7b^, ^7c^, ^7d^, ^7e^, ^7g^, ^7j^, ^7m^, ^7n^] allylindium,[^7h^, ^7j^, ^7l^, ^7m^] allylmagnesium,[^7a^, ^7b^, ^7p^, ^7q^] allylzinc,^[7m]^ or allylsilicon reagents.^[7i–k,^°^,p]^ However, these reactions are somewhat limited in scope and often lead to mixtures of regioisomeric products favoring those of addition to the 2‐ or 6‐positions. (Scheme 1A). Therefore, there is a need for nucleophilic allylations of azinium ions that exhibit high selectivity for addition to the 4‐position. It is known that catalytic enantioselective nucleophilic additions to azinium ions containing a strongly electron‐withdrawing group at the 3‐position often exhibit high selectivity for addition at C4,[^6i^, ^6j^, ^6k^, ^6p^] and this class of substrate therefore seemed a logical choice to study. Furthermore, despite the broad utility of allylboron reagents in nucleophilic allylations,[^8^, ^9^] only limited examples of their use in additions to azines^[10]^ or azinium ions[^7k^, ^7m^] have been reported. Herein, we describe the first gold‐catalyzed nucleophilic allylations of azinium ions with allylboronates to provide dihydropyridines and dihydroquinolines with complete regioselectivity in favor of addition to the 4‐position (Scheme 1B). The reactions proceed well without special precautions to exclude air or moisture. Nucleophilic allylgold(I) species^[11]^ are the likely intermediates in these reactions, which are formed by transmetalation from the allylboronates.

## Results and Discussion

Our study began with the reaction of *N*‐benzyl‐3‐nitropyridinium bromide (**1 a**) with 2‐methylallyl pinacolboronate (**2 a**) (Table 1). Stirring a mixture of **1 a** and **2 a** (1.5 equiv) together with (Me_2_S)AuCl (2.5 mol%) and LiO*i*‐Pr (1.5 equiv) in undried EtOAc/CH_2_Cl_2_ (1 : 1) at −20 °C under an air atmosphere for 1.5 h gave the allylated 1,4‐dihydropyridine **3 aa** in 98 % yield as determined by ^1^H NMR analysis using an internal standard (entry 1). Regioisomeric products resulting from allylation at either the 2‐ or 6‐positions of **1 a** were not detected. However, uncatalyzed additions of potassium allyltrifluoroborate, allyltributylstannane, or allylindium bromide to **1 a** gave mixtures of products resulting from addition to both the 4‐ and 6‐positions (see Supporting Information for details), which suggests that gold catalysis is important for high regioselectivity. A lower yield was obtained at room temperature (entry 2), and the mixed solvent system was important for reaction efficiency as shown by experiments using either EtOAc (entry 3) or CH_2_Cl_2_ (entry 4) alone. Although the reaction was successful using NaO*t*‐Bu (entry 5) or LiOH (entry 6), lower yields were obtained. Omitting either (Me_2_S)AuCl (entry 7) or LiO*i*‐Pr (entry 8) was detrimental, and both (Me_3_P)AuCl (entry 9) and (Ph_3_P)AuCl (entry 10) were inferior precatalysts. Precatalysts based on other metals such as rhodium, iridium, palladium, cobalt, nickel, or copper showed little to no reactivity.

**Table 1:** Evaluation of reaction conditions.^[a]^

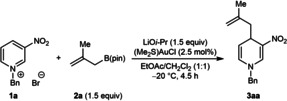		
Entry	Deviation from Standard Conditions	Yield of **3 aa** ^[b]^
**1**	None	98
**2**	At room temperature	64
**3**	EtOAc as solvent	56
**4**	CH_2_Cl_2_ as solvent	36
**5**	NaO*t*‐Bu instead of LiO*i*‐Pr	30
**6**	LiOH instead of LiO*i*‐Pr	71
**7**	No (Me_2_S)AuCl	4
**8**	No LiO*i*‐Pr	NR
**9**	(Me_3_P)AuCl instead of (Me_2_S)AuCl	21
**10**	(Ph_3_P)AuCl instead of (Me_2_S)AuCl	17

[a] Reactions were conducted with 0.10 mmol of **1 a** in 1 mL of solvent. [b] Determined by ^1^H NMR analysis using 1,3,5‐trimethoxybenzene as an internal standard. NR=no reaction.

With effective reaction conditions (Table 1, entry 1) in hand, the scope of the process with respect to the *N*‐benzylazinium bromide **1** was evaluated in reactions with 2‐methylallyl pinacolboronate (**2 a**) (Table 2). In all cases, complete regioselectivity for allylation at the 4‐position of the azinium ion was observed and alternative regioisomers were not detected. As well as the high‐yielding reaction to form **3 aa**, the reaction was successful with *N*‐benzyl pyridinium bromides containing various electron‐withdrawing groups at the 3‐position, such as cyano (**3 ba**), acetyl (**3 ca**), a range of esters (**3 da**–**3 ga**), or a Weinreb amide (**3 ha**). Good yields were generally observed but the yields were lower in the case of substrates containing an *n*‐butyl ester (**3 fa**) or Weinreb amide (**3 ha**). Notably, substrates containing chloro or bromo groups at 3‐position also reacted successfully to give 1,4‐dihydropyridines **3 ia** and **3 ja** in 94 % and 82 % yield, respectively. However, a 3‐unsubstituted substrate and a substrate with a methyl group at the 3‐position were unreactive, and none of the desired products **3 ka** or **3 la** were observed. *N*‐Benzyl‐2‐methyl‐5‐nitropyridinium bromide provided 1,4‐dihydropyridine **3 ma** in 28 % yield. Pleasingly, a range of *N*‐benzylquinolinium bromides also reacted efficiently with **2 a** to give 1,4‐dihydroquinolines **3 na**–**3 qa** in 84–94 % yield; these substrates had cyano (**3 na**), acetyl (**3 oa**), ester (**3 pa**), or Weinreb amide (**3 qa**) groups at the 3‐position. A gram‐scale reaction using 4.00 mmol of substrate **1 e** also proceeded well to give **3 ea** in 85 % yield.

**Table 2:**
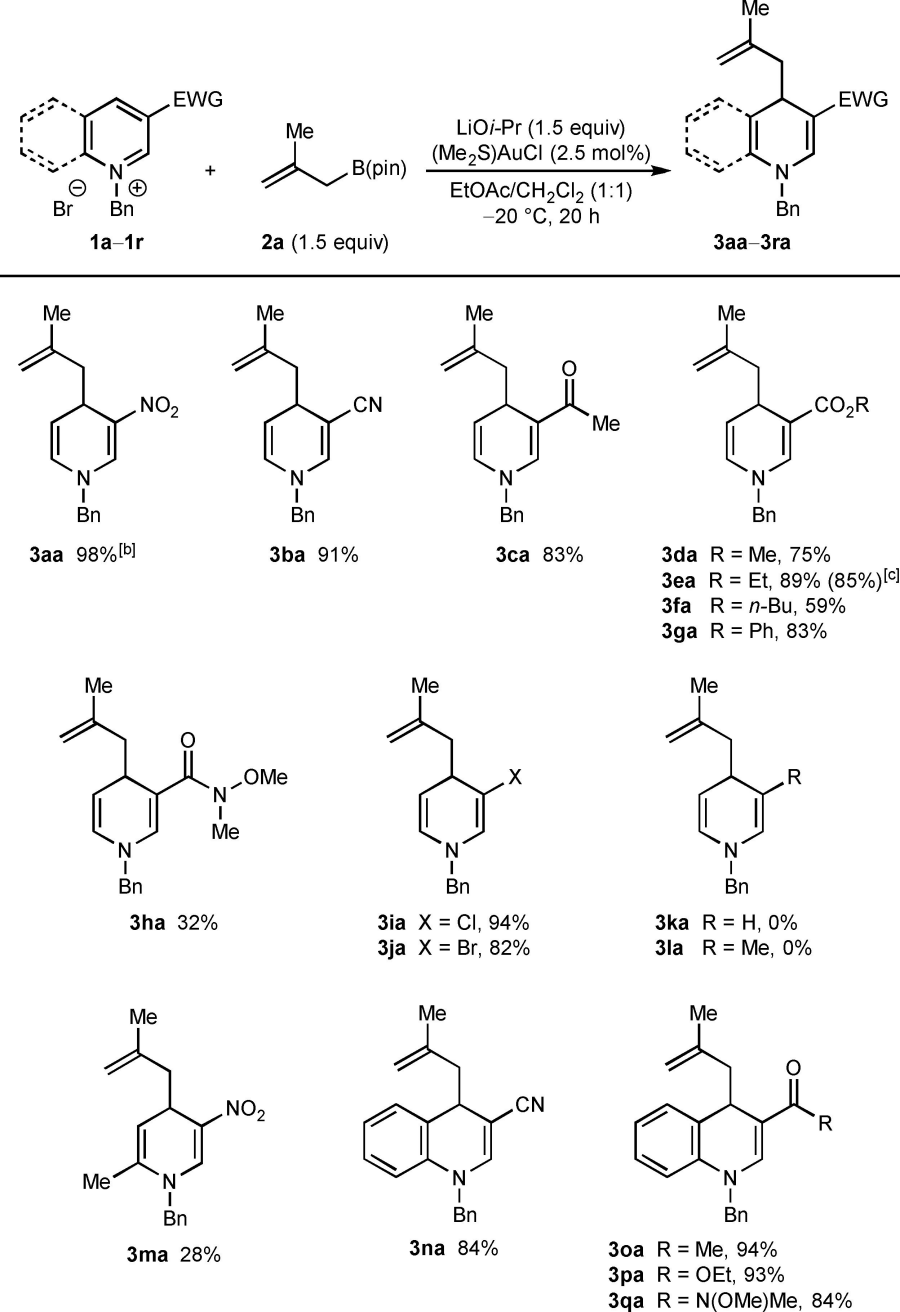
Scope of azinium salts.^[a]^

[a] Reactions were conducted with 0.50 mmol of **1** in 5 mL of EtOAc/CH_2_Cl_2_ (1 : 1). Yields are of isolated products. [b] The reaction time was 4.5 h. [c] Yield in parentheses is of a reaction conducted using 4.0 mmol of **1 e**.

Next, variation of the nitrogen substituent and counterion was briefly investigated using 2‐methylallyl pinacolboronate (**2 a**) as the allylating agent (Table 3). *N*‐Methyl‐3‐nitropyridinium iodide (**1 r**) provided 1,4‐dihydropyridine **3 ra** in 58 % yield. Changing the *N*‐substituent to an *n*‐butyl group was also tolerated (**3 sa**) but a much higher yield was obtained using the bromide salt (84 %) as opposed to the iodide salt (37 %), possibly because of increased solubility. *N*‐Octyl‐3‐nitropyridinium bromide (**1 t**) gave **3 ta** in 81 % yield. Finally, *N*‐(2‐naphthylmethyl)‐3‐nitropyridinium bromide gave **3 ua** in 87 % yield.

**Table 3:**
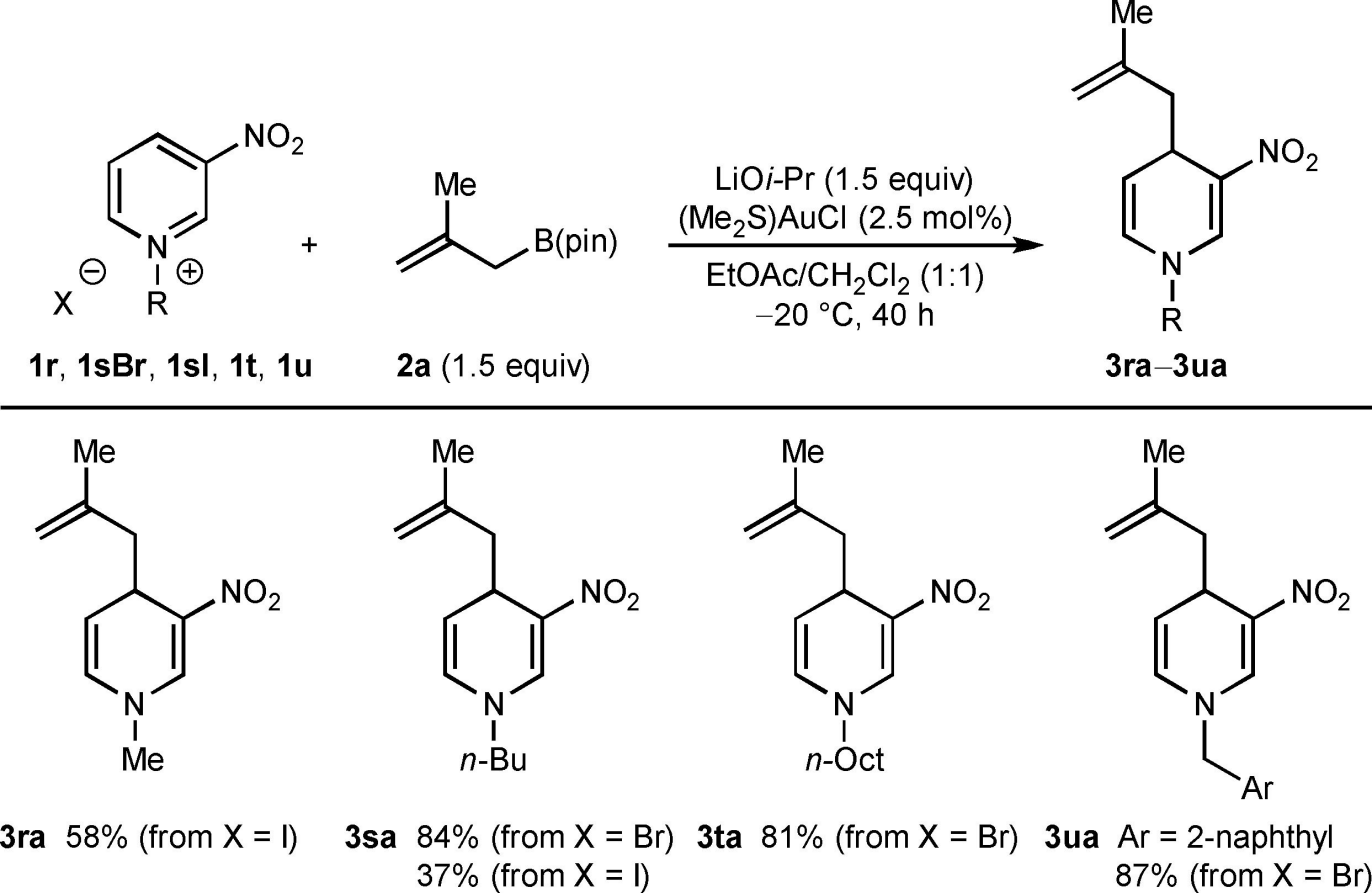
Variation of *N*‐substituent and counterion in the azinium salt.^[a]^

[a] Reactions were conducted with 0.50 mmol of **1** in 5 mL of EtOAc/CH_2_Cl_2_ (1:1)_._

The scope of this method with respect to the allylboronate was then investigated using azinium salts **1 a**, **1 b**, and **1 e** (Table 4). Allyl pinacolboronate (**2 b**) reacted successfully with various 3‐substituted *N*‐benzylpyridinium bromides to give **3 ab**, **3 bb**, and **3 eb** in 41–90 % yield. These yields are lower than those of the corresponding reactions with 2‐methylallyl pinacolboronate (Table 2, **3 aa**, **3 ba**, and **3 ea**) and this may be attributed to the lower nucleophilicity of allyl pinacolboronate. 2‐Phenylallyl pinacolboronate (**2 c**) is an effective allylating agent and provided **3 ac**, **3 bc**, and **3 ec** in good yields. Interestingly, the reaction of α,α‐dimethylallyl pinacolboronate (**2 d**) with **1 a** occurred with high α‐regioselectivity (with respect to the allylating agent) to give the reverse‐prenylated 1,4‐dihydropyridine **3 ad** in 58 % yield, and none of the alternative prenylation product resulting from γ‐allylation was observed. High α‐regioselectivities were also observed in the reactions of **1 a** with α‐methyl‐substituted allylboronate *rac*‐**2 e** and the geranyl‐bromide‐derived α,α‐disubstituted allylboronate *rac*‐**2 f** to give 1,4‐dihydropyridines **3 ae** and **3 af** in 57 % and 25 % yield, respectively, as mixtures of inseparable diastereomers. 2‐Cyclohexenyl pinacolboronate (*rac*‐**2 g**) reacted with **1 a** to give 1,4‐dihydropyridine **3 ag** in 70 % yield as a 2.2 : 1 mixture of inseparable diastereomers.

**Table 4:**
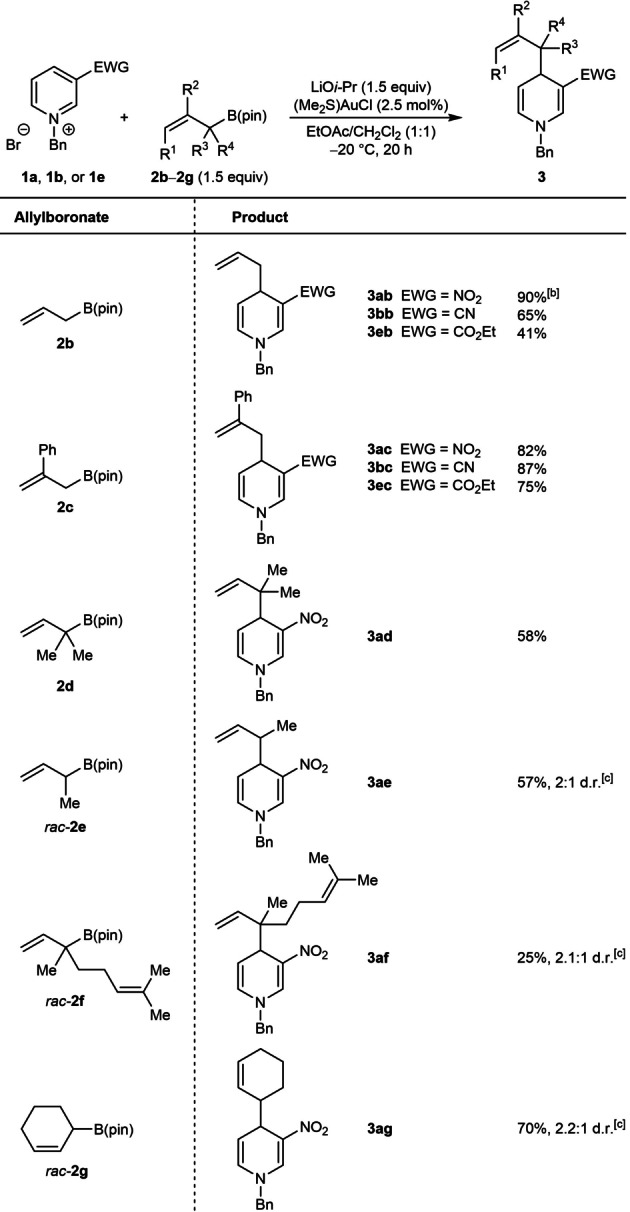
Scope of allylboronate.^[a]^

[a] Reactions were conducted with 0.50 mmol of **1 a**, **1 b**, or **1 e** in 5 mL of EtOAc/CH_2_Cl_2_ (1:1). [b] The reaction time was 1.5 h. [c] Isolated as a mixture of inseparable diastereomers.

The reactions described thus far are completely regioselective with respect to addition to the 4‐position of the azinium ion to give 1,4‐dihydropyridines and 1,4‐dihydroquinolines. Therefore, it was of interest to examine the reaction of pyridinium bromide **1 v**, which contains a methyl group at the 4‐position that could block allylation at this site [Eq. (1)]. Indeed, reaction of **1 v** with allyl pinacolboronate (**2 b**) gave the 2‐allylated product **3 vba** in 43 % yield and the 6‐allylated product **3 vbb** in 13 % yield. The importance of a strong electron‐withdrawing group at the 3‐position in promoting allylation at the 4‐position is shown by the reaction of allylboronate **2 a** with substrate **1 w**, which contains a benzyl group at C3. This reaction gave the 2‐allylated product **3 wa** in 40 % yield and none of the 4‐allylated product was observed [Eq. 2].

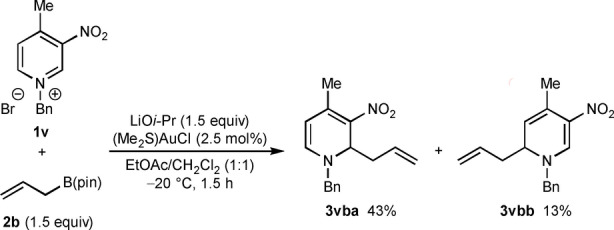




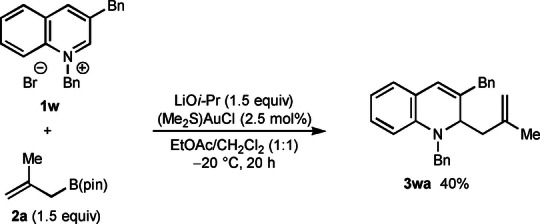




Additional experiments revealed examples where lower α:γ selectivity with respect to the allylboronate was observed [Eqs. (3)–(5)]. First, reaction of prenyl pinacolboronate **2 h** with **1 a** gave a 31 % yield of a 4 : 1 mixture of the inseparable regioisomers **3 ad** and **3 ah** favoring the reverse prenylated product **3 ad**. This result should be contrasted with the corresponding reaction using α,α‐dimethylallyl pinacolboronate shown in Table 2, which gave only the reverse prenylation product **3 ad**. Furthermore, regioisomeric mixtures were also obtained in the reactions of pyridinium salt **1 a** with crotyl pinacolboronates [Eqs. (4) and (5)]. With (*Z*)‐crotyl boronate (*Z*)‐**2 i**, a 12 : 1 mixture of regioisomers was obtained, favoring the α‐allylation product **3 ai** ^[12]^ over the γ‐allylation product **3 ae**, the latter of which was formed in 2 : 1 d.r. [Eq. (4)]. In contrast, with (*E*)‐crotylboronate (*E*)‐**2 i**, the α:γ selectivity decreased to 2.3 : 1 [Eq. (5)]. Interestingly, the α‐addition product **3 ai** was obtained as the *Z*‐isomer^[13]^ and **3 ae** was formed as 1.4 : 1 mixture of inseparable diastereomers.

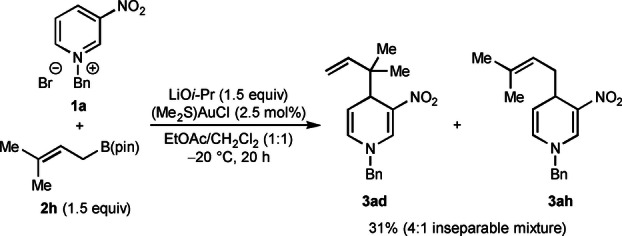




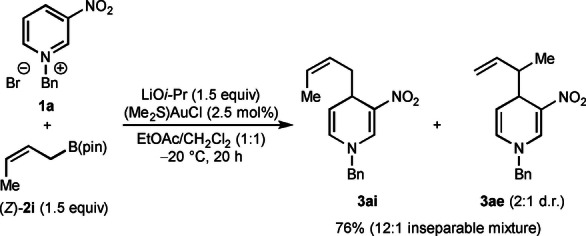




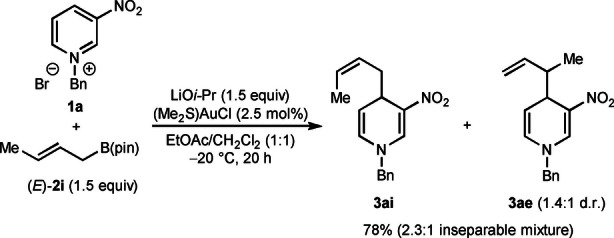




The experiments shown in Equations (3)–(5) employ allylboronates containing unsymmetrical allyl moieties and therefore it was of interest to examine the reaction of **1 a** with the α,α‐dideuterated allylboronate [D]_2_‐**2 b**, which has a pseudosymmetric allyl fragment [Eq. (6)]. This experiment gave a 2 : 1 mixture of regioisomers [D]_2_‐**3 aba** and [D]_2_‐**3 abb** in 76 % yield in favor of the α‐allylation product [D]_2_‐**3 aba**. The production of regioisomers in Equations (3)–(6) suggest that these reactions proceed through nucleophilic allylgold(I) intermediates that can exist in one of two σ‐isomeric forms. Allylgold(I)^[11]^ or allylgold(III) species^[3, 4a,b,i.j, 14]^ have been described as intermediates or products in various gold‐catalyzed or gold‐mediated reactions.

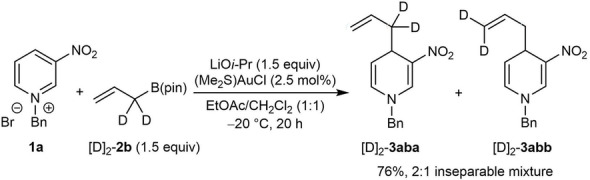




**Scheme 2:**
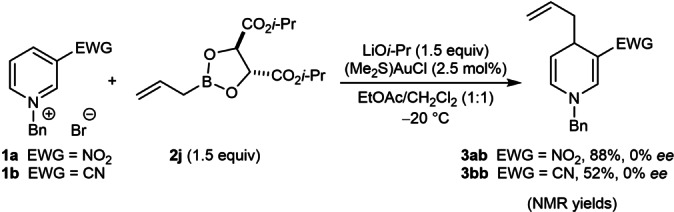
Allylation reactions using a chiral allylboronate.

The reaction of the chiral allylboronate **2 j** ^[15]^ with pyridinium salts **1 a** or **1 b** under the standard conditions gave products **3 ab** and **3 bb** but in 0 % *ee* (Scheme 2). The complete lack of asymmetric induction may indicate that boron is not involved in the carbon‐carbon bond‐forming step, and further suggests that allylgold(I) species are likely intermediates.

To rule out the participation of allylic radicals in these reactions, which in principle could also explain the production of regioisomeric products in Equation (3)–(6), the reaction of **1 a** with **2 a** was conducted in the presence of TEMPO (1.0 equiv) [Eq. (7)]. TEMPO did not have a detrimental effect on the yield of **2 a**, which suggests allylic radicals are not involved.

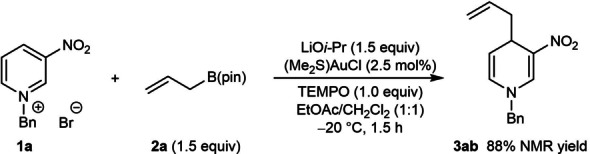




Finally, to provide more direct evidence for allylgold(I) species, equimolar quantities of allyl pinacolboronate **2 b**, LiO*i*‐Pr, and (Me_2_S)AuCl were combined in DMSO‐d_6_ ^[16]^ and the mixture was analyzed by ^1^H NMR spectroscopy [Eq. (8)]. Although full consumption of **2 b** was not observed, new signals consistent with the formation of a 1 : 1 mixture of the σ‐allylgold species **4** and HOB(pin) appeared, the latter of which was further confirmed by ^11^B NMR spectroscopy. The hydroxyl group of HOB(pin) likely results from the presence of H_2_O in DMSO‐d_6_. Essentially identical results were observed when a 1 : 1 mixture of (Me_2_S)AuCl and LiO*i*‐Pr were mixed in DMSO‐d_6_ for 30 min prior to the addition of allyl pinacolboronate. To our knowledge, the reactions described herein are the first examples of the formation of allylgold(I) species from allylboron reagents.[^17^, ^18^]

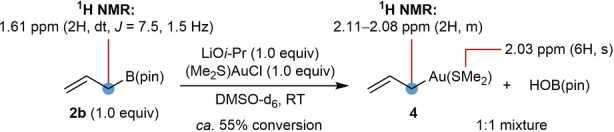




To gain further mechanistic insight, computational studies were performed at the PBE0^[19]^/def2‐TZVP^[20]^/SMD(CH_2_Cl_2_)^[21]^ level. First, the nature of the active catalytic species was investigated. LiO*i*‐Pr is used as the base in the reactions, but because undried solvents were used and no precautions were taken to exclude air or moisture, it is likely that LiOH is also present, formed by reaction of LiO*i*‐Pr with H_2_O. Reaction of (Me_2_S)AuCl with LiO*i*‐Pr or LiOH likely produces a gold(I) isopropoxide or hydroxide by ligand exchange.^[22]^ The relative computational free energies of (Me_2_S)AuCl, (Me_2_S)AuOH and (Me_2_S)AuO*i*‐Pr were calculated to be 0.0 kcal mol^−1^, 1.8 kcal mol^−1^, and 5.8 kcal mol^−1^, respectively, which suggests (Me_2_S)AuO*i*‐Pr is unlikely to be the active catalyst. Because (Me_2_S)AuCl is unable to catalyze the reaction on its own (Table 1, entry 8), (Me_2_S)AuOH was assumed to be the active catalyst in the following calculations.

With the likely catalytic species identified, reactions between the pyridinium bromide **1 a** with either the primary allylboronate **2 h** [Eq. (3)] or the tertiary allylboronate **2 d** (Table 4, product **3 ad**) were investigated computationally. Allylboronates **2 h** and **2 d** were selected to compare their transmetalation to gold, investigate possible interconversion between isomeric σ‐allylgold species, and gain insight into the α:γ allylation regioselectivity.

**Scheme 3:**
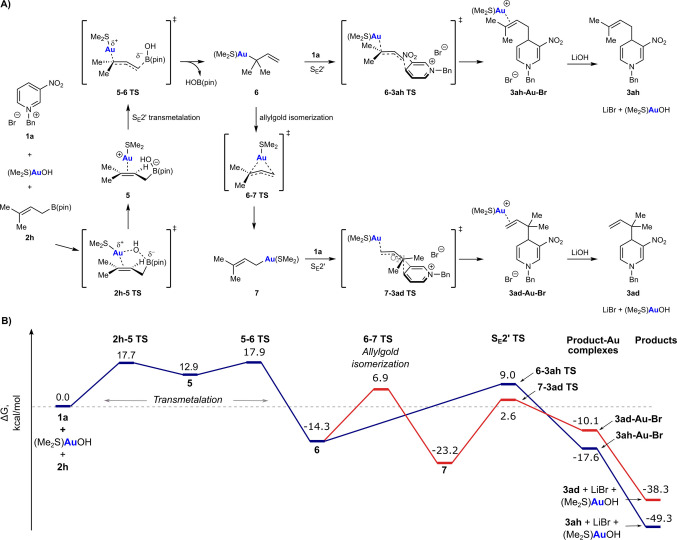
Computational exploration of reaction pathways arising from **1 a** and **2 h**. Mechanistic pathways explored (A) and the corresponding starting material, intermediate, product and transition state energy diagram (B). Free energies shown are relative to the starting materials and calculated at PBE0/def2‐TZVP/SMD(CH_2_Cl_2_).

With the primary allylboronate **2 h**, transmetalation with (Me_2_S)AuOH was calculated to be most favorable through an S_E_2′ mechanism^[23]^ involving the boron‐ate complex **5**, with the alkene coordinated to a cationic [(Me_2_S)Au]^+^ fragment (Scheme 3A). The formation of **5** occurs by a *syn* pathway through transition state **2 h**‐**5‐TS**, which allows facile transfer of the hydroxide ligand from gold to boron, with minimal ion separation. From **5**, S_E_2′ transmetalation is completed by formation of the carbon‐gold bond and loss of HOB(pin) through transition state **5‐6‐TS** to give the tertiary allylgold species **6**. The formation of **5** and **6** have relatively low barriers of 17.7 kcal mol^−1^ and 17.9 kcal mol^−1^, respectively, relative to the starting materials, and overall, transmetalation is thermodynamically very favorable (Scheme 3B).

The tertiary allylgold species **6** can then react directly with pyridinium bromide **1 a** in an S_E_2′ allylation to give the prenylated product **3 ah** through transition state **6‐3 ah‐TS**, with a barrier of 23.3 kcal mol^−1^. Experimentally, however, **3 ah** was the minor product, with the major product being the reverse‐prenylated isomer **3 ad** [Eq. (3)]. Product **3 ad** most likely arises from the isomerization of the tertiary allylgold species **6** into the primary allylgold species **7** through a π‐allylgold transition state **6‐7‐TS**,^[11d]^ which then engages in S_E_2′ allylation of **1 a** with a barrier of 25.8 kcal mol^−1^. The isomerization of **6** to **7** was found to be thermodynamically very favorable, with a barrier of 21.2 kcal mol^−1^, which is 2.1 kcal mol^−1^ lower than the competing nucleophilic allylation of **1 a** with **6**. Therefore, the DFT calculations suggest that the production of **3 ad** should be favored over **3 ah**, which matches the experimental results (4 : 1 ratio of **3 ad**:**3 ah**). Our findings of the relative energies of primary vs tertiary allylgold(I) species and their interconversion through a high‐energy π‐allylgold transition state are consistent with a previous study by Hashmi and co‐workers.^[11d]^ Alternative pathways for producing **3 ad** involving transmetalation and/or nucleophilic allylation proceeding through S_E_2 rather than S_E_2′ mechanisms were also calculated and discounted because of high barriers (see the Supporting Information for details).

**Scheme 4:**
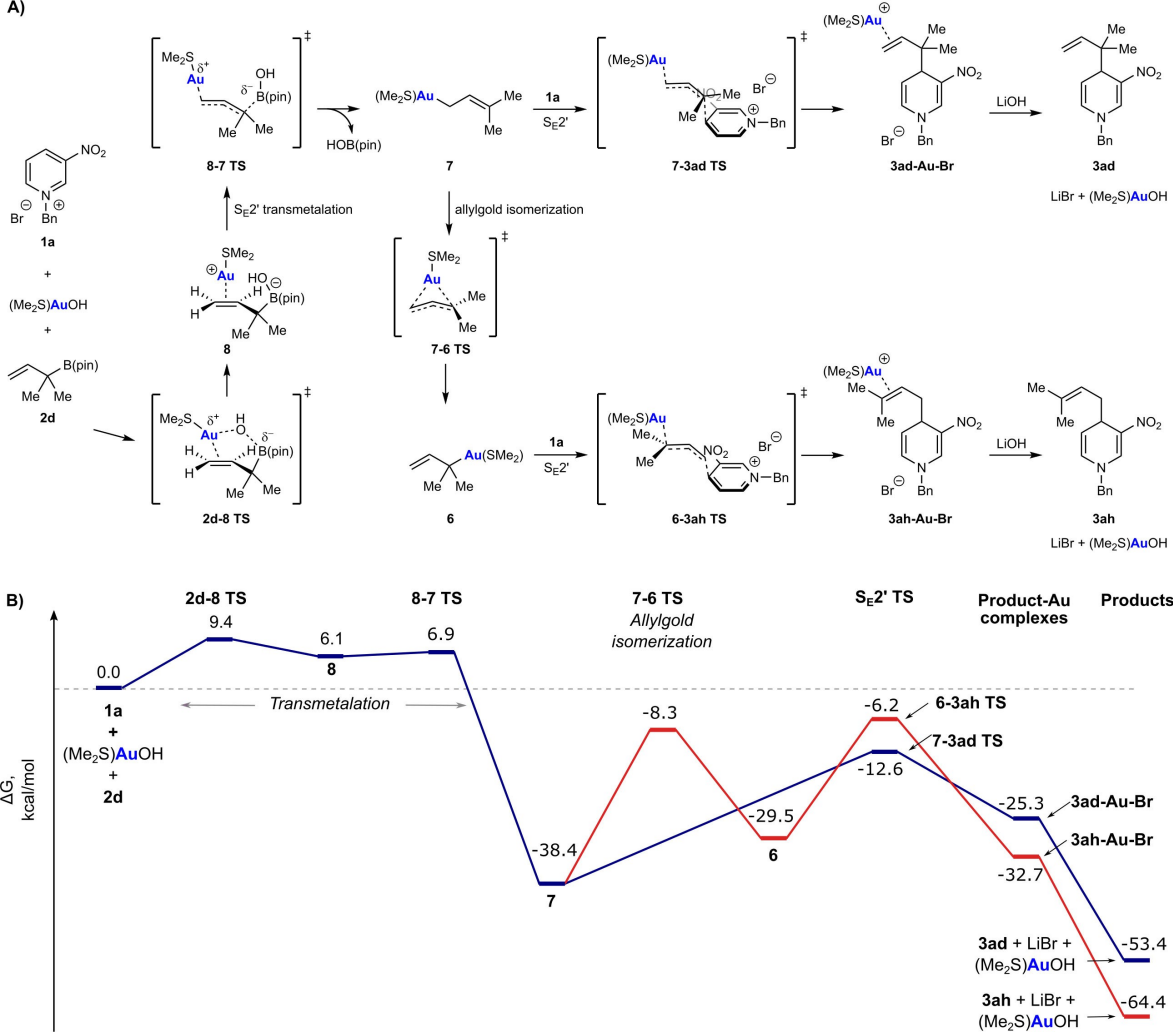
Computational exploration of reaction pathways arising from **1 a** and **2 d**. Mechanistic pathways explored (A) and the corresponding starting material, intermediate, product and transition state energy diagram (B). Free energies shown are relative to the starting materials and calculated at PBE0/def2‐TZVP/SMD(CH_2_Cl_2_).

Next, the reaction of the isomeric tertiary allylboronate **2 d** with pyridinium bromide **1 a**, which gave only the reverse‐prenylated product **3 ad** (Table 4), was investigated computationally (Scheme 4). Transmetalation (via **8**) was once again found to be very facile and compared with the primary allylboronate **2 h** (Scheme 3), is even more thermodynamically favorable (Δ*G*=−38.4 kcal mol^−1^ vs. −14.3 kcal mol^−1^) because it produces a more stable primary allylgold species **7** (relative to the tertiary allylgold species **6**) from a higher energy tertiary allylboronate. As described previously (Scheme 3), the nucleophilic allylation of **1 a** with **7** to give **3 ad** has a barrier of 25.8 kcal mol^−1^. However, the competing allylgold isomerization of **7** to give **6** has a barrier of 30.1 kcal mol^−1^, which is 4.3 kcal mol^−1^ higher than nucleophilic allylation, thus making the production of the tertiary allylgold species **6** and the corresponding allylation product **3 ah** much less feasible. This is a good match for the experiment, where no **3 ah** was observed (Table 4). The different outcomes of the crotylation reactions using allylboronates (*Z*)‐**2 i** or (*E*)‐**2 i** Equations (4) and (5) are more difficult to explain at the present time and are likely to require more detailed computational studies in future.

**Scheme 5:**
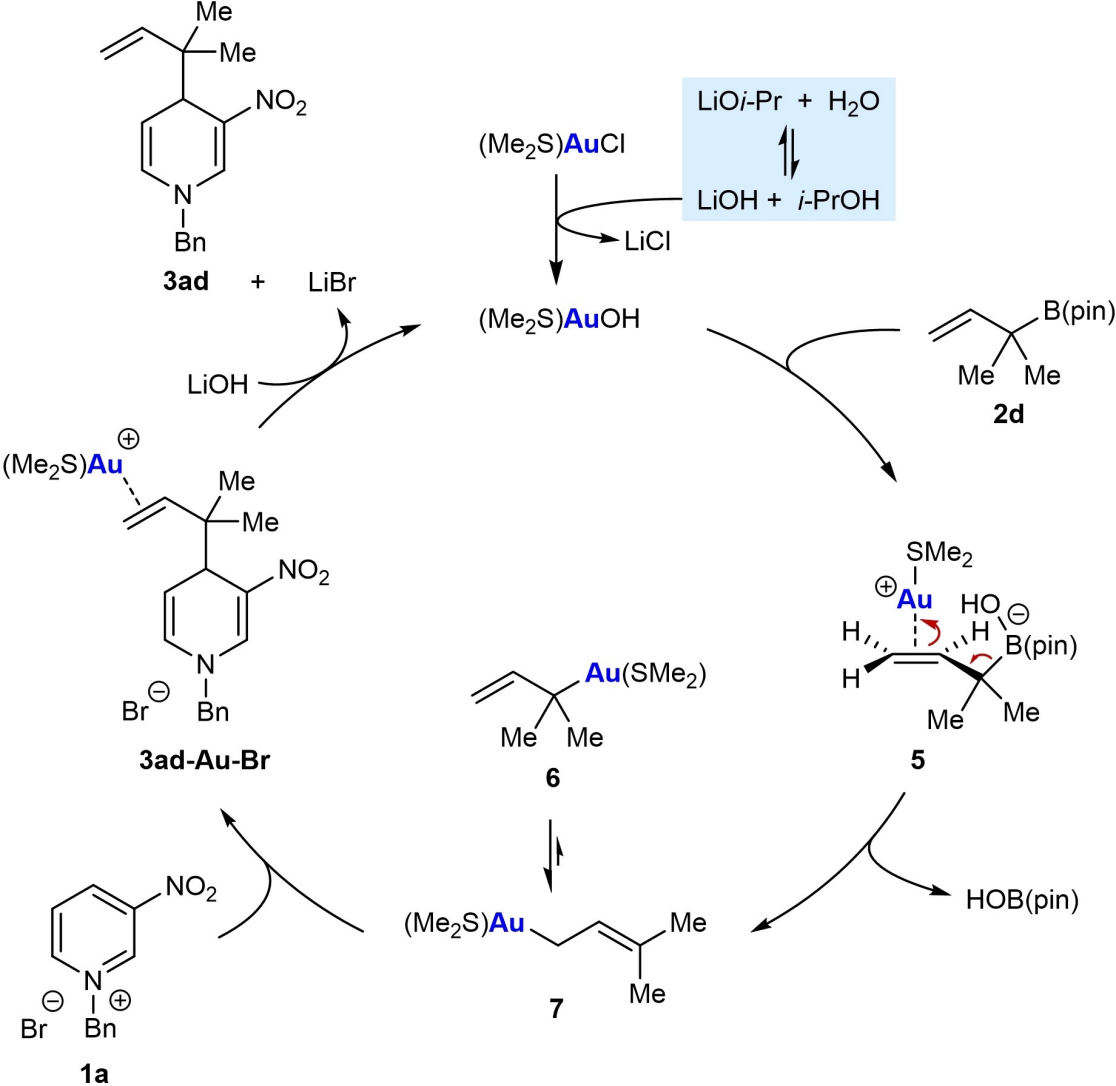
Proposed catalytic cycle.

Based on our experimental results and computational studies, a proposed catalytic cycle for these reactions using representative substrates **1 a** and **2 d** is illustrated in Scheme 5. The reaction of LiO*i*‐Pr with trace H_2_O present in the reactions produces LiOH, which then reacts with (Me_2_S)AuCl to give (Me_2_S)AuOH. Transmetalation of (Me_2_S)AuOH with allylboronate **2 d** though the gold‐bound, boron‐ate complex **5** gives primary allylgold species **7**. As described above, the isomerization of **7** into the tertiary allylgold species **6** is unfavorable compared to the reaction of **7** with the pyridinium salt **1 a**. The S_E_2′ nucleophilic allylation of **1 a** with **7** gives the gold‐bound product **3 ad‐Au‐Br**, which can react with LiOH to release the product **3 ad**, LiBr, and (Me_2_S)AuOH.

**Scheme 6:**
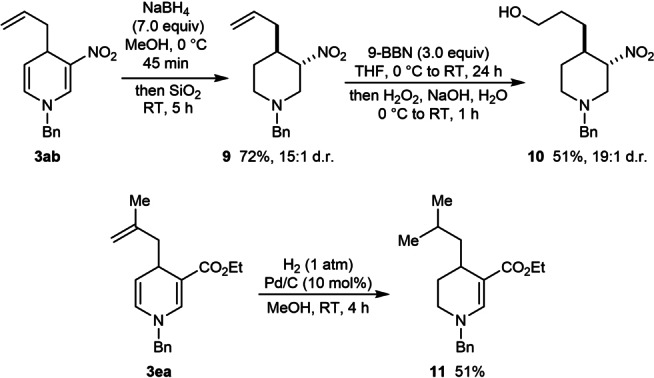
Further transformations of representative products **3 ab** and **3 ea**.

To demonstrate the synthetic utility of the products, further transformations were conducted on representative allylation products **3 ab** and **3 ea** (Scheme 6). Reduction of the enamines of **3 ab** was accomplished using NaBH_4_ in MeOH,^[6^°^,q]^ and after treatment with SiO_2_ to epimerize the stereocenter bearing the nitro group,^[6i]^ piperidine **9** was obtained in 72 % yield as a 15 : 1 mixture of diastereomers. Hydroboration/oxidation of the alkene of **9** then gave primary alcohol **10**, which was isolated in 51 % yield as a 19 : 1 mixture of diastereomers. In another example, Pd‐catalyzed hydrogenation of **3 ea** led to selective reduction of the 1,1‐disubstituted alkene and the less substituted enamine to give tetrahydropyridine **11** in 51 % yield (Scheme 6).

## Conclusion

Herein, we have described gold(I)‐catalyzed nucleophilic allylations of diverse azinium ions with allyl pinacolboronates to give functionalized 1,4‐dihydropyridines and 1,4‐dihydroquinolines. The reactions exhibit exclusive regioselectivity for attack at the 4‐position of the substrates and require no special precautions to exclude air or moisture. The likely reactive species are nucleophilic σ‐allylgold(I) species formed by transmetalation from the allylboronate, and this assertion was supported by NMR spectroscopy, the results of reactions using unsymmetrical allylboronates, and computational studies. To our knowledge, these reactions are the first demonstrations of accessing allylgold(I) species from allylboron reagents. Future work is aimed at enantioselective variants of this process^[24]^ along with gold(I)‐catalyzed nucleophilic allylations of other electrophiles.

## Conflict of interest

The authors declare no conflict of interest.

1

## Supporting information

As a service to our authors and readers, this journal provides supporting information supplied by the authors. Such materials are peer reviewed and may be re‐organized for online delivery, but are not copy‐edited or typeset. Technical support issues arising from supporting information (other than missing files) should be addressed to the authors.

Supporting Information

Supporting Information

Supporting Information

## Data Availability

The data that support the findings of this study are available in the supplementary material of this article and at: https://doi.org/10.17639/nott.7173.
