# Supplementary material for: Gold(I)‐Catalyzed Nucleophilic Allylation of Azinium Ions with Allylboronates
Source: Angew Chem Int Ed Engl. 2022 Mar 25;61(22):e202202305. doi: 10.1002/anie.202202305 (PMC9314030; doi:10.1002/anie.202202305)
Supplement: Supplementary file 3 — Supporting Information [file ANIE-61-0-s003.pdf]

Angewandte Chemie
Eine Zeitschrift der Gesellschaft Deutscher Chemiker

Supporting Information

Gold(I)-Catalyzed Nucleophilic Allylation of Azinium Ions with Allylboronates

L. O'Brien, S. P. Argent, K. Ermanis, H. W. Lam**

Author Contributions

L.O. Conceptualization:Equal; Data curation:Lead; Formal analysis:Lead; Investigation:Lead; Methodology:Lead; Writing – review & editing:Equal
S.A. Data curation:Supporting; Formal analysis:Supporting
K.E. Data curation:Supporting; Formal analysis:Supporting; Investigation:Supporting; Methodology:Supporting; Software:Lead; Writing – review & editing:Supporting
H.L. Conceptualization:Equal; Formal analysis:Supporting; Supervision:Lead; Writing – original draft:Lead; Writing – review & editing:Equal

Supporting Information

1. General Information 3
2. Preparation of Substrates 4
3. Gold-Catalyzed Nucleophilic Allylation of Azinium Salts 16
4. Uncatalyzed Nucleophilic Allylations of Azinium Salt **1a** 37
5. Allylation Using Allyl Pinacolboronate $[D]_2$-**2b** 40
6. Allylations Using Allylboronate **2j** 41
7. Allylation in the Presence of TEMPO 43
8. ^{1}H NMR and ^{11}B NMR Study of Transmetalation from Allylboronate **2b** 44
9. Computational Studies 50
10. Further Transformations 56
11. NMR Spectra of New Compounds 59
12. References 119

1. General Information

All air-sensitive reactions were carried out under an inert atmosphere using oven-dried apparatus. All commercially available reagents were used as received unless otherwise stated. Petroleum ether refers to Sigma-Aldrich product 24587 (petroleum ether boiling point 40-60 °C). Thin layer chromatography (TLC) was performed on Merck DF-Alufoilien 60F254 0.2 mm precoated plates. Compounds were visualized by exposure to UV light or by dipping the plates into solutions of potassium permanganate, ninhydrin, phosphomolybdic acid or vanillin followed by gentle heating. Flash column chromatography was carried out using silica gel (Fisher Scientific 60 Å particle size 35-70 micron or Fluorochem 60 Å particle size 40-63 micron). Melting points were recorded on a Gallenkamp melting point apparatus and are uncorrected. The solvent of recrystallization is reported in parentheses. Infrared (IR) spectra were recorded on Bruker platinum alpha FTIR spectrometer on the neat compound using the attenuated total refraction technique. NMR spectra were acquired on Bruker Ascend 400 or Ascend 500 spectrometers. ^{1}H and ^{13}C NMR spectra were referenced to external tetramethylsilane *via* the residual protonated solvent (^{1}H) or the solvent itself (^{13}C). ^{11}B NMR spectra were referenced through the solvent lock (^{2}H) signal according to the IUPAC-recommended secondary referencing method following Bruker protocols. All chemical shifts are reported in parts per million (ppm). For $CDCl_3$, the shifts are referenced to 7.26 ppm for ^{1}H NMR spectroscopy and 77.16 ppm for ^{13}C NMR spectroscopy. For DMSO-*d*6, the shifts are referenced to 2.50 ppm for ^{1}H NMR spectroscopy and 39.52 ppm for ^{13}C NMR spectroscopy. Coupling constants (*J*) are quoted to the nearest 0.1 Hz. High-resolution mass spectra were recorded using electrospray ionization (ESI) techniques. X-ray diffraction data were collected at 120 K on an Agilent SuperNova diffractometer using CuKα radiation.

2. Preparation of Substrates

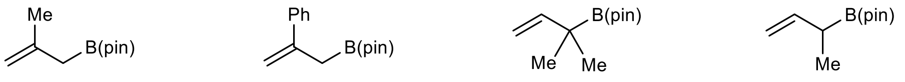

2a **2c** **2d** *rac*-**2e**

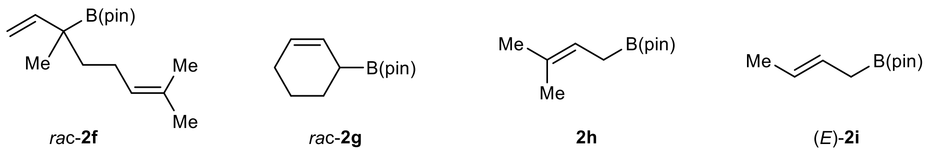

rac-**2f** *rac*-**2g** **2h** (*E*)-**2i**

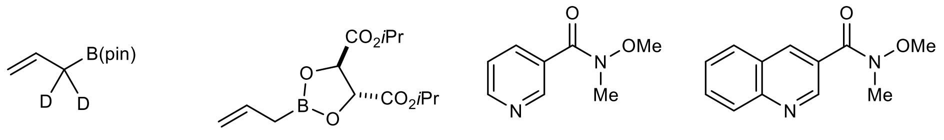

[D]$_2$-**2b** **2j** **S1** **S2**

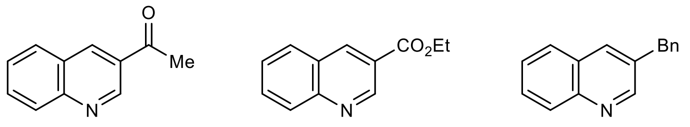

S3 **S4** **S5**

Allyl pinacolboronates **2a**,[1] **2c**,[1] **2d**,[1] *rac*-**2e**,[1] *rac*-**2f**,[2] *rac*-**2g**,[3] **2h**,[3] and (*E*)-**2i**[3] were prepared by reported procedures. Allyl pinacolboronate [D]$_2$-**2b** was prepared by a reported procedure,[4] and was contaminated with 15% vinylboronic acid pinacol ester, which is consistent with previously reported literature[4–6]. Allyl pinacolboronate **2j** was prepared by a reported procedure,[7] and was contaminated with 32% (+)-diisopropyl L-tartrate, which is consistent with previously reported literature.[7] Quinoline **S5**[8] was prepared by a reported procedure. The procedures for the preparation of **S1-S4** are described in detail in the following sections.

General Procedure A: Preparation of Azinium Ions

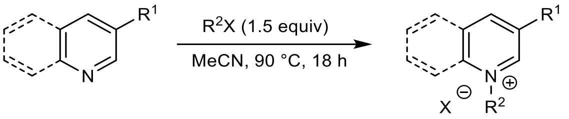

1a–1c, 1e, 1g–1q, 1sBr, 1sI, 1t–1w

Following a modification of a reported procedure,[9] a flask was charged with the appropriate substituted azine (1.0 equiv) and MeCN. The appropriate alkyl halide (1.5 equiv) was added in one portion and the resulting mixture was stirred at 90 °C for 18 h. The mixture was cooled to room temperature and transferred to a beaker which was cooled to 0 °C and stirred vigorously for 5 min. Et_2O (30 mL per mmol of azine) was added portionwise, and the mixture was stirred vigorously at 0 °C for 15 min, at which point the product precipitated. If no precipitation occurred, the mixture was sonicated until precipitation occurred. The precipitated product was removed by vacuum filtration, washed with ice-cold Et_2O (3 × 5 mL per mmol of azine) and dried under vacuum to give the desired product.

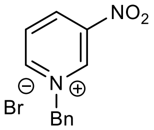

1-Benzyl-3-nitropyridin-1-ium bromide (1a). The title compound was prepared according to General Procedure A, using 3-nitropyridine (2.00 g, 16.1 mmol), benzyl bromide (2.88 mL, 24.2 mmol) and MeCN (30 mL) to give a dark yellow solid (3.35 g, 71%). m.p. 161-162 °C (Et_2O); IR 2982, 1639, 1528 (NO_2), 1455, 1353 (NO_2), 1125, 1030, 963, 731, 663 cm^{-1}; ^1H NMR (400 MHz, DMSO-*d6*) δ 10.36 (1H, s, Ar**H**), 9.52 (1H, dd, *J* = 6.2, 1.2 Hz, Ar**H**), 9.38-9.34 (1H, m, Ar**H**), 8.43 (1H, dd, *J* = 8.6, 6.1 Hz, Ar**H**), 7.63-7.59 (2H, m, Ar**H**), 7.49-7.44 (3H, m, Ar**H**), 6.06 (2H, s, NC**H**$_2$); ^{13}C NMR (101 MHz, DMSO-*d6*) δ 149.2 (CH), 146.7 (C), 142.5 (CH), 140.2 (CH), 133.6 (C), 129.6 (CH), 129.18 (2 × CH), 128.15 (CH), 129.1 (2 × CH), 63.8 (CH_2); HRMS (ESI) Exact mass calculated for $[C_{12}H_{11}N_2O_2]^+$: 215.0815, found 215.0808.

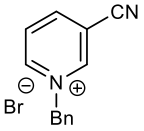

1-Benzyl-3-cyanopyridin-1-ium bromide (1b). The title compound was prepared according to General Procedure A, using 3-cyanopyridine (1.00 g, 9.61 mmol), benzyl bromide (1.71 mL, 14.4 mmol) and MeCN (15 mL) to give a pale pink solid (1.78 g, 68%). ^1H NMR (500 MHz, DMSO-*d6*) δ 10.05 (1H, s, Ar**H**), 9.47 (1H, dd, *J* = 6.3, 1.3 Hz, Ar**H**), 9.15-9.11 (1H, m, Ar**H**), 8.38 (1H, dd, *J* = 8.1, 6.2 Hz, Ar**H**), 7.63-7.60 (2H, m, Ar**H**), 7.48-7.44 (3H, m, Ar**H**), 5.93 (2H, s, NC**H**$_2$); ^{13}C NMR (126 MHz, DMSO-*d6*) δ 149.1 (CH), 149.0 (CH), 148.0 (CH), 133.4 (C), 129.6 (CH), 129.22 (2 × CH), 129.15 (2 × CH), 128.8 (CH), 113.9 (C), 113.2 (C), 63.9 (CH_2). Data consistent with previously reported literature.[9]

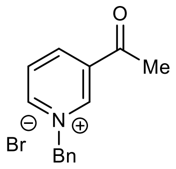

3-Acetyl-1-benzylpyridin-1-ium bromide (1c). The title compound was prepared according to General Procedure A, using 3-acetylpyridine (1.01 mL, 10.0 mmol), benzyl bromide (1.78 mL, 15.0 mmol) and MeCN (20 mL) to give an off-white white solid (2.86 g, 98%). ^{1}H NMR (400 MHz, DMSO-*d6*) δ 9.85 (1H, s, Ar**H**), 9.39 (1H, d, *J* = 6.0 Hz, Ar**H**), 9.04 (1H, dd, *J* = 8.1, 1.4 Hz, Ar**H**), 8.32 (1H, dd, *J* = 8.0, 6.1 Hz, Ar**H**), 7.63-7.59 (2H, m, Ar**H**), 7.48-7.41 (3H, m, Ar**H**), 6.03 (2H, s, NC**H**$_2$), 2.75 (3H, s, C**H**$_3$); ^{13}C NMR (101 MHz, DMSO-*d6*) δ 194.1 (C), 147.3 (CH), 145.4 (CH), 144.5 (CH), 135.8 (C), 134.1 (C), 129.4 (CH), 129.2 (2 × CH), 128.9 (2 × CH), 128.6 (CH), 63.3 (CH$_2$), 27.4 (CH$_3$). Data consistent with previously reported literature.[10]

1-Benzyl-3-(methoxycarbonyl)pyridin-1-ium bromide (1d)

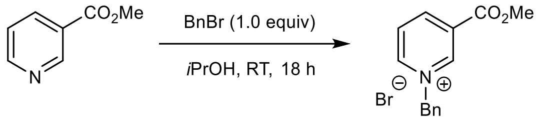

1d

Following a modification of a reported procedure,[11] a flask was charged with methyl nicotinate (1.37 g, 10.0 mmol) and *i*PrOH (5 mL). Benzyl bromide (1.19 mL, 10.0 mmol) was added dropwise, and the resulting mixture was stirred at room temperature for 18 h. MTBE (20 mL) was added in one portion and the mixture was sonicated until precipitation occurred. The product was removed by vacuum filtration and washed with MTBE (5 × 10 mL) and dried under vacuum to give the title compound (**1d**) as an off-white solid (2.58 g, 84%). ^{1}H NMR (400 MHz, CDCl$_3$) δ 10.08 (1H, dd, *J* = 6.2, 1.4 Hz, Ar**H**), 9.79 (1H, s, Ar**H**), 8.84 (1H, dt, *J* = 8.0, 1.5 Hz, Ar**H**), 8.25 (1H, dd, *J* = 8.1, 6.1 Hz, Ar**H**), 7.72-7.68 (2H, m, Ar**H**), 7.33-7.29 (3H, m, Ar**H**), 6.48 (2H, s, NC**H**$_2$), 3.92 (3H, s, C**H**$_3$); ^{13}C NMR (101 MHz, CDCl$_3$) δ 161.5 (C), 148.7 (CH), 145.5 (CH), 145.2 (CH), 132.6 (C), 130.4 (C), 130.2 (CH), 129.9 (2 × CH), 129.7 (2 × CH), 128.7 (CH), 64.5 (CH$_2$), 53.9 (CH$_3$). Data consistent with previously reported literature.[11]

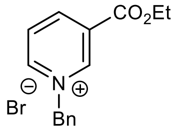

1-Benzyl-3-(ethoxycarbonyl)pyridin-1-ium bromide (1e). The title compound was prepared according to General Procedure A, using ethyl nicotinate (1.37 mL, 10.0 mmol), benzyl bromide (1.78 mL, 15.0 mmol) and MeCN (20 mL) to give an off-white solid (3.13 g, 97%). ^{1}H NMR (400 MHz, CDCl$_3$) δ 10.08-10.05 (1H, m, Ar**H**), 9.76 (1H, s, Ar**H**), 8.83-8.80 (1H, m, Ar**H**), 8.25 (1H, dd, *J* = 8.1, 6.1 Hz, Ar**H**), 7.70-7.67 (2H, m, Ar**H**), 7.32-7.28 (3H, m, Ar**H**), 6.47 (2H, s, NC**H**$_2$), 4.37 (2H, q, *J* = 7.1 Hz, C**H**$_2$CH$_3$), 1.34 (3H, t, *J* = 7.1 Hz, C**H**$_3$); ^{13}C NMR (101 MHz, CDCl$_3$) δ 161.0 (C), 148.6 (CH), 145.4 (CH), 145.1 (CH), 132.6 (C),

130.7 (C), 130.1 (CH), 129.9 (2 × CH), 129.6 (2 × CH), 128.7 (CH), 64.4 (CH_2), 63.4 (CH_2), 14.2 (CH_3). Data consistent with previously reported literature.[12]

1-Benzyl-3-(butoxycarbonyl)pyridin-1-ium bromide (1f)

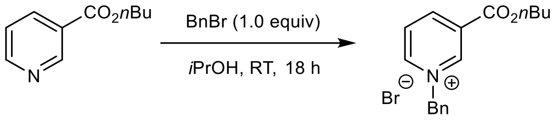

1f

Following a modification of a reported procedure,[11] a flask was charged with butyl nicotinate (1.71 mL, 10.0 mmol) and *i*PrOH (5 mL). Benzyl bromide (1.19 mL, 10.0 mmol) was added dropwise, and the resulting mixture was stirred at room temperature for 18 h. MTBE (20 mL) was added in one portion and the mixture was sonicated until precipitation occurred. The product was removed by vacuum filtration and washed with MTBE (5 × 10 mL) and dried under vacuum to give the title compound (**1f**) as an off-white solid (3.50 g, 100%). m.p. 96-97 °C (Et_2O); IR 3041, 3382, 2958, 1726 (C=O), 1629, 1294, 1209, 1105, 750, 708 cm^{-1}; ^{1}H NMR (400 MHz, $CDCl_3$) δ 10.12 (1H, dt, *J* = 6.3, 1.3 Hz, Ar**H**), 9.50 (1H, s, Ar**H**), 8.83 (1H, dt, *J* = 8.1, 1.5 Hz, Ar**H**), 8.29 (1H, dd, *J* = 8.1, 6.1 Hz, Ar**H**), 7.69-7.65 (2H, m, Ar**H**), 7.39-7.35 (3H, m, Ar**H**), 6.44 (2H, s, NC**H**$_2$), 4.35 (2H, t, *J* = 6.7 Hz, OC**H**$_2$), 1.75-1.70 (2H, m, C**H**$_2$$CH_2CH_3$), 1.44-1.34 (2H, m, CH_2C**H**$_2$$CH_3$), 0.92 (3H, t, *J* = 7.4 Hz, C**H**$_3$); ^{13}C NMR (101 MHz, $CDCl_3$) δ 161.1 (C), 149.1 (CH), 145.1 (CH), 145.0 (CH), 132.4 (C), 130.8 (C), 130.3 (CH), 130.1 (2 × CH), 129.8 (2 × CH), 128.8 (CH), 67.3 (CH_2), 65.0 (CH_2), 30.4 (CH_2), 19.1 (CH_2), 13.7 (CH_3); HRMS (ESI) Exact mass calculated for $[C_{17}H_{20}NO_2]^+$: 270.1489, found 270.1496.

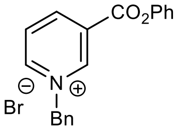

1-Benzyl-3-(phenoxycarbonyl)pyridin-1-ium bromide (1g). The title compound was prepared according to General Procedure A, using phenyl nicotinate (498 mg, 2.50 mmol), benzyl bromide (446 µL, 3.75 mmol) and MeCN (5 mL) to give an off-white solid (750 mg, 81%). m.p. 158-159 °C (Et_2O); IR 3060, 1751 (C=O), 1632, 1585, 1285, 1189, 1088, 729, 694, 668 cm^{-1}; ^{1}H NMR (400 MHz, $CDCl_3$) δ 10.05-10.03 (2H, m, Ar**H**), 8.93 (1H, d, *J* = 7.9 Hz, Ar**H**), 8.25 (1H, dd, *J* = 8.1, 6.0 Hz, Ar**H**), 7.74-7.71 (2H, m, Ar**H**), 7.39-7.31 (5H, m, Ar**H**), 7.28-7.23 (3H, m, Ar**H**), 6.50 (2H, s, NC**H**$_2$); ^{13}C NMR (101 MHz, $CDCl_3$) δ 159.9 (C), 150.0 (C), 149.0 (CH), 146.1 (CH), 145.6 (CH), 132.6 (C), 130.2 (CH), 130.1 (2 × CH and C), 129.78 (2 × CH), 129.76 (2 × CH), 128.8 (CH), 126.9 (CH), 121.4 (2 × CH), 64.6 (CH_2); HRMS (ESI) Exact mass calculated for $[C_{19}H_{16}NO_2]^+$: 290.1176, found 290.1180.

Preparation of substrate 1h

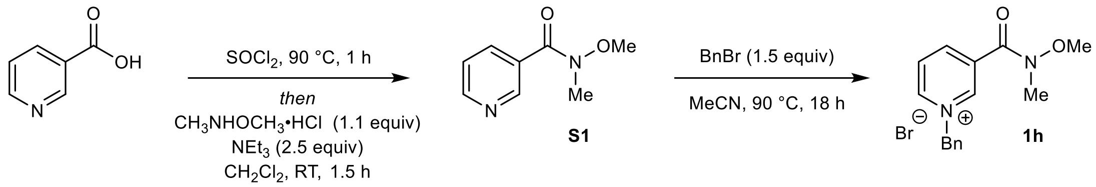

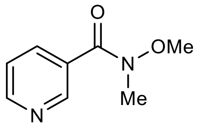

***N*-Methoxy-*N*-methylnicotinamide (S1).** A flask was charged with nicotinic acid (1.48 g, 12.0 mmol). Thionyl chloride (10.0 mL) was added in one portion and the reaction was stirred at 90 °C for 1 h. The reaction was cooled to room temperature and concentrated *in vacuo* to leave a residue, which was dissolved in anhydrous CH_2Cl_2 (10 mL). *N*,*O*-Dimethylhydroxylamine (1.29 g, 13.2 mmol) was added, followed by the dropwise addition of triethylamine (4.18 mL, 30.0 mmol) at 0 °C. The reaction mixture was stirred at room temperature for 1.5 h. H_2O (30 mL) was added and the resulting mixture was extracted with CH_2Cl_2 (3 × 50 mL), and the combined organic layers were dried ($MgSO_4$), filtered, and concentrated *in vacuo* to give the title compound (**S1**) as a brown oil (1.94 g, 97%). ^{1}H NMR (400 MHz, $CDCl_3$) δ 8.95-8.93 (1H, m, Ar**H**), 8.67 (1H, dd, *J* = 4.9, 1.7 Hz, Ar**H**), 8.03 (1H, dt, *J* = 8.0, 2.0 Hz, Ar**H**), 7.36 (1H, dd, *J* = 8.0, 4.9 Hz, Ar**H**), 3.54 (3H, s, C**H**$_3$), 3.38 (3H, s, C**H**$_3$); ^{13}C NMR (101 MHz, $CDCl_3$) δ 167.4 (C), 151.2 (CH), 149.2 (CH), 136.5 (CH), 130.1 (C), 123.2 (CH), 61.4 (CH_3), 33.3 (CH_3). Data consistent with previously reported literature.[13]

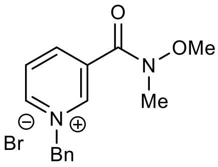

1-Benzyl-3-(methoxy(methyl)carbamoyl)pyridin-1-ium bromide (1h). The title compound was prepared according to General Procedure A, using **S1** (831 mg, 5.0 mmol), benzyl bromide (892 µL, 7.5 mmol) and MeCN (10 mL) to give a light brown solid (1.39 g, 82%). m.p. 144-145 °C (Et_2O); IR 3030, 2994, 1646 (C=O), 1627, 1462, 1199, 982, 772, 715, 678 cm^{-1}; ^{1}H NMR (400 MHz, $CDCl_3$) δ 9.99 (1H, d, *J* = 5.9 Hz, Ar**H**), 9.37 (1H, s, Ar**H**), 8.70 (1H, d, *J* = 7.9 Hz, Ar**H**), 8.17 (1H, dd, *J* = 8.1, 6.0 Hz, Ar**H**), 7.70-7.65 (2H, m, Ar**H**), 7.40-7.36 (3H, m, Ar**H**), 6.44 (2H, s, NC**H**$_2$), 3.62 (3H, s, C**H**$_3$), 3.36 (3H, s, C**H**$_3$); ^{13}C NMR (101 MHz, $CDCl_3$) δ 161.8 (C), 147.0 (CH), 145.1 (CH), 144.5 (CH), 133.9 (C), 132.6 (C), 130.3 (CH), 130.0 (2 × CH), 129.9 (2 × CH), 128.2 (CH), 64.9 (CH_2), 62.7 (CH_3), 33.2 (CH_3); HRMS (ESI) Exact mass calculated for $[C_{15}H_{17}N_2O_2]^+$: 257.1285, found 257.1288.

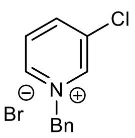

1-Benzyl-3-chloropyridin-1-ium bromide (1i). The title compound was prepared according to General Procedure A, using 3-chloropyridine (951 µL, 10.0 mmol), benzyl bromide (1.78 mL, 15.0 mmol) and MeCN (20 mL) to give an off-white solid (1.99 g, 70%). m.p. 137-138 °C (Et_2O); IR 3004, 1621, 1492, 1329, 1169, 1131, 772, 741, 715, 694 cm^{-1}; ^{1}H

NMR (400 MHz, $CDCl_3$) δ 9.77 (1H, d, *J* = 6.1 Hz, Ar**H**), 9.72 (1H, s, Ar**H**), 8.37 (1H, dd, *J* = 8.7, 1.7 Hz, Ar**H**), 8.09 (1H, dd, *J* = 8.4, 6.1 Hz Ar**H**), 7.77-7.73 (2H, m, Ar**H**), 7.39-7.35 (3H, m, Ar**H**), 6.45 (2H, s, NC**H**$_2$); ^{13}C NMR (101 MHz, $CDCl_3$) δ 145.2 (CH), 144.0 (CH), 143.9 (CH), 135.9 (C), 132.6 (C), 130.4 (CH), 130.1 (2 × CH), 129.8 (2 × CH), 129.0 (CH), 64.4 (CH_2); HRMS (ESI) Exact mass calculated for $[C_{12}H_{11}ClN]^+$: 204.0575, found 204.0577.

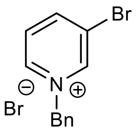

1-Benzyl-3-bromopyridin-1-ium bromide (1j). The title compound was prepared according to General Procedure A, using 3-bromopyridine (963 µL, 10.0 mmol), benzyl bromide (1.78 mL, 15.0 mmol) and MeCN (20 mL) to give an off-white solid (3.29 g, 100%). ^{1}H NMR (400 MHz, DMSO-*d*6) δ 9.74 (1H, s, Ar**H**), 9.25 (1H, d, *J* = 6.2 Hz, Ar**H**), 8.91 (1H, ddd, *J* = 8.7, 1.9, 1.1 Hz, Ar**H**), 8.13 (1H, dd, *J* = 8.4, 6.2 Hz Ar**H**), 7.62-7.58 (2H, m, Ar**H**), 7.48-7.41 (3H, m, Ar**H**), 5.87 (2H, s, NC**H**$_2$); ^{13}C NMR (101 MHz, DMSO-*d*6) δ 148.4 (CH), 146.0 (CH), 143.8 (CH), 133.8 (C), 129.5 (CH), 129.3 (CH), 129.2 (2 × CH), 129.0 (2 × CH), 122.4 (C), 63.4 (CH_2). Data consistent with previously reported literature.[14]

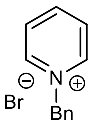

1-Benzylpyridin-1-ium bromide (1k). The title compound was prepared according to General Procedure A, using pyridine (809 µL, 10.0 mmol), benzyl bromide (1.78 mL, 15.0 mmol) and MeCN (20 mL) to give an off-white solid (2.17 g, 87%). ^{1}H NMR (400 MHz, $CDCl_3$) δ 9.61 (2H, d, *J* = 5.9 Hz, Ar**H**), 8.43 (1H, t, *J* = 7.7 Hz, Ar**H**), 8.00 (2H, t, *J* = 7.0 Hz, Ar**H**), 7.68-7.64 (2H, m, Ar**H**), 7.28-7.25 (3H, m, Ar**H**), 6.26 (2H, s, NC**H**$_2$); ^{13}C NMR (101 MHz, $CDCl_3$) δ 145.4 (CH), 145.0 (2 × CH), 133.0 (C), 129.9 (CH), 129.53 (2 × CH), 129.50 (2 × CH), 128.3 (2 × CH), 63.8 (CH_2). Data consistent with previously reported literature.[15]

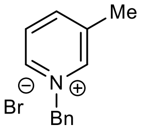

1-Benzyl-3-methylpyridin-1-ium bromide (1l). The title compound was prepared according to General Procedure A, using 3-methylpyridine (973 µL, 10.0 mmol), benzyl bromide (1.78 mL, 15.0 mmol) and MeCN (20 mL) to give an off-white solid (1.88 g, 71%). m.p. 104-105 °C (Et_2O); IR 3450, 3388, 3016, 1625, 1499, 1205, 1146, 775, 730, 707 cm^{-1}; ^{1}H NMR (400 MHz, $CDCl_3$) δ 9.55 (1H, s, Ar**H**), 9.40 (1H, d, *J* = 6.1 Hz, Ar**H**), 8.16 (1H, d, *J* = 7.9 Hz, Ar**H**), 7.88 (1H, dd, *J* = 7.9, 6.1 Hz, Ar**H**), 7.72-7.67 (2H, m, Ar**H**), 7.32-7.28 (3H, m, Ar**H**), 6.21 (2H, s, NC**H**$_2$), 2.53 (3H, s, C**H**$_3$); ^{13}C NMR (101 MHz, $CDCl_3$) δ 145.8 (CH), 144.6 (CH), 142.2 (CH), 139.7 (C), 133.2 (C), 129.9 (CH), 129.7 (2 × CH), 129.6 (2 × CH), 127.7 (CH), 63.7 (CH_2), 18.8 (CH_3); HRMS (ESI) Exact mass calculated for $[C_{13}H_{14}N]^+$: 184.1121, found 184.1128.

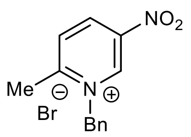

1-Benzyl-2-methyl-5-nitropyridin-1-ium bromide (1m). The title compound was prepared according to General Procedure A, using 2-methyl-5-nitropyridine (1.38 g, 10.0 mmol), benzyl bromide (1.78 mL, 15.0 mmol) and MeCN (20 mL) to give a black solid (359 mg, 12%). m.p. 149-151 °C (Et_2O); IR 2961, 1640, 1582, 1536 (NO_2), 1356 (NO_2), 1293, 812, 740, 732, 553 cm^{-1}; ^{1}H NMR (400 MHz, DMSO-*d6*) δ 10.25 (1H, s, Ar**H**), 9.33 (1H, d, *J* = 8.7 Hz, Ar**H**), 8.37 (1H, d, *J* = 8.7 Hz, Ar**H**), 7.48-7.34 (5H, m, Ar**H**), 6.13 (2H, s, NC**H**$_2$), 2.84 (3H, s, C**H**$_3$); ^{13}C NMR (101 MHz, DMSO-*d6*) δ 161.4 (C), 145.1 (C), 143.7 (CH), 139.5 (CH), 132.3 (C), 131.0 (CH), 129.1 (2 × CH), 129.0 (CH), 127.7 (2 × CH), 61.4 (CH_2), 20.4 (CH_3); HRMS (ESI) Exact mass calculated for $[C_{13}H_{13}N_2O_2]^+$: 229.0972, found 229.0980.

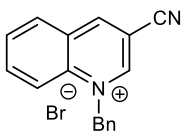

1-Benzyl-3-cyanoquinolin-1-ium bromide (1n). The title compound was prepared according to General Procedure A, using 3-quinolinecarbonitrile (771 mg, 5.00 mmol), benzyl bromide (892 µL, 7.50 mmol) and MeCN (10 mL) to give a light green solid (988 mg, 61%). m.p. 181-182 °C (Et_2O); IR 2947, 2246 (C≡N), 1627, 1381, 1249, 1163, 1051, 752, 731, 698 cm^{-1}; ^{1}H NMR (400 MHz, DMSO-*d6*) δ 10.44 (1H, d, *J* = 1.7 Hz, Ar**H**), 10.03 (1H, d, *J* = 1.7 Hz, Ar**H**), 8.57-8.53 (2H, m, Ar**H**), 8.37 (1H, ddd, *J* = 8.8, 7.0, 1.5 Hz, Ar**H**), 8.14 (1H, t, *J* = 7.6, Ar**H**), 7.53-7.50 (2H, m, Ar**H**), 7.42-7.34 (3H, m, Ar**H**), 6.42 (2H, s, NC**H**$_2$); ^{13}C NMR (101 MHz, DMSO-*d6*) δ 152.6 (2 × CH), 138.7 (CH), 137.9 (C), 133.2 (C), 131.9 (CH), 131.3 (CH), 129.0 (2 × CH), 128.9 (CH), 128.8 (C), 127.5 (2 × CH), 119.8 (CH), 114.6 (C), 107.2 (C), 60.8 (CH_2); HRMS (ESI) Exact mass calculated for $[C_{17}H_{13}N_2]^+$: 245.1073, found 245.1072.

Preparation of substrate 1o

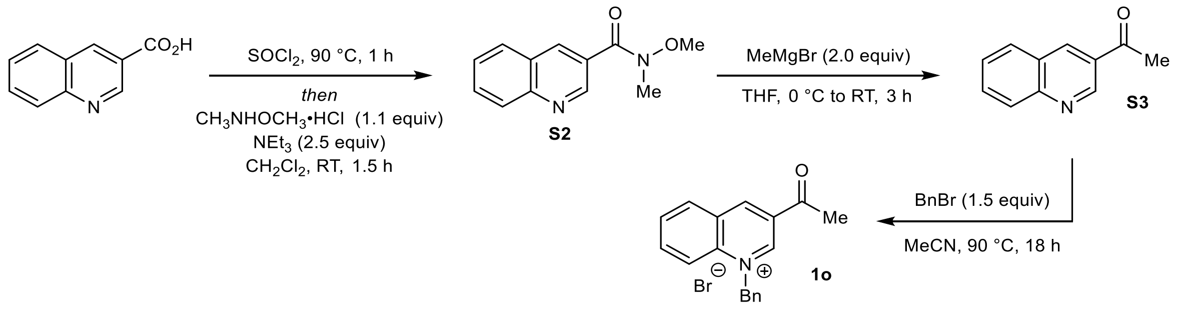

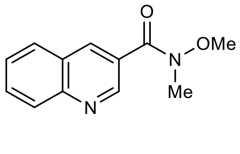

***N*-Methoxy-*N*-methylquinoline-3-carboxamide (S2)**. A flask was charged with 3-quinolinecarboxylic acid (3.46 g, 20.0 mmol). Thionyl chloride (20.0 mL) was added in one portion and the reaction was stirred at 90 °C for 1 h. The reaction was cooled to room temperature and concentrated *in vacuo* to leave a residue, which was dissolved in anhydrous CH_2Cl_2 (20 mL). *N*,*O*-Dimethylhydroxylamine (2.15 g, 22.0 mmol) was added, followed by the dropwise addition of triethylamine (6.97 mL, 50.0 mmol) at 0 °C. The reaction

mixture was stirred at room temperature for 1.5 h. H_2O (50 mL) was added and the resulting mixture was extracted with CH_2Cl_2 (3 × 100 mL), and the combined organic layers were dried ($MgSO_4$), filtered, and concentrated *in vacuo*. Purification of the residue by column chromatography (100% EtOAc) gave the title compound (**S2**) as a brown solid (2.99 g, 69%). ^{1}H NMR (400 MHz, $CDCl_3$) δ 9.21 (1H, d, *J* = 2.1 Hz, Ar**H**), 8.56 (1H, d, *J* = 2.1 Hz, Ar**H**), 8.13 (1H, dd, *J* = 8.5, 1.2 Hz, Ar**H**), 7.88 (1H, dd, *J* = 8.2, 1.5 Hz, Ar**H**), 7.78 (1H, ddd, *J* = 8.5, 6.9, 1.5 Hz, Ar**H**), 7.59 (1H, ddd, *J* = 8.2, 6.9, 1.2 Hz, Ar**H**), 3.56 (3H, s, C**H**$_3$), 3.43 (3H, s, C**H**$_3$); ^{13}C NMR (101 MHz, $CDCl_3$) δ 167.5 (C), 149.5 (CH), 148.7 (C), 137.1 (CH), 131.1 (CH), 129.4 (CH), 128.8 (CH), 127.3 (CH), 127.1 (C), 127.0 (C), 61.5 (CH_3), 33.4 (CH_3). Data consistent with previously reported literature.[16]

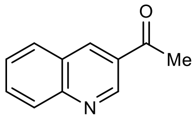

1-(Quinolin-3-yl)ethan-1-one (S3). A flask was charged with Weinreb amide **S2** (1.08 g, 5.00 mmol), purged with argon for 30 min, followed by the addition of THF (5 mL) at 0 °C. MeMgBr (3.0 M in Et_2O, 3.33 mL, 10.0 mmol) was added dropwise, and the resulting solution was warmed to room temperature and stirred for 3 h. The reaction was quenched with saturated aqueous NH_4Cl solution (10 mL) at 0 °C and extracted with EtOAc (3 × 15 mL). The combined organic layers were washed with H_2O (10 mL), brine (10 mL), dried ($MgSO_4$), filtered, and concentrated *in vacuo* to give the title compound (**S3**) as a brown solid (790 mg, 92%). ^{1}H NMR (400 MHz, $CDCl_3$) δ 9.41 (1H, d, *J* = 2.2 Hz, Ar**H**), 8.69 (1H, d, *J* = 2.2 Hz, Ar**H**), 8.14 (1H, dd, *J* = 8.5, 1.2 Hz, Ar**H**), 7.93 (1H, dd, *J* = 8.2, 1.5 Hz, Ar**H**), 7.82 (1H, ddd, *J* = 8.5, 6.9, 1.5 Hz, Ar**H**), 7.61 (1H, ddd, *J* = 8.2, 6.9, 1.2 Hz, Ar**H**), 2.73 (3H, s, C**H**$_3$); ^{13}C NMR (101 MHz, $CDCl_3$) δ 196.8 (C), 149.9 (C), 149.3 (CH), 137.4 (CH), 132.1 (CH), 129.6 (CH), 129.5 (CH), 129.4 (C), 127.7 (CH), 126.9 (C), 26.9 (CH_3). Data consistent with previously reported literature.[17]

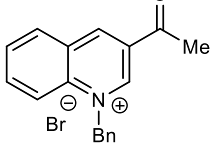

3-Acetyl-1-benzylquinolin-1-ium bromide (1o). The title compound was prepared according to General Procedure A, using **S3** (514 mg, 3.00 mmol), benzyl bromide (535 µL, 4.50 mmol) and MeCN (6 mL) to give a brown solid (844 mg, 82%). m.p. 232-233 °C (Et_2O); IR 2958, 1688 (C=O), 1626, 1361, 1236, 1200, 1019, 776, 742, 699 cm^{-1}; ^{1}H NMR (400 MHz, DMSO-*d6*) δ 10.26 (1H, d, *J* = 1.7 Hz, Ar**H**), 10.01 (1H, d, *J* = 1.7 Hz, Ar**H**), 8.69 (1H, dd, *J* = 8.3, 1.4 Hz, Ar**H**), 8.53 (1H, d, *J* = 8.9 Hz, Ar**H**), 8.31 (1H, ddd, *J* = 8.9, 7.0, 1.4 Hz, Ar**H**), 8.10 (1H, t, *J* = 7.6 Hz, Ar**H**), 7.45-7.33 (5H, m, Ar**H**), 6.54 (2H, s, NC**H**$_2$), 2.89 (3H, s, C**H**$_3$); ^{13}C NMR (101 MHz, DMSO-*d6*) δ 194.3 (C), 150.2 (CH), 147.7 (CH), 138.1 (C), 137.6 (CH), 133.8 (C), 132.5 (CH), 130.7 (CH), 130.2 (C), 129.2 (C), 129.0 (2 × CH), 128.8 (CH), 127.3 (2 × CH), 119.6 (CH), 60.3 (CH_2), 27.3 (CH_3); HRMS (ESI) Exact mass calculated for $[C_{18}H_{16}NO]^+$: 262.1226, found 262.1227.

Preparation of substrate 1p

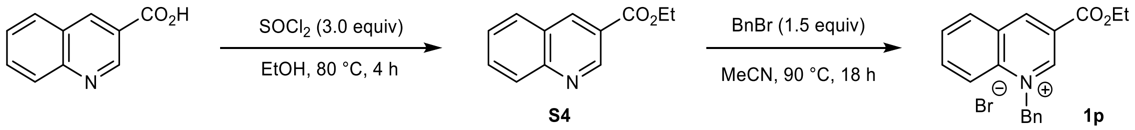

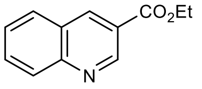

Ethyl quinoline-3-carboxylate (S4). To a stirred solution of 3-quinolinecarboxylic acid (1.73 g, 10.0 mmol) in EtOH (40 mL) was added $SOCl_2$ (2.19 mL, 30.0 mmol) dropwise at room temperature. The mixture was stirred at 80 °C for 4 h. The reaction was cooled to room temperature and concentrated *in vacuo*. The residue was dissolved in EtOAc (40 mL), washed with $NaHCO_3$ (40 mL), brine (40 mL), dried (Na_2SO_4) and concentrated *in vacuo* to afford the title compound (**S4**) as a brown solid (1.76 g, 88%). ^{1}H NMR (400 MHz, $CDCl_3$) δ 9.45 (1H, d, *J* = 2.1 Hz, Ar**H**), 8.83 (1H, d, *J* = 2.1 Hz, Ar**H**), 8.16 (1H, dd, *J* = 8.5, 1.0 Hz, Ar**H**), 7.93 (1H, dd, *J* = 8.2, 1.5 Hz, Ar**H**), 7.82 (1H, ddd, *J* = 8.5, 6.9, 1.5 Hz, Ar**H**), 7.61 (1H, ddd, *J* = 8.2, 6.9, 1.2 Hz, Ar**H**), 4.47 (2H, q, *J* = 7.1 Hz, C**H**$_2$CH$_3$), 1.46 (3H, t, *J* = 7.1 Hz, C**H**$_3$); ^{13}C NMR (101 MHz, $CDCl_3$) δ 165.5 (C), 150.2 (CH), 149.9 (C), 138.8 (CH), 131.9 (CH), 129.6 (CH), 129.2 (CH), 127.5 (CH), 127.0 (C), 123.4 (C), 61.6 (CH_2), 14.5 (CH_3). Data consistent with previously reported literature.[18]

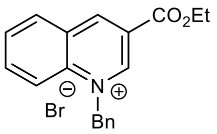

1-Benzyl-3-(ethoxycarbonyl)quinolin-1-ium bromide (1p). The title compound was prepared according to General Procedure A, using **S4** (1.01 g, 5.00 mmol), benzyl bromide (892 µL, 7.50 mmol) and MeCN (10 mL) to give a light brown solid (1.45 g, 78%). ^{1}H NMR (400 MHz, $CDCl_3$) δ 10.51 (1H, s, Ar**H**), 9.54 (1H, s, Ar**H**), 8.81 (1H, d, *J* = 8.9 Hz, Ar**H**), 8.40-8.36 (1H, m, Ar**H**), 8.25 (1H, ddd, *J* = 8.8, 7.0, 1.4 Hz, Ar**H**), 7.97 (1H, t, *J* = 7.6 Hz, Ar**H**), 7.49-7.45 (2H, m, Ar**H**), 7.36-7.30 (3H, m, Ar**H**), 6.91 (2H, s, NC**H**$_2$), 4.54 (2H, q, *J* = 7.1 Hz, C**H**$_2$CH$_3$), 1.51 (3H, t, *J* = 7.1 Hz, CH$_2$C**H**$_3$); ^{13}C NMR (101 MHz, $CDCl_3$) δ 161.6 (C), 150.0 (CH), 148.1 (CH), 139.7 (C), 138.5 (CH), 132.5 (C), 132.0 (CH), 131.3 (CH), 129.64 (2 × CH), 129.59 (CH), 129.3 (C), 128.2 (2 × CH), 125.0 (C), 120.7 (CH), 63.7 (CH_2), 62.1 (CH_2), 14.5 (CH_3). Data consistent with previously reported literature.[19]

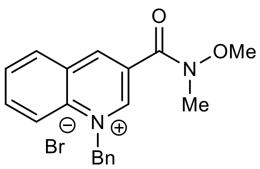

1-Benzyl-3-(methoxy(methyl)carbamoyl)quinolin-1-ium bromide (1q). The title compound was prepared according to General Procedure A, using **S2** (1.08 g, 5.0 mmol), benzyl bromide (892 µL, 7.5 mmol) and MeCN (10 mL) to give an off-white solid (1.53 g, 79%). m.p. 193-194 °C (Et_2O); IR 3036, 2879, 1668 (C=O), 1383, 1363, 1163, 1034, 753, 731, 699 cm^{-1}; ^{1}H NMR (400 MHz, $CDCl_3$) δ 10.19 (1H, s, Ar**H**), 9.37 (1H, s, Ar**H**), 8.71 (1H, d, *J* = 9.0 Hz, Ar**H**), 8.37 (1H, dd, *J* = 8.3, 1.4 Hz, Ar**H**), 8.16 (1H, ddd, *J* = 8.8, 7.0, 1.4 Hz, Ar**H**), 7.90 (1H, t, *J* = 7.6, Ar**H**), 7.49-7.45 (2H, m, Ar**H**), 7.31-

7.26 (3H, m, Ar**H**), 6.77 (2H, s, NC**H**$_2$), 3.75 (3H, s, C**H**$_3$), 3.39 (3H, s, C**H**$_3$); ^{13}C NMR (101 MHz, $CDCl_3$) δ 162.2 (C), 149.7 (CH), 147.2 (CH), 138.4 (C), 137.6 (CH), 132.4 (C), 131.8 (CH), 130.8 (CH), 129.51 (2 × CH), 129.50 (CH), 129.2 (C), 128.3 (2 × CH), 127.8 (C), 120.0 (CH), 62.8 (CH_2), 61.6 (CH_3), 33.3 (CH_3); HRMS (ESI) Exact mass calculated for $[C_{19}H_{19}N_2O_2]^+$: 307.1441, found 307.1448.

1-Methyl-3-nitropyridin-1-ium iodide (1r)

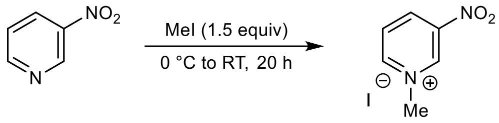

1r

A flask was charged with 3-nitropyridine (745 mg, 6.00 mmol) and purged with Ar for 30 min. Methyl iodide (560 µL, 9.00 mmol) was added dropwise at 0 °C and the reaction was stirred at room temperature for 20 h. The mixture was transferred to a beaker that was cooled to 0 °C and stirred vigorously for 5 min. Et_2O (200 mL) was added portionwise, and the mixture was stirred vigorously at 0 °C for 15 min, at which point the product precipitated. The precipitated product was removed by vacuum filtration and washed with ice-cold Et_2O (2 × 30 mL) and dried under vacuum to give the title compound (**1r**) as a yellow solid (494 mg, 31%). m.p. 223 °C (Et_2O); IR 3013, 1547 (NO_2), 1403, 1355 (NO_2), 1207, 1180, 1138, 803, 743, 652 cm^{-1}; ^{1}H NMR (400 MHz, DMSO-*d6*) δ 10.11 (1H, s, Ar**H**), 9.37-9.30 (2H, m, Ar**H**), 8.42 (1H, dd, *J* = 8.6, 6.1 Hz, Ar**H**), 4.50 (3H, s, C**H**$_3$); ^{13}C NMR (101 MHz, DMSO-*d6*) δ 150.3 (CH), 145.9 (C), 143.0 (CH), 139.5 (CH), 128.2 (CH), 48.7 (CH_3); HRMS (ESI) Exact mass calculated for $[C_6H_7N_2O_2]^+$: 139.0502, found 139.0508.

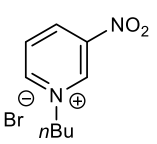

1-Butyl-3-nitropyridin-1-ium bromide (1sBr). The title compound was prepared according to General Procedure A, using 3-nitropyridine (621 mg, 5.00 mmol), *n*-butyl bromide (805 µL, 7.50 mmol) and MeCN (10 mL) to give a brown solid (163 mg, 12%). m.p. 181-182 °C (Et_2O); IR 3099, 2930, 1650, 1590, 1542 (NO_2), 1361 (NO_2), 1210, 1183, 809, 656 cm^{-1}; ^{1}H NMR (400 MHz, DMSO-*d6*) δ 10.20 (1H, s, Ar**H**), 9.50 (1H, d, *J* = 5.9 Hz, Ar**H**), 9.34 (1H, ddd, *J* = 8.7, 2.3, 1.1 Hz, Ar**H**), 8.44 (1H, dd, *J* = 8.6, 6.0 Hz, Ar**H**), 4.80 (2H, t, *J* = 7.5 Hz, NC**H**$_2$), 1.98-1.90 (2H, m, C**H**$_2$$CH_2CH_3$), 1.38-1.28 (2H, m, CH_2C**H**$_2$$CH_3$), 0.92 (3H, t, *J* = 7.4 Hz, C**H**$_3$); ^{13}C NMR (101 MHz, DMSO-*d6*) δ 149.3 (CH), 146.6 (C), 142.5 (CH), 139.7 (CH), 128.7 (CH), 61.4 (CH_2), 32.7 (CH_2), 18.7 (CH_2), 13.3 (CH_3); HRMS (ESI) Exact mass calculated for $[C_9H_{13}N_2O_2]^+$: 181.0972, found 181.0968.

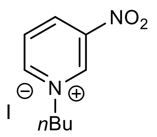

1-Butyl-3-nitropyridin-1-ium iodide (1sI). The title compound was prepared according to General Procedure A, using 3-nitropyridine (621 mg, 5.00 mmol), *n*-butyl iodide (854 μL, 7.50 mmol) and MeCN (10 mL) to give an orange solid (616 mg, 40%). m.p. 181-182 °C (Et_2O); IR 3103, 2988, 2934, 1648, 1542 (NO_2), 1359 (NO_2), 1182, 1131, 731, 694 cm^{-1}; ^1H NMR (400 MHz, DMSO-*d6*) δ 10.18 (1H, s, Ar**H**), 9.44 (1H, dt, *J* = 6.2, 1.2 Hz, Ar**H**), 9.36-9.32 (1H, m, Ar**H**), 8.43 (1H, dd, *J* = 8.6, 6.2 Hz, Ar**H**), 4.77 (2H, t, *J* = 7.5 Hz, NC**H**$_2$), 1.98-1.90 (2H, m, C**H**$_2$$CH_2CH_3$), 1.38-1.28 (2H, m, CH_2C**H**$_2$$CH_3$), 0.93 (3H, t, *J* = 7.4 Hz, C**H**$_3$); ^{13}C NMR (101 MHz, DMSO-*d6*) δ 149.3 (CH), 146.6 (C), 142.5 (CH), 139.7 (CH), 128.7 (CH), 61.5 (CH_2), 32.7 (CH_2), 18.7 (CH_2), 13.3 (CH_3); HRMS (ESI) Exact mass calculated for $[C_9H_{13}N_2O_2]^+$: 181.0972, found 181.0977.

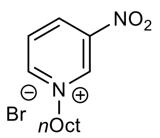

3-Nitro-1-octylpyridin-1-ium bromide (1t). The title compound was prepared according to General Procedure A, using 3-nitropyridine (621 mg, 5.00 mmol), *n*-octyl bromide (1.30 mL, 7.50 mmol) and MeCN (10 mL) to give a brown solid (169 mg, 11%). m.p. 110-111 °C (Et_2O); IR 3094, 2991, 2854, 1650, 1590, 1543 (NO_2), 1465, 1357 (NO_2), 731, 693 cm^{-1}; ^1H NMR (400 MHz, DMSO-*d6*) δ 10.19 (1H, s, Ar**H**), 9.49 (1H, d, *J* = 6.1 Hz, Ar**H**), 9.35-9.32 (1H, m, Ar**H**), 8.43 (1H, dd, *J* = 8.6, 6.0 Hz, Ar**H**), 4.78 (2H, t, *J* = 7.6 Hz, NC**H**$_2$), 1.99-1.91 (2H, m, NCH_2C**H**$_2$), 1.34-1.21 (10H, m, (C**H**$_2$)$_5$$CH_3$), 0.88-0.83 (3H, m, C**H**$_3$); ^{13}C NMR (101 MHz, DMSO-*d6*) δ 149.3 (CH), 146.6 (C), 142.4 (CH), 139.7 (CH), 128.7 (CH), 61.6 (CH_2), 31.1 (CH_2), 30.8 (CH_2), 28.42 (CH_2), 28.37 (CH_2), 25.3 (CH_2), 22.0 (CH_2), 13.9 (CH_3); HRMS (ESI) Exact mass calculated for $[C_{13}H_{21}N_2O_2]^+$: 237.1598, found 237.1600.

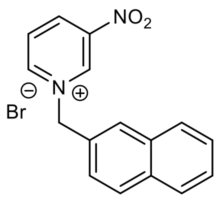

1-(Naphthalen-2-ylmethyl)-3-nitropyridin-1-ium bromide (1u). The title compound was prepared according to General Procedure A, using 3-nitropyridine (1.24 g, 10.0 mmol), 2-(bromomethyl)naphthalene (3.32 g, 15.0 mmol) and MeCN (20 mL) to give a yellow solid (2.72 g, 79%). ^1H NMR (400 MHz, DMSO-*d6*) δ 10.45 (1H, s, Ar**H**), 9.64 (1H, d, *J* = 6.2 Hz, Ar**H**), 9.40-9.36 (1H, m, Ar**H**), 8.45 (1H, dd, *J* = 8.6, 6.2 Hz, Ar**H**), 8.20 (1H, s, Ar**H**), 8.01 (1H, d, *J* = 8.5 Hz, Ar**H**), 7.98-7.92 (2H, m, Ar**H**), 7.72 (1H, dd, *J* = 8.5, 1.8 Hz, Ar**H**), 7.61-7.56 (2H, m, Ar**H**), 6.29 (2H, s, NC**H**$_2$); HRMS (ESI) Exact mass calculated for $[C_{16}H_{13}N_2O_2]^+$: 265.0972, found 265.0974. Data consistent with previously reported literature.[20]

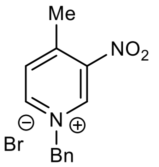

1-Benzyl-4-methyl-3-nitropyridin-1-ium bromide (1v). The title compound was prepared according to General Procedure A, using 4-methyl-3-nitropyridine (562 µL, 5.00 mmol), benzyl bromide (892 µL, 7.50 mmol) and MeCN (10 mL) to give an olive green solid (1.14 g, 74%). m.p. 157-158 °C (Et_2O); IR 2974, 1644, 1565, 1536 (NO_2), 1453, 1349 (NO_2), 1114, 1053, 743, 704 cm^{-1}; ^1H NMR (400 MHz, DMSO-*d6*) δ 10.17 (1H, s, Ar**H**), 9.38 (1H, dd, *J* = 6.4, 1.3 Hz, Ar**H**), 8.37 (1H, d, *J* = 6.4 Hz, Ar**H**), 7.63-7.60 (2H, m, Ar**H**), 7.48-7.42 (3H, m, Ar**H**), 6.00 (2H, s, $NC\mathbf{H}_2$), 2.85 (3H, s, $C\mathbf{H}_3$); ^{13}C NMR (101 MHz, DMSO-*d6*) δ 154.4 (C), 146.8 (C), 146.4 (CH), 142.7 (CH), 133.7 (C), 131.9 (CH), 129.5 (CH), 129.14 (2 × CH), 129.05 (2 × CH), 63.0 (CH_2), 20.7 (CH_3); HRMS (ESI) Exact mass calculated for $[C_{13}H_{13}N_2O_2]^+$: 229.0972, found 229.0977.

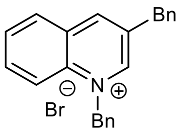

1,3-Dibenzylquinolin-1-ium bromide (1w). The title compound was prepared according to General Procedure A, using **S5** (329 mg, 1.50 mmol), benzyl bromide (268 µL, 2.25 mmol) and MeCN (5 mL) to give a beige solid (292 mg, 50%). m.p. 214-215 °C (Et_2O); IR 2949, 1585, 1528, 1492, 1370, 1217, 1027, 914, 743, 702 cm^{-1}; ^1H NMR (400 MHz, $CDCl_3$) δ 10.80 (1H, s, Ar**H**), 8.61 (1H, s, Ar**H**), 8.41 (1H, d, *J* = 8.9 Hz, Ar**H**), 8.07 (1H, d, *J* = 8.2 Hz, Ar**H**), 7.98 (1H, t, *J* = 8.0 Hz, Ar**H**), 7.78 (1H, t, *J* = 7.6 Hz, Ar**H**), 7.43-7.31 (6H, m, Ar**H**), 7.28-7.23 (4H, m, Ar**H**), 6.64 (2H, s, $NC\mathbf{H}_2$), 4.53 (2H, s, $CC\mathbf{H}_2C$); ^{13}C NMR (101 MHz, $CDCl_3$) δ 151.7 (CH), 145.7 (CH), 137.5 (C), 137.3 (C), 136.8 (C), 135.2 (CH), 132.9 (C), 130.3 (CH), 130.1 (CH), 130.0 (C), 129.6 (2 × CH), 129.5 (2 × CH), 129.4 (2 × CH), 129.3 (CH), 127.7 (2 × CH), 127.5 (CH), 119.2 (CH), 60.9 (CH_2), 38.4 (CH_2); HRMS (ESI) Exact mass calculated for $[C_{23}H_{20}N]^+$: 310.1590, found 310.1593.

3. Gold-Catalyzed Nucleophilic Allylation of Azinium Salts

General Procedure B: Gold-Catalyzed Allylation of Azinium Salts

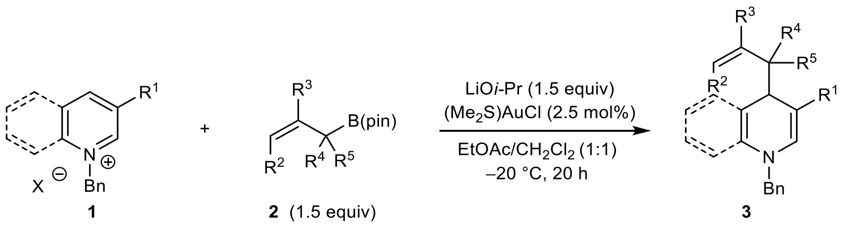

An oven-dried microwave vial fitted with a stirrer bar was charged with the appropriate azinium salt **1** (0.50 mmol), $(Me_2S)AuCl$ (3.7 mg, 0.0125 mmol) and LiO*i*-Pr (49.5 mg, 0.75 mmol). The vial was sealed with a septum-lined cap and $EtOAc/CH_2Cl_2$ (1:1) (4.0 mL) (both of which were undried, obtained from commercial vendors and used without further purification) was added. The mixture was stirred at –20 °C for 5 min and a solution of the appropriate allyl pinacolboronate **2** (0.75 mmol) in 1.0 mL of $EtOAc/CH_2Cl_2$ (1:1) was added dropwise. The resulting solution was stirred at –20 °C for 20 h. The reaction was warmed to room temperature and then passed through a plug of silica (8 cm in height and 2 cm wide) using Et_2O (40 mL) as the eluent and the filtrate was concentrated *in vacuo*. The residue was purified by column chromatography (EtOAc/*n*-pentane) to give the allylated product **3**.

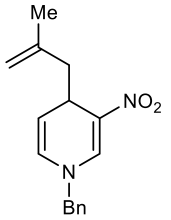

1-Benzyl-4-(2-methylallyl)-3-nitro-1,4-dihydropyridine (3aa). The title compound was prepared according to a slight modification of General Procedure A (in that the reaction time was 4.5 h), using azinium salt **1a** (148 mg, 0.50 mmol) and allyl pinacolboronate **2a** (137 mg, 0.75 mmol), and purified by column chromatography (5% EtOAc/*n*-pentane) to give a red solid (133 mg, 98%). R_f = 0.40 (20% EtOAc/petroleum ether); m.p. 69-70 °C (Et_2O); IR 2922, 1667, 1583 (NO_2), 1471, 1396 (NO_2), 1173, 1156, 1048, 741, 697 cm^{-1}; ^1H NMR (400 MHz, $CDCl_3$) δ 7.95 (1H, s, NC**H**=C), 7.42-7.33 (3H, m, Ar**H**), 7.24-7.20 (2H, m, Ar**H**), 5.86 (1H, dt, *J* = 7.9, 1.2 Hz, NC**H**=CH), 5.19 (1H, dd, *J* = 7.9, 5.0 Hz, NCH=C**H**), 4.82-4.80 (1H, m, C$\mathbf{H}_a$$H_b$=C), 4.65-4.63 (1H, m, C$H_a$$\mathbf{H}_b$=C), 4.49 (2H, s, NC$\mathbf{H}_2$), 3.99 (1H, ddd, *J* = 8.7, 5.0, 3.5 Hz, CH_2C**H**), 2.47-2.43 (1H, m, C$\mathbf{H}_a$$H_b$CH), 2.20 (1H, dd, *J* = 13.1, 8.8 Hz, C$H_a$$\mathbf{H}_b$CH), 1.76 (3H, s, =CC$\mathbf{H}_3$); ^{13}C NMR (101 MHz, $CDCl_3$) δ 141.8 (C), 140.3 (CH), 135.4 (C), 129.3 (2 × CH), 128.7 (CH), 127.4 (2 × CH), 126.4 (CH), 125.8 (C), 113.4 (CH_2), 113.1 (CH), 58.4 (CH_2), 44.3 (CH_2), 32.3 (CH), 22.8 (CH_3); HRMS (ESI) Exact mass calculated for $[C_{16}H_{18}N_2NaO_2]^+$ $[M+Na]^+$: 293.1260, found 293.1256.

Crystals suitable for X-ray analysis were prepared by slow diffusion of petroleum ether into a solution of **3aa** in CH_2Cl_2.

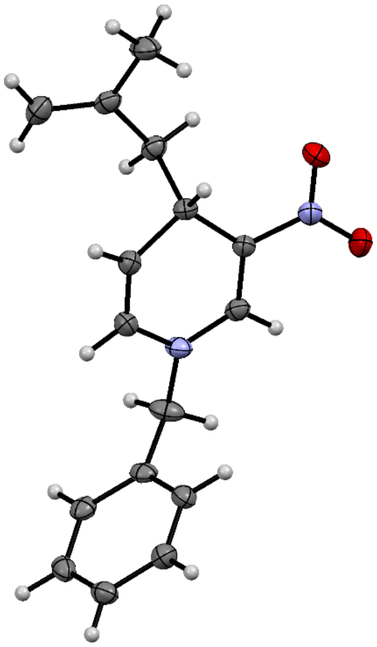

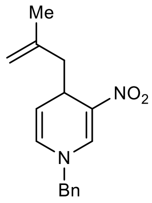

3aa
CCDC 2102857

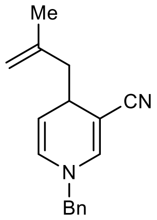

1-Benzyl-4-(2-methylallyl)-1,4-dihydropyridine-3-carbonitrile (3ba). The title compound was prepared according to General Procedure B, using azinium salt **1b** (138 mg, 0.50 mmol) and allyl pinacolboronate **2a** (137 mg, 0.75 mmol), and purified by column chromatography (3 to 5% EtOAc/*n*-pentane) to give a green oil (114 mg, 91%). R_f = 0.51 (20% EtOAc/petroleum ether); IR 3066, 2926, 2191 (C≡N), 1672, 1591, 1409, 1179, 1077, 889, 729 cm^{-1}; ^{1}H NMR (400 MHz, $CDCl_3$) δ 7.41-7.30 (3H, m, Ar**H**), 7.21-7.18 (2H, m, Ar**H**), 6.62 (1H, d, *J* = 1.7 Hz, NC**H**=C), 5.77 (1H, dt, *J* = 8.1, 1.3 Hz, NC**H**=CH), 4.86-4.84 (1H, m, C$\mathbf{H}_a$$H_b$=C), 4.74-4.69 (2H, m, $CH_a$$\mathbf{H}_b$=C and NCH=C**H**), 4.30 (2H, s, NC$\mathbf{H}_2$), 3.40-3.35 (1H, m, CH_2C**H**), 2.37 (1H, ddd, *J* = 13.5, 4.5, 1.1 Hz, C$\mathbf{H}_a$$H_b$CH), 2.23 (1H, ddd, *J* = 13.4, 9.0, 0.8 Hz, $CH_a$$\mathbf{H}_b$CH), 1.74 (3H, s, =CC$\mathbf{H}_3$); ^{13}C NMR (101 MHz, $CDCl_3$) δ 143.0 (CH), 141.4 (C), 136.5 (C), 129.1 (2 × CH), 128.3 (CH), 127.7 (CH), 127.2 (2 × CH), 121.4 (C), 113.6 (CH_2), 105.9 (CH), 83.1 (C), 57.5 (CH_2), 47.4 (CH_2), 31.7 (CH), 22.7 (CH_3); HRMS (ESI) Exact mass calculated for $[C_{17}H_{18}N_2Na]^+$ $[M+Na]^+$: 273.1362, found 273.1359.

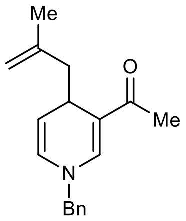

1-(1-Benzyl-4-(2-methylallyl)-1,4-dihydropyridin-3-yl)ethan-1-one (3ca). The title compound was prepared according to General Procedure B, using azinium salt **1c** (146 mg, 0.50 mmol) and allyl pinacolboronate **2a** (137 mg, 0.75 mmol), and purified by column chromatography (5 to 15% EtOAc/*n*-pentane) to give an orange oil (111 mg, 83%). R_f = 0.22 (20% EtOAc/petroleum ether); IR 3065, 2914, 1668

(C=O), 1614, 1570, 1384, 1208, 1177, 881, 729 cm^{-1}; ^{1}H NMR (400 MHz, $CDCl_3$) δ 7.41-7.36 (2H, m, Ar**H**), 7.34-7.29 (1H, m, Ar**H**), 7.24-7.21 (2H, m, Ar**H**), 7.11 (1H, d, *J* = 1.4 Hz, NC**H**=C), 5.85 (1H, dd, *J* = 7.8, 1.4 Hz, NC**H**=CH), 4.96 (1H, dd, *J* = 7.8, 5.1 Hz, NCH=C**H**), 4.76-4.73 (1H, m, C$\mathbf{H}_a$$H_b$=C), 4.61-4.59 (1H, m, C$H_a$$\mathbf{H}_b$=C), 4.43 (2H, s, NC$\mathbf{H}_2$), 3.69 (1H, dt, *J* = 9.1, 4.5 Hz, CH_2C**H**), 2.24-2.19 (1H, m, C$\mathbf{H}_a$$H_b$CH), 2.18 (3H, s, O=CC$\mathbf{H}_3$), 2.02 (1H, dd, *J* = 12.7, 9.3 Hz, C$H_a$$\mathbf{H}_b$CH), 1.77 (3H, s, =CC$\mathbf{H}_3$); ^{13}C NMR (101 MHz, $CDCl_3$) δ 195.1 (C), 143.0 (C), 142.7 (CH), 137.1 (C), 129.1 (2 × CH), 128.1 (CH), 127.3 (CH), 127.0 (2 × CH), 113.9 (C), 112.2 (CH_2), 109.8 (CH), 57.9 (CH_2), 46.9 (CH_2), 29.6 (CH), 24.7 (CH_3), 22.8 (CH_3); HRMS (ESI) Exact mass calculated for $[C_{18}H_{22}NO]^+$ $[M+H]^+$: 268.1696, found 268.1693.

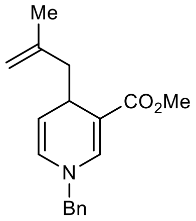

Methyl 1-benzyl-4-(2-methylallyl)-1,4-dihydropyridine-3-carboxylate (3da). The title compound was prepared according to General Procedure B, using azinium salt **1d** (154 mg, 0.50 mmol) and allyl pinacolboronate **2a** (137 mg, 0.75 mmol), and purified by column chromatography (3% EtOAc/*n*-pentane) to give a pale-yellow oil (106 mg, 75%). R_f = 0.48 (20% EtOAc/petroleum ether); IR 2945, 1685 (C=O), 1588, 1408, 1271, 1161, 1082, 884, 764, 729 cm^{-1}; ^{1}H NMR (400 MHz, $CDCl_3$) δ 7.38-7.33 (2H, m, Ar**H**), 7.32-7.27 (1H, m, Ar**H**), 7.24 (1H, d, *J* = 1.6 Hz, NC**H**=C), 7.23-7.19 (2H, m, Ar**H**), 5.81 (1H, dd, *J* = 7.8, 1.6 Hz, NC**H**=CH), 4.86 (1H, dd, *J* = 7.8, 5.0 Hz, NCH=C**H**), 4.77-4.75 (1H, m, C$\mathbf{H}_a$$H_b$=C), 4.64-4.61 (1H, m, C$H_a$$\mathbf{H}_b$=C), 4.37 (2H, s, NC$\mathbf{H}_2$), 3.68 (3H, s, OC$\mathbf{H}_3$), 3.54 (1H, dt, *J* = 9.0, 4.4 Hz, CH_2C**H**), 2.27 (1H, dd, *J* = 12.8, 3.8 Hz, C$\mathbf{H}_a$$H_b$CH), 2.08 (1H, dd, *J* = 12.8, 9.1 Hz, C$H_a$$\mathbf{H}_b$CH), 1.76 (3H, s, =CC$\mathbf{H}_3$); ^{13}C NMR (101 MHz, $CDCl_3$) δ 168.8 (C), 142.8 (C), 141.5 (CH), 137.4 (C), 129.0 (2 × CH), 127.9 (CH), 127.6 (CH), 127.1 (2 × CH), 112.3 (CH_2), 108.2 (CH), 101.9 (C), 57.7 (CH_2), 51.0 (CH_3), 47.9 (CH_2), 30.4 (CH), 22.7 (CH_3); HRMS (ESI) Exact mass calculated for $[C_{18}H_{21}NNaO_2]^+$ $[M+Na]^+$: 306.1464, found 306.1465.

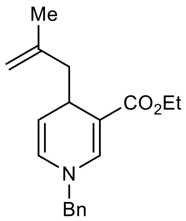

Ethyl 1-benzyl-4-(2-methylallyl)-1,4-dihydropyridine-3-carboxylate (3ea). The title compound was prepared according to General Procedure B, using azinium salt **1e** (161 mg, 0.50 mmol) and allyl pinacolboronate **2a** (137 mg, 0.75 mmol), and purified by column chromatography (3% EtOAc/*n*-pentane) to give a pale-yellow oil (133 mg, 89%). R_f = 0.55 (20% EtOAc/petroleum ether); IR 2977, 1680 (C=O), 1587, 1397, 1203, 1160, 1074, 885, 729, 697 cm^{-1}; ^{1}H NMR (500 MHz, $CDCl_3$) δ 7.38-7.34 (2H, m, Ar**H**), 7.32-7.28 (1H, m, Ar**H**), 7.25 (1H, d, *J* = 1.6 Hz, NC**H**=C), 7.23-7.20 (2H, m, Ar**H**), 5.80 (1H, dd, *J* = 7.8, 1.6 Hz, NC**H**=CH), 4.86 (1H, dd, *J* = 7.8, 5.0 Hz, NCH=C**H**), 4.77-4.75 (1H, m, C$\mathbf{H}_a$$H_b$=C), 4.64-4.62 (1H, m, C$H_a$$\mathbf{H}_b$=C), 4.38 (2H, s, NC$\mathbf{H}_2$), 4.21-4.12 (2H, m, OC$\mathbf{H}_2$), 3.54 (1H,

ddd, *J* = 9.0, 5.0, 3.8 Hz, $CH_2C\mathbf{H}$), 2.28 (1H, ddd, *J* = 12.9, 3.8, 1.2 Hz, $C\mathbf{H}_aH_bCH$), 2.09 (1H, ddd, *J* = 12.8, 9.2, 0.7 Hz, $CH_a\mathbf{H}_bCH$), 1.76 (3H, s, =$CC\mathbf{H}_3$), 1.27 (3H, t, *J* = 7.1 Hz, $CH_2C\mathbf{H}_3$); ^{13}C NMR (126 MHz, $CDCl_3$) δ 168.5 (C), 142.8 (C), 141.3 (CH), 137.5 (C), 129.0 (2 × CH), 127.9 (CH), 127.5 (CH), 127.1 (2 × CH), 112.3 (CH_2), 108.2 (CH), 102.1 (C), 59.5 (CH_2), 57.7 (CH_2), 48.0 (CH_2), 30.5 (CH), 22.7 (CH_3), 14.6 (CH_3); HRMS (ESI) Exact mass calculated for $[C_{19}H_{23}NNaO_2]^+$ $[M+Na]^+$: 320.1621, found 320.1616.

Gram-Scale Reaction:

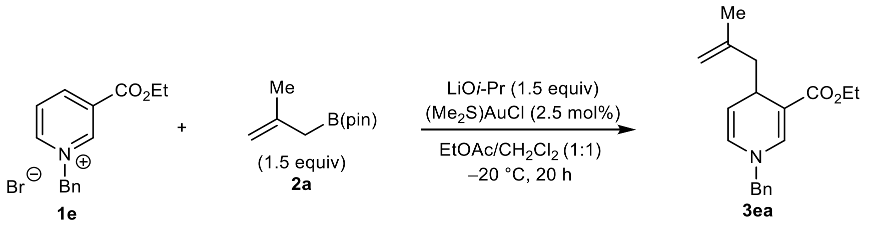

An oven-dried flask equipped with a stirrer bar was charged with azinium salt **1e** (1.29 g, 4.00 mmol), $(Me_2S)AuCl$ (29.5 mg, 0.10 mmol) and LiO-*i*Pr (396 mg, 6.00 mmol). The vial was sealed and $EtOAc/CH_2Cl_2$ (1:1) (20 mL) (both of which were undried, obtained from commercial vendors and used without further purification) was added. The mixture was stirred at –20 °C for 5 min and a solution of allyl pinacolboronate **2a** (1.09 g, 6.00 mmol) in 10 mL of $EtOAc/CH_2Cl_2$ (1:1) was added dropwise. The resulting solution was stirred at –20 °C for 20 h. The reaction was warmed to room temperature and then passed through a plug of silica (8 cm in height and 2 cm wide) using Et_2O (100 mL) as the eluent and the filtrate was concentrated *in vacuo*. The residue was purified by column chromatography using EtOAc/*n*-pentane (1 to 3% EtOAc/*n*-pentane) to give the title compound (**3ea**) as an orange oil (1.01 g, 85%).

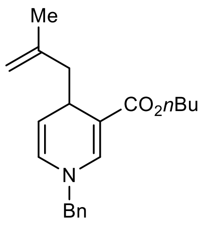

Butyl 1-benzyl-4-(2-methylallyl)-1,4-dihydropyridine-3-carboxylate (3fa). The title compound was prepared according to General Procedure B, using azinium salt **1f** (175 mg, 0.50 mmol) and allyl pinacolboronate **2a** (137 mg, 0.75 mmol), and purified by column chromatography (1 to 2% EtOAc/*n*-pentane) to give a brown oil (96.0 mg, 59%). R_f = 0.59 (20% EtOAc/petroleum ether); IR 2959, 1684 (C=O), 1588, 1455, 1271, 1162, 1044, 884, 730, 697 cm^{-1}; ^{1}H NMR (400 MHz, $CDCl_3$) δ 7.38-7.33 (2H, m, Ar**H**), 7.32-7.27 (1H, m, Ar**H**), 7.24 (1H, d, *J* = 1.6 Hz, NC**H**=C), 7.23-7.20 (2H, m, Ar**H**), 5.81 (1H, dd, *J* = 7.9, 1.6 Hz, NC**H**=CH), 4.86 (1H, dd, *J* = 7.8, 5.0 Hz, NCH=C**H**), 4.77-4.75 (1H, m, $C\mathbf{H}_aH_b$=C), 4.64-4.62 (1H, m, $CH_a\mathbf{H}_b$=C), 4.37 (2H, s, $NC\mathbf{H}_2$), 4.16-4.05 (2H, m, $OC\mathbf{H}_2$), 3.53 (1H, ddd, *J* = 9.0, 5.0, 3.8 Hz, $CH_2C\mathbf{H}$), 2.29 (1H, dd, *J* = 12.8, 3.7 Hz, $C\mathbf{H}_aH_bCH$), 2.09 (1H, dd, *J* = 12.8,

9.3 Hz, $CH_a\mathbf{H}_bCH$), 1.75 (3H, s, =CC**H**$_3$), 1.66-1.59 (2H, m, C**H**$_2CH_2CH_3$), 1.44-1.35 (2H, m, C**H**$_2CH_3$), 0.93 (3H, t, *J* = 7.4 Hz, $CH_2C\mathbf{H}_3$); ^{13}C NMR (101 MHz, $CDCl_3$) δ 168.6 (C), 142.8 (C), 141.3 (CH), 137.5 (C), 129.0 (2 × CH), 127.9 (CH), 127.5 (CH), 127.1 (2 × CH), 112.3 (CH_2), 108.2 (CH), 102.1 (C), 63.5 (CH_2), 57.7 (CH_2), 47.9 (CH_2), 31.1 (CH_2), 30.5 (CH), 22.7 (CH_3), 19.5 (CH_2), 13.9 (CH_3); HRMS (ESI) Exact mass calculated for $[C_{21}H_{27}NNaO_2]^+$ $[M+Na]^+$: 348.1934, found 348.1929.

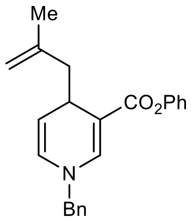

Phenyl 1-benzyl-4-(2-methylallyl)-1,4-dihydropyridine-3-carboxylate (3ga). The title compound was prepared according to General Procedure B, using azinium salt **1g** (185 mg, 0.50 mmol) and allyl pinacolboronate **2a** (137 mg, 0.75 mmol), and purified by column chromatography (1 to 3% EtOAc/*n*-pentane) to give a brown oil (144 mg, 83%). R_f = 0.65 (20% EtOAc/petroleum ether); IR 2929, 1699 (C=O), 1582, 1415, 1195, 1148, 1026, 907, 726, 690 cm^{-1}; ^{1}H NMR (400 MHz, $CDCl_3$) δ 7.48 (1H, d, *J* = 1.5 Hz, NC**H**=C), 7.42-7.30 (5H, m, Ar**H**), 7.27-7.24 (2H, m, Ar**H**), 7.21-7.16 (1H, m, Ar**H**), 7.12-7.09 (2H, m, Ar**H**), 5.86 (1H, dd, *J* = 7.9, 1.5 Hz, NC**H**=CH), 4.96 (1H, dd, *J* = 7.9, 5.0 Hz, NCH=C**H**), 4.81-4.78 (1H, m, $C\mathbf{H}_aH_b$=C), 4.69-4.67 (1H, m, $CH_a\mathbf{H}_b$=C), 4.44 (2H, s, NC**H**$_2$), 3.65 (1H, dt, *J* = 9.0, 4.5 Hz, $CH_2C\mathbf{H}$), 2.38 (1H, dd, *J* = 12.8, 4.1 Hz, $C\mathbf{H}_aH_bCH$), 2.19 (1H, dd, *J* = 12.8, 9.0 Hz, $CH_a\mathbf{H}_bCH$), 1.77 (3H, s, =CC**H**$_3$); ^{13}C NMR (101 MHz, $CDCl_3$) δ 166.8 (C), 151.5 (C), 142.9 (CH), 142.7 (C), 137.1 (C), 129.4 (2 × CH), 129.1 (2 × CH), 128.1 (CH), 127.4 (CH), 127.2 (2 × CH), 125.2 (CH), 122.1 (2 × CH), 112.6 (CH_2), 109.1 (CH), 100.9 (C), 57.9 (CH_2), 47.9 (CH_2), 30.6 (CH), 22.7 (CH_3); HRMS (ESI) Exact mass calculated for $[C_{23}H_{23}NNaO_2]^+$ $[M+Na]^+$: 368.1621, found 368.1620.

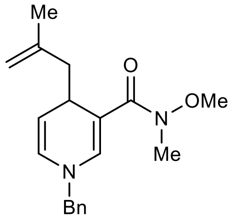

1-Benzyl-*N*-methoxy-*N*-methyl-4-(2-methylallyl)-1,4-dihydropyridine-3-carboxamide (3ha). The title compound was prepared according to General Procedure B, using azinium salt **1h** (169 mg, 0.50 mmol) and allyl pinacolboronate **2a** (137 mg, 0.75 mmol), and purified by column chromatography (15% EtOAc/*n*-pentane) to give a brown oil (49.3 mg, 32%). R_f = 0.32 (40% EtOAc/petroleum ether); IR 2929, 1672 (C=O), 1591, 1397, 1349, 1170, 1134, 885, 728, 700 cm^{-1}; ^{1}H NMR (400 MHz, $CDCl_3$) δ 7.37-7.32 (2H, m, Ar**H**), 7.30-7.27 (1H, m, Ar**H**), 7.24-7.20 (2H, m, Ar**H**), 7.08 (1H, d, *J* = 1.6 Hz, NC**H**=C), 5.84 (1H, dd, *J* = 7.8, 1.6 Hz, NC**H**=CH), 4.79 (1H, dd, *J* = 7.8, 4.9 Hz, NCH=C**H**), 4.76-4.74 (1H, m, $C\mathbf{H}_aH_b$=C), 4.66-4.63 (1H, m, $CH_a\mathbf{H}_b$=C), 4.34 (2H, s, NC**H**$_2$), 3.75 (1H, dt, *J* = 9.3, 4.7 Hz, $CH_2C\mathbf{H}$), 3.57 (3H, s, C**H**$_3$), 3.20 (3H, s, C**H**$_3$), 2.19 (1H, dd, *J* = 13.0, 4.5 Hz, $C\mathbf{H}_aH_bC$), 2.07 (1H, ddd, *J* = 13.0, 9.2, 0.8 Hz, $CH_a\mathbf{H}_bC$), 1.74 (3H, s,

=CC**H**$_3$); ^{13}C NMR (101 MHz, CDCl$_3$) δ 170.7 (C), 142.8 (C), 139.0 (CH), 137.9 (C), 128.9 (2 × CH), 127.8 (2 × CH), 127.2 (2 × CH), 112.3 (CH$_2$), 106.9 (CH), 104.8 (C), 60.4 (CH$_3$), 57.6 (CH$_2$), 48.3 (CH$_2$), 34.6 (CH$_3$), 31.2 (CH), 22.5 (CH$_3$); HRMS (ESI) Exact mass calculated for [$C_{19}H_{24}N_2NaO_2$]$^+$ [M+Na]$^+$: 335.1730, found 335.1730.

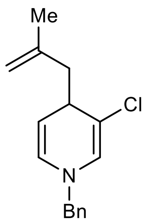

1-Benzyl-3-chloro-4-(2-methylallyl)-1,4-dihydropyridine (3ia). The title compound was prepared according to General Procedure B, using azinium salt **1i** (142 mg, 0.50 mmol) and allyl pinacolboronate **2a** (137 mg, 0.75 mmol), and purified by passing through a plug of neutralised silica (8 cm in height and 2 cm wide) using 89:10:1 *n*-pentane:EtOAc:Et$_3$N (60 mL) as the eluent to give a brown oil (122 mg, 94%). R$_f$ = 0.50 (10% EtOAc/petroleum ether); IR 2928, 1674, 1615, 1392, 1214, 1168, 1024, 887, 729, 697 cm^{-1}; ^{1}H NMR (400 MHz, CDCl$_3$) δ 7.38-7.32 (2H, m, Ar**H**), 7.31-7.27 (1H, m, Ar**H**), 7.24-7.20 (2H, m, Ar**H**), 6.08 (1H, d, *J* = 1.6 Hz, NC**H**=C), 5.82 (1H, dt, *J* = 7.9, 1.2 Hz, NC**H**=CH), 4.84-4.81 (1H, m, C**H**$_a$H$_b$=C), 4.71-4.69 (1H, m, CH$_a$**H**$_b$=C), 4.47 (1H, dd, *J* = 7.8, 3.9 Hz, NCH=C**H**), 4.20 (2H, s, NC**H**$_2$), 3.44 (1H, dt, *J* = 8.5, 3.8 Hz, CH$_2$C**H**), 2.51 (1H, dd, *J* = 13.4, 3.7 Hz, C**H**$_a$H$_b$CH), 2.18 (1H, dd, *J* = 13.4, 9.0 Hz, CH$_a$**H**$_b$CH), 1.74 (3H, s, =CC**H**$_3$); ^{13}C NMR (101 MHz, CDCl$_3$) δ 142.6 (C), 138.2 (C), 129.1 (CH), 128.85 (CH), 128.83 (2 × CH), 127.7 (CH), 127.3 (2 × CH), 112.7 (CH$_2$), 111.2 (C), 101.0 (CH), 56.9 (CH$_2$), 45.3 (CH$_2$), 38.7 (CH), 22.9 (CH$_3$); HRMS (ESI) Exact mass calculated for [$C_{16}H_{19}ClN$]$^+$ [M+H]$^+$: 262.1176, found 262.1173.

The regioisomeric configuration of **3ia** was determined by HMBC NMR spectroscopy (see page 68).

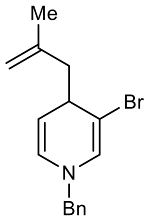

1-Benzyl-3-bromo-4-(2-methylallyl)-1,4-dihydropyridine (3ja). The title compound was prepared according to General Procedure B, using azinium salt **1j** (165 mg, 0.50 mmol) and allyl pinacolboronate **2a** (137 mg, 0.75 mmol), and purified by passing through a plug of neutralised silica (8 cm in height and 2 cm wide) using 89:10:1 *n*-pentane:EtOAc:Et$_3$N (60 mL) as the eluent to give a brown oil (124 mg, 82%). R$_f$ = 0.54 (10% EtOAc/petroleum ether); IR 2928, 1671, 1610, 1389, 1213, 1166, 1010, 887, 728, 696 cm^{-1}; ^{1}H NMR (400 MHz, CDCl$_3$) δ 7.38-7.33 (2H, m, Ar**H**), 7.31-7.27 (1H, m, Ar**H**), 7.24-7.20 (2H, m, Ar**H**), 6.22 (1H, d, *J* = 1.6 Hz, NC**H**=C), 5.84 (1H, dt, *J* = 7.9, 1.1 Hz, NC**H**=CH), 4.84-4.81 (1H, m, C**H**$_a$H$_b$=C), 4.72-4.69 (1H, m, CH$_a$**H**$_b$=C), 4.43 (1H, dd, *J* = 7.9, 4.1 Hz, NCH=C**H**), 4.20 (2H, s, NC**H**$_2$), 3.48 (1H, dt, *J* = 9.5, 3.8 Hz, CH$_2$C**H**), 2.52 (1H, dd, *J* = 13.2, 3.4 Hz, C**H**$_a$H$_b$CH), 2.19 (1H, dd, *J* = 13.2, 9.2 Hz, CH$_a$**H**$_b$CH), 1.73 (3H, s, =CC**H**$_3$); ^{13}C NMR (101 MHz, CDCl$_3$) δ 142.5 (C), 138.2 (C), 131.7 (CH), 129.0 (CH), 128.8 (2 × CH), 127.7 (CH), 127.3 (2 × CH), 112.8 (CH$_2$), 101.6

(C), 100.9 (CH), 56.9 (CH_2), 46.0 (CH_2), 39.8 (CH), 22.9 (CH_3); HRMS (ESI) Exact mass calculated for $[C_{16}H_{19}BrN]^+$ $[M+H]^+$: 306.0680, found 306.0675.

The regioisomeric configuration of **3ja** was determined by HMBC NMR spectroscopy (see page 70).

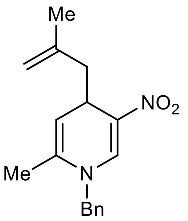

1-Benzyl-2-methyl-4-(2-methylallyl)-5-nitro-1,4-dihydropyridine (3ma). The title compound was prepared according to General Procedure B, using azinium salt **1m** (155 mg, 0.50 mmol) and allyl pinacolboronate **2a** (137 mg, 0.75 mmol), and purified by column chromatography (5% EtOAc/*n*-pentane) to give a red oil (40.3 mg, 28%). R_f = 0.34 (20% EtOAc/petroleum ether); IR 2914, 1676, 1600 (NO_2), 1386 (NO_2), 1271, 1210, 1075, 891, 726, 695 cm^{-1}; ^{1}H NMR (400 MHz, $CDCl_3$) δ 7.96 (1H, s, NC**H**=C), 7.42-7.36 (2H, m, Ar**H**), 7.35-7.30 (1H, m, Ar**H**), 7.21-7.17 (2H, m, Ar**H**), 4.98 (1H, dd, *J* = 5.3, 1.3 Hz, NC=C**H**), 4.83-4.81 (1H, m, C$\mathbf{H}_a$$H_b$=C), 4.69 (1H, d, *J* = 16.5 Hz, NC$\mathbf{H}_a$$H_b$), 4.66-4.64 (1H, m, C$H_a$$\mathbf{H}_b$=C), 4.57 (1H, d, *J* = 16.5 Hz, NC$H_a$$\mathbf{H}_b$), 3.99-3.93 (1H, m, CH_2C**H**), 2.44 (1H, dd, *J* = 13.2, 3.5 Hz, C$\mathbf{H}_a$$H_b$CH), 2.21 (1H, dd, *J* = 13.2, 8.6 Hz, C$H_a$$\mathbf{H}_b$CH), 1.81 (3H, s, NCC$\mathbf{H}_3$), 1.77 (3H, s, =CC$\mathbf{H}_3$); ^{13}C NMR (101 MHz, $CDCl_3$) δ 142.1 (C), 142.0 (CH), 136.9 (C), 132.6 (C), 129.3 (2 × CH), 128.3 (CH), 126.3 (C), 126.1 (2 × CH), 113.3 (CH_2), 110.8 (CH), 55.3 (CH_2), 44.3 (CH_2), 33.1 (CH), 23.0 (CH_3), 18.2 (CH_3); HRMS (ESI) Exact mass calculated for $[C_{17}H_{20}N_2NaO_2]^+$ $[M+Na]^+$: 307.1417, found 307.1416.

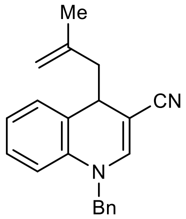

1-Benzyl-4-(2-methylallyl)-1,4-dihydroquinoline-3-carbonitrile (3na). The title compound was prepared according to General Procedure B, using azinium salt **1n** (163 mg, 0.50 mmol) and allyl pinacolboronate **2a** (137 mg, 0.75 mmol), and purified by column chromatography (2 to 20% EtOAc/*n*-pentane) to give a brown solid (126 mg, 84%). R_f = 0.49 (20% EtOAc/petroleum ether); m.p. 82-83 °C (Et_2O); IR 2977, 2183 (C≡N), 1601, 1471, 1394, 1218, 1161, 1106, 791, 751 cm^{-1}; ^{1}H NMR (500 MHz, $CDCl_3$) δ 7.38-7.33 (2H, m, Ar**H**), 7.32-7.28 (1H, m, Ar**H**), 7.24-7.21 (2H, m, Ar**H**), 7.09-7.04 (2H, m, Ar**H**), 6.99 (1H, td, *J* = 7.4, 1.2 Hz, Ar**H**), 6.95 (1H, s, NC**H**=C), 6.72 (1H, dd, *J* = 8.1, 1.1 Hz, Ar**H**), 4.88-4.83 (2H, m, NC$\mathbf{H}_a$$H_b$ and C$\mathbf{H}_a$$H_b$=C), 4.72 (1H, d, *J* = 16.6 Hz, NC$H_a$$\mathbf{H}_b$), 4.65-4.63 (1H, m, C$H_a$$\mathbf{H}_b$=C), 3.90 (1H, t, *J* = 6.2 Hz, CH_2C**H**), 2.38 (2H, qdd, *J* = 13.2, 6.3, 0.9 Hz, C$\mathbf{H}_2$CH), 1.69 (3H, s, =CC$\mathbf{H}_3$); ^{13}C NMR (126 MHz, $CDCl_3$) δ 144.3 (CH), 141.4 (C), 137.1 (C), 136.1 (C), 129.5 (CH), 129.1 (2 × CH), 128.0 (CH), 127.6 (CH), 126.4 (2 × CH), 124.6 (C), 123.7 (CH), 121.1 (C), 115.2 (CH_2), 113.6 (CH), 82.5 (C), 54.9 (CH_2), 47.9 (CH_2), 37.2 (CH), 23.0 (CH_3); HRMS (ESI) Exact mass calculated for $[C_{21}H_{21}N_2]^+$ $[M+H]^+$: 301.1699, found 301.1696.

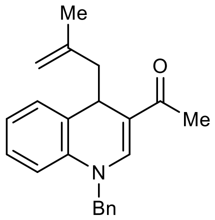

1-(1-Benzyl-4-(2-methylallyl)-1,4-dihydroquinolin-3-yl)ethan-1-one (3oa). The title compound was prepared according to General Procedure B, using azinium salt **1o** (171 mg, 0.50 mmol) and allyl pinacolboronate **2a** (137 mg, 0.75 mmol), and purified by column chromatography (5 to 15% EtOAc/*n*-pentane) to give a brown solid (149 mg, 94%). R_f = 0.22 (20% EtOAc/petroleum ether); m.p. 83-84 °C (Et_2O); IR 2956, 1643 (C=O), 1594, 1567, 1358, 1222, 968, 887, 752, 693 cm^{-1}; ^1H NMR (500 MHz, $CDCl_3$) δ 7.39 (1H, s, NC**H**=C), 7.38-7.34 (2H, m, Ar**H**), 7.32-7.25 (3H, m, Ar**H**), 7.13 (1H, dd, *J* = 7.5, 1.6 Hz, Ar**H**), 7.04 (1H, td, *J* = 7.4, 1.2 Hz, Ar**H**), 6.97 (1H, td, *J* = 7.4, 1.1 Hz, Ar**H**), 6.75 (1H, dd, *J* = 8.2, 1.1 Hz, Ar**H**), 4.99 (1H, d, *J* = 16.6 Hz, NC$\mathbf{H}_a H_b$), 4.84 (1H, d, *J* = 16.6 Hz, NC$H_a\mathbf{H}_b$), 4.68-4.66 (1H, m, C$\mathbf{H}_a H_b$=C), 4.34-4.29 (2H, m, C$H_a\mathbf{H}_b$=C and CH_2C**H**), 2.26 (3H, s, O=CC$\mathbf{H}_3$), 2.18 (2H, d, *J* = 6.3 Hz, C$\mathbf{H}_2$CH), 1.69 (3H, s, =CC$\mathbf{H}_3$); ^{13}C NMR (126 MHz, $CDCl_3$) δ 194.2 (C), 144.1 (CH), 143.1 (C), 137.5 (C), 136.4 (C), 130.0 (CH), 129.1 (2 × CH), 127.9 (CH), 127.5 (C), 126.8 (CH), 126.4 (2 × CH), 123.4 (CH), 114.4 (C), 113.6 (CH_2), 113.2 (CH), 55.3 (CH_2), 47.2 (CH_2), 34.8 (CH), 24.6 (CH_3), 22.9 (CH_3); HRMS (ESI) Exact mass calculated for $[C_{22}H_{24}NO]^+$ $[M+H]^+$: 318.1852, found 318.1853.

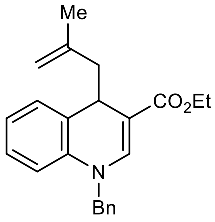

Ethyl 1-benzyl-4-(2-methylallyl)-1,4-dihydroquinoline-3-carboxylate (3pa). The title compound was prepared according to General Procedure B, using azinium salt **1p** (186 mg, 0.50 mmol) and allyl pinacolboronate **2a** (137 mg, 0.75 mmol), and purified by column chromatography (3% EtOAc/*n*-pentane) to give an off-white solid (161 mg, 93%). R_f = 0.35 (20% EtOAc/petroleum ether); m.p. 70-71 °C (Et_2O); IR 2902, 1687 (C=O), 1623, 1572, 1468, 1180, 1139, 1076, 850, 769 cm^{-1}; ^1H NMR (400 MHz, $CDCl_3$) δ 7.52 (1H, s, NC**H**=C), 7.37-7.31 (2H, m, Ar**H**), 7.29-7.23 (3H, m, Ar**H**), 7.10 (1H, dd, *J* = 7.4, 1.6 Hz, Ar**H**), 7.04-6.99 (1H, m, Ar**H**), 6.97-6.92 (1H, m, Ar**H**), 6.70 (1H, dd, *J* = 8.2, 1.1 Hz, Ar**H**), 4.92 (1H, d, *J* = 16.8 Hz, NC$\mathbf{H}_a H_b$), 4.80 (1H, d, *J* = 16.8 Hz, NC$H_a\mathbf{H}_b$), 4.71-4.69 (1H, m, C$\mathbf{H}_a H_b$=C), 4.39-4.37 (1H, m, C$H_a\mathbf{H}_b$=C), 4.25-4.18 (2H, m, OC$\mathbf{H}_2$), 4.13 (1H, dd, *J* = 7.1, 5.4 Hz, CH_2C**H**), 2.29-2.19 (2H, m, C$\mathbf{H}_2$CH), 1.71 (3H, s, =CC$\mathbf{H}_3$), 1.31 (3H, t, *J* = 7.1 Hz, $CH_2C\mathbf{H}_3$); ^{13}C NMR (101 MHz, $CDCl_3$) δ 167.7 (C), 142.8 (C), 142.6 (CH), 137.8 (C), 136.9 (C), 129.9 (CH), 129.0 (2 × CH), 127.7 (CH), 127.0 (C), 126.9 (CH), 126.4 (2 × CH), 123.0 (CH), 113.7 (CH_2), 113.2 (CH), 102.5 (C), 59.7 (CH_2), 55.1 (CH_2), 48.0 (CH_2), 35.9 (CH), 22.9 (CH_3), 14.7 (CH_3); HRMS (ESI) Exact mass calculated for $[C_{23}H_{26}NNaO_2]^+$ $[M+H]^+$: 348.1958, found 348.1955.

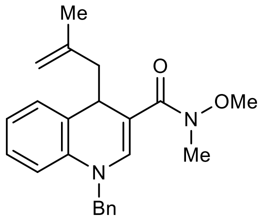

1-Benzyl-*N*-methoxy-*N*-methyl-4-(2-methylallyl)-1,4-dihydroquinoline-3-carboxamide (3qa). The title compound was prepared according to General Procedure B, using azinium salt **1q** (194 mg, 0.50 mmol) and allyl pinacolboronate **2a** (137 mg, 0.75 mmol), and purified by column chromatography (10 to 15% EtOAc/*n*-pentane) to give an off-white solid (152 mg, 84%). R_f = 0.48 (40% EtOAc/petroleum ether); m.p. 127-128 °C (Et_2O); IR 2971, 1630 (C=O), 1602, 1389, 1218, 1184, 971, 756, 729, 695 cm^{-1}; ^{1}H NMR (500 MHz, $CDCl_3$) δ 7.52 (1H, s, NC**H**=C), 7.36-7.32 (2H, m, Ar**H**), 7.29-7.24 (3H, m, Ar**H**), 7.10 (1H, dd, *J* = 7.5, 1.6 Hz, Ar**H**), 7.04 (1H, ddd, *J* = 8.1, 7.3, 1.6 Hz, Ar**H**), 6.94 (1H, td, *J* = 7.4, 1.1 Hz, Ar**H**), 6.74 (1H, dd, *J* = 8.2, 1.1 Hz, Ar**H**), 4.91 (1H, d, *J* = 16.5 Hz, NC$\mathbf{H}_a$$H_b$), 4.78 (1H, d, *J* = 16.5 Hz, NC$H_a$$\mathbf{H}_b$), 4.70-4.68 (1H, m, C$\mathbf{H}_a$$H_b$=C), 4.41-4.39 (1H, m, C$H_a$$\mathbf{H}_b$=C), 4.29 (1H, dd, *J* = 7.6, 5.8 Hz, CH_2C**H**), 3.58 (3H, s, C$\mathbf{H}_3$), 3.25 (3H, s, C$\mathbf{H}_3$), 2.31 (1H, ddd, *J* = 12.8, 5.8, 1.0 Hz, C$\mathbf{H}_a$$H_b$CH), 2.16 (1H, ddd, *J* = 12.8, 7.6, 0.8 Hz, C$H_a$$\mathbf{H}_b$CH), 1.74 (3H, s, =CC$\mathbf{H}_3$); ^{13}C NMR (126 MHz, $CDCl_3$) δ 169.4 (C), 143.1 (C), 140.8 (CH), 138.1 (C), 137.1 (C), 129.7 (CH), 129.0 (2 × CH), 127.7 (CH), 126.9 (C), 126.7 (CH), 126.6 (2 × CH), 122.5 (CH), 113.5 (CH_2), 112.6 (CH), 105.1 (C), 60.3 (CH_3), 54.7 (CH_2), 47.9 (CH_2), 36.9 (CH), 34.7 (CH_3), 22.7 (CH_3); HRMS (ESI) Exact mass calculated for $[C_{23}H_{27}N_2O_2]^+$ $[M+H]^+$: 363.2067, found 363.2064.

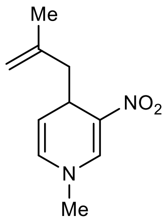

1-Methyl-4-(2-methylallyl)-3-nitro-1,4-dihydropyridine (3ra). The title compound was prepared according to a slight modification of General Procedure B (in that the reaction time was 40 h), using azinium salt **1r** (133 mg, 0.50 mmol) and allyl pinacolboronate **2a** (137 mg, 0.75 mmol), and purified by column chromatography (5% EtOAc/*n*-pentane) to give a red solid (56.3 mg, 58%). R_f = 0.29 (20% EtOAc/petroleum ether); m.p. 71-72 °C (Et_2O); IR 3070, 2918, 1668, 1579 (NO_2), 1364 (NO_2), 1248, 1192, 1076, 880, 739 cm^{-1}; ^{1}H NMR (400 MHz, $CDCl_3$) δ 7.82 (1H, t, *J* = 1.2 Hz, NC**H**=C), 5.81 (1H, dt, *J* = 7.9, 1.2 Hz, NC**H**=CH), 5.19 (1H, dd, *J* = 7.9, 5.1 Hz, NCH=C**H**), 4.81-4.79 (1H, m, C$\mathbf{H}_a$$H_b$=C), 4.66-4.63 (1H, m, C$H_a$$\mathbf{H}_b$=C), 3.97-3.91 (1H, m, CH_2C**H**), 3.16 (3H, s, NC$\mathbf{H}_3$), 2.50-2.44 (1H, m, C$\mathbf{H}_a$$H_b$CH), 2.13 (1H, dd, *J* = 13.1, 9.2 Hz, C$H_a$$\mathbf{H}_b$CH), 1.76 (3H, s, =CC$\mathbf{H}_3$); ^{13}C NMR (101 MHz, $CDCl_3$) δ 141.8 (C), 140.8 (CH), 127.1 (CH), 125.2 (C), 113.2 (CH_2), 113.0 (CH), 44.5 (CH_2), 41.7 (CH_3), 31.9 (CH), 22.6 (CH_3); HRMS (ESI) Exact mass calculated for $[C_{10}H_{14}N_2NaO_2]^+$ $[M+Na]^+$: 217.0947, found 217.0948.

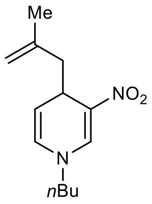

1-Butyl-4-(2-methylallyl)-3-nitro-1,4-dihydropyridine (3sa).

Using azinium salt **1sBr**: The title compound was prepared according to a slight modification of General Procedure B (in that the reaction time was 40 h), using azinium salt **1sBr** (131 mg, 0.50 mmol) and allyl pinacolboronate **2a** (137 mg, 0.75 mmol), and purified by column chromatography (4% EtOAc/*n*-pentane) to give a red oil (98.7 mg, 84%).

Using azinium salt **1sI**: The title compound was prepared according to a slight modification of General Procedure B (in that the reaction time was 40 h), using azinium salt **1sI** (154 mg, 0.50 mmol) and allyl pinacolboronate **2a** (137 mg, 0.75 mmol), and purified by column chromatography (5% EtOAc/*n*-pentane) to give an orange oil (44.1 mg, 37%).

R_f = 0.46 (20% EtOAc/petroleum ether); IR 3072, 2930, 1667, 1584 (NO_2), 1371 (NO_2), 1259, 1183, 1017, 888, 734 cm^{-1}; ^{1}H NMR (400 MHz, $CDCl_3$) δ 7.85 (1H, t, *J* = 1.2 Hz, NC**H**=C), 5.85 (1H, dt, *J* = 7.9, 1.2 Hz, NC**H**=CH), 5.19 (1H, dd, *J* = 7.9, 5.1 Hz, NCH=C**H**), 4.82-4.80 (1H, m, C$\mathbf{H}_a$$H_b$=C), 4.66-4.64 (1H, m, C$H_a$$\mathbf{H}_b$=C), 3.97 (1H, ddd, *J* = 8.7, 5.0, 3.5 Hz, CH_2C**H**), 3.30 (2H, td, *J* = 7.0, 3.4 Hz, NC$\mathbf{H}_2$), 2.45 (1H, dd, *J* = 13.1, 8.9 Hz, C$\mathbf{H}_a$$H_b$CH), 2.17 (1H, dd, *J* = 13.1, 3.4 Hz, C$H_a$$\mathbf{H}_b$CH), 1.77 (3H, s, =CC$\mathbf{H}_3$), 1.65-1.57 (2H, m, C$\mathbf{H}_2$$CH_2$$CH_3$), 1.40-1.30 (2H, m, C$\mathbf{H}_2$$CH_3$), 0.96 (3H, t, *J* = 7.3 Hz, CH_2C$\mathbf{H}_3$); ^{13}C NMR (101 MHz, $CDCl_3$) δ 141.9 (C), 140.3 (CH), 126.3 (CH), 125.0 (C), 113.3 (CH_2), 113.1 (CH), 54.9 (CH_2), 44.3 (CH_2), 32.3 (CH), 32.0 (CH_2), 22.7 (CH_3), 19.6 (CH_2), 13.7 (CH_3); HRMS (ESI) Exact mass calculated for $[C_{13}H_{20}N_2NaO_2]^+$ $[M+Na]^+$: 259.1417, found 259.1416.

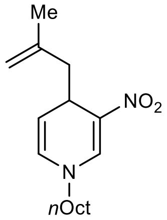

4-(2-Methylallyl)-3-nitro-1-octyl-1,4-dihydropyridine (3ta). The title compound was prepared according to a slight modification of General Procedure B (in that the reaction time was 40 h), using azinium salt **1t** (159 mg, 0.50 mmol) and allyl pinacolboronate **2a** (137 mg, 0.75 mmol), and purified by column chromatography (2 to 3% EtOAc/*n*-pentane) to give a red oil (118 mg, 81%). R_f = 0.55 (20% EtOAc/petroleum ether); IR 3073, 2925, 2855, 1667, 1585 (NO_2), 1373 (NO_2), 1259, 1189, 888, 730 cm^{-1}; ^{1}H NMR (400 MHz, $CDCl_3$) δ 7.84 (1H, t, *J* = 1.1 Hz, NC**H**=C), 5.84 (1H, dt, *J* = 8.0, 1.0 Hz, NC**H**=CH), 5.19 (1H, dd, *J* = 7.8, 5.1 Hz, NCH=C**H**), 4.82-4.80 (1H, m, C$\mathbf{H}_a$$H_b$=C), 4.66-4.64 (1H, m, C$H_a$$\mathbf{H}_b$=C), 3.97 (1H, ddd, *J* = 8.7, 5.0, 3.6 Hz, CH_2C**H**), 3.35-3.23 (2H, m, NC$\mathbf{H}_2$), 2.45 (1H, dd, *J* = 13.0, 3.4 Hz, C$\mathbf{H}_a$$H_b$CH), 2.16 (1H, dd, *J* = 13.0, 8.9 Hz, C$H_a$$\mathbf{H}_b$CH), 1.76 (3H, s, =CC$\mathbf{H}_3$), 1.65-1.58 (2H, m, N$CH_2C\mathbf{H}_2$), 1.34-1.22 (10H, m, (C$\mathbf{H}_2$)$_5$$CH_3$), 0.90-0.86 (3H, m, CH_2C$\mathbf{H}_3$); ^{13}C NMR (126 MHz, $CDCl_3$) δ 141.9 (C), 140.3 (CH), 126.3 (CH), 125.0 (C), 113.3 (CH_2), 113.1 (CH), 55.2 (CH_2), 44.3 (CH_2), 32.3 (CH), 31.8 (CH_2), 29.9 (CH_2), 29.23 (CH_2), 29.21 (CH_2), 26.3 (CH_2), 22.7

(CH_2 and CH_3), 14.2 (CH_3); HRMS (ESI) Exact mass calculated for $[C_{17}H_{29}N_2O_2]^+$ $[M+H]^+$: 293.2224, found 293.2221.

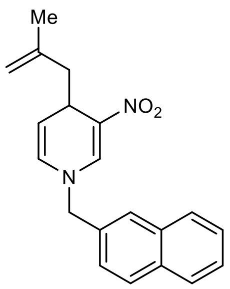

4-(2-Methylallyl)-1-(naphthalen-2-ylmethyl)-3-nitro-1,4-dihydropyridine (3ua). The title compound was prepared according to a slight modification of General Procedure B (in that the reaction time was 40 h), using azinium salt **1u** (173 mg, 0.50 mmol) and allyl pinacolboronate **2a** (137 mg, 0.75 mmol), and purified by column chromatography (5% EtOAc/*n*-pentane) to give a red oil (139 mg, 87%). R_f = 0.36 (20% EtOAc/petroleum ether); IR 3066, 2916, 1999, 1765, 1509 (NO_2), 1369 (NO_2), 1176, 1079, 889, 730 cm^{-1}; ^1H NMR (400 MHz, $CDCl_3$) δ 8.03 (1H, t, *J* = 1.1 Hz, NC**H**=C), 7.90-7.82 (3H, m, Ar**H**), 7.67 (1H, s, Ar**H**), 7.56-7.50 (2H, m, Ar**H**), 7.31 (1H, dd, *J* = 8.4, 1.8 Hz, Ar**H**), 5.91 (1H, dt, *J* = 7.9, 1.2 Hz, NC**H**=CH), 5.19 (1H, dd, *J* = 7.9, 5.1 Hz, NCH=C**H**), 4.83-4.81 (1H, m, C$\mathbf{H}_a$$H_b$=C), 4.67-4.65 (1H, m, C$H_a$$\mathbf{H}_b$=C), 4.64 (2H, s, NC$\mathbf{H}_2$), 4.02 (1H, ddd, *J* = 8.7, 5.0, 3.5 Hz, CH_2C**H**), 2.47 (1H, dd, *J* = 13.1, 3.4 Hz, C$\mathbf{H}_a$$H_b$CH), 2.23 (1H, dd, *J* = 13.1, 8.8 Hz, C$H_a$$\mathbf{H}_b$CH), 1.77 (3H, s, =CC$\mathbf{H}_3$); ^{13}C NMR (101 MHz, $CDCl_3$) δ 141.8 (C), 140.4 (CH), 133.4 (C), 133.3 (C), 132.7 (C), 129.4 (CH), 127.98 (CH), 127.96 (CH), 127.0 (CH), 126.8 (CH), 126.7 (CH), 126.4 (CH), 125.9 (C), 124.7 (CH), 113.5 (CH_2), 113.2 (CH), 58.7 (CH_2), 44.4 (CH_2), 32.4 (CH), 22.8 (CH_3); HRMS (ESI) Exact mass calculated for $[C_{20}H_{21}N_2O_2]^+$ $[M+H]^+$: 321.1598, found 321.1595.

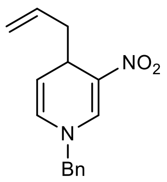

4-Allyl-1-benzyl-3-nitro-1,4-dihydropyridine (3ab). The title compound was prepared according to a slight modification of General Procedure B (in that the reaction time was 1.5 h), using azinium salt **1a** (148 mg, 0.50 mmol) and allyl pinacolboronate **2b** (141 µL, 0.75 mmol), and purified by column chromatography (5% EtOAc/*n*-pentane) to give a red oil (115 mg, 90%). R_f = 0.46 (20% EtOAc/petroleum ether); IR 2918, 1672, 1593 (NO_2), 1475, 1361 (NO_2), 1271, 1211, 1171, 1028, 698 cm^{-1}; ^1H NMR (400 MHz, $CDCl_3$) δ 7.96 (1H, s, NC**H**=C), 7.43-7.33 (3H, m, Ar**H**), 7.24-7.20 (2H, m, Ar**H**), 5.89 (1H, dd, *J* = 8.0, 1.3 Hz, NC**H**=CH), 5.79-7.68 (1H, m, CH_2=C**H**), 5.13 (1H, dd, *J* = 7.9, 5.1 Hz, NCH=C**H**), 5.07-4.99 (2H, m, C$\mathbf{H}_2$=CH), 4.47 (2H, s, NC$\mathbf{H}_2$), 3.99-3.95 (1H, m, CH_2C**H**), 2.49-2.42 (1H, m, C$\mathbf{H}_a$$H_b$CH), 2.30-2.23 (1H, m, C$H_a$$\mathbf{H}_b$CH); ^{13}C NMR (101 MHz, $CDCl_3$) δ 140.6 (CH), 135.3 (C), 134.4 (CH), 129.3 (2 × CH), 128.7 (CH), 127.5 (2 × CH), 127.1 (CH), 125.0 (C), 118.2 (CH_2), 112.8 (CH), 58.5 (CH_2), 39.4 (CH_2), 33.7 (CH); HRMS (ESI) Exact mass calculated for $[C_{15}H_{16}N_2NaO_2]^+$ $[M+Na]^+$: 279.1104, found 279.1106.

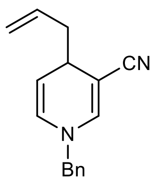

4-Allyl-1-benzyl-1,4-dihydropyridine-3-carbonitrile (3bb). The title compound was prepared according to General Procedure B, using azinium salt **1b** (138 mg, 0.50 mmol) and allyl pinacolboronate **2b** (141 µL, 0.75 mmol), and purified by column chromatography (3% EtOAc/*n*-pentane) to give a green oil (77.4 mg, 65%). R_f = 0.38 (20% EtOAc/petroleum ether); IR 2922, 2190 (C≡N), 1672, 1592, 1409, 1179, 1077, 913, 732, 699 cm^{-1}; ^1H NMR (400 MHz, $CDCl_3$) δ 7.40-7.30 (3H, m, Ar**H**), 7.22-7.18 (2H, m, Ar**H**), 6.62 (1H, d, *J* = 1.6 Hz, NC**H**=C), 5.87-5.76 (2H, m, CH_2=C**H** and NC**H**=CH), 5.15-5.08 (2H, m, C**H**$_2$=CH), 4.67 (1H, dd, *J* = 8.1, 4.1 Hz, NCH=C**H**), 4.28 (2H, s, NC**H**$_2$), 3.36-3.31 (1H, m, CH_2C**H**), 2.30 (2H, t, *J* = 6.1 Hz, C**H**$_2$CH); ^{13}C NMR (101 MHz, $CDCl_3$) δ 143.2 (CH), 136.5 (C), 134.4 (CH), 129.0 (2 × CH), 128.3 (CH), 128.2 (CH), 127.2 (2 × CH), 121.2 (C), 117.9 (CH_2), 105.7 (CH), 82.4 (C), 57.5 (CH_2), 42.4 (CH_2), 33.3 (CH); HRMS (ESI) Exact mass calculated for $[C_{16}H_{16}N_2Na]^+$ $[M+Na]^+$: 259.1206, found 259.1201.

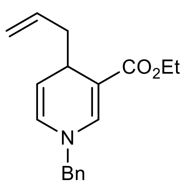

Ethyl 4-allyl-1-benzyl-1,4-dihydropyridine-3-carboxylate (3eb). The title compound was prepared according to General Procedure B, using azinium salt **1e** (161 mg, 0.50 mmol) and allyl pinacolboronate **2b** (141 µL, 0.75 mmol), and purified by column chromatography (5% EtOAc/*n*-pentane) to give a green oil (57.9 mg, 41%). R_f = 0.62 (20% EtOAc/petroleum ether); IR 2977, 1680 (C=O), 1587, 1397, 1276, 1203, 1160, 1027, 909, 730 cm^{-1}; ^1H NMR (400 MHz, $CDCl_3$) δ 7.39-7.27 (3H, m, Ar**H**), 7.25 (1H, d, *J* = 1.7 Hz, NC**H**=C), 7.24-7.20 (2H, m, Ar**H**), 5.86-5.75 (2H, m, CH_2=C**H** and NC**H**=CH), 5.03-4.96 (2H, m, C**H**$_2$=CH), 4.80 (1H, dd, *J* = 7.9, 5.0 Hz, NCH=C**H**), 4.35 (2H, s, NC**H**$_2$), 4.22-4.09 (2H, m, OC**H**$_2$), 3.54-3.48 (1H, m, CH_2C**H**), 2.30-2.14 (2H, m, C**H**$_2$CH), 1.26 (3H, t, *J* = 7.1 Hz, C**H**$_3$); ^{13}C NMR (101 MHz, $CDCl_3$) δ 168.3 (C), 141.7 (CH), 137.4 (C), 136.0 (CH), 128.9 (2 × CH), 128.2 (CH), 127.9 (CH), 127.1 (2 × CH), 116.6 (CH_2), 107.9 (CH), 101.2 (C), 59.5 (CH_2), 57.7 (CH_2), 42.7 (CH_2), 32.0 (CH), 14.6 (CH_3); HRMS (ESI) Exact mass calculated for $[C_{18}H_{21}NNaO_2]^+$ $[M+Na]^+$: 306.1464, found 306.1461.

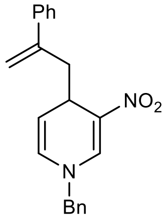

1-Benzyl-3-nitro-4-(2-phenylallyl)-1,4-dihydropyridine (3ac). The title compound was prepared according to General Procedure B, using azinium salt **1a** (148 mg, 0.50 mmol) and allyl pinacolboronate **2c** (183 mg, 0.75 mmol), and purified by column chromatography (5% EtOAc/*n*-pentane) to give a red oil (137 mg, 82%). R_f = 0.32 (20% EtOAc/petroleum ether); IR 2926, 1666, 1586 (NO_2), 1476, 1302 (NO_2), 1262, 1173, 905, 728, 696 cm^{-1}; ^1H NMR (400 MHz, $CDCl_3$) δ 7.84 (1H, t, *J* = 1.1 Hz, NC**H**=C), 7.49-7.46 (2H, m, Ar**H**), 7.42-7.35 (3H, m, Ar**H**), 7.35-7.26 (3H, m, Ar**H**), 7.21-7.18 (2H, m, Ar**H**), 5.77 (1H,

dt, *J* = 7.9, 1.2 Hz, NC**H**=CH), 5.38 (1H, d, *J* = 1.6 Hz, C**H**$_a$H$_b$=C), 5.05 (1H, dd, *J* = 7.9, 5.0 Hz, NCH=C**H**), 5.01 (1H, d, *J* = 1.6 Hz, CH$_a$**H**$_b$=C), 4.42 (2H, s, NC**H**$_2$), 3.97 (1H, ddd, *J* = 8.6, 4.9, 3.4 Hz, CH$_2$C**H**), 3.19-3.14 (1H, m, C**H**$_a$H$_b$CH), 2.61 (1H, dd, *J* = 13.5, 9.0 Hz, CH$_a$**H**$_b$CH); ^{13}C NMR (101 MHz, CDCl$_3$) δ 144.4 (C), 140.4 (C), 140.3 (CH), 135.2 (C), 129.3 (2 × CH), 128.8 (CH), 128.4 (2 × CH), 127.7 (CH), 127.5 (2 × CH), 126.5 (2 × CH), 126.3 (CH), 125.6 (C), 115.6 (CH$_2$), 112.8 (CH), 58.4 (CH$_2$), 41.4 (CH$_2$), 32.7 (CH); HRMS (ESI) Exact mass calculated for $[C_{21}H_{20}N_2NaO_2]^+$ $[M+Na]^+$: 355.1417, found 355.1417.

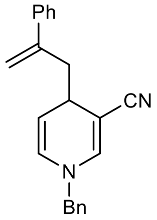

1-Benzyl-4-(2-phenylallyl)-1,4-dihydropyridine-3-carbonitrile (3bc). The title compound was prepared according to General Procedure B, using azinium salt **1b** (138 mg, 0.50 mmol) and allyl pinacolboronate **2c** (183 mg, 0.75 mmol), and purified by column chromatography (2 to 5% EtOAc/*n*-pentane) to give a yellow oil (136 mg, 87%). R$_f$ = 0.56 (20% EtOAc/petroleum ether); IR 2928, 2190 (C≡N), 1672, 1591, 1409, 1179, 1119, 1028, 902, 700 cm^{-1}; ^{1}H NMR (400 MHz, CDCl$_3$) δ 7.43-7.24 (8H, m, Ar**H**), 7.19-7.15 (2H, m, Ar**H**), 6.59 (1H, d, *J* = 1.6 Hz, NC**H**=C), 5.71 (1H, dt, *J* = 8.1, 1.3 Hz, NC**H**=CH), 5.38-5.36 (1H, m, C**H**$_a$H$_b$=C), 5.11-5.09 (1H, m, CH$_a$**H**$_b$=C), 4.63 (1H, dd, *J* = 8.1, 4.1 Hz, NCH=C**H**), 4.27 (2H, s, NC**H**$_2$), 3.32-3.26 (1H, m, CH$_2$C**H**), 3.01 (1H, ddd, *J* = 13.8, 4.1, 1.3 Hz, C**H**$_a$H$_b$CH), 2.62 (1H, dd, *J* = 13.8, 9.2 Hz, CH$_a$**H**$_b$CH); ^{13}C NMR (101 MHz, CDCl$_3$) δ 144.3 (C), 142.9 (CH), 140.7 (C), 136.5 (C), 129.1 (2 × CH), 128.5 (2 × CH), 128.3 (CH), 127.7 (CH), 127.6 (CH), 127.3 (2 × CH), 126.5 (2 × CH), 121.3 (C), 115.7 (CH$_2$), 105.7 (CH), 82.9 (C), 57.5 (CH$_2$), 45.1 (CH$_2$), 32.0 (CH); HRMS (ESI) Exact mass calculated for $[C_{22}H_{20}N_2Na]^+$ $[M+Na]^+$: 335.1519, found 335.1517.

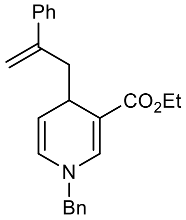

Ethyl 1-benzyl-4-(2-phenylallyl)-1,4-dihydropyridine-3-carboxylate (3ec). The title compound was prepared according to General Procedure B, using azinium salt **1e** (161 mg, 0.50 mmol) and allyl pinacolboronate **2c** (183 mg, 0.75 mmol), and purified by column chromatography (1 to 3% EtOAc/*n*-pentane) to give a yellow oil (134 mg, 75%). R$_f$ = 0.65 (20% EtOAc/petroleum ether); IR 2979, 1678 (C=O), 1585, 1398, 1204, 1160, 1073, 907, 727, 695 cm^{-1}; ^{1}H NMR (500 MHz, CDCl$_3$) δ 7.55-7.52 (2H, m, Ar**H**), 7.39-7.28 (5H, m, Ar**H**), 7.27-7.25 (1H, m, Ar**H**), 7.24-7.22 (2H, m, Ar**H**), 7.21 (1H, s, NC**H**=C), 5.77 (1H, dd, *J* = 7.9, 1.6 Hz, NC**H**=CH), 5.38-5.37 (1H, m, C**H**$_a$H$_b$=C), 5.01-5.00 (1H, m, CH$_a$**H**$_b$=C), 4.75 (1H, dd, *J* = 7.9, 5.0 Hz, NCH=C**H**), 4.37 (2H, s, NC**H**$_2$), 4.23-4.14 (2H, m, OC**H**$_2$), 3.52 (1H, ddd, *J* = 10.2, 5.0, 3.6 Hz, CH$_2$C**H**), 3.05 (1H, dd, *J* = 13.3, 3.7 Hz, C**H**$_a$H$_b$CH), 2.43 (1H, dd, *J* = 13.3, 10.0 Hz, CH$_a$**H**$_b$CH), 1.30 (3H, t, *J* = 7.1 Hz, CH$_2$C**H**$_3$); ^{13}C NMR (126 MHz, CDCl$_3$) δ 168.5 (C), 144.7 (C), 141.5 (CH), 140.8 (C), 137.4 (C), 129.0 (2 × CH), 128.3 (2 × CH),

127.9 (CH), 127.5 (CH), 127.4 (CH), 127.1 (2 × CH), 126.5 (2 × CH), 114.5 (CH_2), 107.9 (CH), 101.9 (C), 59.6 (CH_2), 57.7 (CH_2), 44.9 (CH_2), 30.8 (CH), 14.7 (CH_3); HRMS (ESI) Exact mass calculated for $[C_{24}H_{25}NNaO_2]^+$ $[M+Na]^+$: 382.1777, found 382.1776.

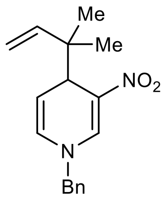

1-Benzyl-4-(2-methylbut-3-en-2-yl)-3-nitro-1,4-dihydropyridine (3ad). The title compound was prepared according to General Procedure B, using azinium salt **1a** (148 mg, 0.50 mmol) and allyl pinacolboronate **2d** (147 mg, 0.75 mmol), and purified by column chromatography (5% EtOAc/*n*-pentane) to give a red oil (77.3 mg, 54%). R_f = 0.45 (20% EtOAc/petroleum ether); IR 2965, 1660, 1581 (NO_2), 1350 (NO_2), 1265, 1176, 1073, 909, 732, 699 cm^{-1}; ^{1}H NMR (400 MHz, $CDCl_3$) δ 8.08 (1H, t, *J* = 1.5 Hz, NC**H**=C), 7.42-7.35 (3H, m, Ar**H**), 7.24-7.21 (2H, m, Ar**H**), 6.07 (1H, dd, *J* = 7.6, 1.4 Hz, NC**H**=CH), 5.77 (1H, dd, *J* = 17.4, 10.8 Hz, CH_2=C**H**), 5.15 (1H, dd, *J* = 7.6, 6.3 Hz, NCH=C**H**), 4.89-4.83 (2H, m, C**H**$_2$=CH), 4.50 (2H, d, *J* = 1.5 Hz, NC**H**$_2$), 3.91 (1H, dd, *J* = 6.3, 1.6 Hz, CC**H**C), 0.91 (3H, s, C**H**$_3$), 0.89 (3H, s, C**H**$_3$); ^{13}C NMR (101 MHz, $CDCl_3$) δ 145.1 (CH), 140.6 (CH), 135.3 (C), 129.3 (2 × CH), 128.8 (CH), 128.4 (CH), 127.8 (2 × CH), 123.2 (C), 112.3 (CH_2), 109.4 (CH), 58.8 (CH_2), 45.1 (C), 41.9 (CH), 22.9 (2 × CH_3); HRMS (ESI) Exact mass calculated for $[C_{17}H_{20}N_2NaO_2]^+$ $[M+Na]^+$: 307.1417, found 307.1414.

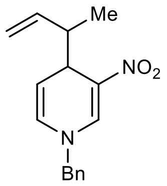

1-Benzyl-4-(but-3-en-2-yl)-3-nitro-1,4-dihydropyridine (3ae). The title compound was prepared according to General Procedure B, using azinium salt **1a** (148 mg, 0.50 mmol) and allyl pinacolboronate *rac*-**2e** (137 mg, 0.75 mmol), and purified by column chromatography (5% EtOAc/*n*-pentane) to give a 2:1 mixture of inseparable diastereomers as a red solid (76.5 mg, 57%). R_f = 0.44 (20% EtOAc/petroleum ether); m.p. 79-80 °C (Et_2O); IR 3069, 2963, 1666, 1576 (NO_2), 1314 (NO_2), 1263, 1172, 913, 737, 696 cm^{-1}; HRMS (ESI) Exact mass calculated for $[C_{16}H_{18}N_2NaO_2]^+$ $[M+Na]^+$: 293.1260, found 293.1266.

NMR data of major diastereomer: ^{1}H NMR (400 MHz, $CDCl_3$) δ 7.99 (1H, t, *J* = 1.3 Hz, NC**H**=C), 7.42-7.32 (3H, m, Ar**H**), 7.24-7.20 (2H, m, Ar**H**), 5.96-5.93 (1H, m, NC**H**=CH), 5.64 (1H, ddd, *J* = 17.0, 10.4, 8.3 Hz, CH_2=C**H**), 5.15 (1H, dd, *J* = 7.9, 5.4 Hz, NCH=C**H**), 4.97-4.90 (2H, m, C**H**$_2$=CH), 4.46 (2H, s, NC**H**$_2$), 3.89-3.86 (1H, m, CHC**H**C), 2.73-2.64 (1H, m, C**H**CH_3), 1.01 (3H, d, *J* = 7.1 Hz, C**H**$_3$); ^{13}C NMR (101 MHz, $CDCl_3$) δ 140.75 (CH), 140.5 (CH), 135.30 (C), 129.2 (2 × CH), 128.69 (CH), 128.2 (CH), 127.6 (2 × CH), 124.9 (C), 116.1 (CH_2), 109.1 (CH), 58.58 (CH_2), 40.5 (CH), 39.4 (CH_2), 15.9 (CH_3).

Characteristic NMR data of minor diastereomer: ^{1}H NMR (400 MHz, $CDCl_3$) δ 8.04 (1H, t, *J* = 1.2 Hz, NC**H**=C), 5.99-5.96 (1H, m, NC**H**=CH), 5.81 (1H, ddd, *J* = 16.9, 10.6, 6.2 Hz, CH_2=C**H**), 5.07-

5.01 (3H, m, NCH=C**H** and C**H**$_2$=CH), 4.48 (2H, s, NC**H**$_2$), 4.01-3.98 (1H, m, CHC**H**C), 2.81-2.74 (1H, m, C**H**CH$_3$), 0.93 (3H, d, *J* = 7.1 Hz, C**H**$_3$); ^{13}C NMR (101 MHz, CDCl$_3$) δ 140.83 (CH), 140.4 (CH), 135.27 (C), 129.3 (2 × CH), 128.74 (CH), 127.6 (2 × CH), 124.4 (C), 114.1 (CH$_2$), 109.8 (CH), 58.55 (CH$_2$), 38.94 (CH), 38.86 (CH$_2$), 13.4 (CH$_3$).

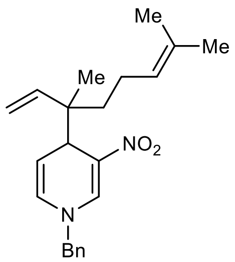

1-Benzyl-4-(3,7-dimethylocta-1,6-dien-3-yl)-3-nitro-1,4-dihydropyridine (3af). The title compound was prepared according to General Procedure B, using azinium salt **1a** (148 mg, 0.50 mmol) and allyl pinacolboronate *rac*-**2f** (198 mg, 0.75 mmol), and purified by column chromatography (5% EtOAc/*n*-pentane) to give a 2.1:1 mixture of inseparable diastereomers as a red oil (44.0 mg, 25%). R_f = 0.52 (20% EtOAc/petroleum ether); IR 2922, 1661, 1581 (NO$_2$), 1375 (NO$_2$), 1268, 1176, 1072, 909, 730, 698 cm^{-1}; HRMS (ESI) Exact mass calculated for $[C_{22}H_{28}N_2NaO_2]^+$ [M+Na]$^+$: 375.2043, found 375.2042.

NMR data of major diastereomer: ^{1}H NMR (400 MHz, CDCl$_3$) δ 8.07 (1H, t, *J* = 1.5 Hz, NC**H**=C), 7.41-7.33 (3H, m, Ar**H**), 7.24-7.21 (2H, m, Ar**H**), 6.09 (1H, dd, *J* = 7.6, 1.4 Hz, NC**H**=CH), 5.76 (1H, dd, *J* = 17.4, 10.8 Hz, CH$_2$=C**H**), 5.14 (1H, dd, *J* = 7.6, 6.3 Hz, NCH=C**H**), 5.06-5.01 (1H, m, CH$_2$C**H**=C), 4.90 (1H, dd, *J* = 10.8, 1.5 Hz, C**H**$_a$H$_b$=CH), 4.85 (1H, dd, *J* = 17.4, 1.5 Hz, CH$_a$**H**$_b$=CH), 4.50 (2H, s, NC**H**$_2$), 3.99-3.95 (1H, m, CC**H**C), 1.85-1.77 (2H, m, CCH$_2$C**H**$_2$), 1.66 (3H, s, =C(C**H**$_3$), 1.56 (3H, s, =C(C**H**$_3$)), 1.42-1.27 (2H, m, CC**H**$_2$CH$_2$), 0.85 (3H, s, CHCC**H**$_3$); ^{13}C NMR (101 MHz, CDCl$_3$) δ 143.5 (CH), 140.5 (CH), 135.4 (C), 131.4 (C), 129.27 (2 × CH), 128.78 (CH), 128.7 (CH), 127.8 (2 × CH), 124.9 (CH), 123.1 (C), 113.6 (CH$_2$), 108.7 (CH), 58.8 (CH$_2$), 48.7 (C), 42.0 (CH), 36.5 (CH$_2$), 25.9 (CH$_3$), 22.9 (CH$_2$), 17.8 (CH$_3$), 16.9 (CH$_3$).

Characteristic NMR data of minor diastereomer: ^{1}H NMR (400 MHz, CDCl$_3$) 8.09 (1H, t, *J* = 1.5 Hz, NC**H**=C), 5.59 (1H, dd, *J* = 17.4, 10.8 Hz, CH$_2$=C**H**), 5.44-5.40 (1H, m, CH$_2$C**H**=C), 4.95 (1H, dd, *J* = 10.8, 1.5 Hz, C**H**$_a$H$_b$=CH), 4.16 (1H, d, *J* = 7.0 Hz, CC**H**C), 1.68 (3H, s, =C(C**H**$_3$); ^{13}C NMR (101 MHz, CDCl$_3$) δ 143.1 (CH), 140.9 (CH), 135.2 (C), 129.25 (2 × CH), 128.77 (CH), 128.4 (CH), 127.9 (2 × CH), 125.0 (CH), 123.5 (C), 114.3 (CH$_2$), 109.4 (CH), 58.9 (CH$_2$), 48.4 (C), 41.7 (CH), 36.4 (CH$_2$), 25.8 (CH$_3$), 23.0 (CH$_2$), 17.9 (CH$_3$), 16.4 (CH$_3$).

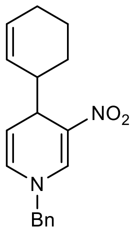

1-Benzyl-4-(cyclohex-2-en-1-yl)-3-nitro-1,4-dihydropyridine (3ag). The title compound was prepared according to General Procedure B, using azinium salt **1a** (148 mg, 0.50 mmol) and allyl pinacolboronate *rac*-**2g** (156 mg, 0.75 mmol), and purified by column chromatography (5% EtOAc/*n*-pentane) to give a 2.2:1 mixture of inseparable diastereomers as a red solid (103 mg, 70%). R_f = 0.48 (20% EtOAc/petroleum ether);

m.p. 76-77 °C (Et_2O); IR 3066, 2931, 1661, 1579 (NO_2), 1345 (NO_2), 1253, 1165, 931, 702, 586 cm^{-1}; HRMS (ESI) Exact mass calculated for $[C_{18}H_{20}N_2NaO_2]^+$ $[M+Na]^+$: 319.1417, found 319.1418.

NMR data of major diastereomer: ^{1}H NMR (400 MHz, $CDCl_3$) δ 8.06 (1H, t, *J* = 1.3 Hz, NC**H**=C), 7.42-7.32 (3H, m, Ar**H**), 7.25-7.21 (2H, m, Ar**H**), 5.96-5.93 (1H, m, NC**H**=CH), 5.70 (1H, dt, *J* = 10.1, 3.3 Hz, C**H**=CHCH₂), 5.53-5.49 (1H, m, CH=C**H**CH₂), 5.12 (1H, dd, *J* = 7.9, 5.4 Hz, NCH=C**H**), 4.49 (2H, s, NC**H₂**), 3.90-3.87 (1H, m, CHC**H**C), 2.59-2.52 (1H, m, =CHC**H**CH₂), 1.96-1.90 (2H, m, =CHC**H₂**), 1.81-1.68 (2H, m, =CHCH₂C**H₂**CH₂), 1.62-1.19 (2H, m, =CHCH₂CH₂C**H₂**); ^{13}C NMR (101 MHz, $CDCl_3$) δ 140.9 (CH), 135.34 (C), 129.3 (2 × CH), 129.2 (CH), 128.7 (CH), 128.3 (CH), 127.9 (CH), 127.6 (2 × CH), 124.4 (C), 110.5 (CH), 58.6 (CH_2), 39.8 (CH), 37.9 (CH), 25.7 (CH_2), 25.32 (CH_2), 22.4 (CH_2).

Characteristic NMR data of minor diastereomer: ^{1}H NMR (400 MHz, $CDCl_3$) δ 8.04-8.01 (1H, m, NC**H**=C), 5.82-5.76 (1H, m, C**H**=CHCH₂), 5.45 (1H, dt, *J* = 10.1, 1.9 Hz, CH=C**H**CH₂), 5.05 (1H, dd, *J* = 7.9, 5.0 Hz, NCH=C**H**), 4.47 (2H, s, NC**H₂**), 4.03-3.99 (1H, m, CHC**H**C), 2.82-2.75 (1H, m, =CHC**H**CH₂); ^{13}C NMR (101 MHz, $CDCl_3$) δ 135.30 (C), 129.7 (CH), 128.8 (CH), 127.8 (CH), 127.5 (2 × CH), 124.3 (C), 111.2 (CH), 58.5 (CH_2), 38.6 (CH), 37.8 (CH), 25.30 (CH_2), 24.6 (CH_2), 21.8 (CH_2).

2-Allyl-1-benzyl-4-methyl-3-nitro-1,2-dihydropyridine (3vba) and 2-allyl-1-benzyl-4-methyl-5-nitro-1,2-dihydropyridine (3vbb)

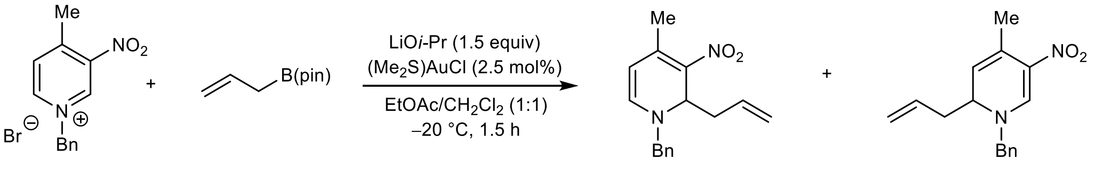

1v **2b** (1.5 equiv) **3vba** 43% **3vbb** 13%

The title compounds were prepared according to a slight modification of General Procedure B (in that the reaction was left to stir at –20 °C for 1.5 h), using azinium salt **1v** (155 mg, 0.50 mmol), and allyl pinacolboronate **2b** (141 μL, 0.75 mmol), and purified by column chromatography (5% to 15% EtOAc/*n*-pentane) to give *allylated product* **3vba** as a red oil (57.9 mg, 43%), followed by *allylated product* **3vbb** as a yellow oil (17.6 mg, 13%).

Data of major regioisomer **3vba**: R_f = 0.51 (20% EtOAc/petroleum ether); IR 2924, 1600 (NO_2), 1480, 1398, 1333 (NO_2), 1258, 1160, 994, 917, 728 cm^{-1}; ^{1}H NMR (400 MHz, $CDCl_3$) δ 7.39-7.30 (3H, m, Ar**H**), 7.23-7.19 (2H, m, Ar**H**), 6.64 (1H, d, *J* = 6.3 Hz, NC**H**=CH), 5.96-5.85 (1H, m, C**H**=CH₂), 5.24 (1H, ddd, *J* = 6.6, 5.2, 1.3 Hz, NC**H**CH₂), 5.13-5.06 (2H, m, CH=C**H₂**), 4.90 (1H, d, *J* = 6.3 Hz, NCH=C**H**), 4.57 (1H, d, *J* = 15.0 Hz, NC**H**ₐH_b), 4.50 (1H, d, *J* = 15.0 Hz, NCHₐ**H**_b), 2.52-2.45 (1H, m, CHC**H**ₐH_b), 2.44 (3H, s, C**H₃**), 2.33-2.25 (1H, m, CHCHₐ**H**_b); ^{13}C NMR (101 MHz,

$CDCl_3$) δ 148.1 (C), 145.0 (CH), 135.4 (C), 133.7 (CH), 129.3 (2 × CH), 128.7 (CH), 127.8 (2 × CH), 123.1 (C), 118.8 (CH_2), 101.0 (CH), 59.5 (CH_2), 57.4 (CH), 35.6 (CH_2), 22.6 (CH_3); HRMS (ESI) Exact mass calculated for $[C_{16}H_{18}N_2NaO_2]^+$ $[M+Na]^+$: 293.1260, found 293.1256.

Data of minor regioisomer **3vbb**: R_f = 0.45 (20% EtOAc/petroleum ether); IR 2967, 1643, 1575 (NO_2), 1390, 1346 (NO_2), 1274, 1168, 993, 816, 699 cm^{-1}; ^1H NMR (400 MHz, $CDCl_3$) δ 8.28 (1H, s, NC**H**=C), 7.44-7.36 (3H, m, Ar**H**), 7.29-7.25 (2H, m, Ar**H**), 5.86-5.76 (1H, m, CH_2=C**H**), 5.19-5.12 (2H, m, C**H**$_2$=CH), 4.78 (1H, d, *J* = 4.9 Hz, NCHC**H**), 4.57-4.49 (2H, m, NC**H**$_2$), 4.12-4.07 (1H, m, CH_2C**H**), 2.46-2.37 (1H, m, C**H**$_a$H$_b$CH), 2.29-2.23 (1H, m, CH$_a$**H**$_b$CH), 2.21 (3H, s, C**H**$_3$); ^{13}C NMR (101 MHz, $CDCl_3$) δ 148.5 (CH), 134.0 (C), 132.2 (CH), 129.4 (2 × CH), 129.0 (CH and C), 128.0 (2 × CH), 124.8 (C), 119.6 (CH_2), 112.2 (CH), 58.8 (CH_2), 57.9 (CH), 39.1 (CH_2), 21.4 (CH_3); HRMS (ESI) Exact mass calculated for $[C_{16}H_{18}N_2NaO_2]^+$ $[M+Na]^+$: 293.1260, found 293.1257.

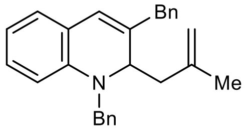

1,3-Dibenzyl-2-(2-methylallyl)-1,2-dihydroquinoline (3wa). The title compound was prepared according to General Procedure B, using azinium salt **1w** (195 mg, 0.50 mmol) and allyl pinacolboronate **2a** (137 mg, 0.75 mmol), and purified by column chromatography (0.5 to 1% EtOAc/*n*-pentane) to give a yellow oil (73.6 mg, 40%). R_f = 0.69 (10% EtOAc/petroleum ether); IR 3026, 2912, 1644, 1597, 1493, 1451, 1273, 907, 728, 696 cm^{-1}; ^1H NMR (400 MHz, $CDCl_3$) δ 7.30-7.16 (8H, m, Ar**H**), 7.13-7.08 (2H, m, Ar**H**), 7.04-6.96 (2H, m, Ar**H**), 6.66 (1H, td, *J* = 7.4, 1.0 Hz, Ar**H**), 6.55 (1H, d, *J* = 8.1 Hz, Ar**H**), 6.23 (1H, s, C**H**=CCH_2), 4.82-4.79 (1H, m, C**H**$_a$H$_b$=C), 4.76-4.73 (1H, m, CH$_a$**H**$_b$=C), 4.64 (1H, d, *J* = 15.7 Hz, NC**H**$_a$H$_b$), 4.23 (1H, d, *J* = 15.7 Hz, NCH$_a$**H**$_b$), 3.87 (1H, t, *J* = 6.5 Hz, C**H**CH_2), 3.55 (1H, d, *J* = 15.3 Hz, CC**H**$_a$H$_b$C), 3.35 (1H, dd, *J* = 15.3, 1.7 Hz, CCH$_a$**H**$_b$C), 2.34 (1H, dd, *J* = 13.0, 6.8 Hz, CHC**H**$_a$H$_b$), 2.13 (1H, dd, *J* = 13.0, 6.2 Hz, CHCH$_a$**H**$_b$), 1.69 (3H, s, =CC**H**$_3$); ^{13}C NMR (101 MHz, $CDCl_3$) δ 142.9 (C), 142.6 (C), 139.1 (C), 138.2 (C), 137.4 (C), 129.2 (2 × CH), 128.5 (4 × CH), 128.2 (CH), 127.4 (2 × CH), 127.0 (CH), 126.7 (CH), 126.5 (CH), 123.8 (C), 122.4 (CH), 117.2 (CH), 114.1 (CH_2), 112.6 (CH), 58.9 (CH), 53.7 (CH_2), 41.9 (CH_2), 40.7 (CH_2), 22.7 (CH_3); HRMS (ESI) Exact mass calculated for $[C_{27}H_{28}N]^+$ $[M+H]^+$: 366.2216, found 366.2218.

The regioisomeric configuration of **3va** was determined by HMBC NMR spectroscopy (see page 92).

1-Benzyl-4-(2-methylbut-3-en-2-yl)-3-nitro-1,4-dihydropyridine (3ad) and 1-benzyl-4-(3-methylbut-2-en-1-yl)-3-nitro-1,4-dihydropyridine (3ah).

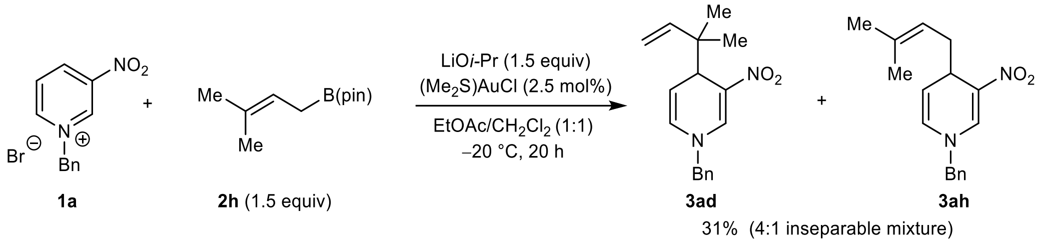

The title compounds were prepared according to General Procedure B, using azinium salt **1a** (148 mg, 0.50 mmol) and allyl pinacolboronate **2h** (166 µL, 0.75 mmol), and purified by column chromatography (5% EtOAc/*n*-pentane) to give a 4:1 mixture of inseparable regioisomers as a red oil (43.9 mg, 31%). R_f = 0.49 (20% EtOAc/petroleum ether); IR 2965, 1661, 1581 (NO_2), 1478, 1350 (NO_2), 1265, 1175, 1073, 909, 698 cm^{-1}; HRMS (ESI) Exact mass calculated for $[C_{17}H_{20}N_2NaO_2]^+$ $[M+Na]^+$: 307.1417, found 307.1418.

NMR data of major regioisomer **3ad**: ^{1}H NMR (400 MHz, $CDCl_3$) δ 8.08 (1H, t, *J* = 1.6 Hz, NC**H**=C), 7.41-7.33 (3H, m, Ar**H**), 7.24-7.21 (2H, m, Ar**H**), 6.08 (1H, dd, *J* = 7.6, 1.4 Hz, NC**H**=CH), 5.77 (1H, dd, *J* = 17.4, 10.8 Hz, CH_2=C**H**), 5.17-5.11 (1H, m, NCH=C**H**), 4.89-4.83 (2H, m, C**H**$_2$=CH), 4.50 (2H, d, *J* = 1.5 Hz, NC**H**$_2$), 3.90 (1H, dd, *J* = 6.3, 1.6 Hz, CC**H**C), 0.91 (3H, s, C**H**$_3$), 0.89 (3H, s, C**H**$_3$); ^{13}C NMR (101 MHz, $CDCl_3$) δ 145.0 (CH), 140.6 (CH), 135.3 (C), 129.2 (2 × CH), 128.7 (CH), 128.4 (CH), 127.8 (2 × CH), 123.1 (C), 112.2 (CH_2), 109.4 (CH), 58.8 (CH_2), 45.1 (C), 41.9 (CH), 22.9 (2 × CH_3).

Characteristic NMR data of minor regioisomer **3ah**: ^{1}H NMR (400 MHz, $CDCl_3$) δ 7.95 (1H, t, *J* = 1.2 Hz, NC**H**=C), 5.86 (1H, dt, *J* = 7.9, 1.3 Hz, NC**H**=CH), 4.47 (2H, s, NC**H**$_2$), 2.53-2.43 (1H, m, C**H**$_a$H$_b$CH), 2.19-2.12 (1H, m, CH$_a$**H**$_b$CH), 1.69 (3H, s, C**H**$_3$), 1.56 (3H, s, C**H**$_3$); ^{13}C NMR (101 MHz, $CDCl_3$) δ 140.7 (CH), 129.3 (2 × CH), 128.6 (CH), 127.3 (2 × CH), 127.0 (CH), 119.9 (CH), 113.4 (CH), 110.1 (C), 58.5 (CH_2), 34.2 (CH), 33.3 (CH_2), 26.1 (CH_3), 18.0 (CH_3).

(*Z*)-1-Benzyl-4-(but-2-en-1-yl)-3-nitro-1,4-dihydropyridine (3ai) and 1-benzyl-4-(but-3-en-2-yl)-3-nitro-1,4-dihydropyridine (3ae)

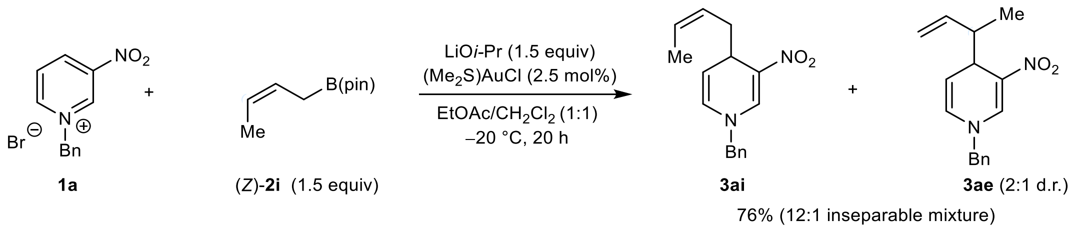

The title compounds were prepared according to General Procedure B, using azinium salt **1a** (148 mg, 0.50 mmol) and allyl pinacolboronate (*Z*)-**2i** (137 mg, 0.75 mmol), and purified by column chromatography (5% EtOAc/*n*-pentane) to give a 12:1 mixture of inseparable regioisomers as a red solid (102 mg, 76%). R_f = 0.44 (20% EtOAc/petroleum ether); m.p. 84-85 °C (Et_2O); IR 3064, 3020, 1655, 1570 (NO_2), 1496, 1365 (NO_2), 1255, 1172, 908, 696 cm^{-1}; HRMS (ESI) Exact mass calculated for $[C_{16}H_{18}N_2NaO_2]^+$ $[M+Na]^+$: 293.1260, found 293.1251.

NMR data of major regioisomer **3ai**: ^{1}H NMR (400 MHz, $CDCl_3$) δ 7.96 (1H, s, NC**H**=C), 7.42-7.32 (3H, m, Ar**H**), 7.25-7.21 (2H, m, Ar**H**), 5.87 (1H, dt, *J* = 7.9, 1.3 Hz, NC**H**=CH), 5.62-5.54 (1H, m, CH_3C**H**=CH), 5.43-4.34 (1H, m, CH_3CH=C**H**), 5.14 (1H, dd, *J* = 7.9, 5.1 Hz, NCH=C**H**), 4.47 (2H, s, NC$\mathbf{H}_2$), 3.99-3.95 (1H, m, CH_2C**H**), 2.59-2.51 (1H, m, C$\mathbf{H}_a$$H_b$CH), 2.24-2.17 (1H, m, CHa$\mathbf{H}_b$CH), 1.58-1.54 (3H, m, C$\mathbf{H}_3$); ^{13}C NMR (101 MHz, $CDCl_3$) δ 140.7 (CH), 135.4 (C), 129.3 (2 × CH), 128.7 (CH), 127.4 (2 × CH), 127.3 (CH), 127.1 (CH), 125.9 (CH), 125.2 (C), 112.8 (CH), 58.5 (CH_2), 34.0 (CH), 32.2 (CH_2), 13.1 (CH_3).

Characteristic NMR data of minor regioisomer **3ae** *as a 2:1 mixture of diastereoisomers*; *major diastereomer*: ^{1}H NMR (400 MHz, $CDCl_3$) δ 7.99 (1H, t, *J* = 1.3 Hz, NC**H**=C), 4.97-4.90 (2H, m, C$\mathbf{H}_2$=CH), 3.90-3.86 (1H, m, CHC**H**C), 2.72-2.64 (1H, m, C**H**CH_3), 1.01 (3H, d, *J* = 7.1 Hz, C$\mathbf{H}_3$); ^{13}C NMR (101 MHz, $CDCl_3$) δ 128.2 (CH), 127.6 (2 × CH), 116.1 (CH_2), 109.1 (CH), 40.5 (CH), 39.4 (CH_2), 15.9 (CH_3); *minor diastereomer*: ^{1}H NMR (400 MHz, $CDCl_3$) δ 8.04 (1H, t, *J* = 1.3 Hz, NC**H**=C), 5.07-5.01 (2H, m, C$\mathbf{H}_2$=CH), 2.80-2.74 (1H, m, C**H**CH_3), 0.93 (3H, d, *J* = 7.1 Hz, C$\mathbf{H}_3$).

Crystals suitable for X-ray analysis were prepared by slow diffusion of petroleum ether into a solution of **3ai** in CH_2Cl_2.

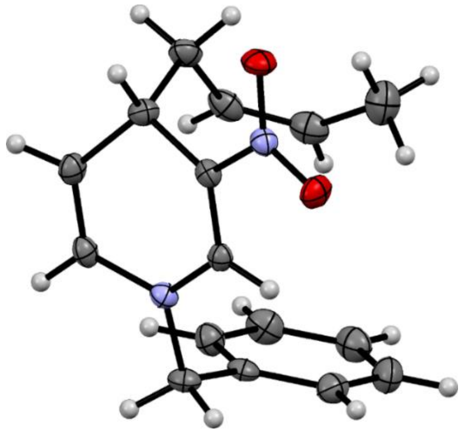

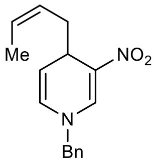

3ai
CCDC 2102858

(*Z*)-1-Benzyl-4-(but-2-en-1-yl)-3-nitro-1,4-dihydropyridine (3ai) and 1-benzyl-4-(but-3-en-2-yl)-3-nitro-1,4-dihydropyridine (3ae)

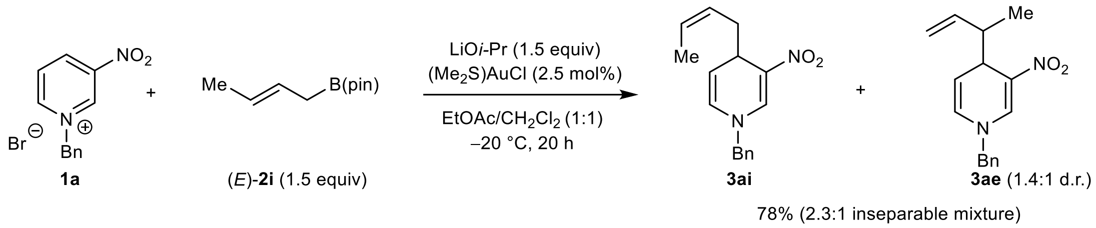

The title compounds were prepared according to General Procedure B, using azinium salt **1a** (148 mg, 0.50 mmol) and allyl pinacolboronate (*E*)-**2i** (137 mg, 0.75 mmol), and purified by column chromatography (5% EtOAc/*n*-pentane) to give a 2.3:1 mixture of inseparable regioisomers as a red solid (105 mg, 78%). R_f = 0.42 (20% EtOAc/petroleum ether); m.p. 72-73 °C (Et_2O); IR 3065, 2964, 1656, 1574 (NO_2), 1364 (NO_2), 1260, 1204, 1171, 909, 696 cm^{-1}; HRMS (ESI) Exact mass calculated for $[C_{16}H_{18}N_2NaO_2]^+$ $[M+Na]^+$: 293.1260, found 293.1259.

NMR data of major regioisomer **3ai**: ^{1}H NMR (400 MHz, $CDCl_3$) δ 7.96 (1H, s, NC**H**=C), 7.42-7.32 (3H, m, Ar**H**), 7.25-7.20 (2H, m, Ar**H**), 5.87 (1H, dt, *J* = 7.9, 1.3 Hz, NC**H**=CH), 5.62-5.53 (1H, m, CH_3C**H**=CH), 5.43-4.34 (1H, m, CH_3CH=C**H**), 5.14 (1H, dd, *J* = 7.9, 5.7 Hz, NCH=C**H**), 4.47 (2H, s, NC**H**$_2$), 3.99-3.95 (1H, m, CH_2C**H**), 2.60-2.51 (1H, m, C**H**$_a$H$_b$CH), 2.24-2.16 (1H, m, CH$_a$**H**$_b$CH), 1.58-1.53 (3H, m, C**H**$_3$); ^{13}C NMR (101 MHz, $CDCl_3$) δ 140.7 (CH), 135.4 (C), 129.26 (2 × CH), 128.7 (CH), 127.42 (2 × CH), 127.36 (CH), 127.1 (CH), 125.9 (CH), 125.2 (C), 113.1 (CH), 58.5 (CH_2), 34.0 (CH), 32.2 (CH_2), 13.1 (CH_3).

Characteristic NMR data of minor regioisomer **3ae** *as a 1.4:1 mixture of diastereoisomers*; *major diastereomer*: ^{1}H NMR (400 MHz, $CDCl_3$) δ 7.99 (1H, t, *J* = 1.3 Hz, NC**H**=C), 5.96-5.93 (1H, m, NC**H**=CH), 4.97-4.90 (2H, m, C**H**$_2$=CH), 4.46 (2H, s, NC**H**$_2$), 3.90-3.86 (1H, m, CHC**H**C), 2.72-2.65 (1H, m, C**H**CH_3), 1.01 (3H, d, *J* = 7.1 Hz, C**H**$_3$); ^{13}C NMR (101 MHz, $CDCl_3$) δ 140.75 (CH), 140.5 (CH), 135.31 (C), 129.2 (2 × CH), 128.2 (CH), 127.6 (2 × CH), 124.9 (C), 116.1 (CH_2), 109.2

(CH), 58.6 (CH_2), 40.5 (CH), 39.4 (CH_2), 15.9 (CH_3); *minor diastereomer*: ^{1}H NMR (400 MHz, $CDCl_3$) δ 8.04 (1H, t, *J* = 1.3 Hz, NC**H**=C), 5.99-5.96 (1H, m, NC**H**=CH), 5.07-5.01 (2H, m, C**H**$_2$=CH), 4.48 (2H, s, NC**H**$_2$), 2.79-2.74 (1H, m, C**H**CH_3), 0.93 (3H, d, *J* = 7.1 Hz, C**H**$_3$); ^{13}C NMR (101 MHz, $CDCl_3$) δ 140.83 (CH), 140.4 (CH), 135.27 (C), 129.32 (2 × CH), 127.5 (2 × CH), 114.8 (CH_2), 109.8 (CH), 39.0 (CH), 38.9 (CH_2), 13.4 (CH_3).

4. Uncatalyzed Nucleophilic Allylations of Azinium Salt 1a

4-Allyl-1-benzyl-3-nitro-1,4-dihydropyridine (3ab) and 2-allyl-1-benzyl-5-nitro-1,2-dihydropyridine (S6)

Using potassium allyltrifluoroborate:

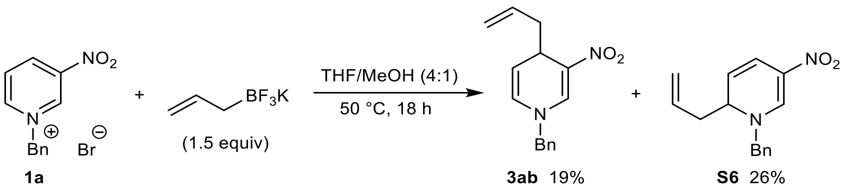

An oven-dried microwave vial fitted with a stirrer bar was charged with azinium salt **1a** (148 mg, 0.50 mmol) and potassium allyltrifluoroborate (111 mg, 0.75 mmol). The vial was sealed with a septum-lined cap and purged with argon for 30 min. THF (4.0 mL) and MeOH (1.0 mL), both of which were freshly degassed separately (purging with argon for 30 min) were added. The mixture was stirred at 50 °C for 18 h. The reaction was cooled to room temperature and then passed through a plug of silica (8 cm in height and 2 cm wide) using Et_2O (40 mL) as the eluent and the filtrate was concentrated *in vacuo*. The residue was purified by column chromatography (5% to 20% EtOAc/*n*-pentane) to give *allylated product* **3ab** as a red oil (23.9 mg, 19%) followed by *allylated product* **S6** (32.9 mg, 26%) as a yellow oil.

Data of minor regioisomer **3ab**: see above (page 26).

Data of major regioisomer **S6**: R_f = 0.40 (20% EtOAc/petroleum ether); IR 3063, 1633, 1575 (NO_2), 1492, 1420, 1288, 1172, 916, 728, 698 cm^{-1}; ^1H NMR (400 MHz, $CDCl_3$) δ 8.14 (1H, s, NC**H**=C), 7.45-7.37 (3H, m, Ar**H**), 7.29-7.25 (2H, m, Ar**H**), 6.84 (1H, dd, *J* = 10.3, 1.7 Hz, CHCH=C**H**), 5.82 (1H, dddd, *J* = 16.8, 10.4, 8.0, 6.5 Hz, CH_2=C**H**), 5.20-5.14 (2H, m, C**H**$_2$=CH), 5.07 (1H, dd, *J* = 10.3, 4.8 Hz, CHC**H**=CH), 4.56 (1H, d, *J* = 14.9 Hz, NC**H**$_a$H$_b$), 4.50 (1H, d, *J* = 14.9 Hz, NCH$_a$**H**$_b$), 4.21-4.17 (1H, m, CHC**H**N), 2.51-2.43 (1H, m, C**H**$_a$H$_b$CH), 2.32-2.24 (1H, m, CH$_a$**H**$_b$CH); ^{13}C NMR (101 MHz, $CDCl_3$) δ 146.8 (CH), 133.8 (C), 131.6 (CH), 129.5 (2 × CH), 129.1 (CH), 128.0 (2 × CH), 123.4 (C), 120.0 (CH_2), 119.3 (CH), 113.8 (CH), 59.0 (CH_2), 57.6 (CH), 39.1 (CH_2); HRMS (ESI) Exact mass calculated for $[C_{15}H_{16}N_2NaO_2]^+$ $[M+Na]^+$: 279.1104, found 279.1106.

<u>*Using allyltributylstannane:*</u>

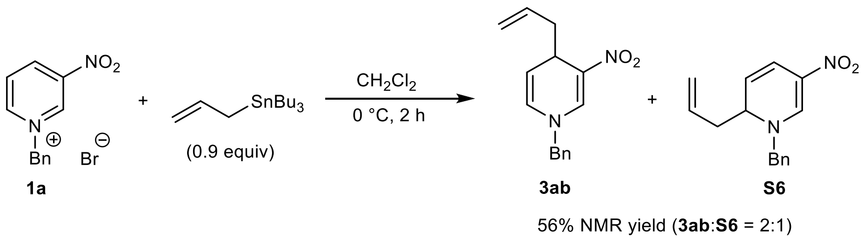

An oven-dried microwave vial fitted with a stirrer bar was charged with azinium salt **1a** (32.5 mg, 0.11 mmol). The vial was sealed with a septum-lined cap and purged with argon for 30 min. Freshly degassed (purging with argon for 30 min) CH_2Cl_2 (1.0 mL) was added, followed by the dropwise addition of allyltributylstannane (31.0 µL, 0.10 mmol) at 0 °C. The mixture was stirred at room temperature for 3 h. The reaction was passed through a plug of silica (4 cm in height and 1 cm wide) using Et_2O (20 mL) as the eluent and the filtrate was concentrated *in vacuo*. 1,3,5-Trimethoxybenzene was added as an internal standard, and the crude material was analyzed by ^{1}H NMR spectroscopy (56% yield as a 2:1 mixture of **3ab**:**S6**).

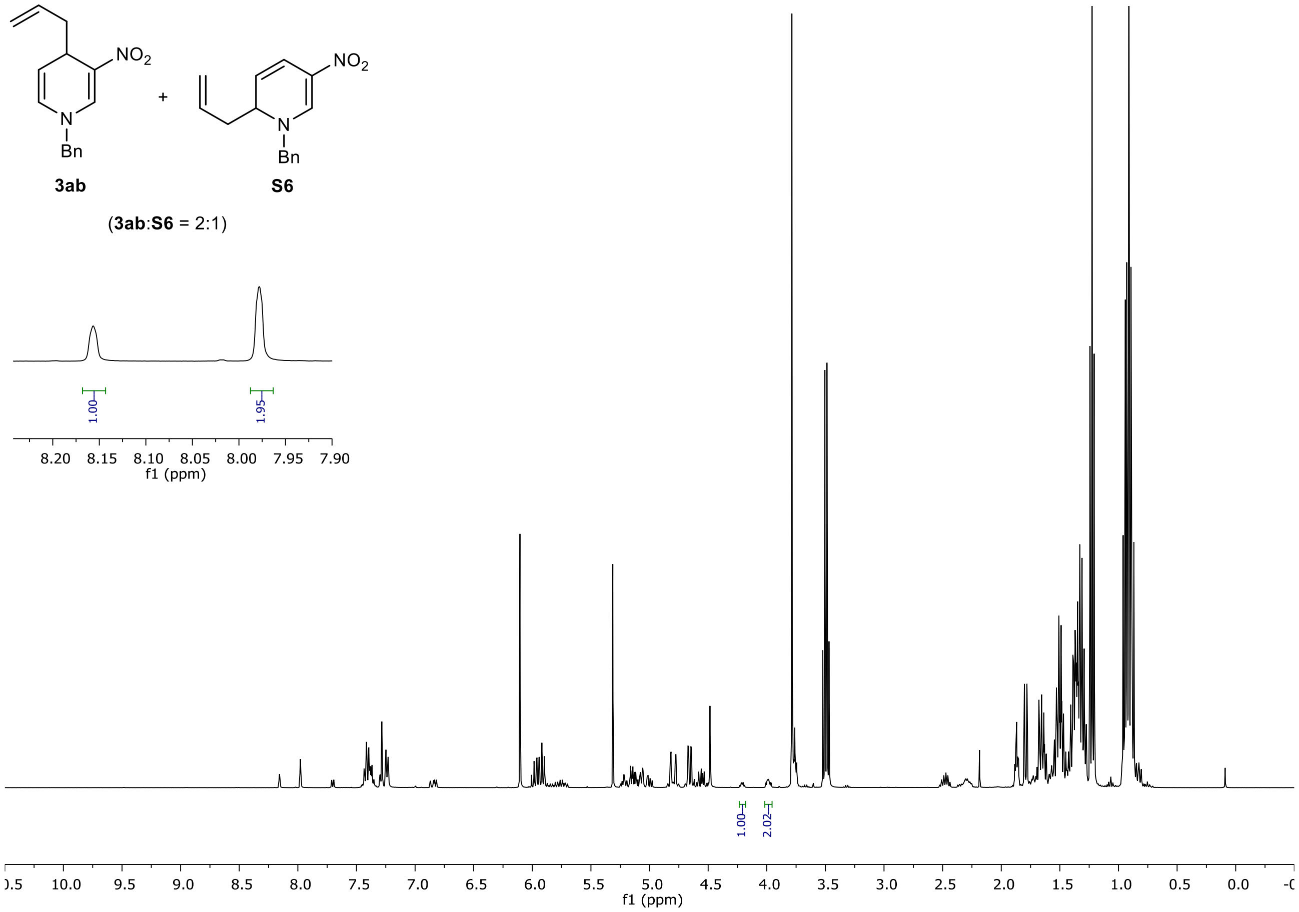

<u>*Using allylindium bromide*</u>:

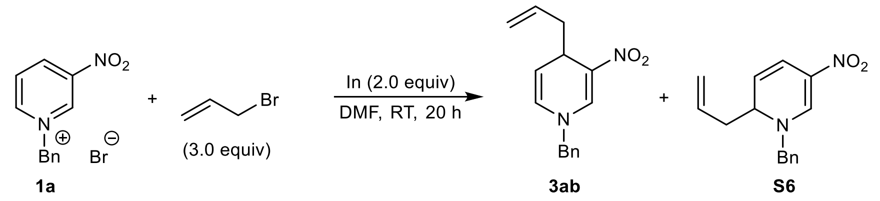

43% NMR yield (**3ab**:**S6** =1.3:1)

An oven-dried microwave vial fitted with a stirrer bar was charged with indium (23.0 mg, 0.20 mmol). The vial was sealed with a septum-lined cap and purged with argon for 30 min. A solution of allyl bromide (26.0 µL, 0.30 mmol) in freshly degassed (purging with argon for 30 min) DMF (0.5 mL) was added dropwise. The mixture was stirred at room temperature for 4 h. To a separate oven-dried microwave vial fitted with a stirrer bar was added azinium salt **1a** (29.5 mg, 0.10 mmol). The vial was sealed with a septum-lined cap and purged with argon for 30 min. Freshly degassed (purging with argon for 30 min) DMF (0.5 mL) was added, followed by the dropwise addition of the allyl indium solution (see above). The mixture was stirred at room temperature for 16 h. The reaction was passed through a plug of silica (4 cm in height and 1 cm wide) using Et_2O (20 mL) as the eluent and the filtrate was concentrated *in vacuo*. 1,3,5-Trimethoxybenzene was added as an internal standard, and the crude material was analyzed by ^{1}H NMR spectroscopy (43% yield as a 1.3:1 mixture of **3ab**:**S6**).

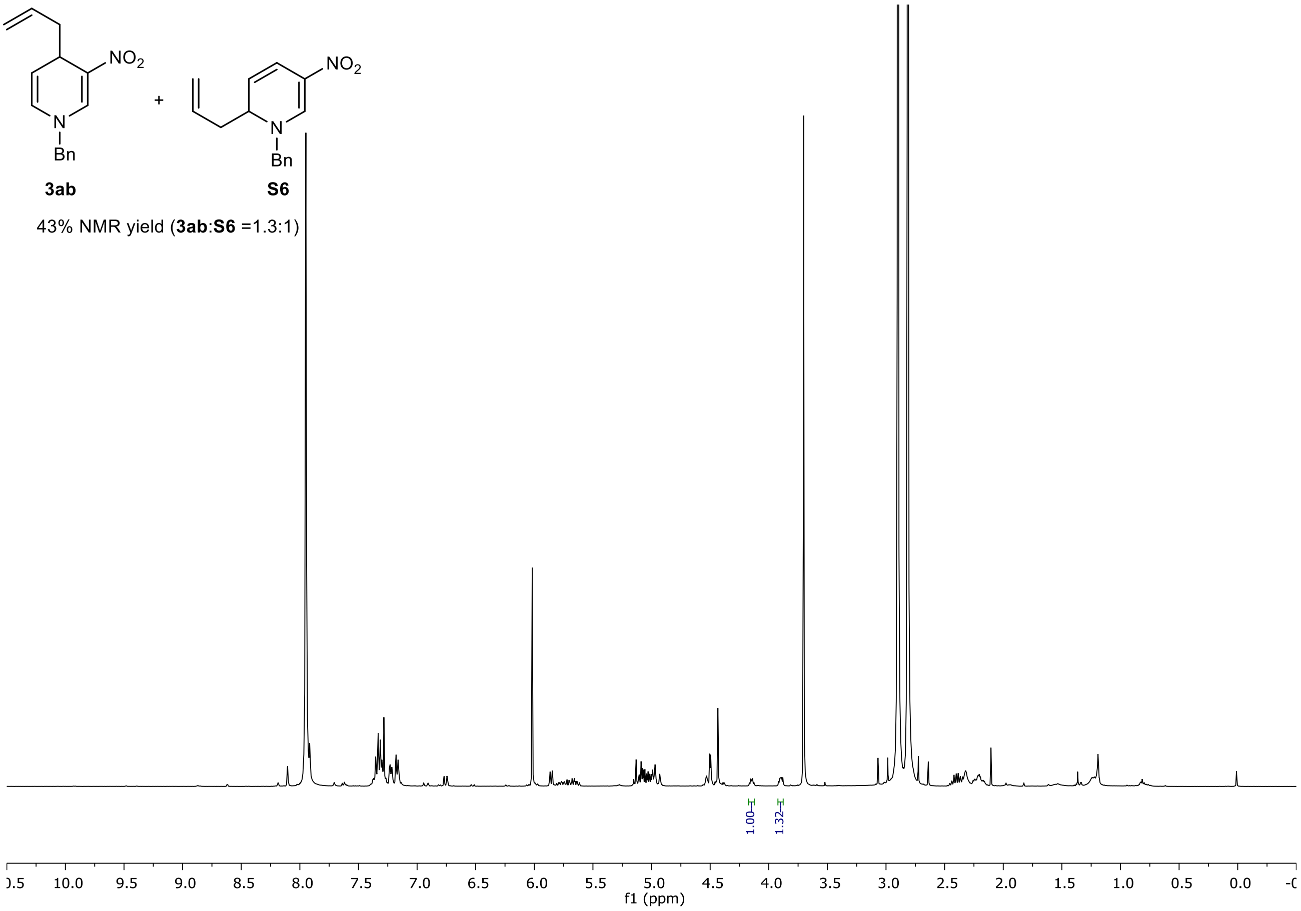

5. Allylation Using Allyl Pinacolboronate [D]$_2$-2b

4-(Allyl-1,1-d$_2$)-1-benzyl-3-nitro-1,4-dihydropyridine ([D]$_2$-3aba) and 4-(allyl-3,3-d$_2$)-1-benzyl-3-nitro-1,4-dihydropyridine ([D]$_2$-3abb).

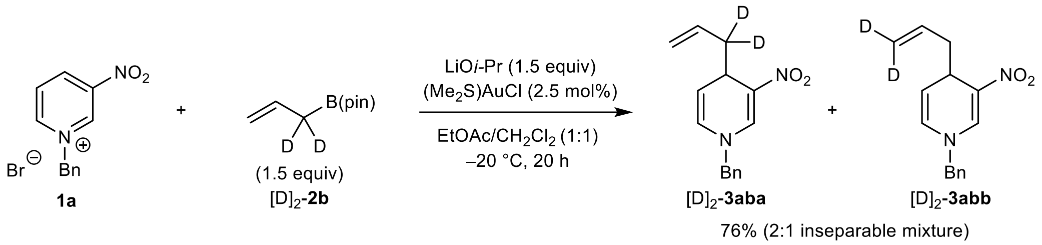

An oven-dried microwave vial fitted with a stirrer bar was charged with azinium salt **1a** (29.5 mg, 0.10 mmol), $(Me_2S)AuCl$ (0.74 mg, 0.0025 mmol) and LiO*i*-Pr (9.9 mg, 0.15 mmol). The vial was sealed with a septum-lined cap and $EtOAc/CH_2Cl_2$ (1:1) (0.6 mL) (both of which were undried, obtained from commercial vendors and used without further purification) was added. The mixture was stirred at –20 °C for 5 min and a solution of dideuterated allyl pinacolboronate [D]$_2$-**2b** (contaminated with 15% vinylboronic acid pinacol ester, which is consistent with previously reported literature[4–6]) (29.3 mg, 0.15 mmol) in 0.4 mL of $EtOAc/CH_2Cl_2$ (1:1) was added dropwise. The resulting solution was stirred at –20 °C for 20 h. The reaction was warmed to room temperature and then passed through a plug of silica (4 cm in height and 1 cm wide) using Et_2O (20 mL) as the eluent and the filtrate was concentrated *in vacuo*. Purification of the residue by column chromatography (5% EtOAc/*n*-pentane) gave a 2:1 mixture of *allylated products* [D]$_2$-**3aba** and [D]$_2$-**3abb** as a red oil (19.6 mg, 76%). R_f = 0.40 (20% EtOAc/petroleum ether); IR 2925, 1667, 1585 (NO_2), 1356 (NO_2), 1264, 1173, 1076, 915, 730, 698 cm^{-1}; ^1H NMR (400 MHz, $CDCl_3$) δ 7.96 (1H, t, J = 1.1 Hz, NC**H**=C), 7.42-7.33 (3H, m, Ar**H**), 7.24-7.20 (2H, m, Ar**H**), 5.89 (1H, dt, J = 7.9, 1.2 Hz, NC**H**=CH), 5.73 (1H, dd, J = 16.9, 10.2 Hz, CH_2=C**H** of [D]$_2$-**3aba** and CD_2=C**H** of [D]$_2$-**3abb**), 5.13 (1H, dd, J = 7.9, 5.1 Hz, NCH=C**H**), 5.07-5.00 (1.33H, m, C**H**$_2$=CH of [D]$_2$-**3aba**), 4.47 (2H, s, NC**H**$_2$), 3.99-3.95 (1H, m, CHC**H**C), 2.45 (0.33H, ddd, J = 13.7, 8.3, 6.6 Hz, C**H**$_a$H$_b$CH of [D]$_2$-**3abb**), 2.26 (0.33H, ddd, J = 13.7, 6.6, 3.4 Hz, CH$_a$**H**$_b$CH of [D]$_2$-**3abb**); ^2H NMR (77 MHz, $CHCl_3$) δ 5.13-5.01 (2D, m, C**D**$_2$=C of [D]$_2$-**3abb**), 2.44 (1D, s, C**D**$_a$D$_b$CH of [D]$_2$-**3aba**), 2.25 (1D, s, CD$_a$**D**$_b$CH of [D]$_2$-**3aba**); ^{13}C NMR (101 MHz, $CDCl_3$) δ 140.7 (CH), 135.3 (C), 134.3 (CH), 134.2 (CH), 129.3 (2 × CH), 128.7 (CH), 127.5 (2 × CH), 127.1 (CH), 125.0 (C), 118.2 (CH_2), 112.8 (CH), 58.5 (CH_2), 39.3 (CH_2) 33.7 (CH), 33.6 (CH); HRMS (ESI) Exact mass calculated for $[C_{15}H_{15}D_2N_2O_2]^+$ $[M+H]^+$: 259.1410, found 259.1409.

6. Allylations Using Allylboronate 2j

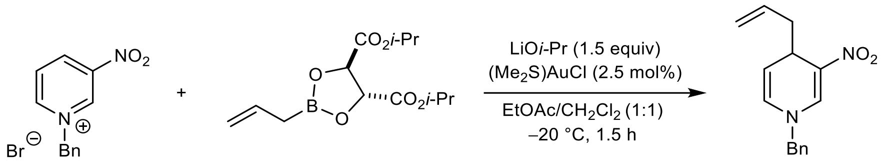

1a **2j** (1.5 equiv) **3ab** 88%, 0% ee

An oven-dried microwave vial fitted with a stirrer bar was charged with azinium salt **1a** (29.5 mg, 0.10 mmol), $(Me_2S)AuCl$ (0.74 mg, 0.0025 mmol) and LiO*i*-Pr (9.9 mg, 0.15 mmol). The vial was sealed with a septum-lined cap and $EtOAc/CH_2Cl_2$ (1:1) (0.6 mL) (both of which were undried, obtained from commercial vendors and used without further purification) was added. The mixture was stirred at –20 °C for 5 min and a solution of allyl pinacolboronate **2j** (contaminated with 32% (+)-diisopropyl L-tartrate, which is consistent with previously reported literature[7]) (56.2 mg, 0.15 mmol) in 0.4 mL of $EtOAc/CH_2Cl_2$ (1:1) was added dropwise. The resulting solution was stirred at –20 °C for 1.5 h. The reaction was warmed to room temperature and then passed through a plug of silica (4 cm in height and 1 cm wide) using Et_2O (20 mL) as the eluent and the filtrate was concentrated *in vacuo*. 1,3,5-Trimethoxybenzene was added as an internal standard, and the crude material was analyzed by ^{1}H NMR spectroscopy (88% yield). Enantiomeric excess was determined by HPLC using a Chiralcel IC column (80:20 *iso*-hexane:*i*-PrOH, 1.0 mL/min, 210 nm, 25 °C): t_r = 27.2 and 34.5 min, 0% ee.

HPLC trace using allyl pinacolboronate **2b**

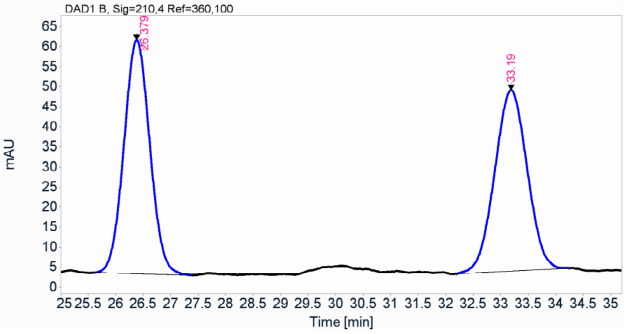

Signal: DAD1 B, Sig=210,4 Ref=360,100

RT [min]	Type	Width [min]	Area	Height	Area%
26.379	BB	0.4948	1912.465	58.2828	50.77
33.190	BB	0.5953	1854.317	45.1389	49.23

HPLC trace using allylboronate **2j**

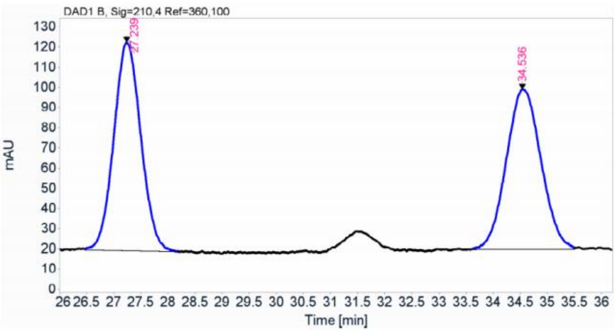

Signal: DAD1 B, Sig=210,4 Ref=360,100

RT [min]	Type	Width [min]	Area	Height	Area%
27.239	VB	0.4640	3567.040	103.0003	50.28
34.536	VV	0.5381	3527.187	79.1174	49.72

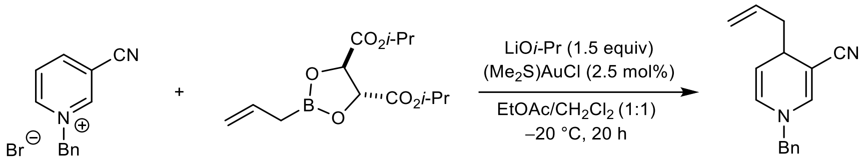

1b **2j** (1.5 equiv) **3bb** 52%, 0% ee

An oven-dried microwave vial fitted with a stirrer bar was charged with azinium salt **1b** (29.5 mg, 0.10 mmol), $(Me_2S)AuCl$ (0.74 mg, 0.0025 mmol) and Li*Oi*-Pr (9.9 mg, 0.15 mmol). The vial was sealed with a septum-lined cap and $EtOAc/CH_2Cl_2$ (1:1) (0.6 mL) (both of which were undried, obtained from commercial vendors and used without further purification) was added. The mixture was stirred at –20 °C for 5 min and a solution of allyl pinacolboronate **2j** (contaminated with 32% (+)-diisopropyl L-tartrate, which is consistent with previously reported literature[7]) (56.2 mg, 0.15 mmol) in 0.4 mL of $EtOAc/CH_2Cl_2$ (1:1) was added dropwise. The resulting solution was stirred at –20 °C for 20 h. The reaction was warmed to room temperature and then passed through a plug of silica (4 cm in height and 1 cm wide) using Et_2O (20 mL) as the eluent and the filtrate was concentrated *in vacuo*. 1,3,5-Trimethoxybenzene was added as an internal standard, and the crude material was analyzed by ^{1}H NMR spectroscopy (52% yield). Enantiomeric excess was determined by HPLC using a Chiralcel IC column (90:10 *iso*-hexane:*i*-PrOH, 1.0 mL/min, 230 nm, 25 °C): t_r = 19.3 and 20.2 min, 0% ee.

HPLC trace using allyl pinacolboronate **2b**

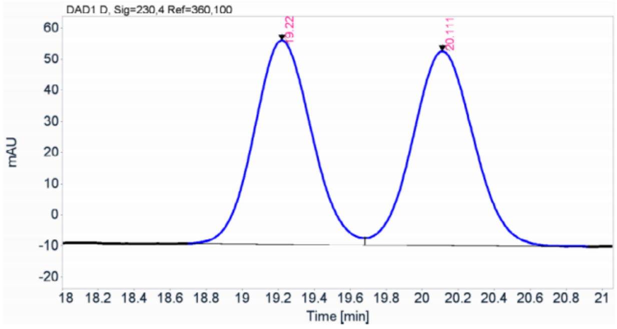

Signal: DAD1 D, Sig=230,4 Ref=360,100

RT [min]	Type	Width [min]	Area	Height	Area%
19.220	BV	0.3444	1451.283	65.5285	49.74
20.111	VB	0.3539	1466.676	62.4817	50.26

HPLC trace using allylboronate **2j**

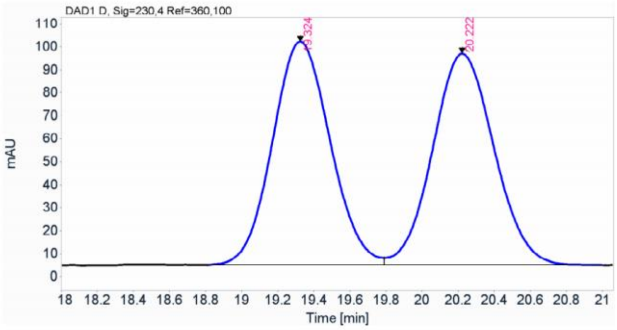

Signal: DAD1 D, Sig=230,4 Ref=360,100

RT [min]	Type	Width [min]	Area	Height	Area%
19.324	BV	0.3475	2160.957	97.1660	49.83
20.222	VB	0.3595	2175.803	92.1442	50.17

7. Allylation in the Presence of TEMPO

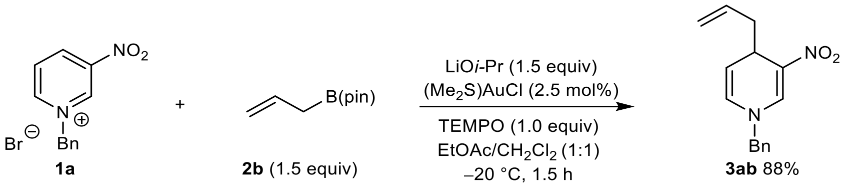

An oven-dried flask equipped with a stirrer bar was charged with azinium salt **1a** (29.5 mg, 0.10 mmol), $(Me_2S)AuCl$ (0.74 mg, 0.0025 mmol), TEMPO (15.6 mg, 0.10 mmol) and LiO-*i*Pr (9.9 mg, 0.15 mmol). The vial was sealed and $EtOAc/CH_2Cl_2$ (1:1) (0.6 mL) (both of which were undried, obtained from commercial vendors and used without further purification) was added. The mixture was stirred at –20 °C for 5 min and a solution of the allyl pinacolboronate **2b** (28.1 µL, 0.15 mmol) in 0.4 mL of $EtOAc/CH_2Cl_2$ (1:1) was added dropwise. The resulting solution was stirred at –20 °C for 1.5 h. The reaction was warmed to room temperature and then passed through a plug of silica (4 cm in height and 1 cm wide) using Et_2O (20 mL) as the eluent and the filtrate was concentrated *in vacuo*. 1,3,5-Trimethoxybenzene was added as an internal standard, and the crude material was analyzed by ^1H NMR spectroscopy (88% yield).

8. ^{1}H NMR and ^{11}B NMR Study of Transmetalation from Allylboronate 2b

(a) First, the ^{1}H and ^{11}B NMR spectra of allylboronate **2b** in DMSO-d_6 were measured:

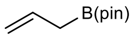

2b

^{1}H NMR (400 MHz, DMSO-d_6) δ 5.76 (1H, ddt, *J* = 17.4, 10.1, 7.5 Hz, CH$_2$=C**H**), 4.95-4.90 (1H, m, C**H**$_a$H$_b$=), 4.85 (1H, ddt, *J* = 10.1, 2.4, 1.3 Hz, CH$_a$**H**$_b$=), 1.61 (2H, dt, *J* = 7.5, 1.5 Hz, C**H**$_2$B), 1.18 (12H, s, 2 × C(C**H**$_3$)$_2$); ^{11}B NMR (DMSO-d_6, 128 MHz) δ 32.7.

Pictures of the NMR spectra are in the stacked plots on subsequent pages.

(b) Next, the NMR spectra of a 1:1 mixture of allylboronate **2b** (0.10 mmol) and LiO*i*-Pr (0.10 mmol) in DMSO-d_6 were measured.

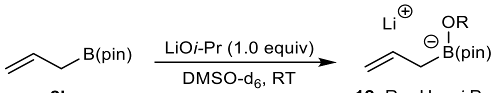

2b **12** R = H or *i*-Pr

In the ^{1}H NMR spectrum, compared with the spectrum of **2b** alone, small changes were observed, which may indicate the formation of boronate species **12**. However, we hesitated to draw firm conclusions from these data alone because there was considerable broadening of the signals, resulting from the incomplete solubility of LiO*i*-Pr.

In the ^{11}B NMR spectrum, however, two main peaks were observed, indicative of remaining **2b** (32.4 ppm) and a boronate species **12** (7.1 ppm; though this signal is quite broad) containing either a hydroxide group or isopropoxide group (hydroxide ions will be formed from the reaction of LiO*i*-Pr with the significant amount of H_2O present in DMSO-d_6). These two chemical shifts are consistent with those observed in previous NMR studies of the reaction of allylboronate **2b** with KOMe.[21,22]
^{11}B NMR (DMSO-d_6, 128 MHz) δ 32.4, 7.1.

Pictures of the NMR spectra are in the stacked plots on subsequent pages.

(c) Finally, a 1:1:1 mixture of allylboronate **2b**, LiO*i*-Pr, and (Me$_2$S)AuCl was prepared in DMSO-d_6 by the following procedure:

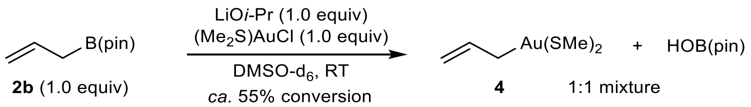

2b (1.0 equiv) **4** 1:1 mixture

To an NMR tube was added a solution of (Me$_2$S)AuCl (29.5 mg, 0.10 mmol) and LiO*i*-Pr (6.6 mg, 0.10 mmol) in DMSO-d_6 (0.5 mL). Allylboronate **2b** (18.8 µL, 0.10 mmol) was added and the mixture was analyzed by ^{1}H and ^{11}B NMR spectroscopy.

Full consumption of allylboronate **2b** was not observed, likely because of the incomplete solubility of LiO*i*-Pr. However, NMR spectroscopy showed new signals that are consistent with the formation of allylgold(I) species **4**, along with HOBpin, in a 1:1 ratio (*ca.* 55% conversion). In the ^{1}H NMR spectrum, a new multiplet appeared at 2.11-2.08 ppm, attributable to the methylene protons adjacent to gold, and a broad singlet appeared at 7.99 ppm, attributable to the hydroxyl proton of HOB(pin). In the ^{11}B NMR spectrum, a new signal appeared at 22.2 ppm, consistent with HOB(pin) [and possibly a trace quantity of *i*-PrOB(pin)]. *i*-PrOB(pin) originates from LiO*i*-Pr, but because DMSO-d$_6$ contains a significant quantity of H_2O, which would react with LiO*i*-Pr to give LiOH, HOB(pin) is expected as the major boron-containing by-product. The formation of ClB(pin) (*ca.* 28 ppm)[23,24] was not observed.

*NMR data for **4***: ^{1}H NMR (400 MHz, DMSO-d$_6$) δ 5.81-5.71 (1H, m, =C**H**), 5.04-4.98 (1H, m, C**H**$_a$H$_b$=), 4.97-4.92 (1H, m, CH$_a$**H**$_b$=), 2.11-2.08 (2H, m, C**H**$_2$Au), 2.03 (6H, s, S(C**H**$_3$)$_2$).

NMR data for HOB(pin): ^{1}H NMR (400 MHz, DMSO-d$_6$) δ 7.99 (1H, s, O**H**), 1.14 (12H, s, 2 × C(C**H**$_3$)$_2$); ^{11}B NMR (DMSO-d$_6$, 128 MHz) δ 22.2. The NMR data are consistent with those reported previously[25] and identical to those of an authentic sample.

Pictures of the NMR spectra follow below.

^{1}H NMR Spectra (Stacked Plot)

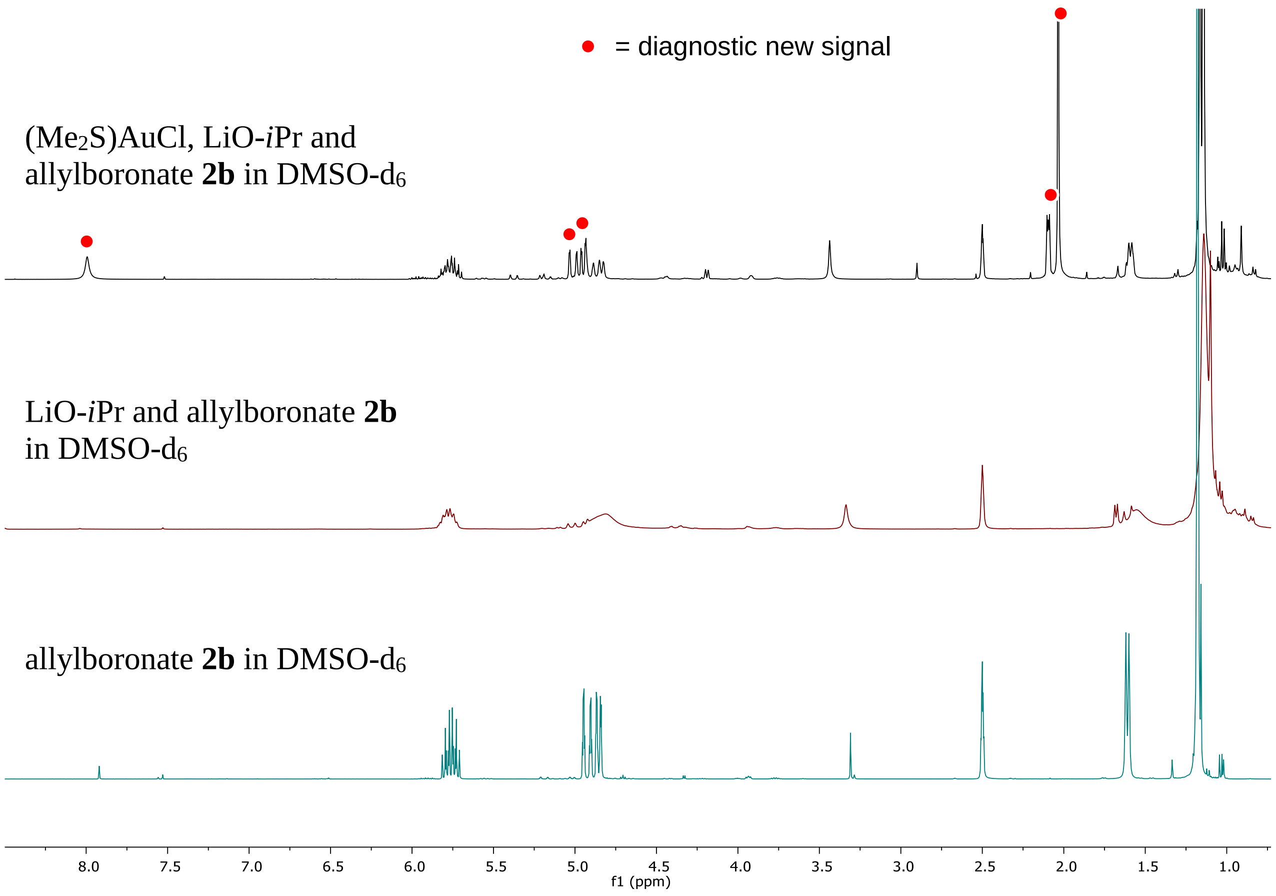

Annotated ^{1}H NMR Spectrum

$(Me_2S)AuCl$, LiO-*i*Pr and allylboronate **2b** in DMSO-d_6

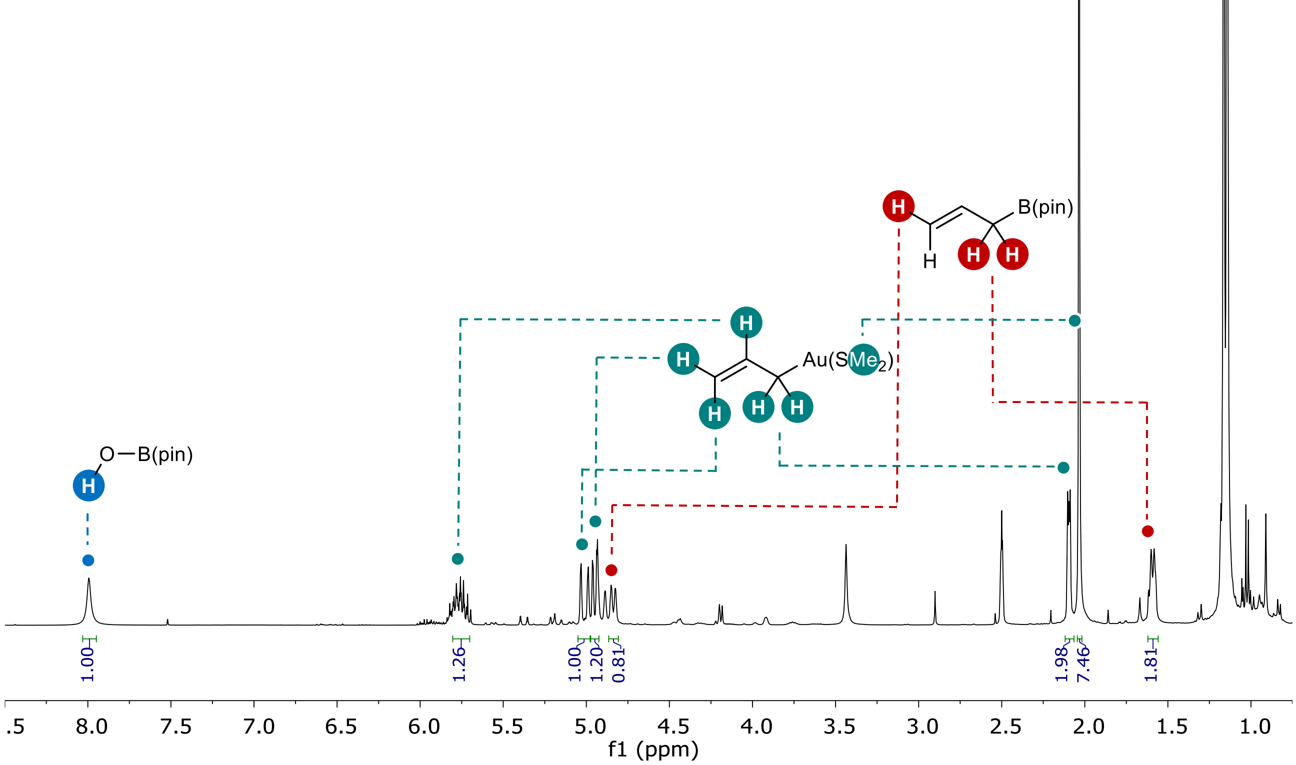

^{11}B NMR Spectra

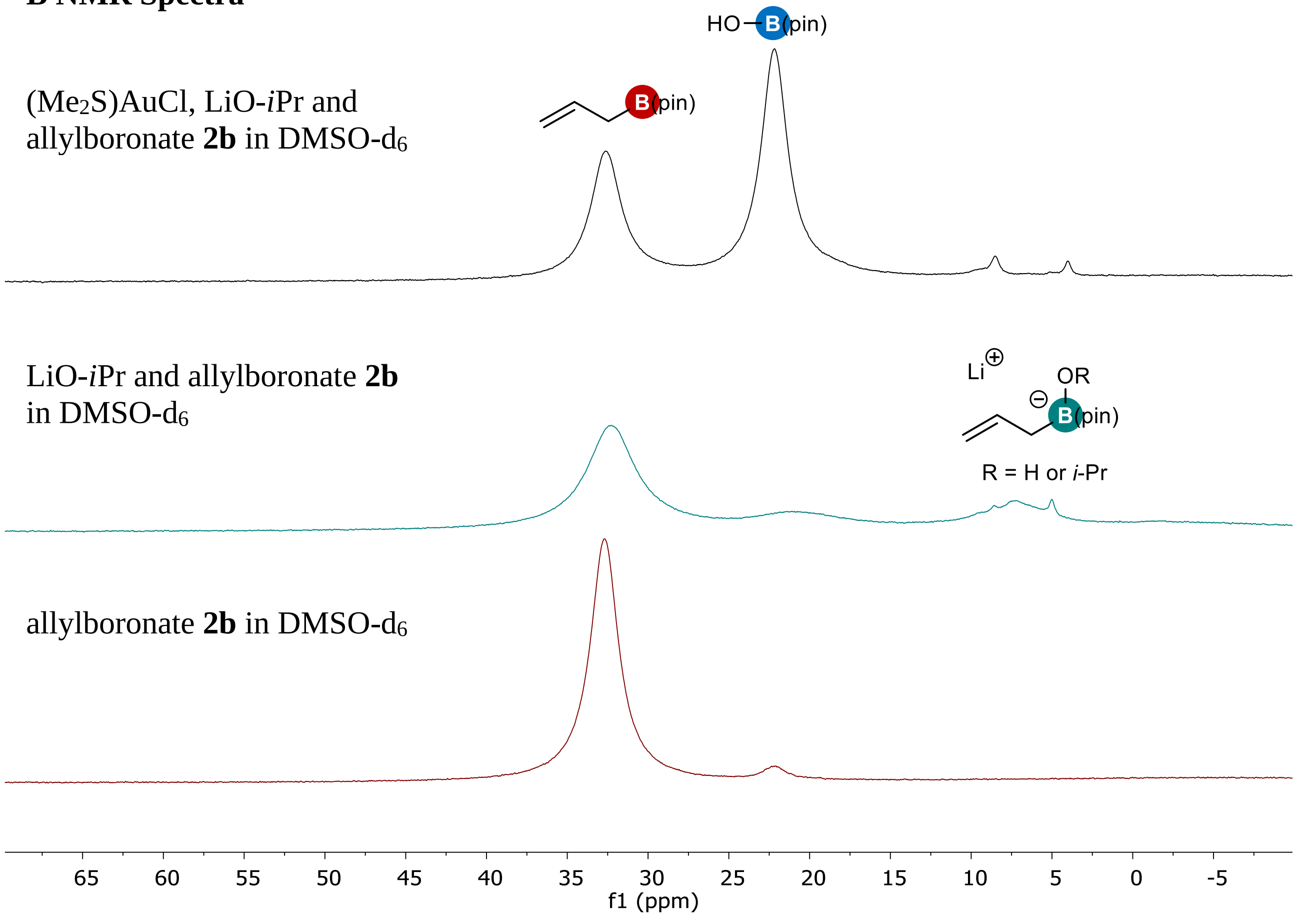

An additional experiment was also conducted where $(Me_2S)AuCl$ and LiO*i*-Pr (1.0 equiv) were mixed in DMSO-d_6 for 30 min in the absence of allyl pinacolboronate and the ^1H NMR spectrum was measured. The ^1H NMR spectrum was complex, and no firm conclusions could be drawn (e.g. regarding equilibria between gold chloride, hydroxide, and isopropoxide species). Subsequent addition of allyl pinacolboronate to this mixture led to essentially identical results as observed previously, i.e., observation of a 1:1 mixture of an allylgold complex and HOB(pin).

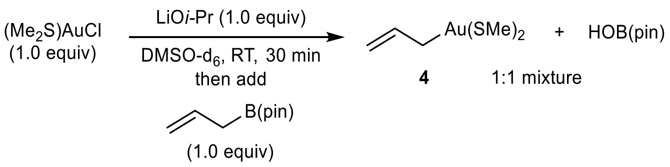

The relevant ^1H NMR spectra are shown below:

$(Me_2S)AuCl$ in DMSO-d_6

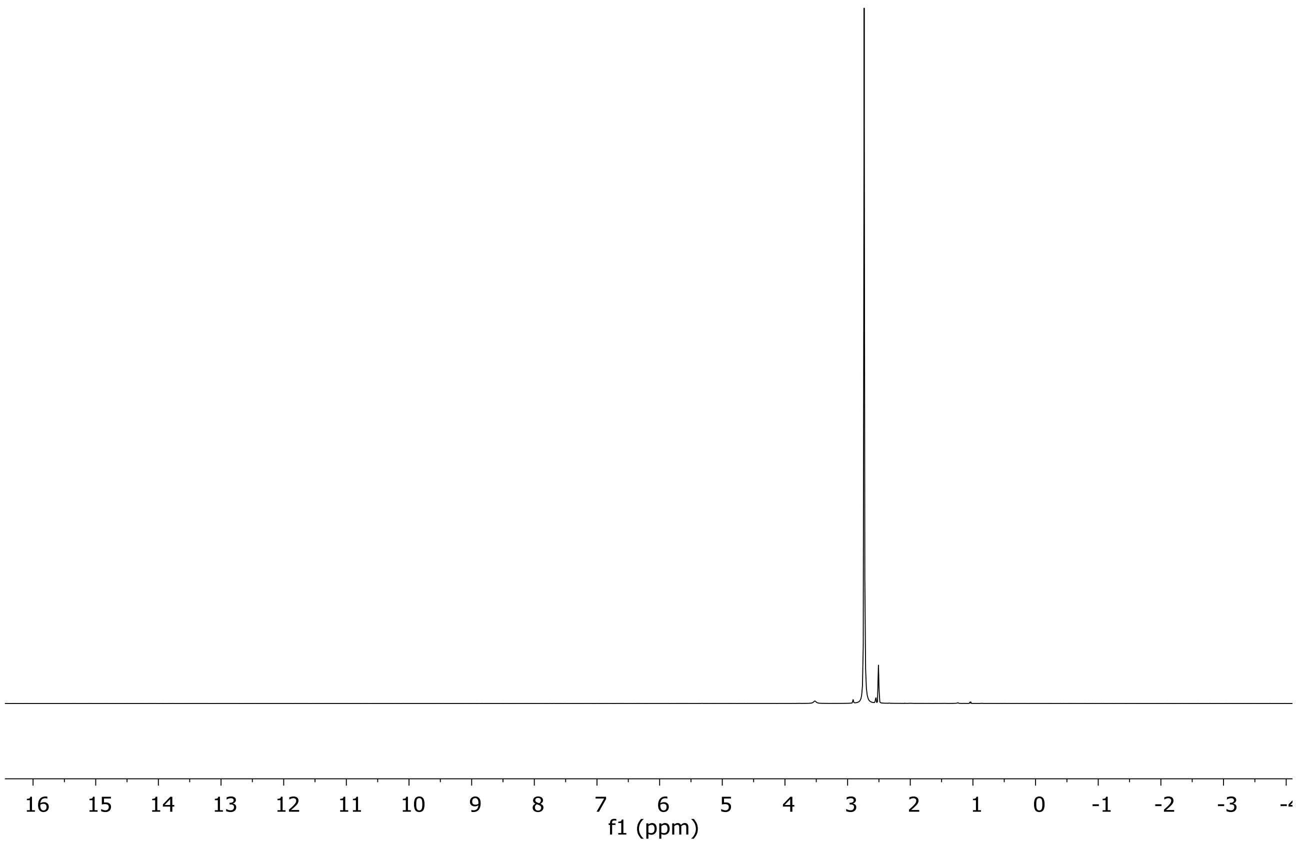

LiO*i*-Pr in DMSO-d_6

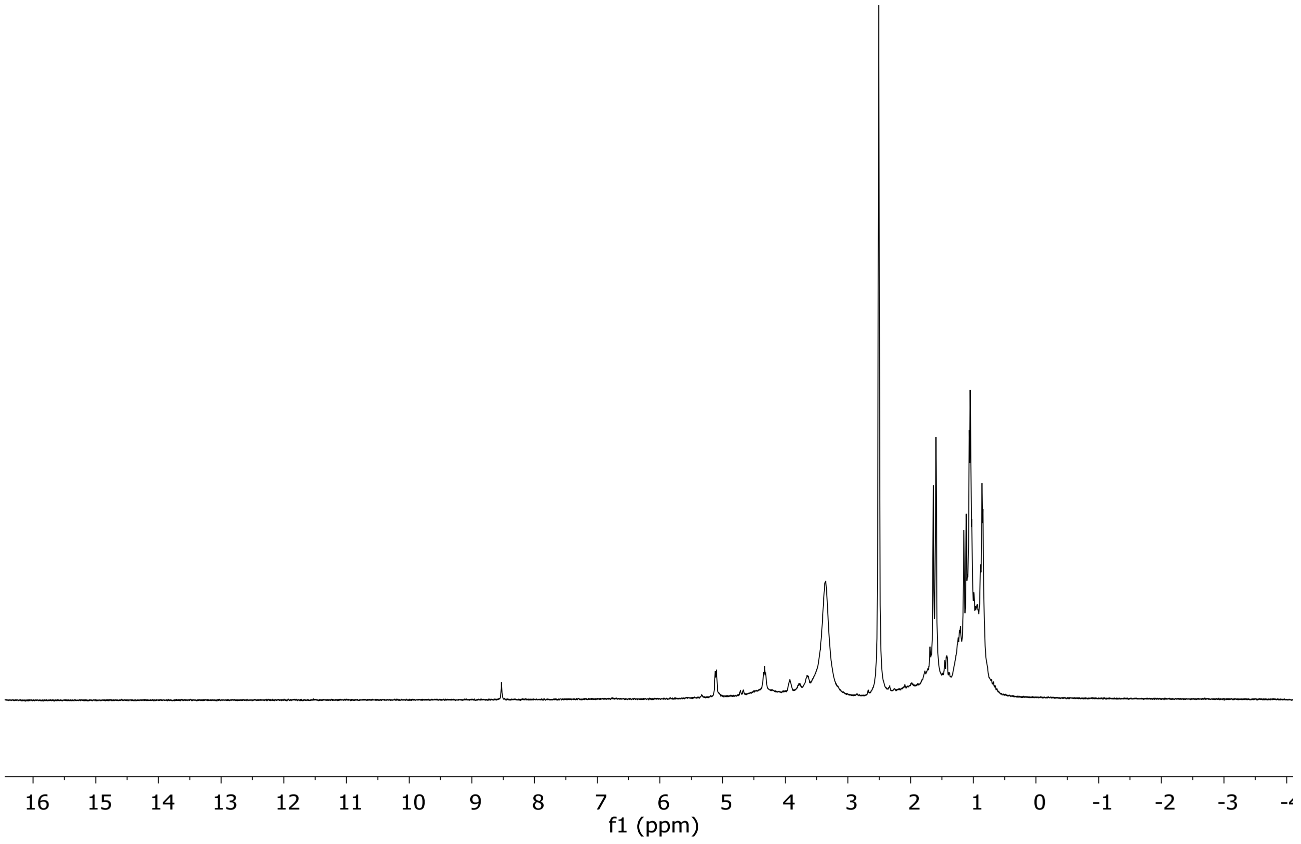

(Me_2S)AuCl and LiO-*i*Pr after stirring for 30 min in DMSO-d_6

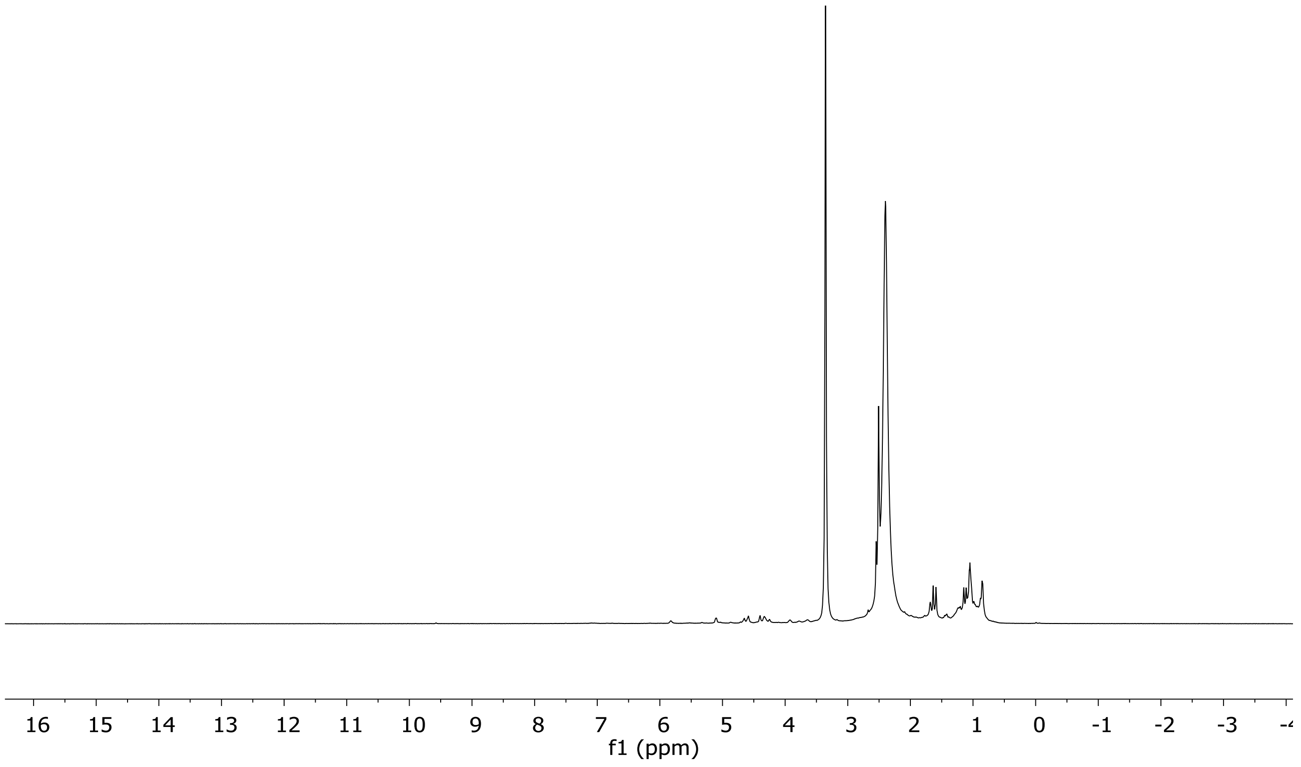

$(Me_2S)AuCl$ and LiO-*i*Pr after stirring for 30 min in DMSO-d_6, followed by the addition of allyl pinacolboronate (compare with the spectrum above)

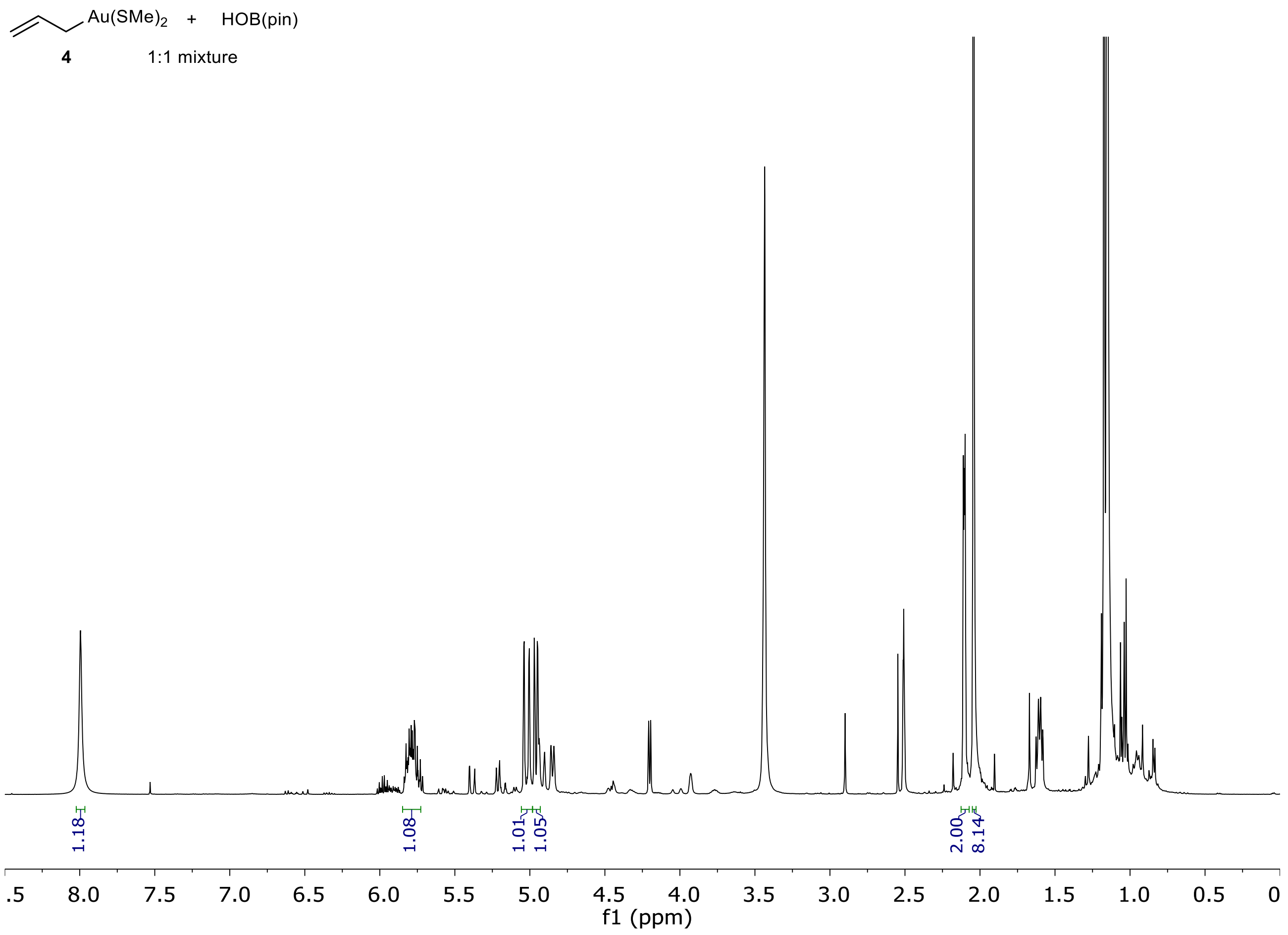

9. Computational Studies

Computational Methods

All quantum mechanical calculations were carried out using Gaussian 16.[26] All DFT structure optimizations and frequency calculations were done with the implicit SMD solvent model.[27] The molecular geometries were optimized at the DFT level of theory using the PBE0 functional[28] with the def2-TZVP basis set.[29] Frequency calculations were performed on all structures and confirmed to contain no imaginary frequencies or just one imaginary frequency for ground states and transition states, respectively. The free energies were corrected using quasi-harmonic approximation and corrections were done using the GoodVibes script.[30] The full set of DFT output files with optimized structures and frequencies are included as part of the Supporting Information (see below for a description) and can found at https://doi.org/10.17639/nott.7173.

Additional Pathways Investigated

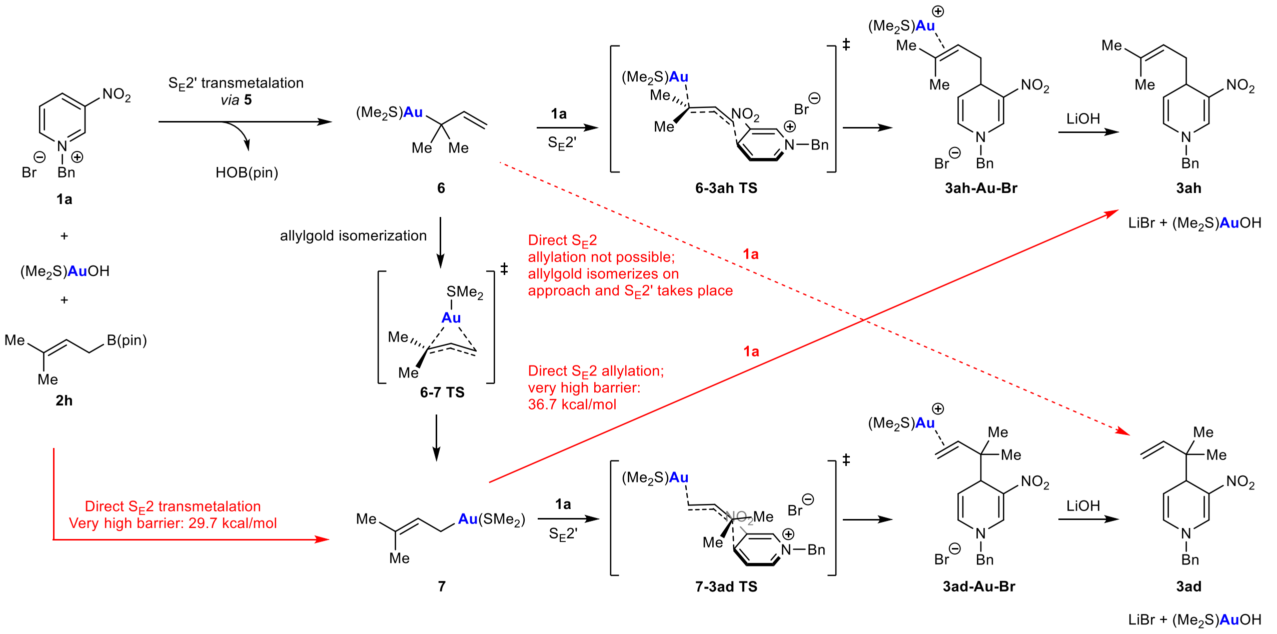

Scheme S1. Additional possible mechanistic pathways leading from **1a** and **2h** to products **3ah** and **3ad**, as explored computationally (shown in **red**). Barriers shown are free energies of activation relative to the preceding intermediates and calculated at PBE0/def2-TZVP/SMD(CH_2Cl_2).

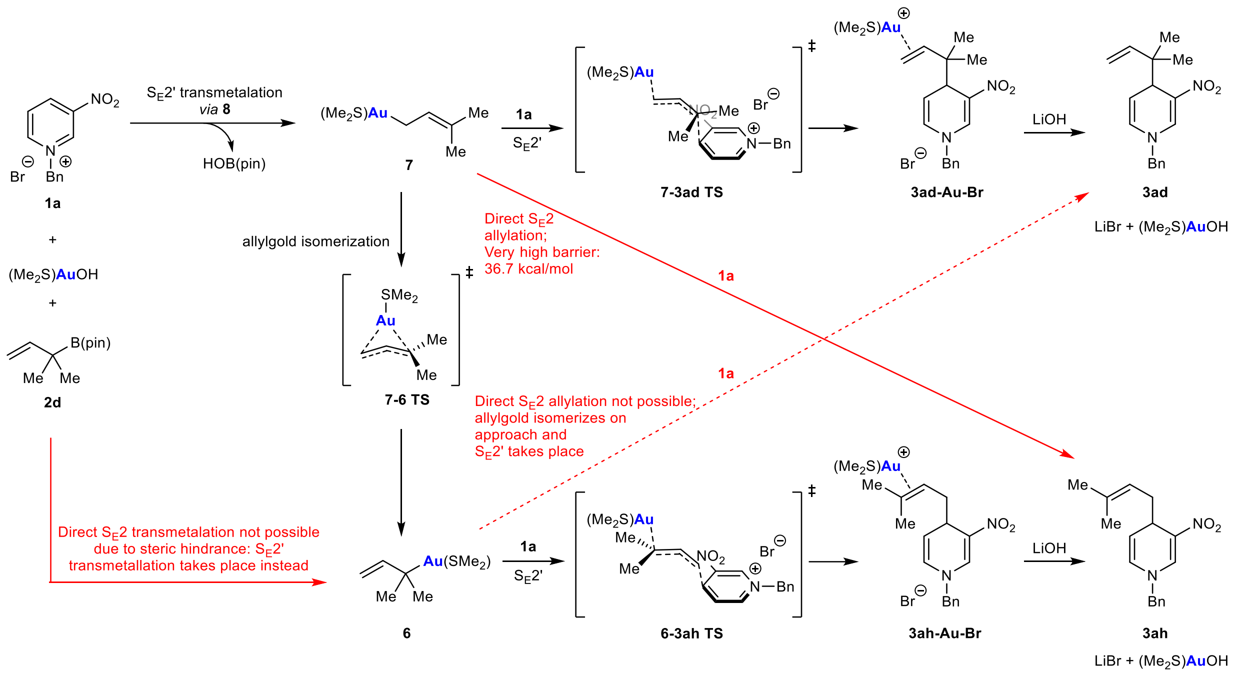

Scheme S2. Additional possible mechanistic pathways leading from **1a** and **2d** to products **3ad** and **3ah**, as explored computationally (shown in **red**). Barriers shown are free energies of activation relative to the preceding intermediates and calculated at PBE0/def2-TZVP/SMD(CH_2Cl_2).

Summary of the Associated Computational Dataset Contents

This dataset contains Gaussian DFT output files of the key ground states and transition state DFT optimized structures. The data are organized in one archive containing 40 separate folders; the full structure of the archive is shown below. All folder and file names refer to the compound numbers used in the main paper.

There are three top level folders. '**Catalysts**' contain the files from computational comparison of likely active catalysts. **'1a_2h_reaction'** contains computational files from the study of the reaction between **1a** and **2h** (Scheme 3 in main paper text). **'1a_2d_reaction**' contains computational files from the study of the reaction between **1a** and **2d** (Scheme 4 in main paper text).

Each of the lower-level folders contain the output of a frequency calculation at PBE0/def2-TZVP level with SMD(CH_2Cl_2) solvent model (*_freq.out files). All optimized geometries are also provided as *.sdf files for even better usability.

All the files can be opened in any text editor. Gaussian output structures can be viewed and the frequency modes visualised in GausView, Avogadro, jmol and in most other molecular viewers/editors. *.sdf files can be viewed in essentially all 3D molecular editors and viewers.

```
├── 1a_2d_reaction
│   ├── 1a
│   │   ├── 1a_freq.out
│   │   └── 1a.sdf
│   ├── 2d
│   │   ├── 2d_freq.out
│   │   └── 2d.sdf
│   ├── 2d-8_TS
│   │   ├── 2d-8_TS_freq.out
│   │   └── 2d-8_TS.sdf
│   ├── 3ad
│   │   ├── 3ad_freq.out
│   │   └── 3ad.sdf
│   ├── 3ad-Au-Br
│   │   ├── 3ad-Au-Br_freq.out
│   │   └── 3ad-Au-Br.sdf
│   ├── 3ah
│   │   ├── 3ah_freq.out
│   │   └── 3ah.sdf
│   ├── 3ah-Au-Br
│   │   ├── 3ah-Au-Br_freq.out
│   │   └── 3ah-Au-Br.sdf
│   ├── 6
│   │   ├── 6_freq.out
│   │   └── 6.sdf
│   ├── 6-3ah_TS
│   │   ├── 6-3ah_TS_freq.out
│   │   └── 6-3ah_TS.sdf
│   ├── 7
│   │   ├── 7_freq.out
│   │   └── 7.sdf
│   ├── 7-3ad_TS
```

```
│   │   ├── 7-3ad_TS_freq.out
│   │   └── 7-3ad_TS.sdf
│   ├── 7-6_TS
│   │   ├── 7-6_TS_freq.out
│   │   └── 7-6_TS.sdf
│   ├── 8
│   │   ├── 8_freq.out
│   │   └── 8.sdf
│   ├── 8-7_TS
│   │   ├── 8-7_TS_freq.out
│   │   └── 8-7_TS.sdf
│   ├── LiBr
│   │   ├── LiBr_freq.out
│   │   └── LiBr.sdf
│   ├── LiOH
│   │   ├── LiOH_freq.out
│   │   └── LiOH.sdf
│   └── Me2SAuOH
│       ├── Me2SAuOH_freq.out
│       └── Me2SAuOH.sdf
├── 1a_2h_reaction
│   ├── 1a
│   │   ├── 1a_freq.out
│   │   └── 1a.sdf
│   ├── 2h
│   │   ├── 2h_freq.out
│   │   └── 2h.sdf
│   ├── 2h-5_TS
│   │   ├── 2h-5_TS_freq.out
│   │   └── 2h-5_TS.sdf
│   ├── 3ad
│   │   ├── 3ad_freq.out
│   │   └── 3ad.sdf
```

```
│   ├── 3ad-Au-Br
│   │   ├── 3ad-Au-Br_freq.out
│   │   └── 3ad-Au-Br.sdf
│   ├── 3ah
│   │   ├── 3ah_freq.out
│   │   └── 3ah.sdf
│   ├── 3ah-Au-Br
│   │   ├── 3ah-Au-Br_freq.out
│   │   └── 3ah-Au-Br.sdf
│   ├── 5
│   │   ├── 5_freq.out
│   │   └── 5.sdf
│   ├── 5-6_TS
│   │   ├── 5-6_TS_freq.out
│   │   └── 5-6_TS.sdf
│   ├── 6
│   │   ├── 6_freq.out
│   │   └── 6.sdf
│   ├── 6-3ah_TS
│   │   ├── 6-3ah_TS_freq.out
│   │   └── 6-3ah_TS.sdf
│   ├── 6-7_TS
│   │   ├── 6-7_TS_freq.out
│   │   └── 6-7_TS.sdf
│   ├── 7
│   │   ├── 7_freq.out
│   │   └── 7.sdf
│   ├── 7-3ad_TS
│   │   ├── 7-3ad_TS_freq.out
│   │   └── 7-3ad_TS.sdf
│   ├── LiBr
│   │   ├── LiBr_freq.out
```

```
│   │   └── LiBr.sdf
│   ├── LiOH
│   │   ├── LiOH_freq.out
│   │   └── LiOH.sdf
│   └── Me2SAuOH
│       ├── Me2SAuOH_freq.out
│       └── Me2SAuOH.sdf
├── Catalysts
│   ├── Me2SAuCl
│   │   ├── Me2SAuCl_freq.out
│   │   └── Me2SAuCl.sdf
│   ├── Me2SAuOH
│   │   ├── Me2SAuOH_freq.out
│   │   └── Me2SAuOH.sdf
│   └── Me2SAuOiPr
│       ├── Me2SAuOiPr_freq.out
│       └── Me2SAuOiPr.sdf
└── DFTDataReadme
```

10. Further Transformations

(±)-(3*S*,4*R*)-4-Allyl-1-benzyl-3-nitropiperidine (9)

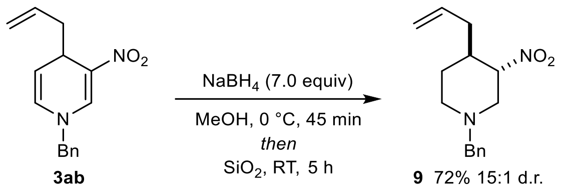

To a solution of 1,4-dihydropyridine **3ab** (128 mg, 0.50 mmol) in MeOH (3 mL) at 0 °C was added $NaBH_4$ (132 mg, 3.50 mmol) portionwise. The resulting reaction mixture was stirred at 0 °C for 45 min. The reaction was quenched with saturated aqueous NH_4Cl solution (10 mL) and extracted with CH_2Cl_2 (15 mL). The organic layer was washed with saturated aqueous NH_4Cl solution (10 mL), dried ($MgSO_4$), filtered, and concentrated *in vacuo*. The residue was dissolved in CH_2Cl_2 (10 mL) and SiO_2 (10.0 g) was added. The resulting suspension was concentrated *in vacuo* and left standing absorbed on SiO_2 at room temperature for 5 h. $CHCl_3$ (75 mL) was added, and the slurry was stirred for an additional 30 min. The slurry was filtered using EtOAc (50 mL) as the eluent and the filtrate was concentrated *in vacuo* to leave the title compound **9** as a 15:1 mixture of inseparable diastereomers as a pale-yellow oil (93.2 mg, 72%). R_f = 0.31 (10% EtOAc/petroleum ether); IR 2921, 2812, 1552 (NO_2), 1453, 1357 (NO_2), 1163, 983, 916, 735, 698 cm^{-1}; HRMS (ESI) Exact mass calculated for $[C_{15}H_{21}N_2O_2]^+$ $[M+H]^+$: 261.1598, found 261.1602.

NMR data of major diastereomer: ^{1}H NMR (400 MHz, $CDCl_3$) δ 7.34-7.24 (5H, m, Ar**H**), 5.71 (1H, ddt, *J* = 17.0, 10.5, 7.1 Hz, CH_2=C**H**), 5.09-5.03 (2H, m, C$\mathbf{H}_2$=), 4.41 (1H, td, *J* = 10.4, 4.1 Hz, C**H**NO_2), 3.59 (1H, d, *J* = 13.1 Hz, C$\mathbf{H}_a$$H_b$Ph), 3.51 (1H, d, *J* = 13.1 Hz, $CH_a$$\mathbf{H}_b$Ph), 3.24 (1H, ddd, *J* = 10.4, 4.1, 1.7 Hz, NC$\mathbf{H}_a$$H_b$CH), 2.89-2.84 (1H, m, NC$\mathbf{H}_a$$H_b$$CH_2$), 2.43 (1H, t, *J* = 10.4 Hz, N$CH_a$$\mathbf{H}_b$CH), 2.23-2.16 (1H, m, CH_2=CHC$\mathbf{H}_a$$H_b$), 2.12-2.03 (2H, m, N$CH_a$$\mathbf{H}_b$$CH_2$ and C**H**CHN), 2.02-1.94 (1H, m, CH_2=CH$CH_a$$\mathbf{H}_b$), 1.84 (1H, dq, *J* = 13.8, 3.2 Hz, NCH_2C$\mathbf{H}_a$$CH_b$), 1.42-1.32 (1H, m, N$CH_2$$CH_aC\mathbf{H}_b$); ^{13}C NMR (101 MHz, $CDCl_3$) δ 137.5 (C), 134.0 (CH), 129.1 (2 × CH), 128.53 (2 × CH), 127.5 (CH), 118.3 (CH_2), 87.8 (CH), 62.5 (CH_2), 56.6 (CH_2), 52.6 (CH_2), 39.1 (CH), 36.6 (CH_2), 28.8 (CH_2).

Characteristic NMR data of minor diastereomer: ^{1}H NMR (400 MHz, $CDCl_3$) δ 4.61-4.57 (1H, m, C**H**N), 2.77-2.70 (1H, m, NC$\mathbf{H}_a$$H_b$CH), 2.60-2.54 (1H, m, NC$\mathbf{H}_a$$H_b$$CH_2$), 1.63 (1H, dq, *J* = 13.8, 4.6 Hz, NCH_2C$\mathbf{H}_a$$H_b$); ^{13}C NMR (101 MHz, $CDCl_3$) δ 137.8 (C), 135.3 (CH), 128.8 (2 × CH), 128.47 (2 × CH), 127.4 (CH), 117.8 (CH_2), 83.7 (CH), 26.8 (CH_2).

The relative configuration of **7** was determined by ^{1}H NMR spectroscopy, on the basis of the coupling constant of the α-proton adjacent to the nitro substituent at 4.41 ppm (^2J = 10.4 Hz). This coupling

constant indicates a *trans*-diaxial relationship, allowing the two bulky substituents to occupy the more favorable equatorial positions.

(±)-3-[(3*S*,4*R*)-1-Benzyl-3-nitropiperidin-4-yl]propan-1-ol (10)

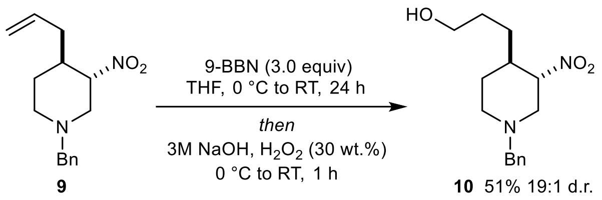

To a solution of alkene **9** (26.0 mg, 0.10 mmol) in THF (0.5 mL) at 0 °C was added 9-BBN (0.5 M in THF, 600 µL, 0.30 mmol). The resulting solution was warmed to room temperature and stirred for 24 h. The reaction was cooled to 0 °C and 3 M aqueous NaOH (0.3 mL) and H_2O_2 (30 wt.% in H_2O, 0.5 mL) were added. The mixture was warmed to room temperature and stirred for 1 h. The reaction was diluted with H_2O (5 mL), extracted with EtOAc (3 × 10 mL), and the combined organic layers were dried ($MgSO_4$), filtered, and concentrated *in vacuo*. Purification of the residue by column chromatography (10 to 30% EtOAc/*n*-pentane) gave the title compound **10** as a 19:1 mixture of inseparable diastereomers as a colorless oil (14.3 mg, 51%). R_f = 0.21 (40% EtOAc/petroleum ether); IR 3364 (OH), 2924, 2815, 1558 (NO_2), 1344 (NO_2), 1173, 1055, 910, 734, 698 cm^{-1}; HRMS (ESI) Exact mass calculated for $[C_{15}H_{23}N_2O_3]^+$ $[M+H]^+$: 279.1703, found 279.1701.

NMR data of major diastereomer: ^{1}H NMR (400 MHz, $CDCl_3$) δ 7.34-7.25 (5H, m, Ar**H**), 4.40 (1H, td, *J* = 10.5, 4.0 Hz, C**H**NO_2), 3.66-3.57 (3H, m, HOC**H**$_2$ and C**H**$_a$H$_b$Ph), 3.52 (1H, d, *J* = 13.1 Hz, CH$_a$**H**$_b$Ph), 3.24 (1H, ddd, *J* = 10.5, 4.0, 1.7 Hz, NC**H**$_a$H$_b$CH), 2.91-2.87 (1H, m, NC**H**$_a$H$_b$$CH_2$), 2.43 (1H, t, *J* = 10.5 Hz, NCH$_a$**H**$_b$CH), 2.10 (1H, td, *J* = 11.8, 2.8 Hz, NCH$_a$**H**$_b$$CH_2$), 2.05-1.96 (1H, m, C**H**CHN), 1.93-1.87 (1H, m, NCH_2C**H**$_a$H$_b$), 1.70-1.58 (2H, m, HOCH_2C**H**$_a$H$_b$ and **H**OCH_2), 1.52-1.43 (2H, m, HOCH_2CH$_a$**H**$_b$ and HO$CH_2$$CH_2$C**H**$_aH_b$), 1.38-1.28 (2H, m, HO$CH_2$$CH_2CH_a$**H**$_b$ and NCH_2CH$_a$**H**$_b$); ^{13}C NMR (101 MHz, $CDCl_3$) δ 137.5 (C), 129.1 (2 × CH), 128.6 (2 × CH), 127.6 (CH), 88.6 (CH), 62.8 (CH_2), 62.5 (CH_2), 56.6 (CH_2), 52.7 (CH_2), 39.5 (CH), 29.1 (CH_2), 29.0 (CH_2), 28.5 (CH_2).

Characteristic NMR data of minor diastereomer: ^{1}H NMR (400 MHz, $CDCl_3$) δ 4.64-4.59 (1H, m, C**H**N), 2.74-2.67 (1H, m, NC**H**$_a$H$_b$CH), 2.65-2.59 (1H, m, NC**H**$_a$H$_b$$CH_2$).

Ethyl 1-benzyl-4-isobutyl-1,4,5,6-tetrahydropyridine-3-carboxylate (11)

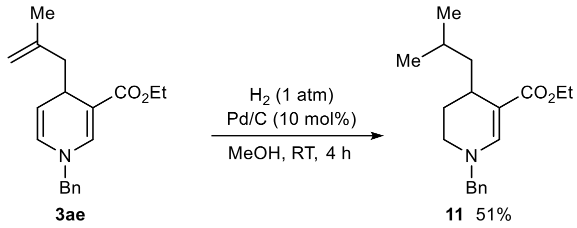

To a solution of 1,4-dihydropyridine **3ae** (297 mg, 1.00 mmol) in MeOH (10 mL) was added 10% Pd/C (106 mg, 0.10 mmol). The flask was sealed, evacuated, and backfilled with hydrogen (3 cycles) and the solution was stirred at room temperature for 4 h. The reaction was filtered and concentrated *in vacuo*. Purification of the residue by column chromatography (1 to 3% EtOAc/*n*-pentane) gave the title compound **11** as a yellow oil (154 mg, 51%). R_f = 0.34 (10% EtOAc/petroleum ether); IR 2952, 2867, 1674 (C=O), 1599, 1361, 1263, 1193, 1148, 729, 697 cm^{-1}; ^{1}H NMR (500 MHz, $CDCl_3$) δ 7.54 (1H, s, NC**H**=C), 7.37-7.33 (2H, m, Ar**H**), 7.31-7.28 (1H, m, Ar**H**), 7.22-7.19 (2H, m, Ar**H**), 4.33 (1H, d, *J* = 15.3 Hz, C$\mathbf{H}_a$$H_b$Ph), 4.29 (1H, d, *J* = 15.3 Hz, C$H_a$$\mathbf{H}_b$Ph), 4.21-4.08 (2H, m, OC$\mathbf{H}_2$), 3.01 (1H, td, *J* = 12.7, 3.7 Hz, NC$\mathbf{H}_a$$H_b$$CH_2$), 2.92-2.88 (1H, m, NC$H_a$$\mathbf{H}_b$$CH_2$), 2.71 (1H, dtt, *J* = 9.3, 4.6, 1.5 Hz, C**H**C=C), 1.73 (1H, ddt, *J* = 13.3, 3.9, 2.2 Hz, NCH_2C$\mathbf{H}_a$$H_b$), 1.67-1.56 (2H, m, N$CH_2CH_a$$\mathbf{H}_b$ and $(CH_3)_2$C**H**), 1.34 (1H, dddd, *J* = 13.6, 9.4, 4.2, 0.9 Hz, $(CH_3)_2$CHC$\mathbf{H}_a$$H_b$), 1.27 (3H, t, *J* = 7.1 Hz, CH_2C$\mathbf{H}_3$), 1.06 (1H, dddd, *J* = 14.0, 9.7, 4.7, 0.9 Hz, $(CH_3)_2$CHC$H_a$$\mathbf{H}_b$), 0.94 (3H, d, *J* = 6.5 Hz, $(C\mathbf{H}_3)_2$CH), 0.89 (3H, d, *J* = 6.5 Hz, $(C\mathbf{H}_3)_2$CH); ^{13}C NMR (126 MHz, $CDCl_3$) δ 168.9 (C), 145.7 (CH), 137.3 (C), 128.9 (2 × CH), 127.9 (CH), 127.5 (2 × CH), 100.1 (C), 59.9 (CH_2), 59.0 (CH_2), 45.4 (CH_2), 41.4 (CH_2), 27.0 (CH), 25.1 (CH), 24.5 (CH_2), 24.1 (CH_3), 21.9 (CH_3), 14.8 (CH_3); HRMS (ESI) Exact mass calculated for $[C_{19}H_{27}NNaO_2]^+$ $[M+Na]^+$: 324.1934, found 324.1927.

11. NMR Spectra of New Compounds

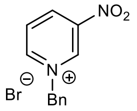

1a

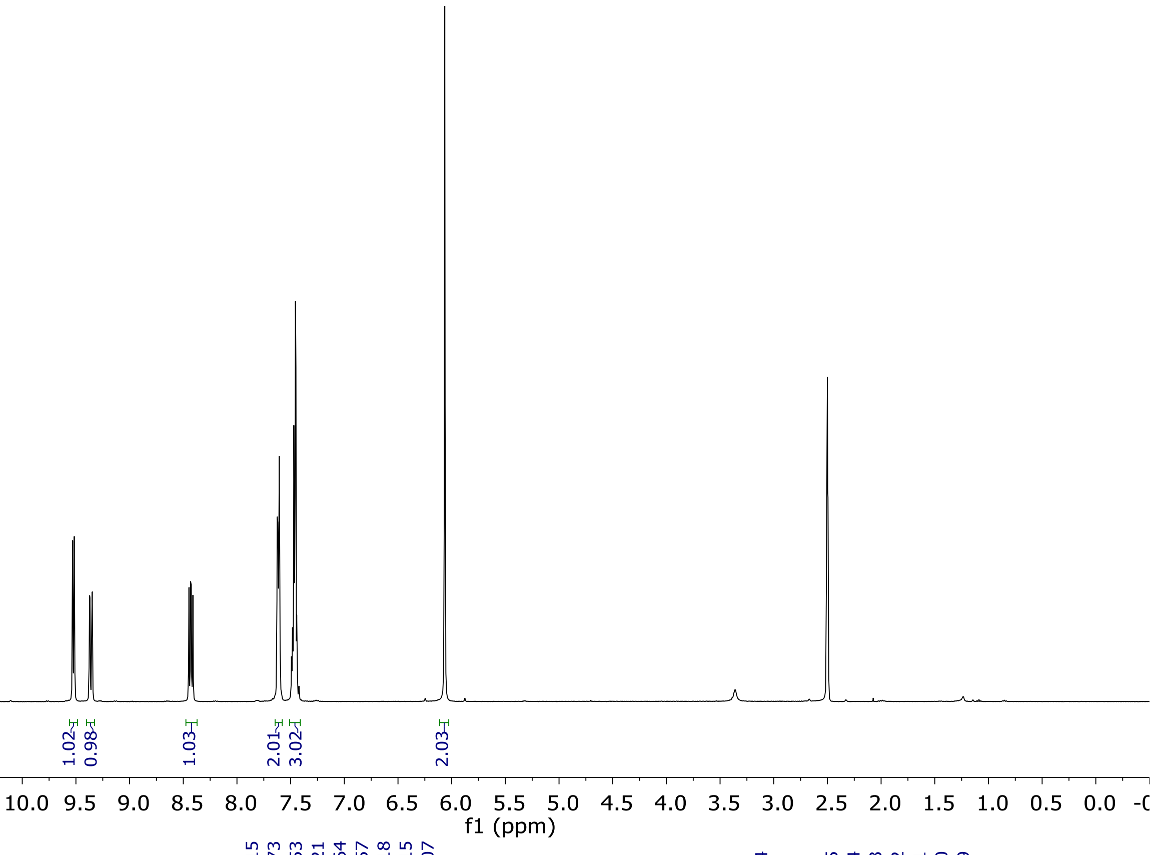

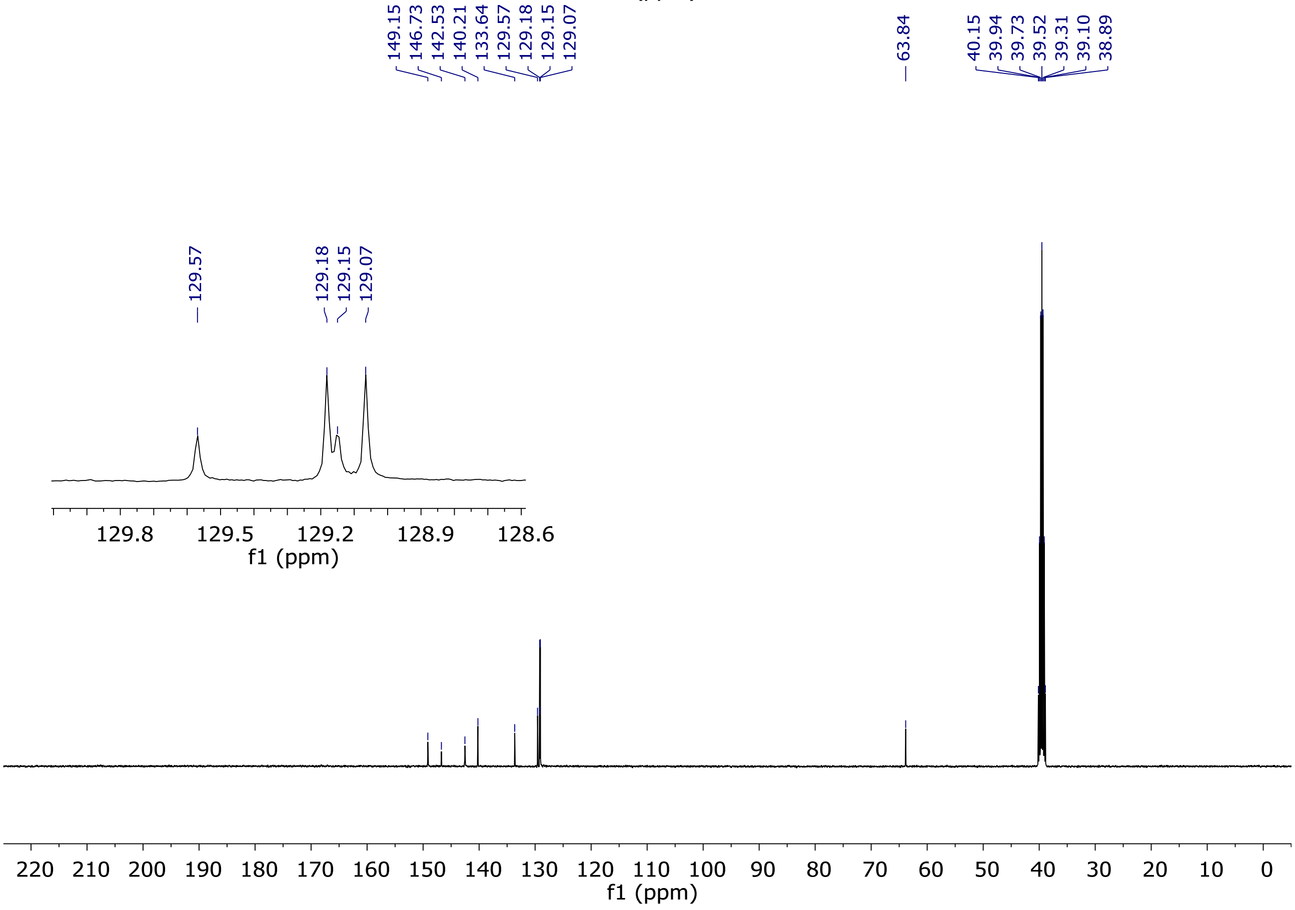

1f

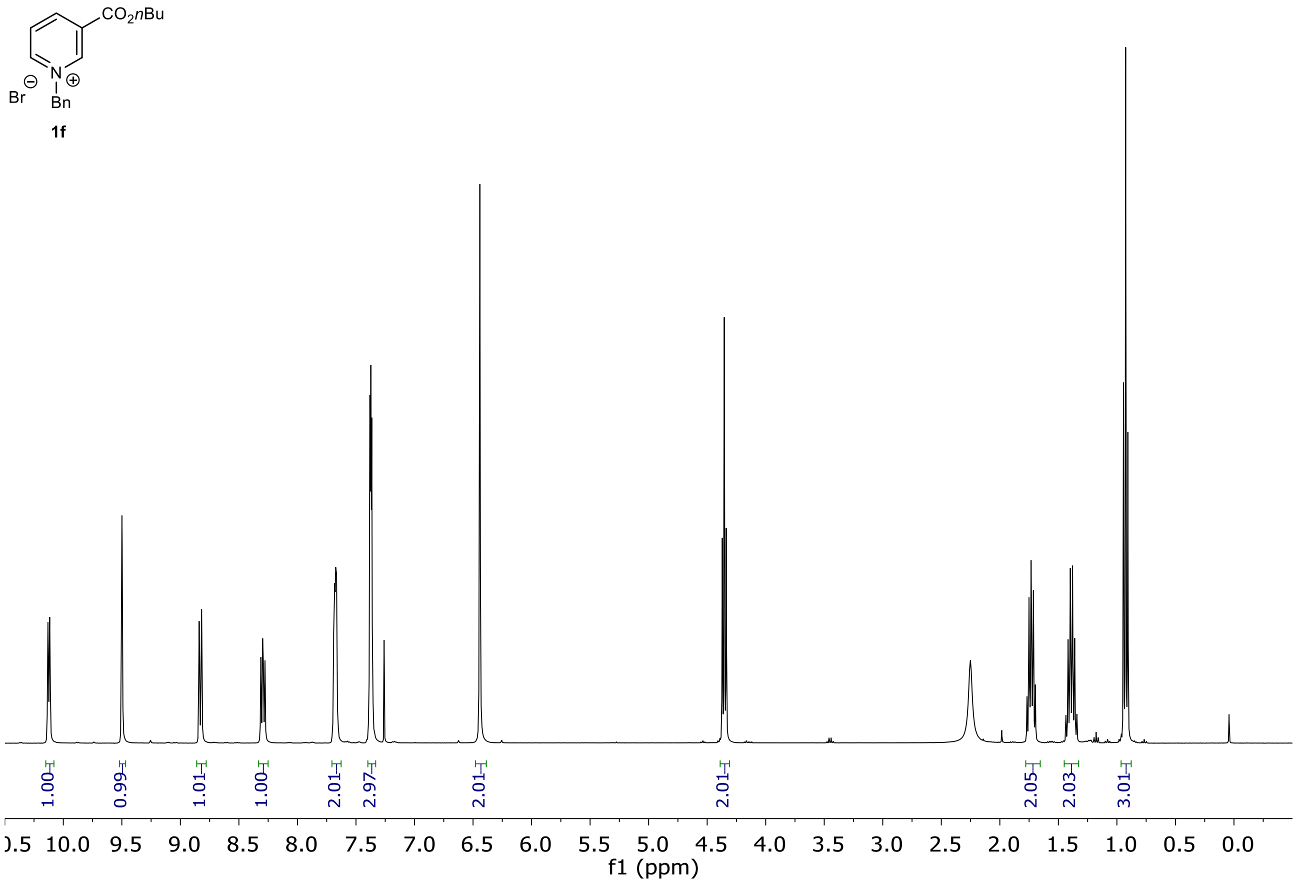
CO2nBu
Br
N
Bn
1.00
0.99
1.01
1.00
2.01
2.97
2.01
2.01
2.05
2.03
3.01
f1 (ppm)

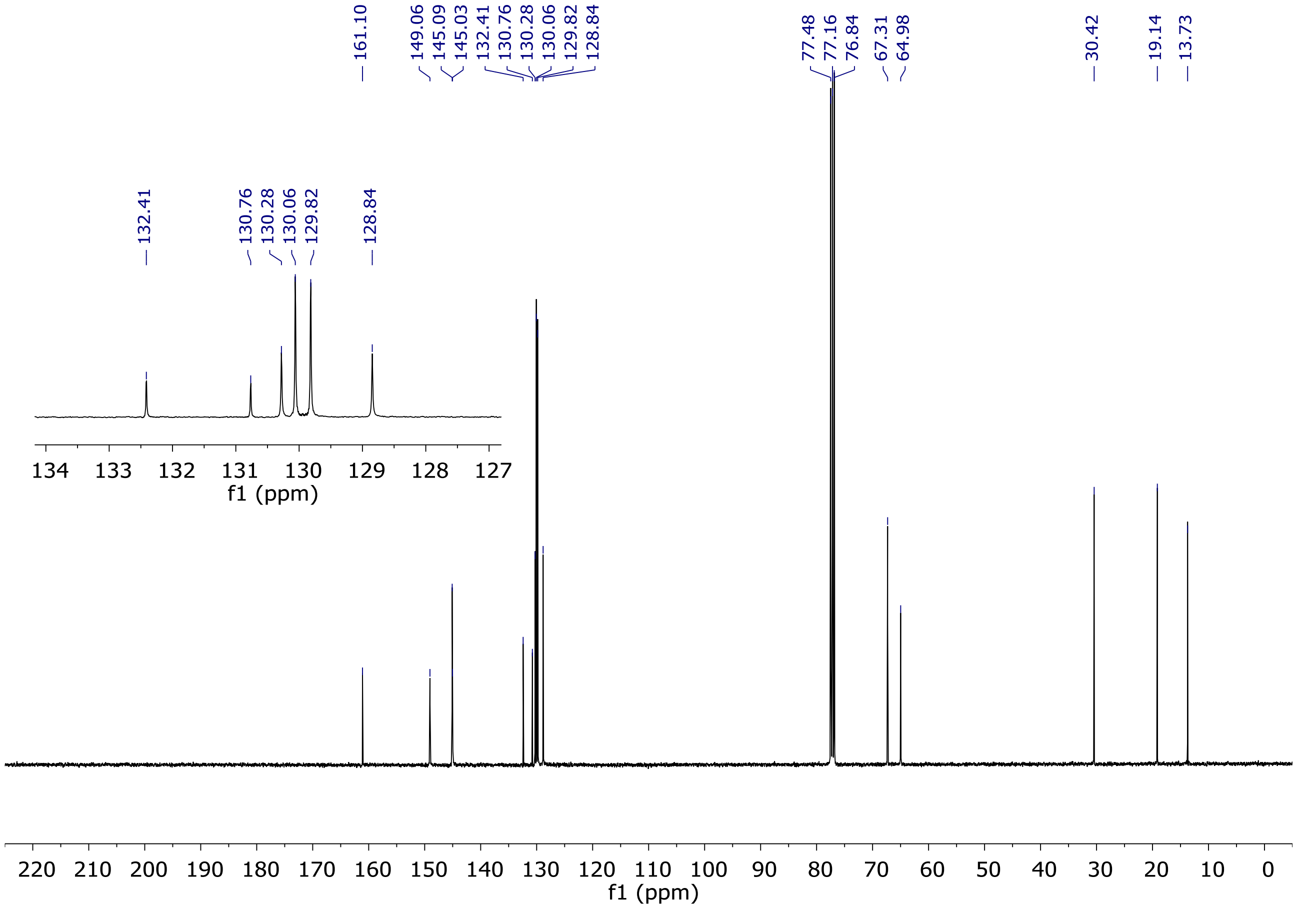
161.10
149.06
145.09
145.03
132.41
130.76
130.28
130.06
129.82
128.84
77.48
77.16
76.84
67.31
64.98
30.42
19.14
13.73
f1 (ppm)

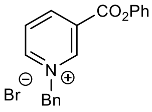
CO2Ph
N
Br
Bn

1g

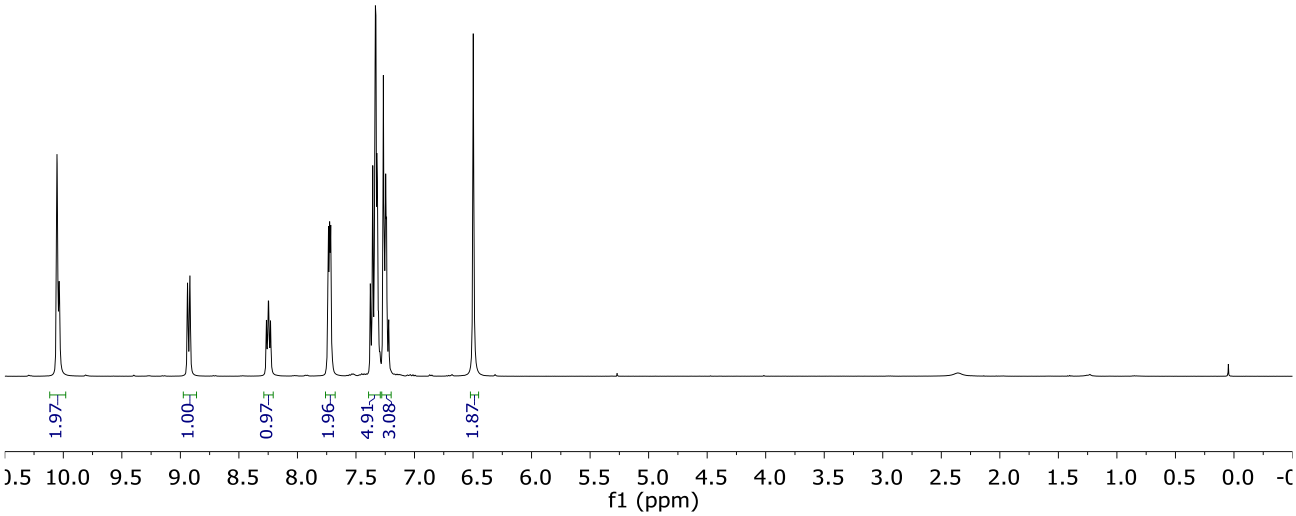
1.97
1.00
0.97
1.96
4.91
3.08
1.87
10.0 9.5 9.0 8.5 8.0 7.5 7.0 6.5 6.0 5.5 5.0 4.5 4.0 3.5 3.0 2.5 2.0 1.5 1.0 0.5 0.0
f1 (ppm)

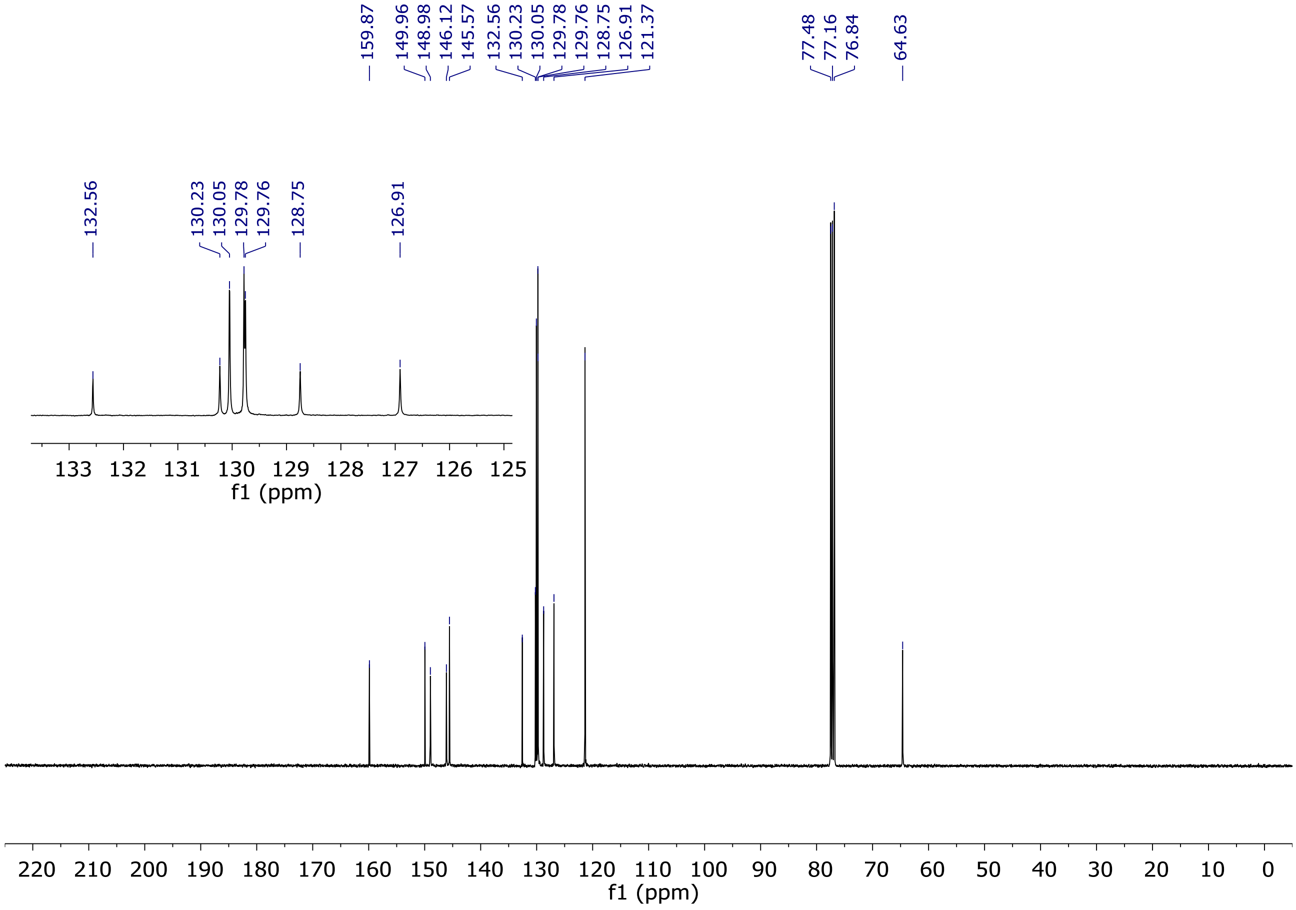
159.87
149.96
148.98
146.12
145.57
132.56
130.23
130.05
129.78
129.76
128.75
126.91
121.37
77.48
77.16
76.84
64.63
132.56
130.23
130.05
129.78
129.76
128.75
126.91
133 132 131 130 129 128 127 126 125
f1 (ppm)
220 210 200 190 180 170 160 150 140 130 120 110 100 90 80 70 60 50 40 30 20 10 0
f1 (ppm)

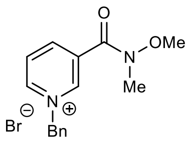
O
OMe
N
Me
N
Br
Bn

1h

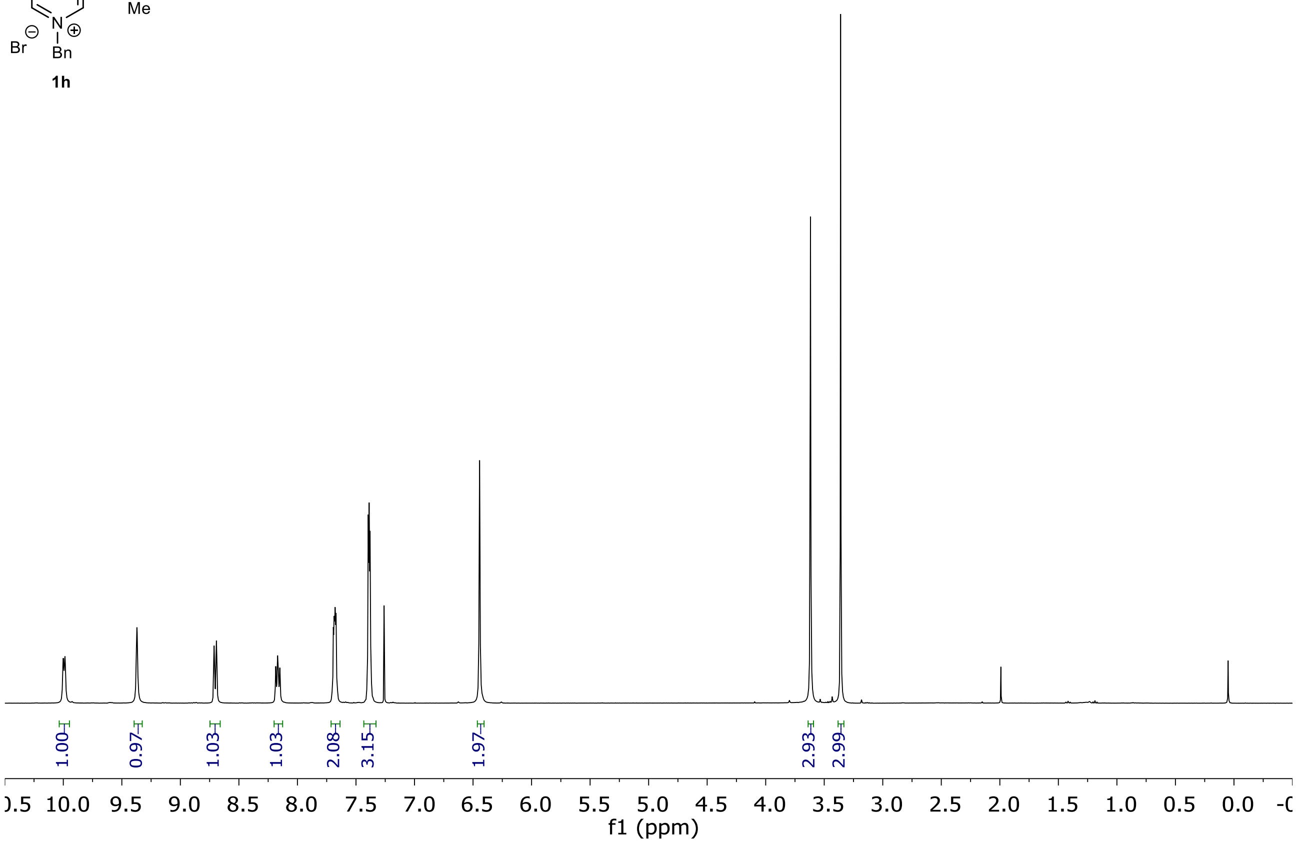
1.00
0.97
1.03
1.03
2.08
3.15
1.97
2.93
2.99
10.0 9.5 9.0 8.5 8.0 7.5 7.0 6.5 6.0 5.5 5.0 4.5 4.0 3.5 3.0 2.5 2.0 1.5 1.0 0.5 0.0
f1 (ppm)

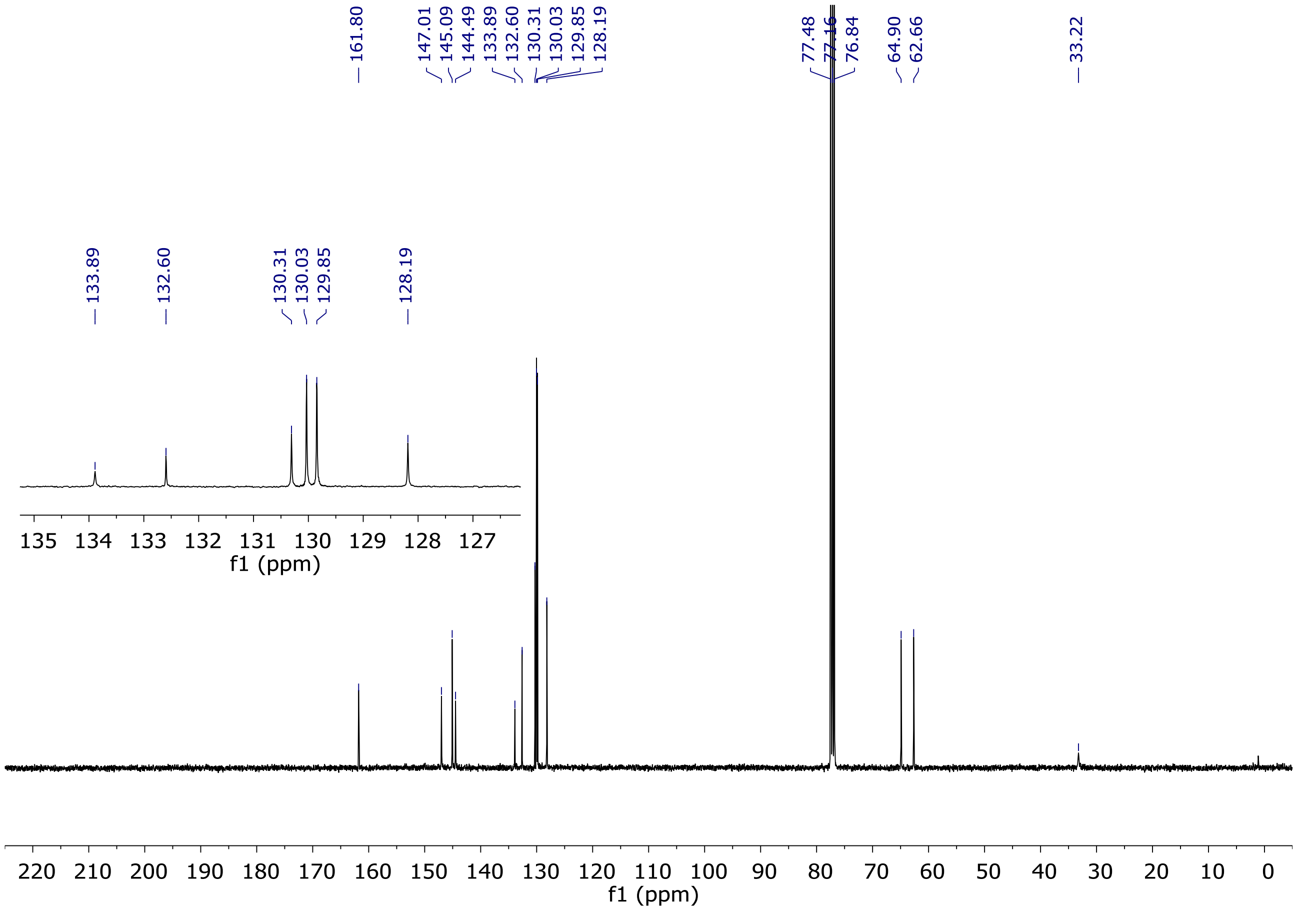
161.80
147.01
145.09
144.49
133.89
132.60
130.31
130.03
129.85
128.19
77.48
77.16
76.84
64.90
62.66
33.22
133.89
132.60
130.31
130.03
129.85
128.19
135 134 133 132 131 130 129 128 127
f1 (ppm)
220 210 200 190 180 170 160 150 140 130 120 110 100 90 80 70 60 50 40 30 20 10 0
f1 (ppm)

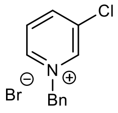
Cl
N
Br
Bn

1i

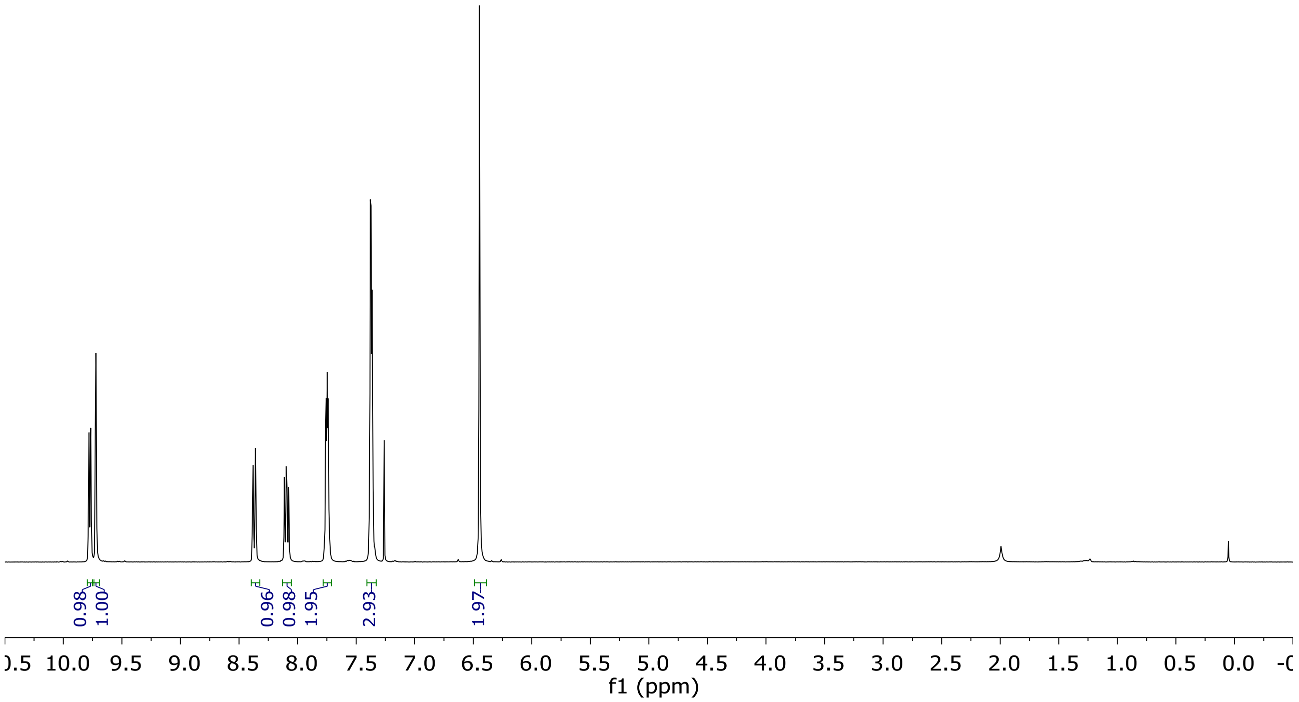
0.98
1.00
0.96
0.98
1.95
2.93
1.97
).5 10.0 9.5 9.0 8.5 8.0 7.5 7.0 6.5 6.0 5.5 5.0 4.5 4.0 3.5 3.0 2.5 2.0 1.5 1.0 0.5 0.0 -(
f1 (ppm)

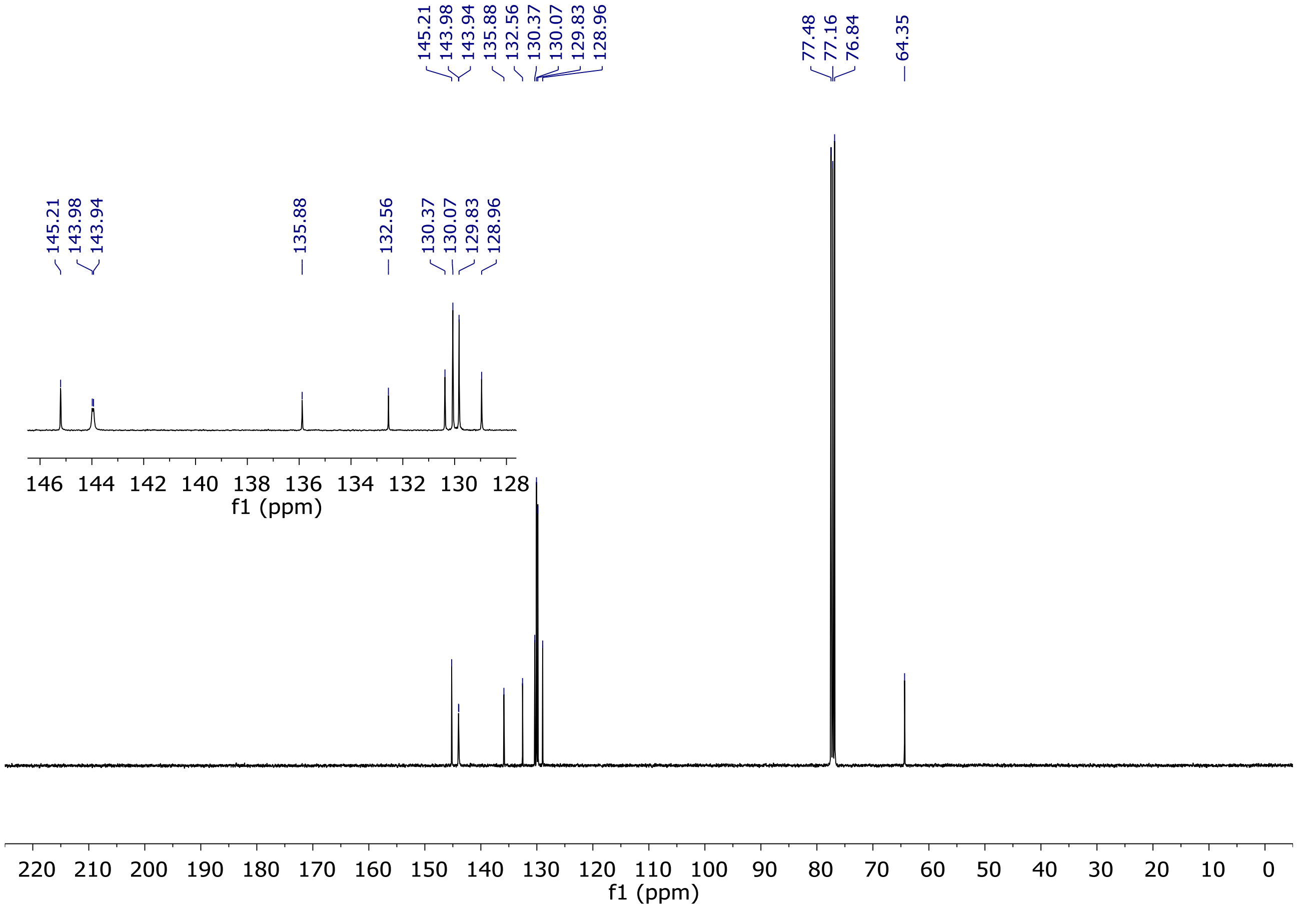
145.21
143.98
143.94
135.88
132.56
130.37
130.07
129.83
128.96
77.48
77.16
76.84
64.35
146 144 142 140 138 136 134 132 130 128
f1 (ppm)
220 210 200 190 180 170 160 150 140 130 120 110 100 90 80 70 60 50 40 30 20 10 0
f1 (ppm)

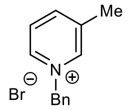
Me
N
Br
Bn

1l

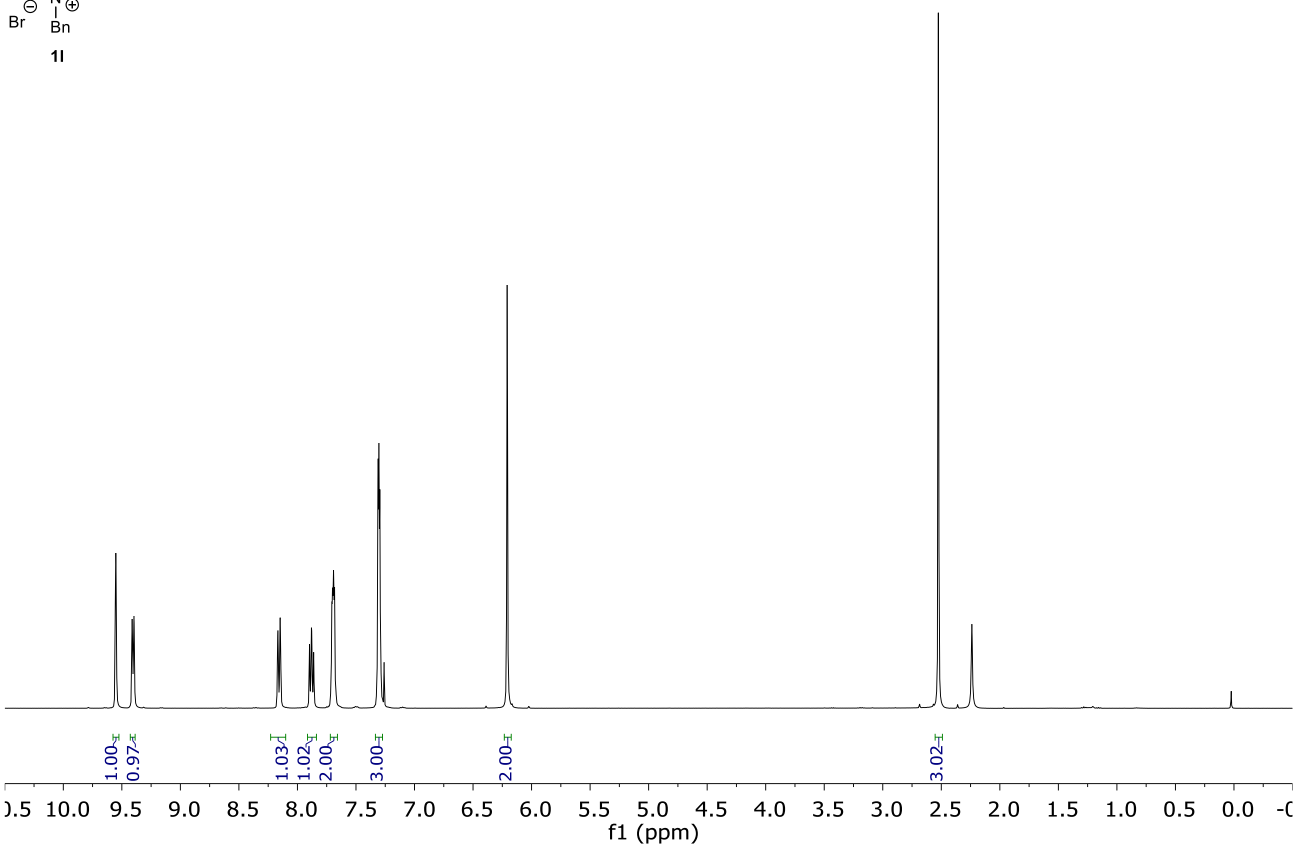
1.00
0.97
1.03
1.02
2.00
3.00
2.00
3.02
f1 (ppm)

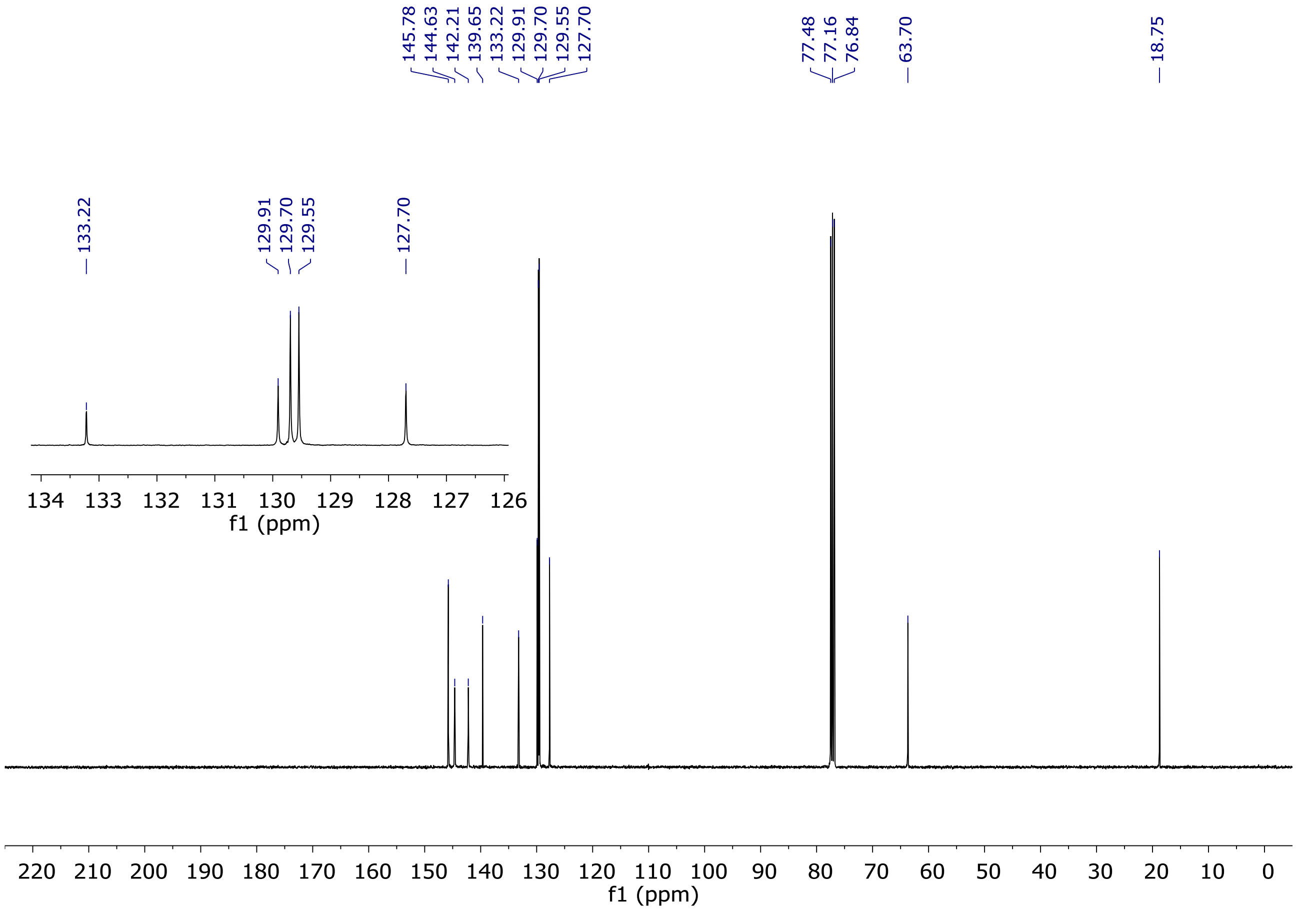
145.78
144.63
142.21
139.65
133.22
129.91
129.70
129.55
127.70
77.48
77.16
76.84
63.70
18.75
f1 (ppm)

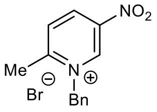
NO2
Me
Br
N
Bn

1m

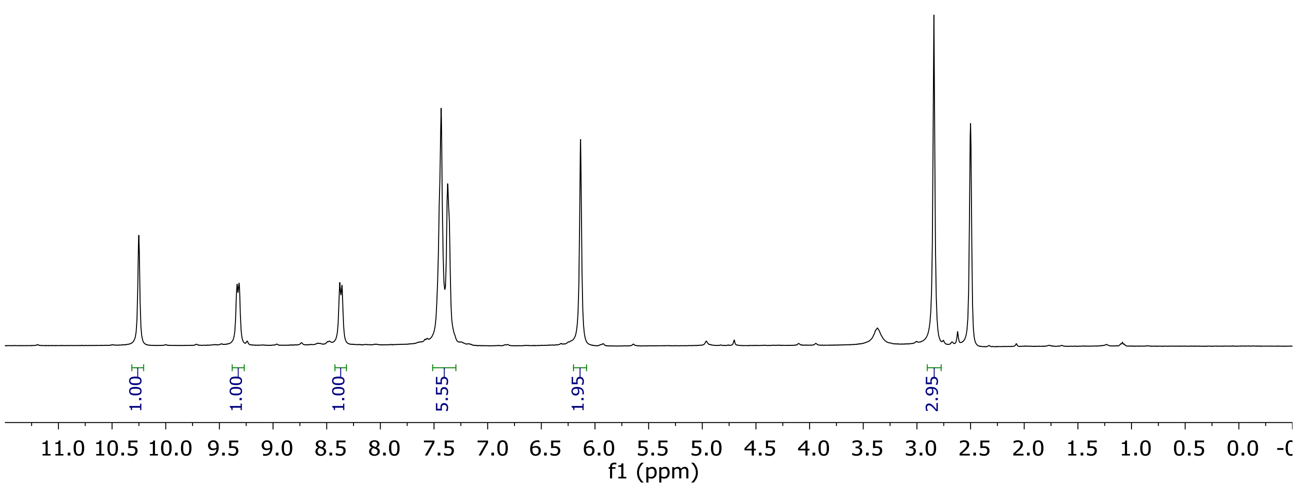
1.00
1.00
1.00
5.55
1.95
2.95
11.0 10.5 10.0 9.5 9.0 8.5 8.0 7.5 7.0 6.5 6.0 5.5 5.0 4.5 4.0 3.5 3.0 2.5 2.0 1.5 1.0 0.5 0.0
f1 (ppm)

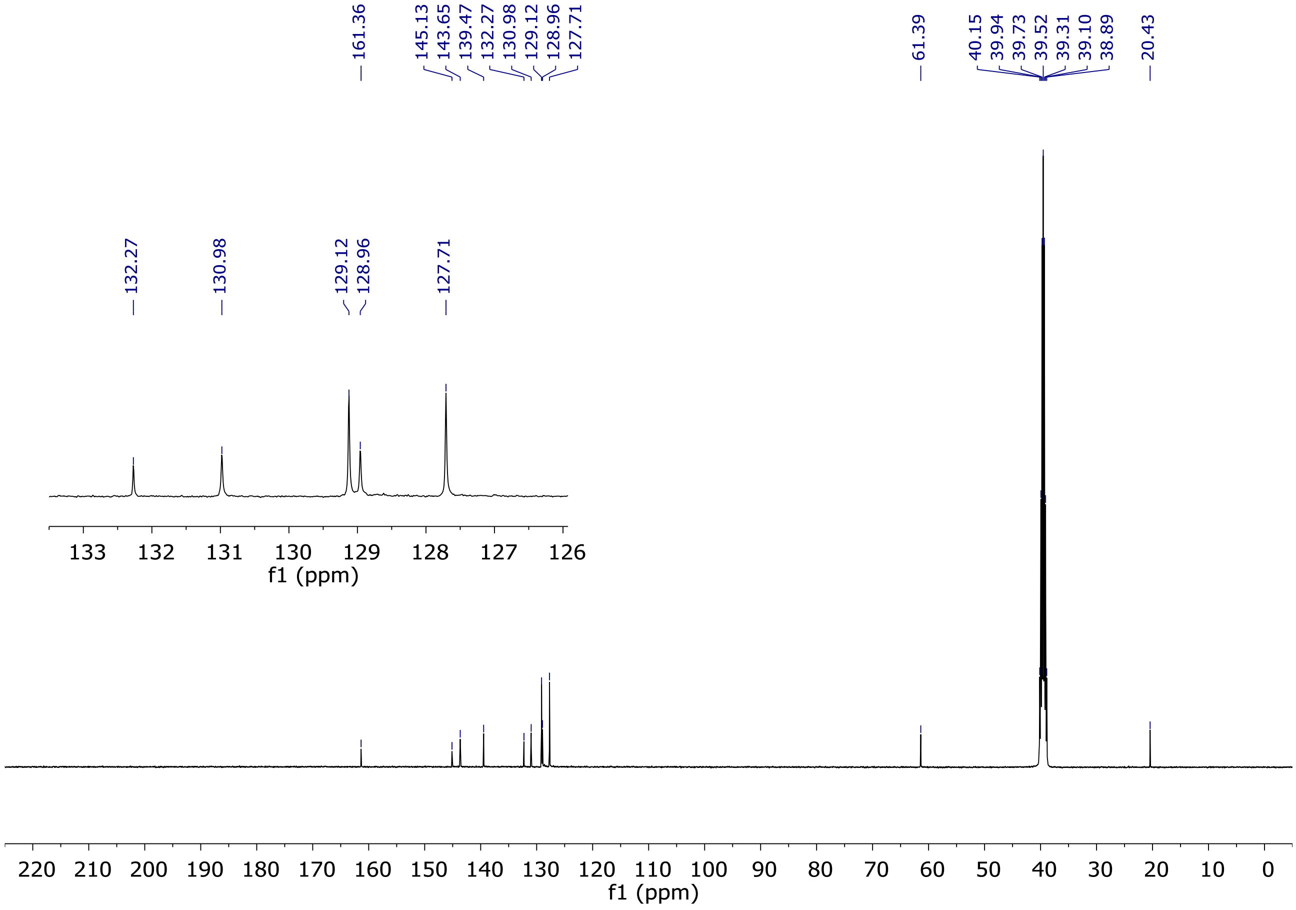
161.36
145.13
143.65
139.47
132.27
130.98
129.12
128.96
127.71
61.39
40.15
39.94
39.73
39.52
39.31
39.10
38.89
20.43
132.27
130.98
129.12
128.96
127.71
133 132 131 130 129 128 127 126
f1 (ppm)
220 210 200 190 180 170 160 150 140 130 120 110 100 90 80 70 60 50 40 30 20 10 0
f1 (ppm)

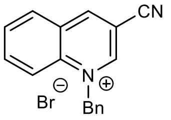
CN
Br
N
Bn

1n

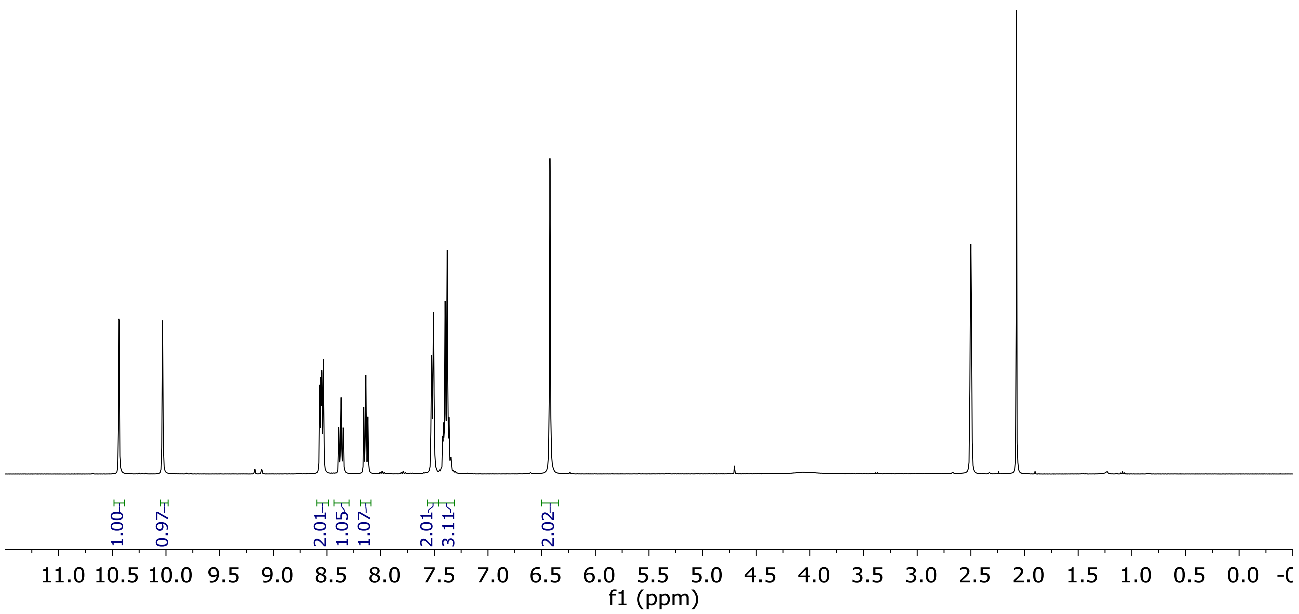
1.00
0.97
2.01
1.05
1.07
2.01
3.11
2.02
f1 (ppm)

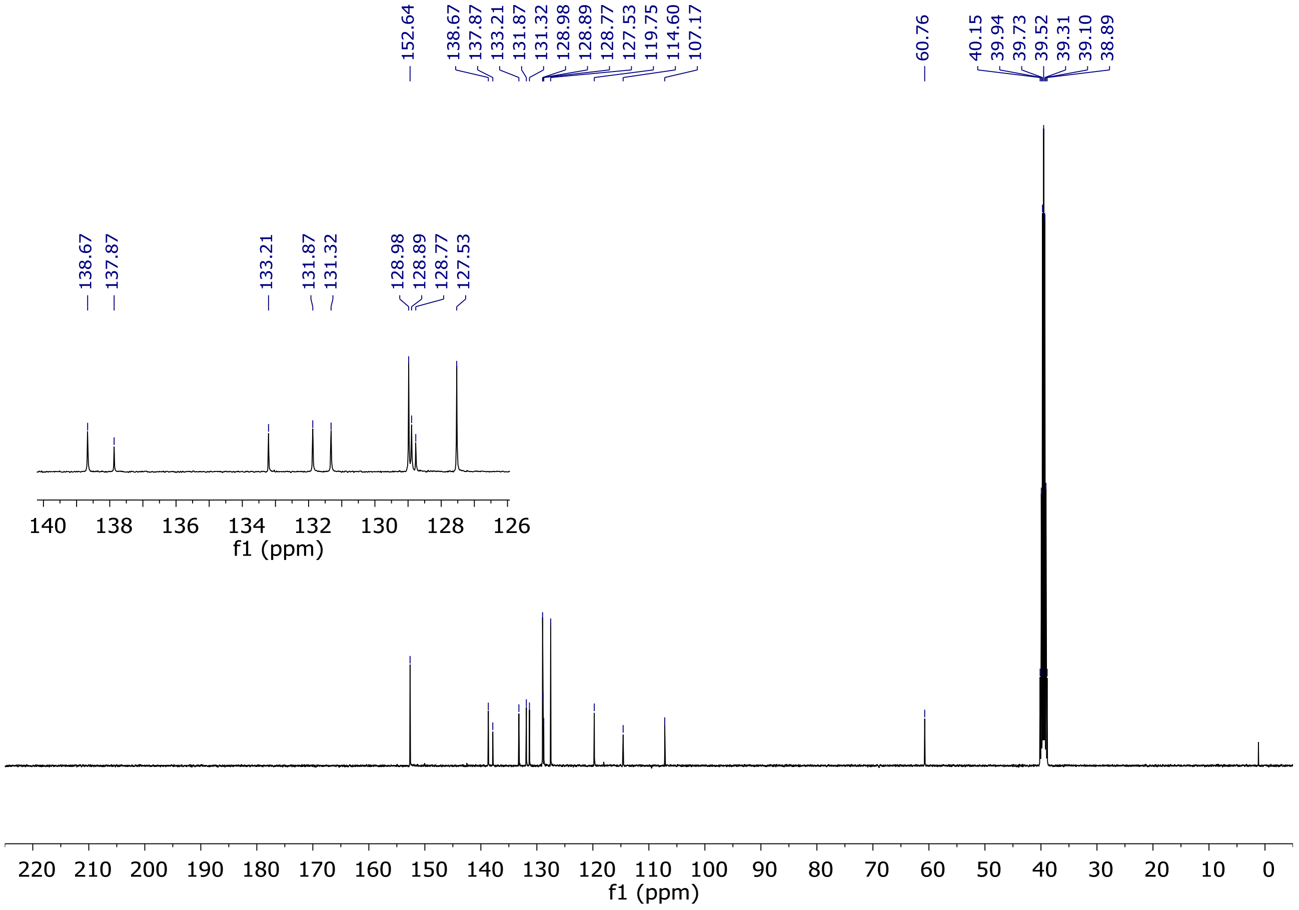
152.64
138.67
137.87
133.21
131.87
131.32
128.98
128.89
128.77
127.53
119.75
114.60
107.17
60.76
40.15
39.94
39.73
39.52
39.31
39.10
38.89
f1 (ppm)

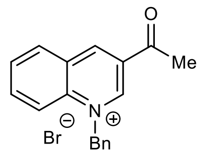
O
Me
Br
N
Bn

1o

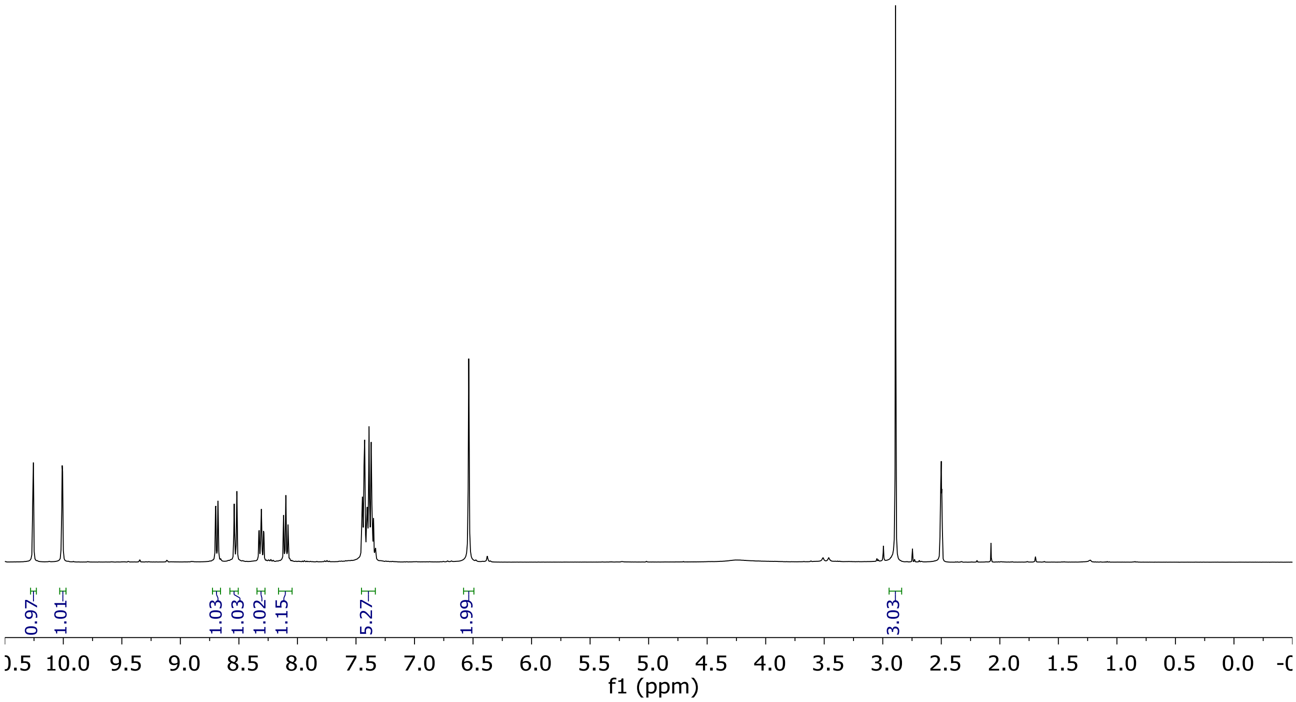
0.97
1.01
1.03
1.03
1.02
1.15
5.27
1.99
3.03
10.0 9.5 9.0 8.5 8.0 7.5 7.0 6.5 6.0 5.5 5.0 4.5 4.0 3.5 3.0 2.5 2.0 1.5 1.0 0.5 0.0
f1 (ppm)

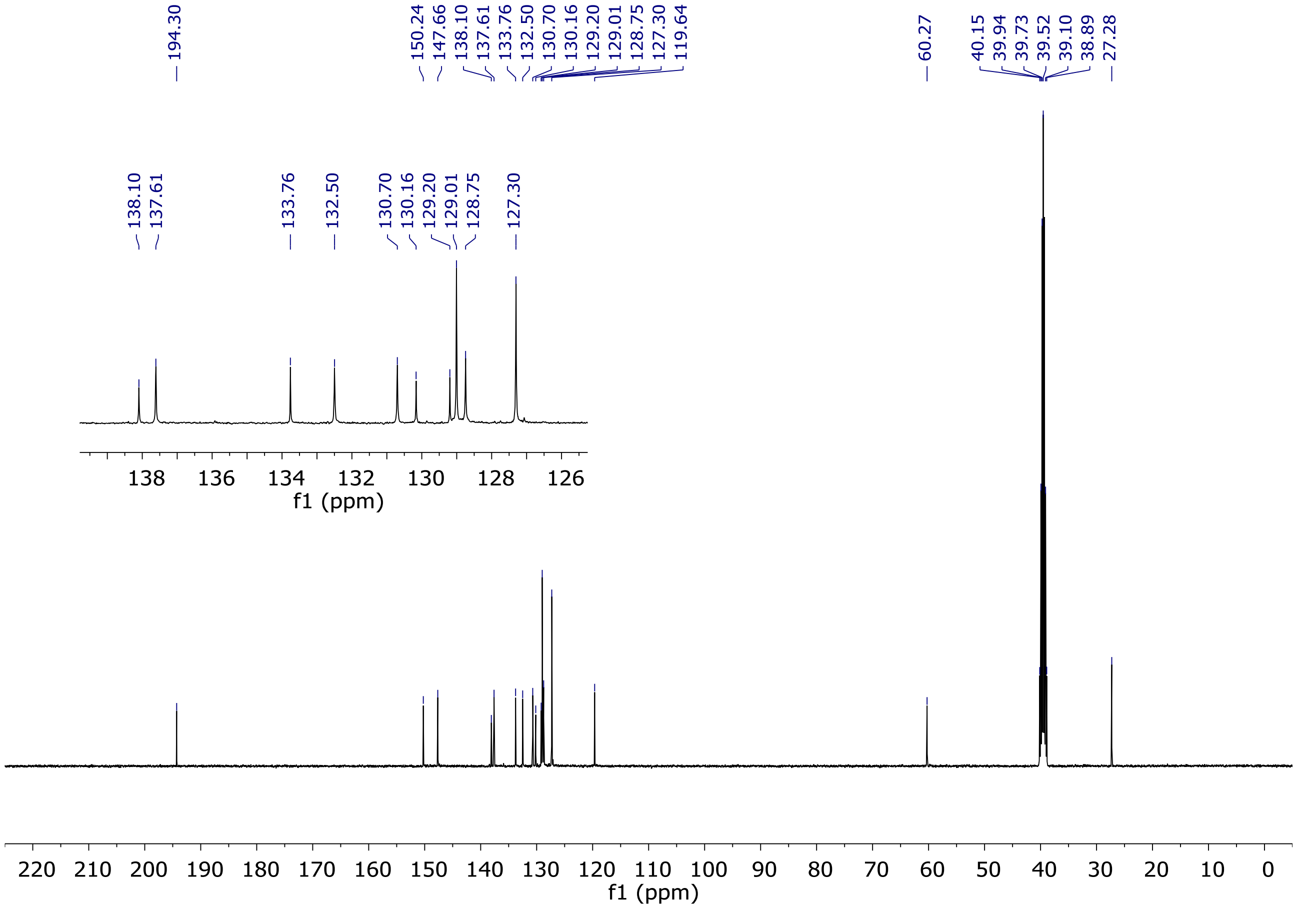
194.30
150.24
147.66
138.10
137.61
133.76
132.50
130.70
130.16
129.20
129.01
128.75
127.30
119.64
60.27
40.15
39.94
39.73
39.52
39.10
38.89
27.28
138 136 134 132 130 128 126
f1 (ppm)
220 210 200 190 180 170 160 150 140 130 120 110 100 90 80 70 60 50 40 30 20 10 0
f1 (ppm)

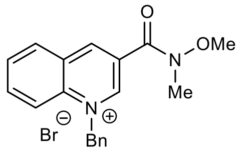
O
N
OMe
Me
Br
N
Bn

1q

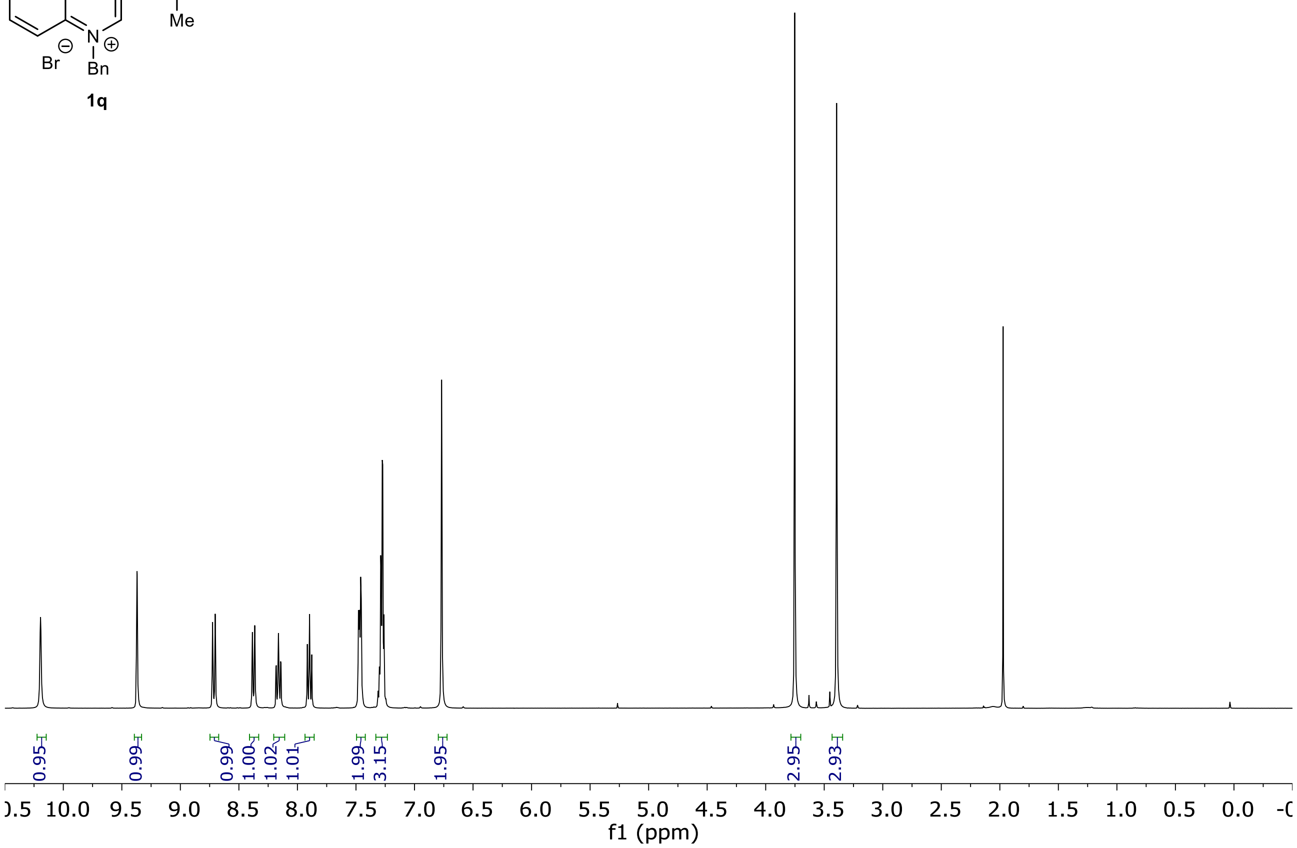
0.95
0.99
0.99
1.00
1.02
1.01
1.99
3.15
1.95
2.95
2.93
10.0 9.5 9.0 8.5 8.0 7.5 7.0 6.5 6.0 5.5 5.0 4.5 4.0 3.5 3.0 2.5 2.0 1.5 1.0 0.5 0.0
f1 (ppm)

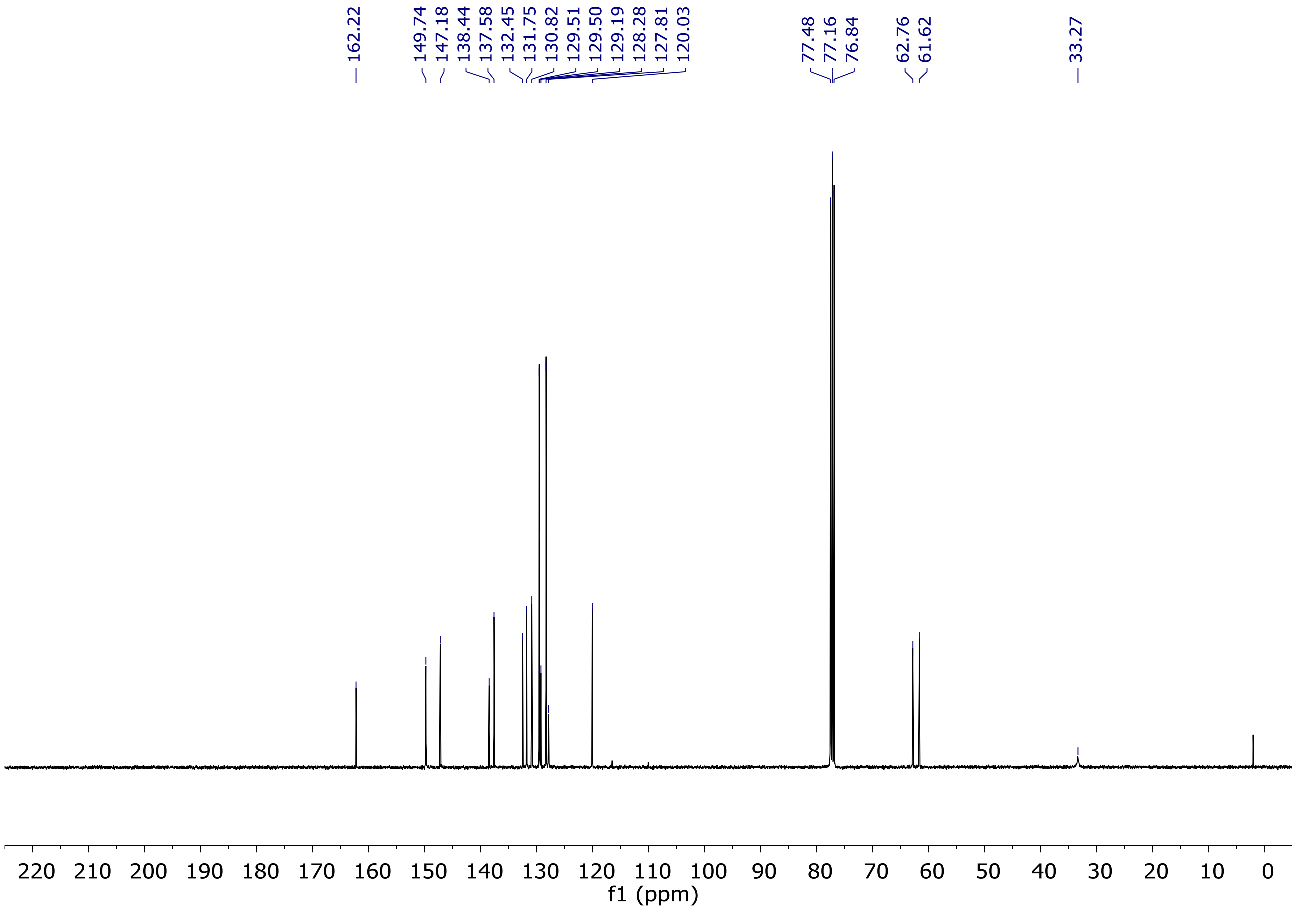
162.22
149.74
147.18
138.44
137.58
132.45
131.75
130.82
129.51
129.50
129.19
128.28
127.81
120.03
77.48
77.16
76.84
62.76
61.62
33.27
220 210 200 190 180 170 160 150 140 130 120 110 100 90 80 70 60 50 40 30 20 10 0
f1 (ppm)

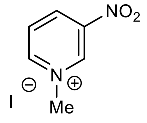
NO2
N
I
Me

1r

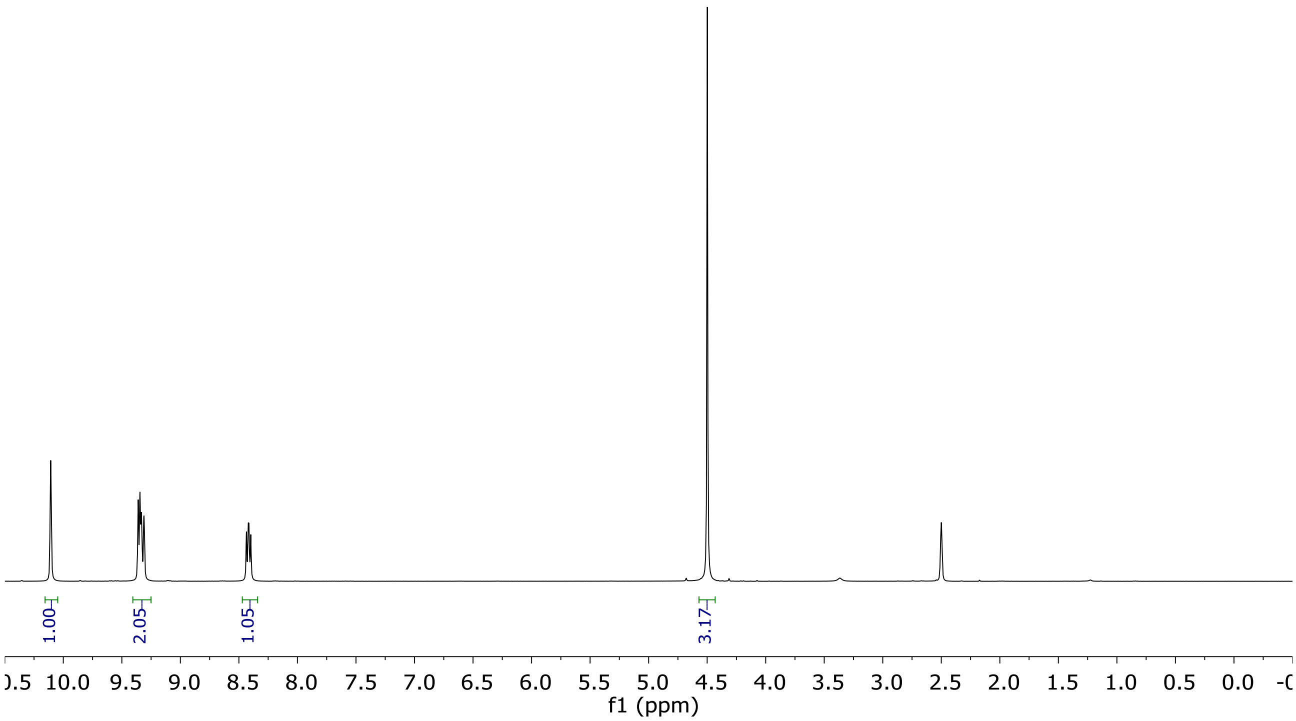
1.00
2.05
1.05
3.17
f1 (ppm)

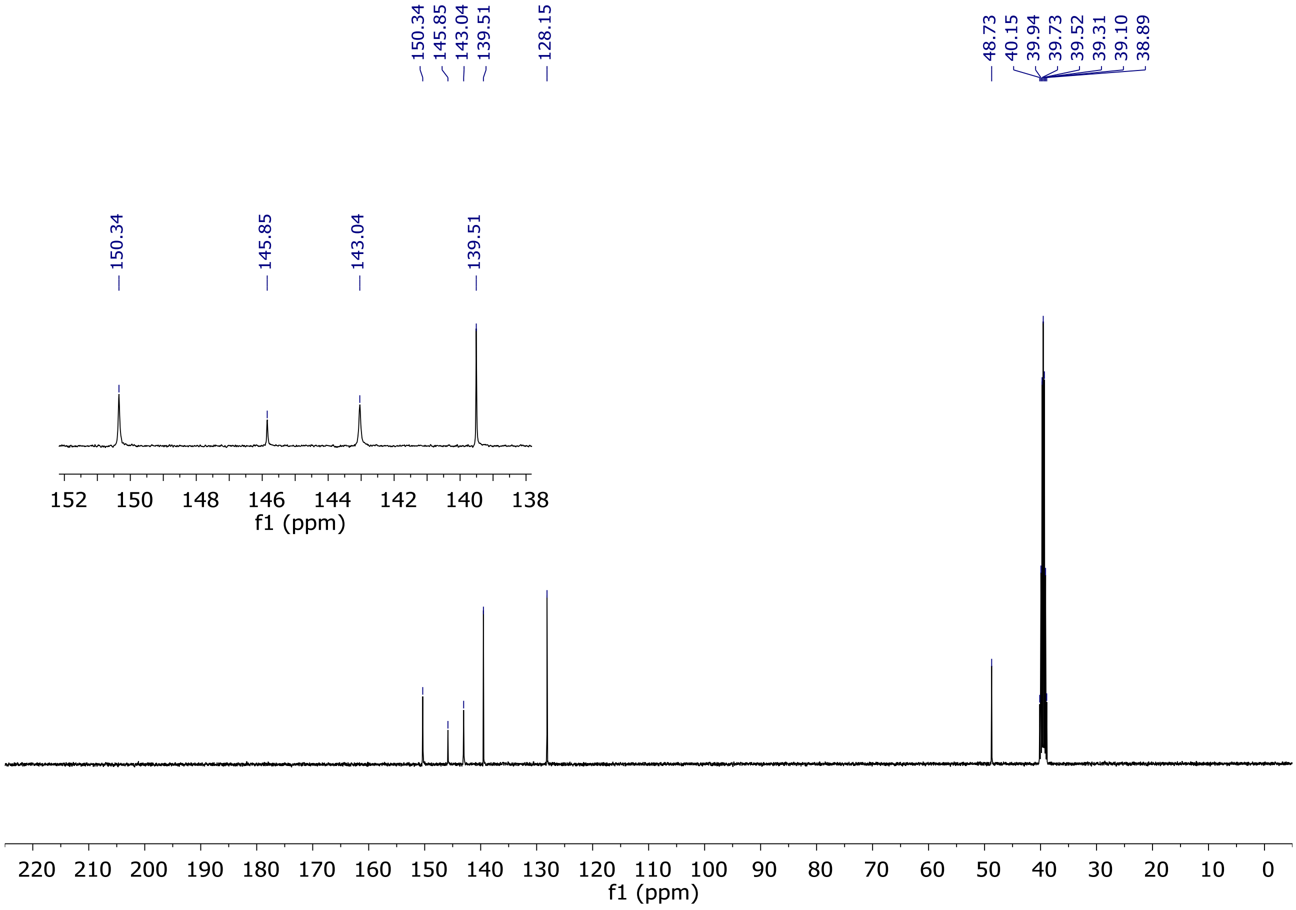
150.34
145.85
143.04
139.51
128.15
48.73
40.15
39.94
39.73
39.52
39.31
39.10
38.89
f1 (ppm)

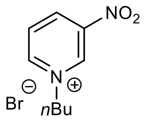
NO2
N
Br
nBu

1sBr

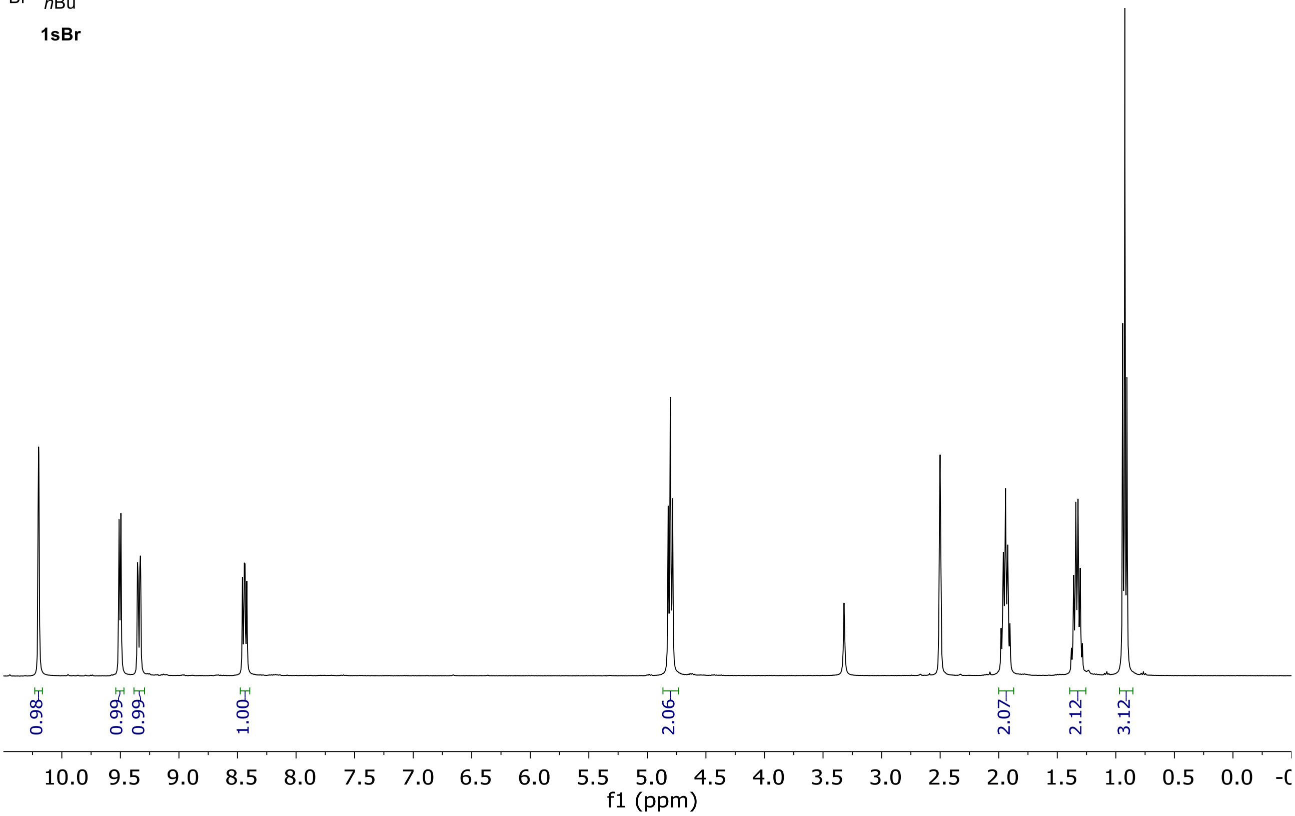
0.98
0.99
0.99
1.00
2.06
2.07
2.12
3.12
10.0 9.5 9.0 8.5 8.0 7.5 7.0 6.5 6.0 5.5 5.0 4.5 4.0 3.5 3.0 2.5 2.0 1.5 1.0 0.5 0.0
f1 (ppm)

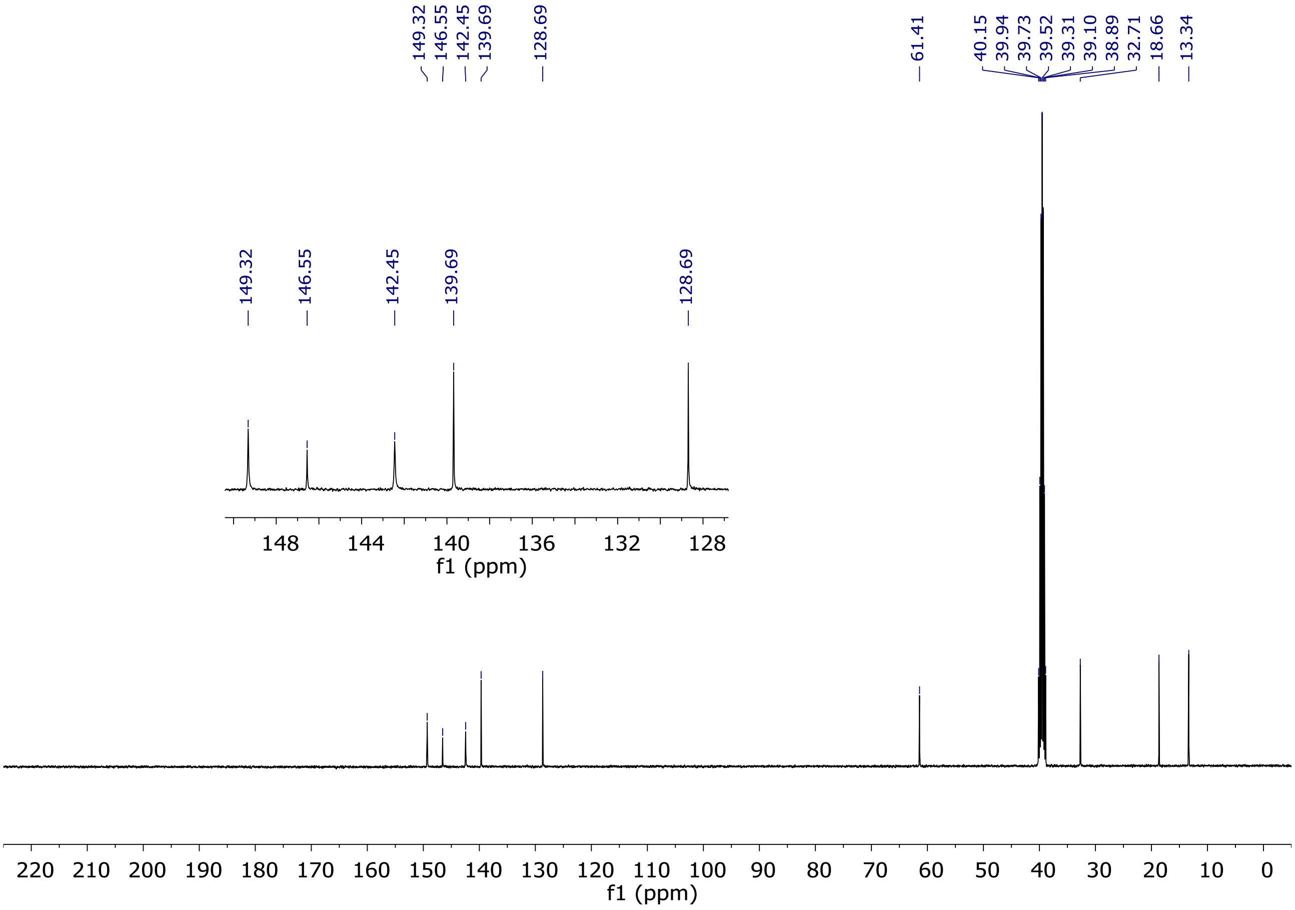
149.32
146.55
142.45
139.69
128.69
61.41
40.15
39.94
39.73
39.52
39.31
39.10
38.89
32.71
18.66
13.34
148 144 140 136 132 128
f1 (ppm)
220 210 200 190 180 170 160 150 140 130 120 110 100 90 80 70 60 50 40 30 20 10 0
f1 (ppm)

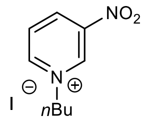
NO2
N
I
nBu

1sl

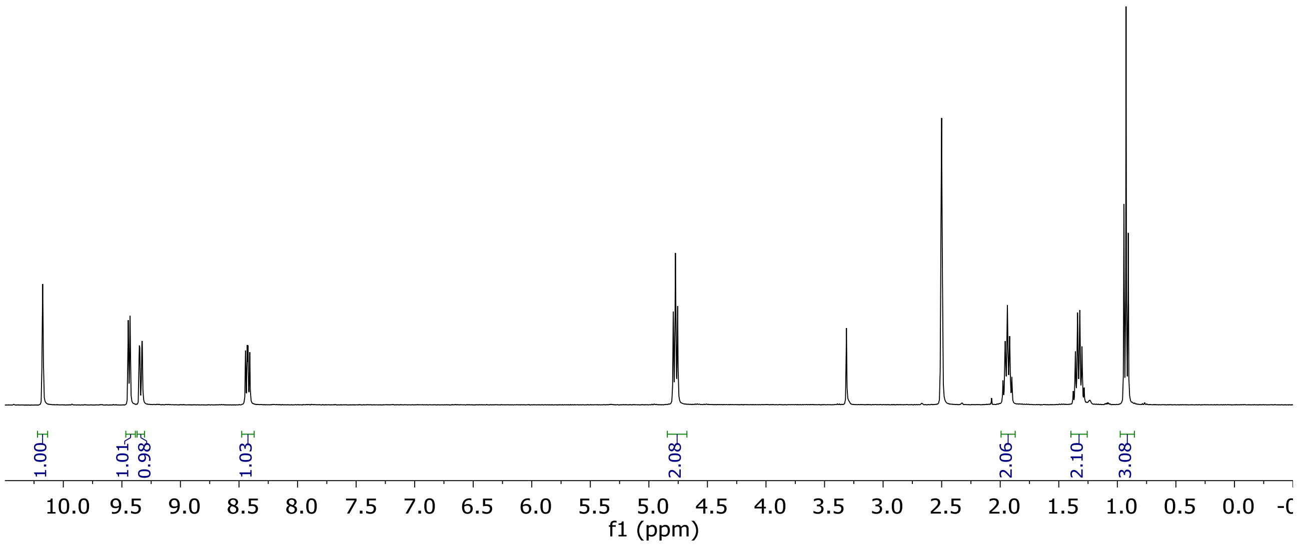
1.00
1.01
0.98
1.03
2.08
2.06
2.10
3.08
10.0 9.5 9.0 8.5 8.0 7.5 7.0 6.5 6.0 5.5 5.0 4.5 4.0 3.5 3.0 2.5 2.0 1.5 1.0 0.5 0.0
f1 (ppm)

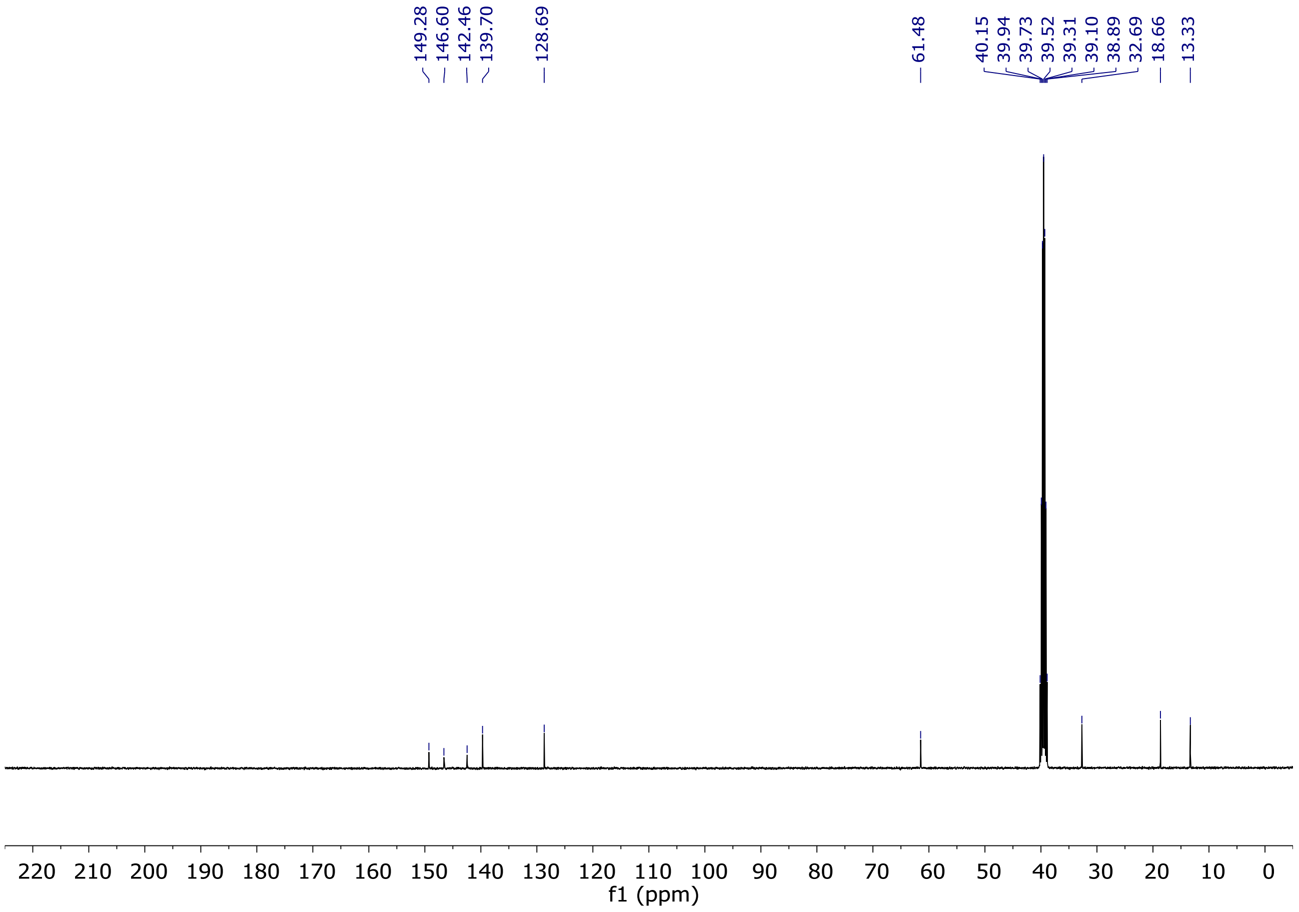
149.28
146.60
142.46
139.70
128.69
61.48
40.15
39.94
39.73
39.52
39.31
39.10
38.89
32.69
18.66
13.33
220 210 200 190 180 170 160 150 140 130 120 110 100 90 80 70 60 50 40 30 20 10 0
f1 (ppm)

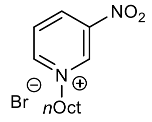
NO2
N
Br
nOct

1t

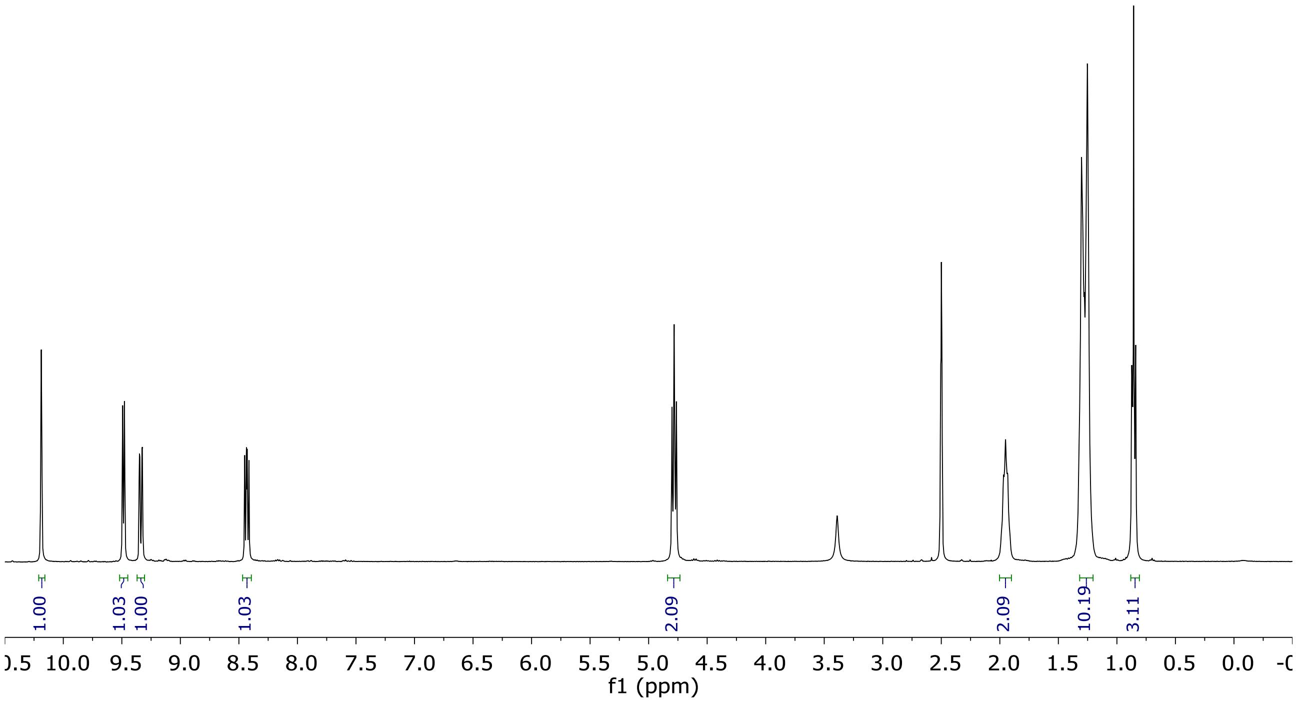
1.00
1.03
1.00
1.03
2.09
2.09
10.19
3.11
f1 (ppm)

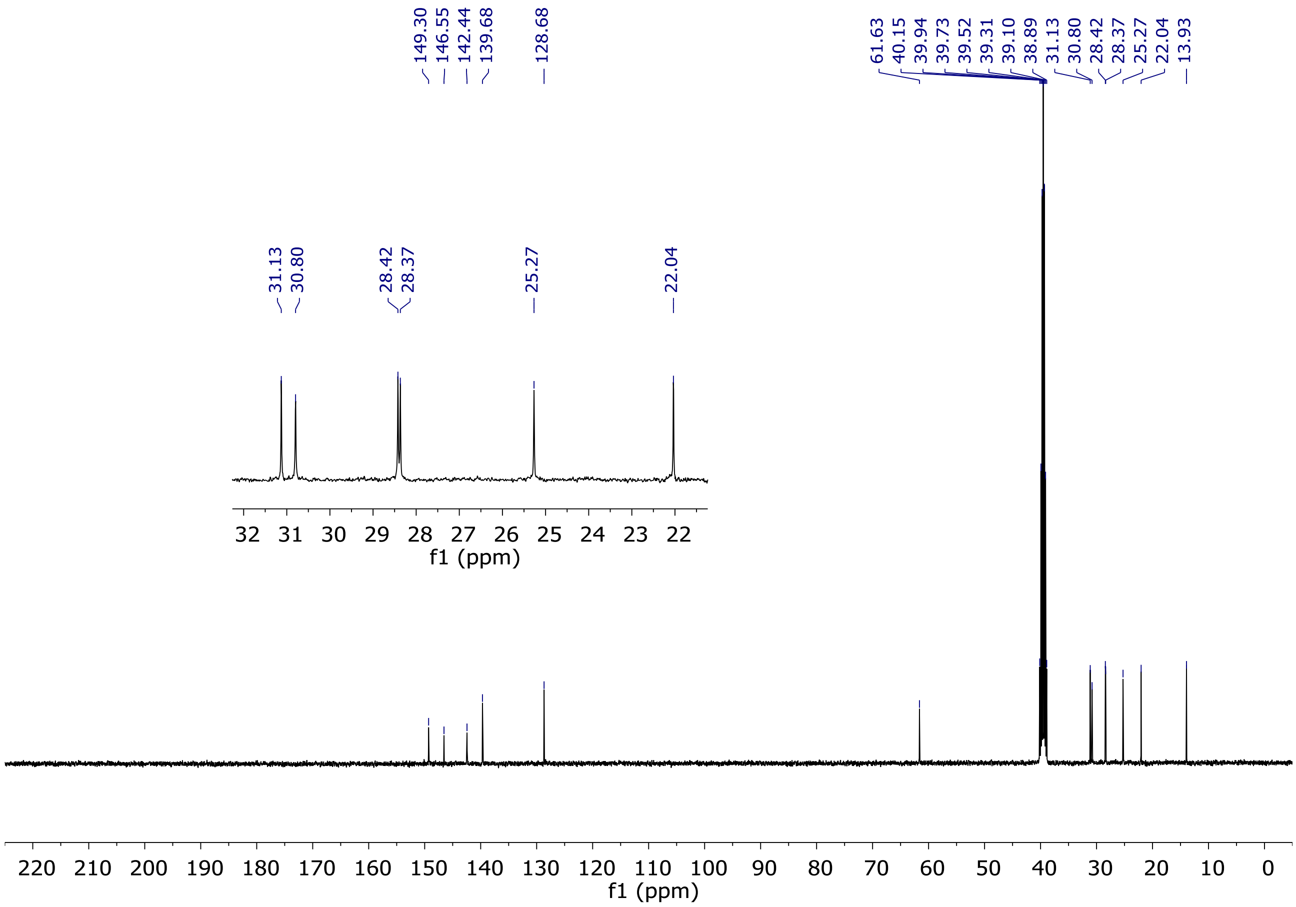
149.30
146.55
142.44
139.68
128.68
61.63
40.15
39.94
39.73
39.52
39.31
39.10
38.89
31.13
30.80
28.42
28.37
25.27
22.04
13.93
f1 (ppm)

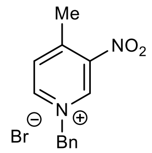
Me
NO2
N
Br
Bn

1v

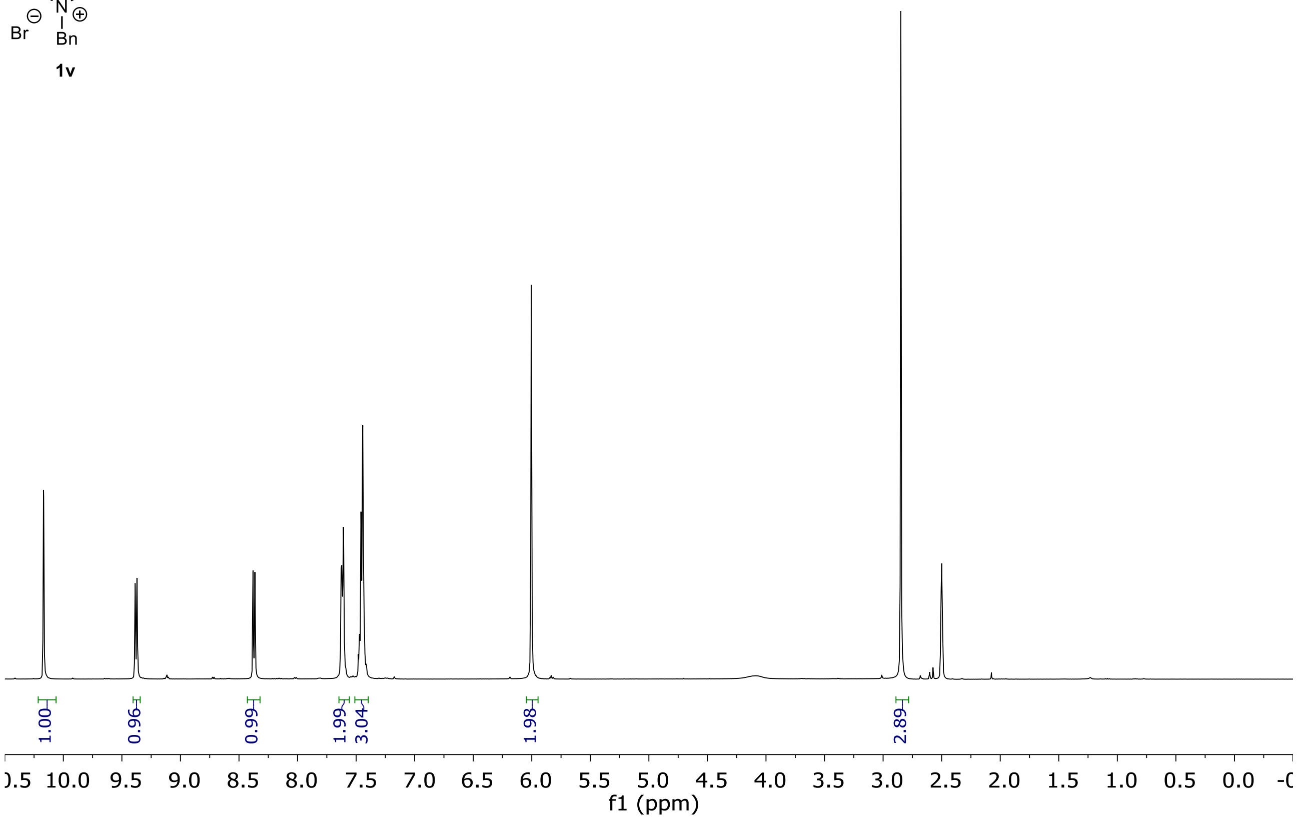
1.00
0.96
0.99
1.99
3.04
1.98
2.89
10.0 9.5 9.0 8.5 8.0 7.5 7.0 6.5 6.0 5.5 5.0 4.5 4.0 3.5 3.0 2.5 2.0 1.5 1.0 0.5 0.0
f1 (ppm)

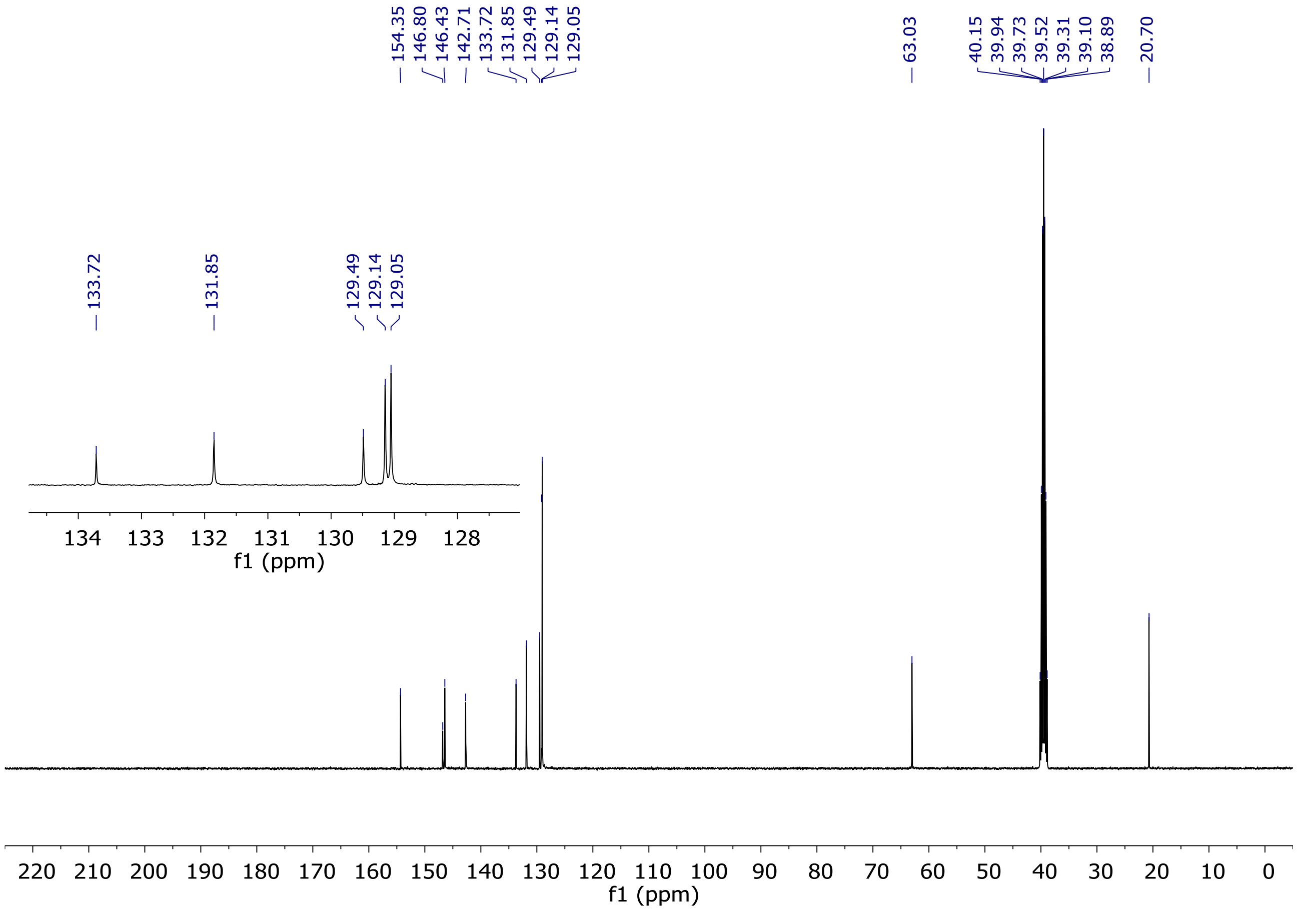
154.35
146.80
146.43
142.71
133.72
131.85
129.49
129.14
129.05
63.03
40.15
39.94
39.73
39.52
39.31
39.10
38.89
20.70
133.72
131.85
129.49
129.14
129.05
134 133 132 131 130 129 128
f1 (ppm)
220 210 200 190 180 170 160 150 140 130 120 110 100 90 80 70 60 50 40 30 20 10 0
f1 (ppm)

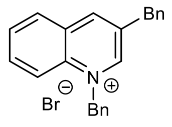
Bn
N
Br
Bn

1w

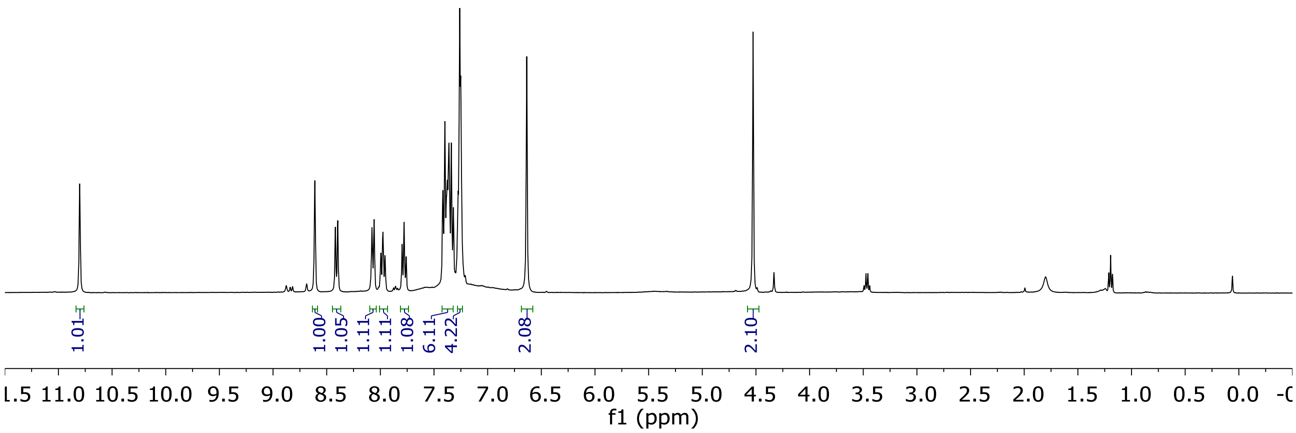
1.01
1.00
1.05
1.11
1.11
1.08
6.11
4.22
2.08
2.10
f1 (ppm)

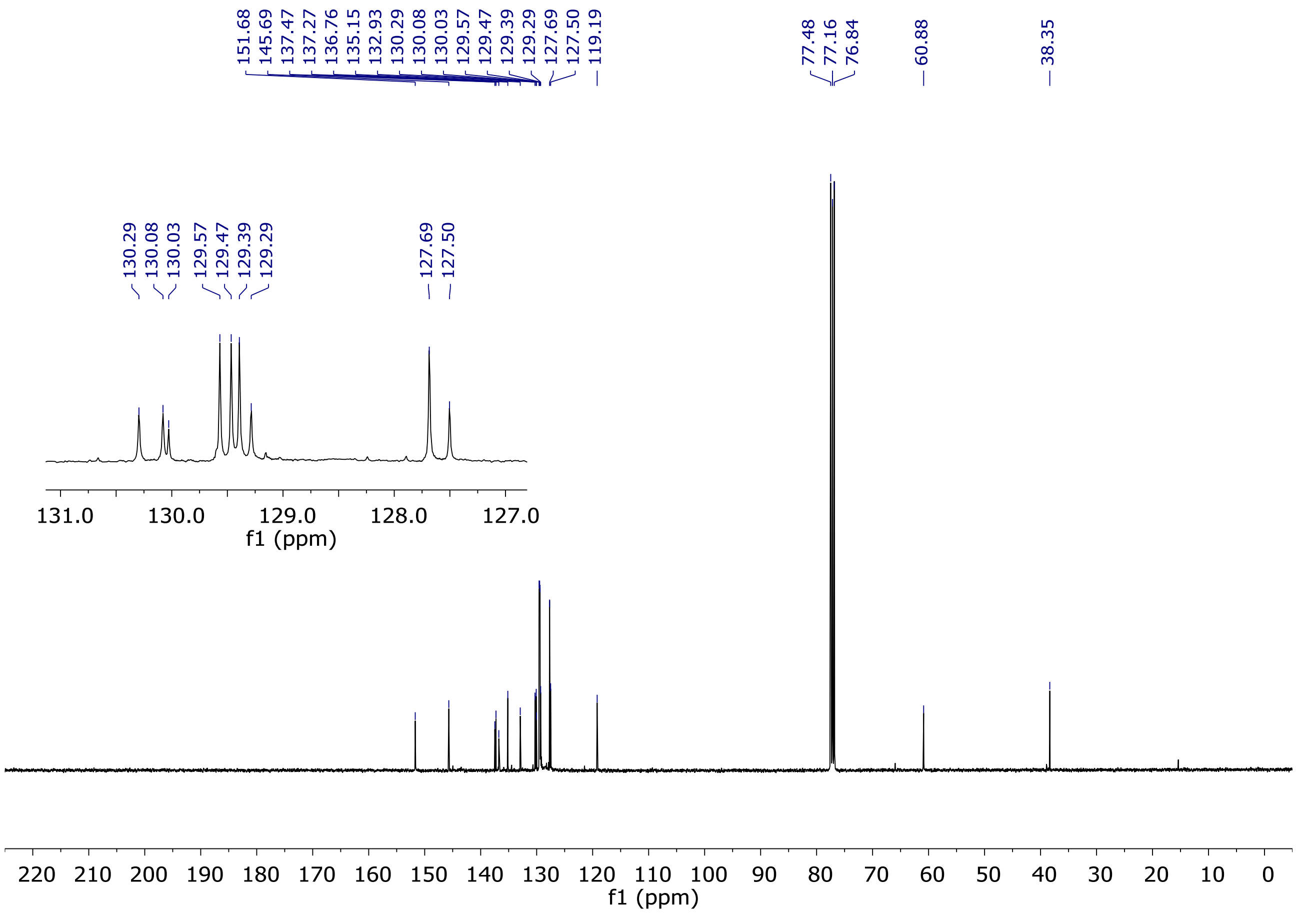
151.68
145.69
137.47
137.27
136.76
135.15
132.93
130.29
130.08
130.03
129.57
129.47
129.39
129.29
127.69
127.50
119.19
77.48
77.16
76.84
60.88
38.35
f1 (ppm)

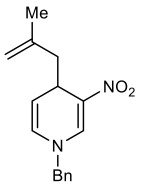
Me
NO2
N
Bn

3aa

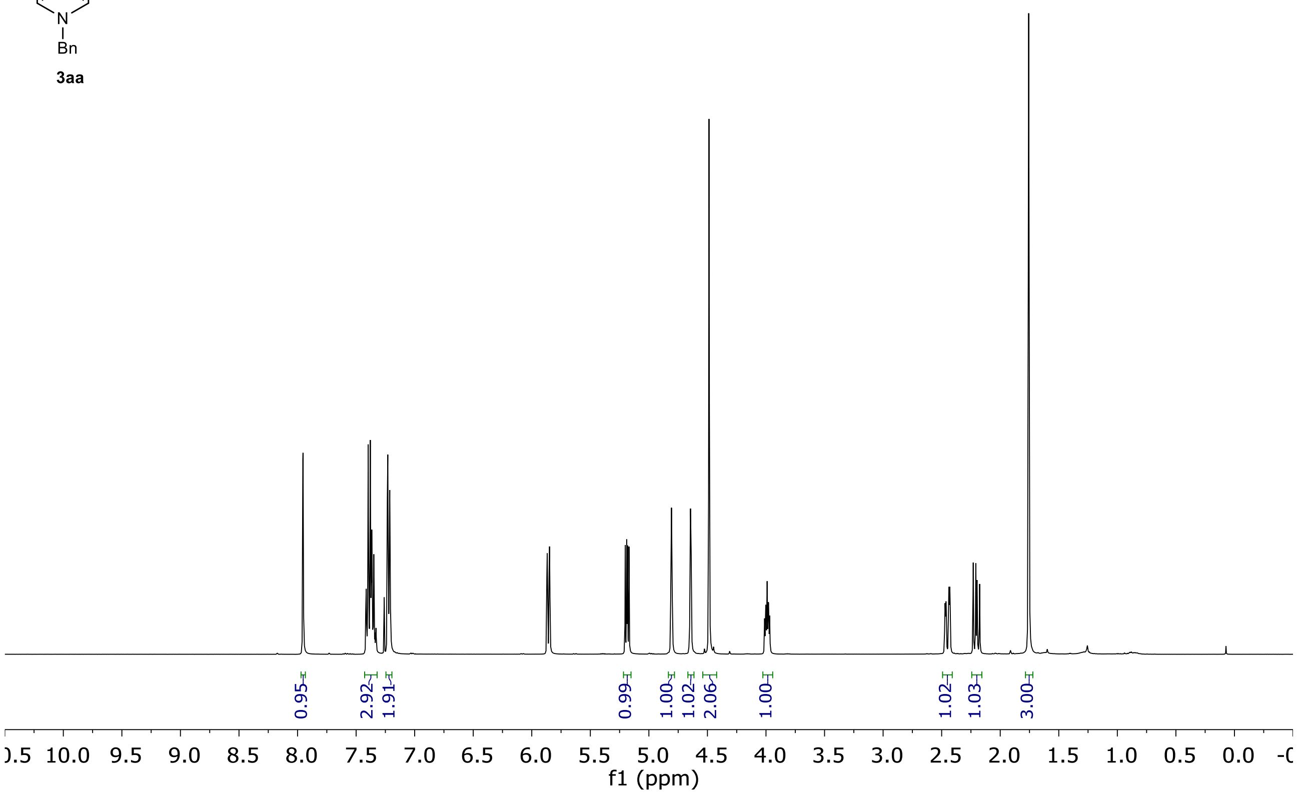
0.95
2.92
1.91
0.99
1.00
1.02
2.06
1.00
1.02
1.03
3.00
f1 (ppm)

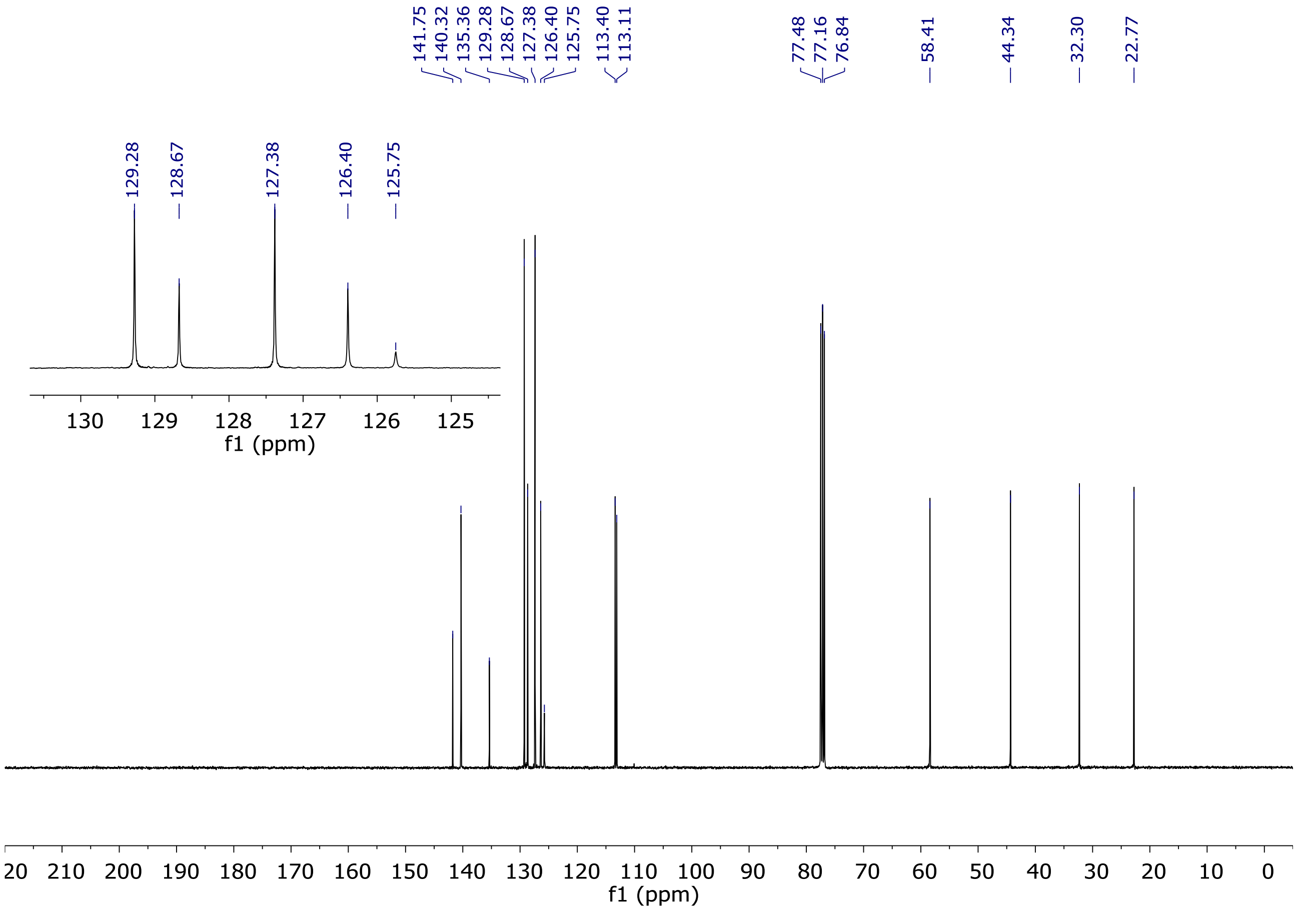
141.75
140.32
135.36
129.28
128.67
127.38
126.40
125.75
113.40
113.11
77.48
77.16
76.84
58.41
44.34
32.30
22.77
129.28
128.67
127.38
126.40
125.75
f1 (ppm)
f1 (ppm)

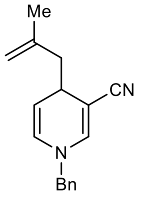
Me
CN
N
Bn

3ba

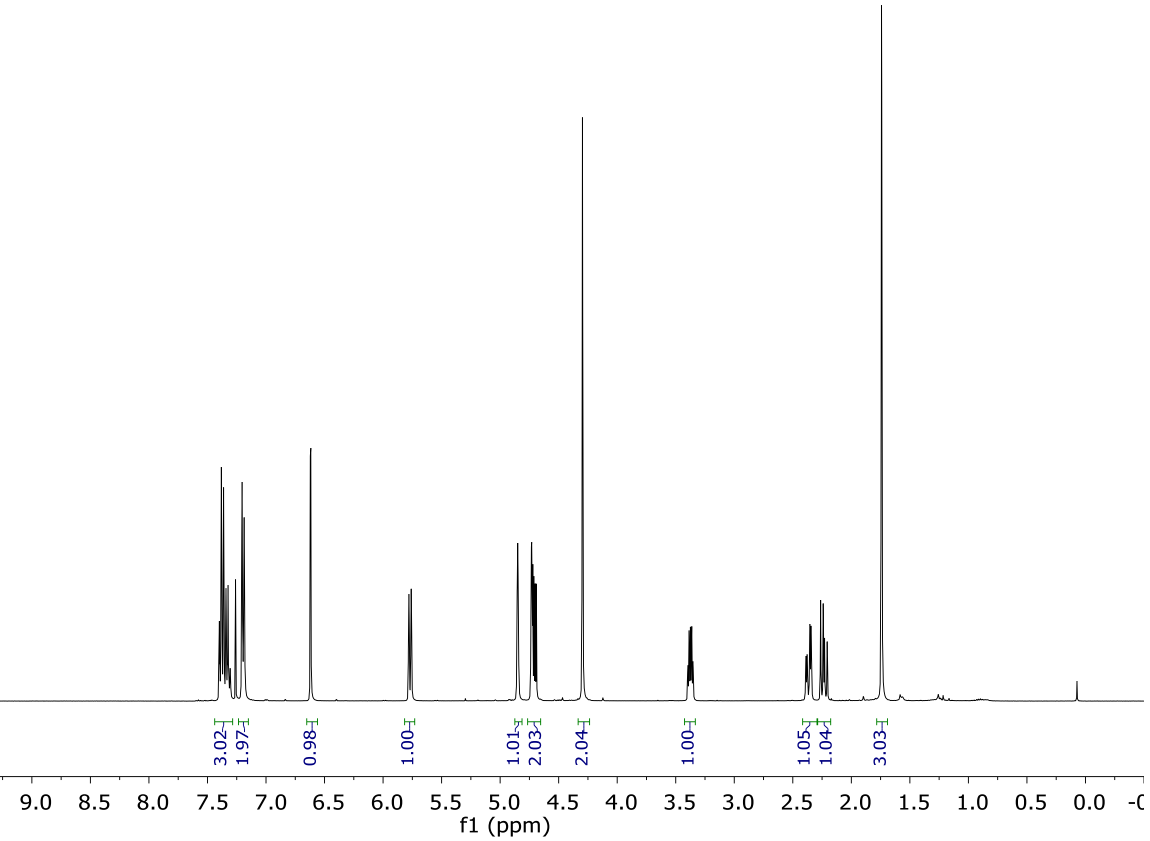
3.02
1.97
0.98
1.00
1.01
2.03
2.04
1.00
1.05
1.04
3.03
f1 (ppm)

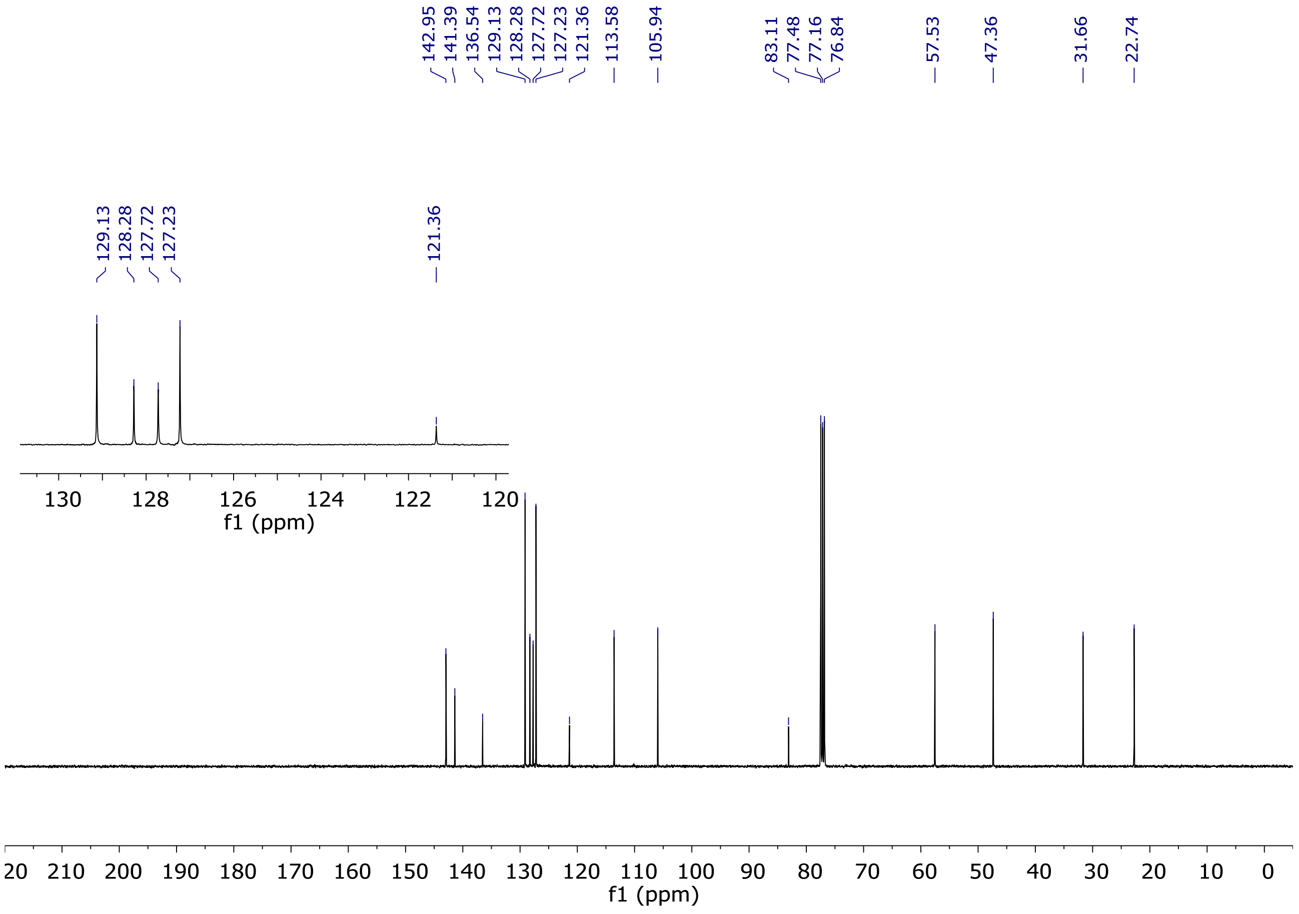
142.95
141.39
136.54
129.13
128.28
127.72
127.23
121.36
113.58
105.94
83.11
77.48
77.16
76.84
57.53
47.36
31.66
22.74
f1 (ppm)

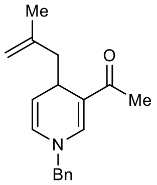
Me
O
Me
N
Bn

3ca

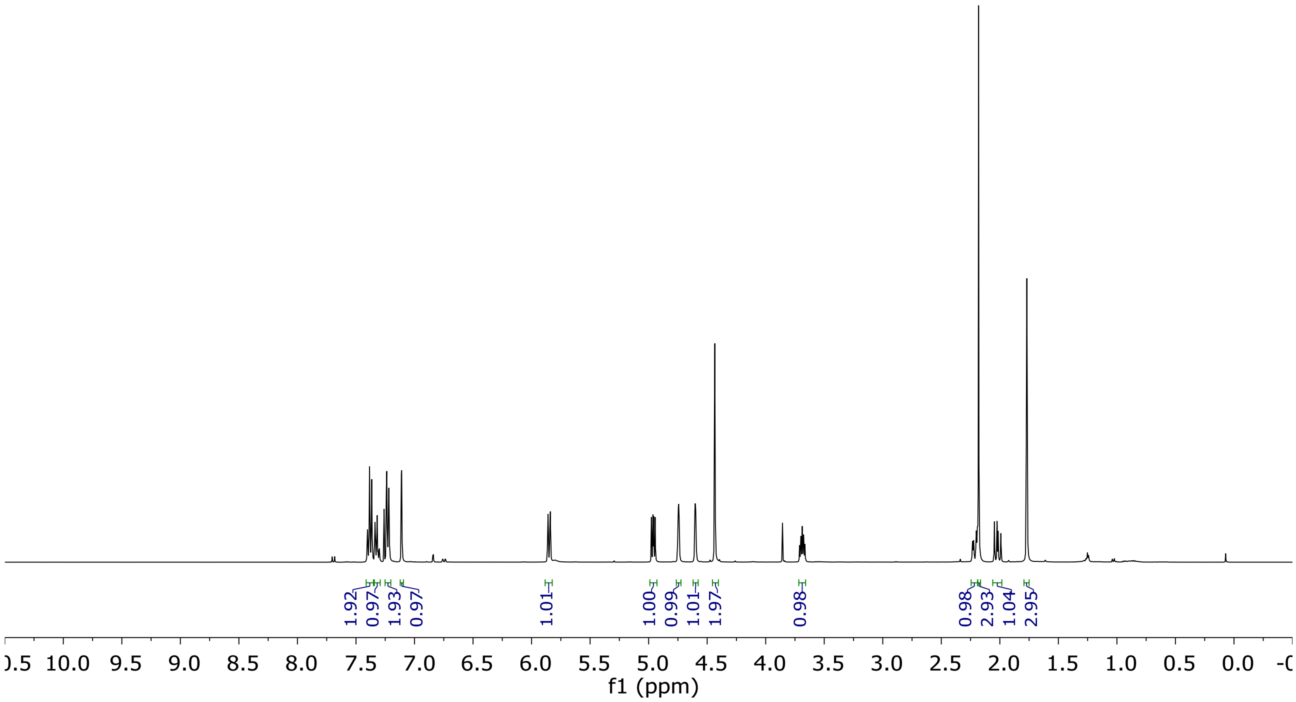
1.92
0.97
1.93
0.97
1.01
1.00
0.99
1.01
1.97
0.98
0.98
2.93
1.04
2.95
10.0 9.5 9.0 8.5 8.0 7.5 7.0 6.5 6.0 5.5 5.0 4.5 4.0 3.5 3.0 2.5 2.0 1.5 1.0 0.5 0.0
f1 (ppm)

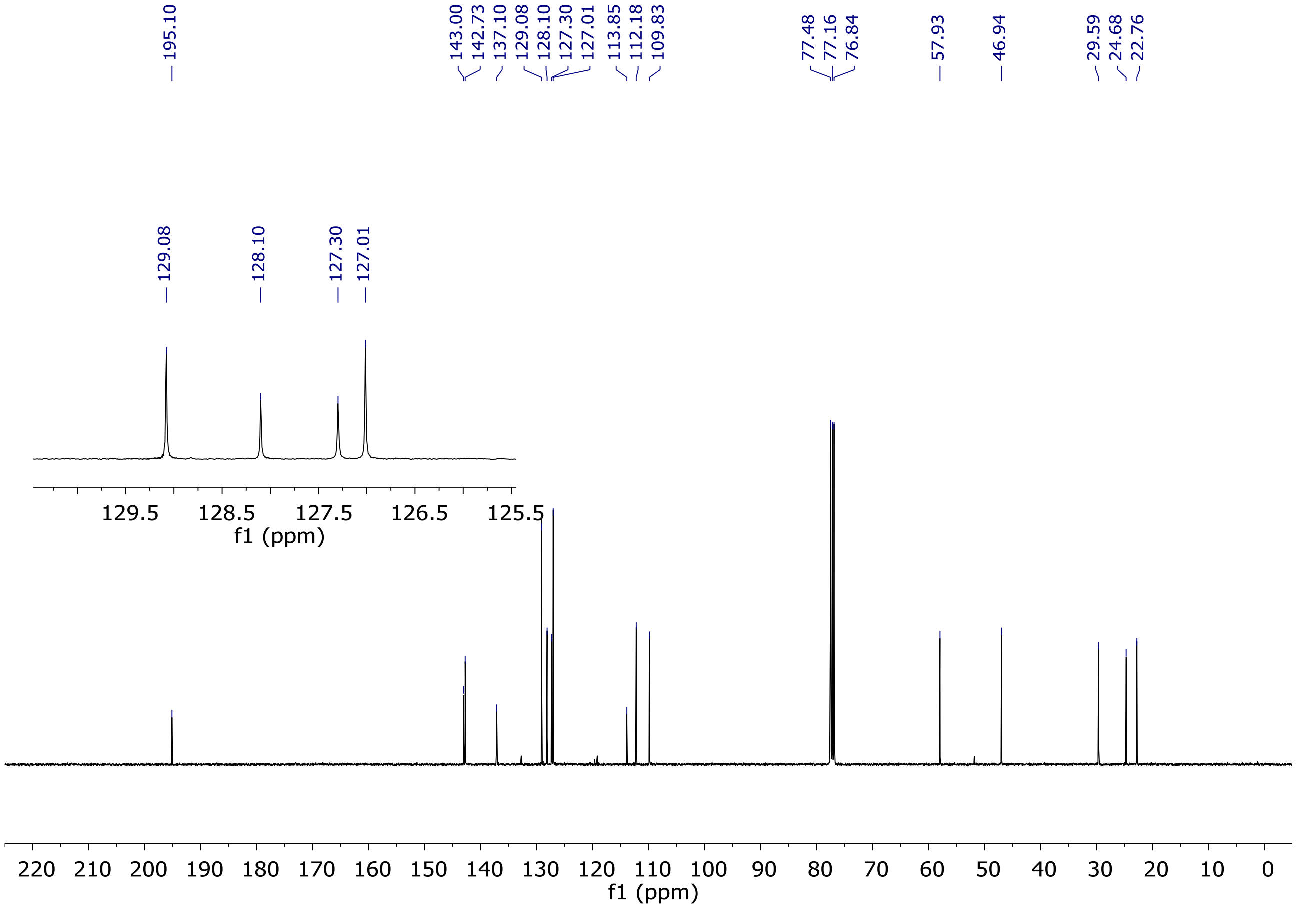
195.10
143.00
142.73
137.10
129.08
128.10
127.30
127.01
113.85
112.18
109.83
77.48
77.16
76.84
57.93
46.94
29.59
24.68
22.76
129.08
128.10
127.30
127.01
129.5 128.5 127.5 126.5 125.5
f1 (ppm)
220 210 200 190 180 170 160 150 140 130 120 110 100 90 80 70 60 50 40 30 20 10 0
f1 (ppm)

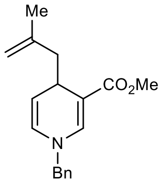
Me
CO2Me
N
Bn

3da

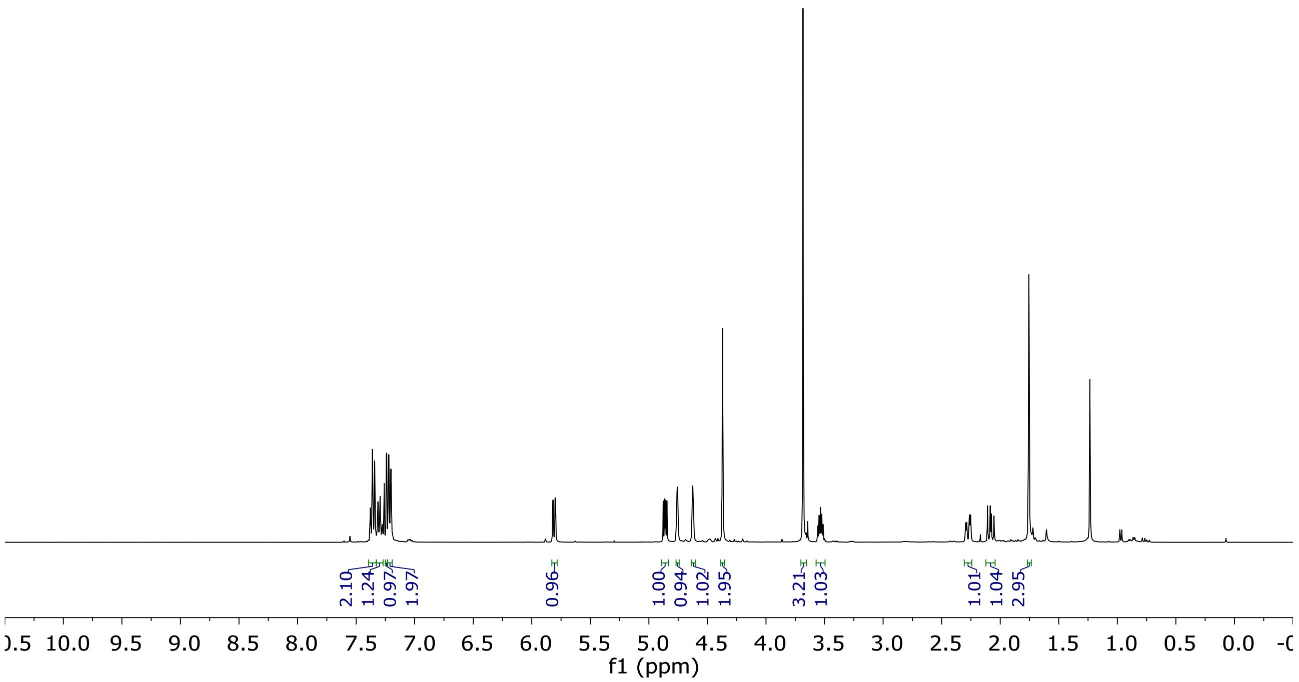
2.10
1.24
0.97
1.97
0.96
1.00
0.94
1.02
1.95
3.21
1.03
1.01
1.04
2.95
f1 (ppm)

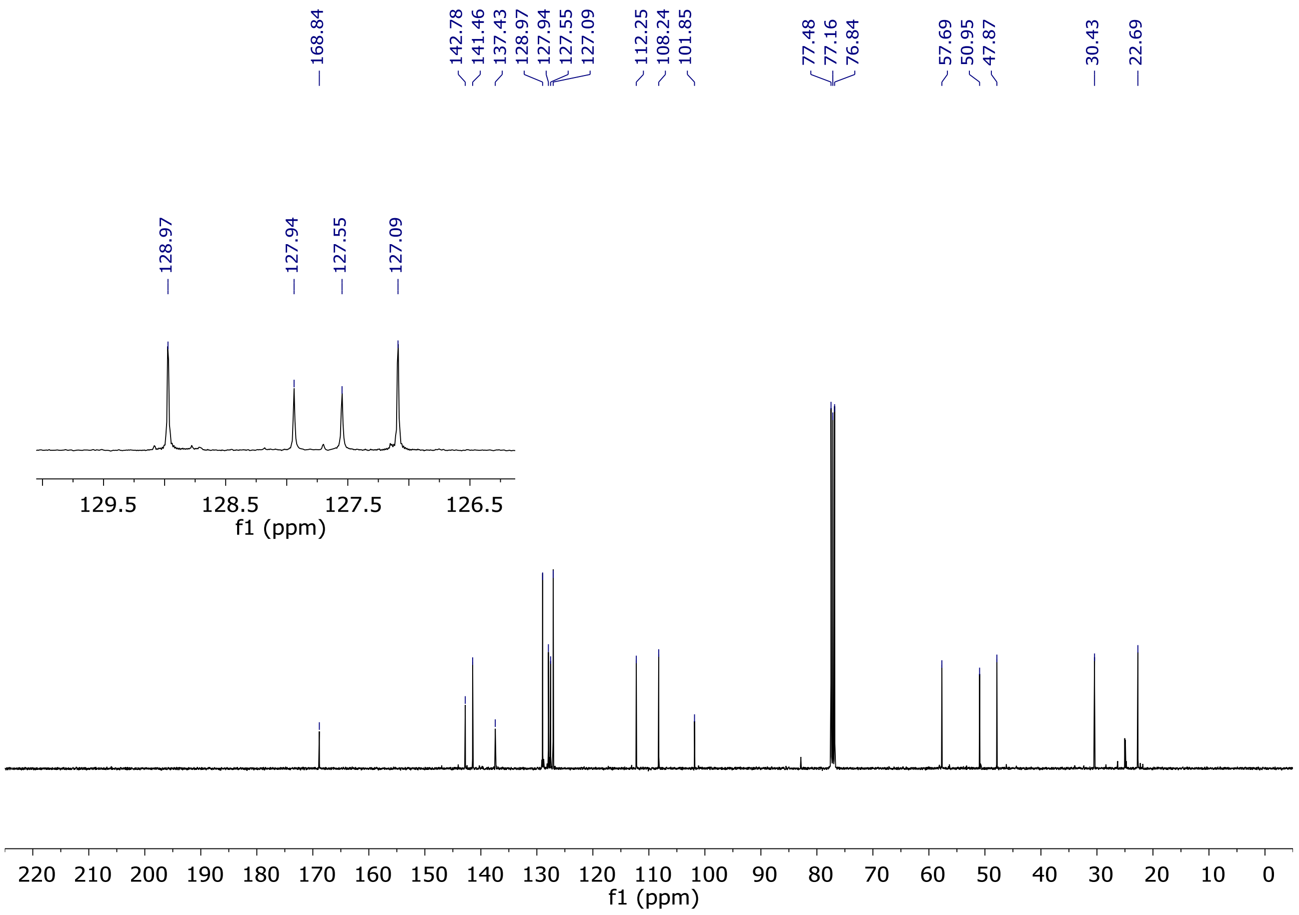
168.84
142.78
141.46
137.43
128.97
127.94
127.55
127.09
112.25
108.24
101.85
77.48
77.16
76.84
57.69
50.95
47.87
30.43
22.69
f1 (ppm)

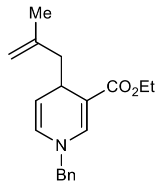
Me
CO_2Et
N
Bn

3ea

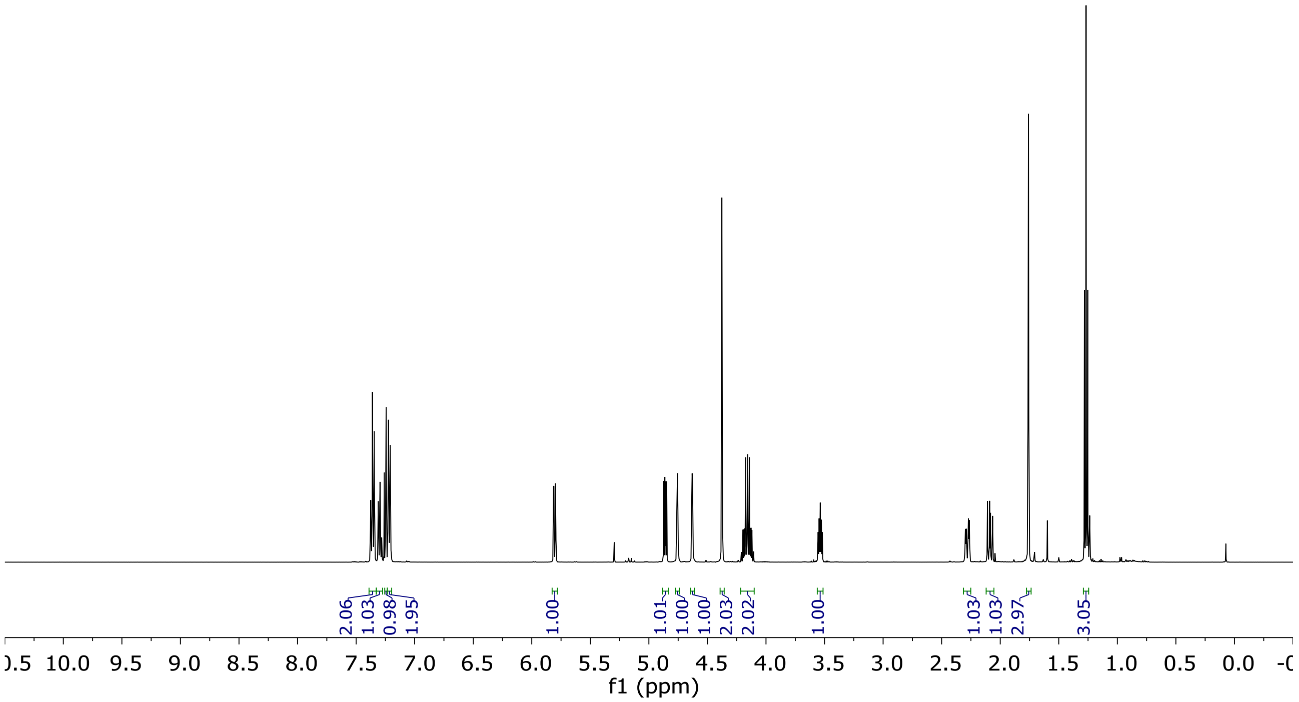
2.06
1.03
0.98
1.95
1.00
1.01
1.00
1.00
2.03
2.02
1.00
1.03
1.03
2.97
3.05
f1 (ppm)

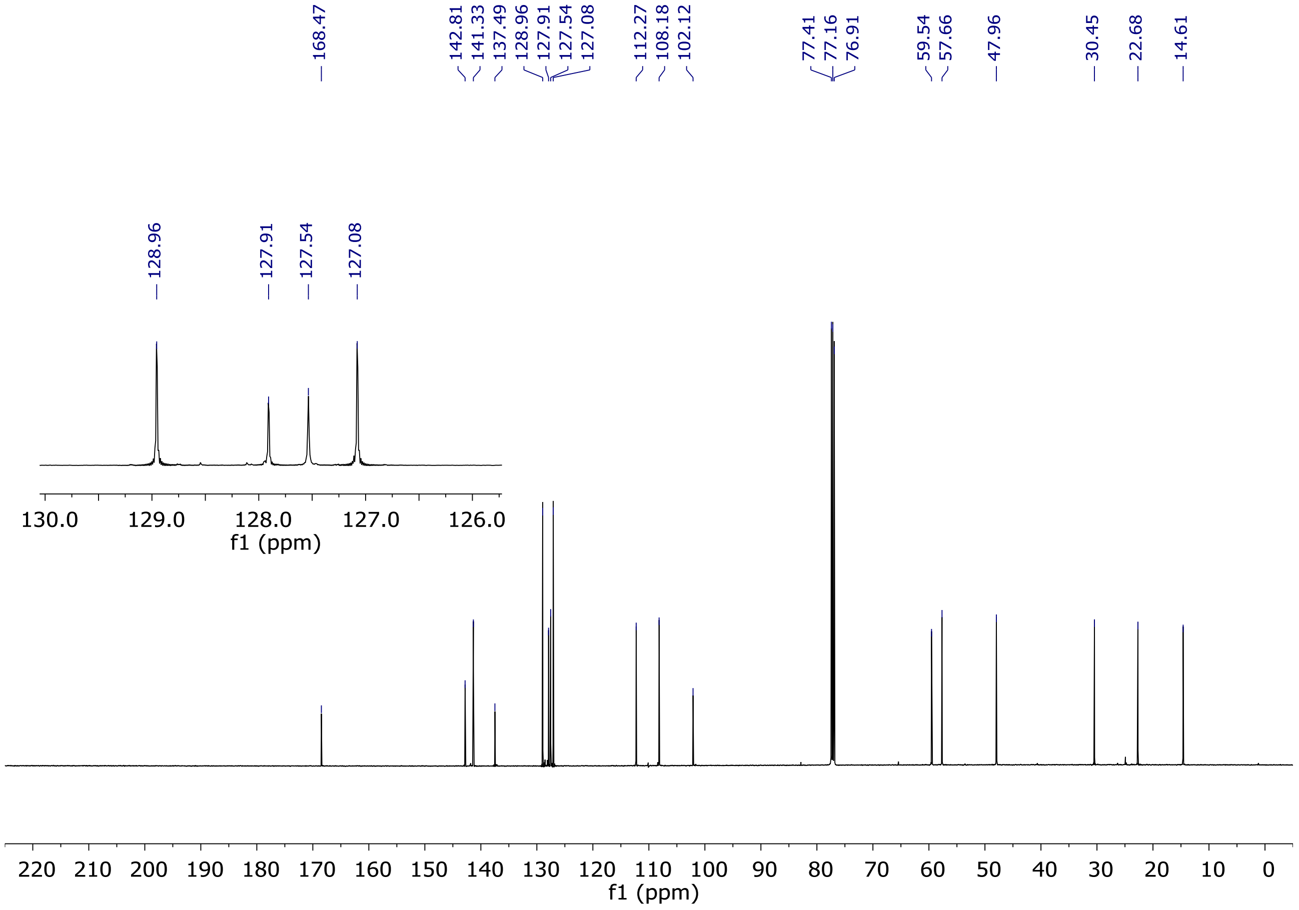
168.47
142.81
141.33
137.49
128.96
127.91
127.54
127.08
112.27
108.18
102.12
77.41
77.16
76.91
59.54
57.66
47.96
30.45
22.68
14.61
f1 (ppm)

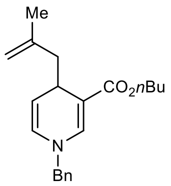
Me
CO2nBu
N
Bn

3fa

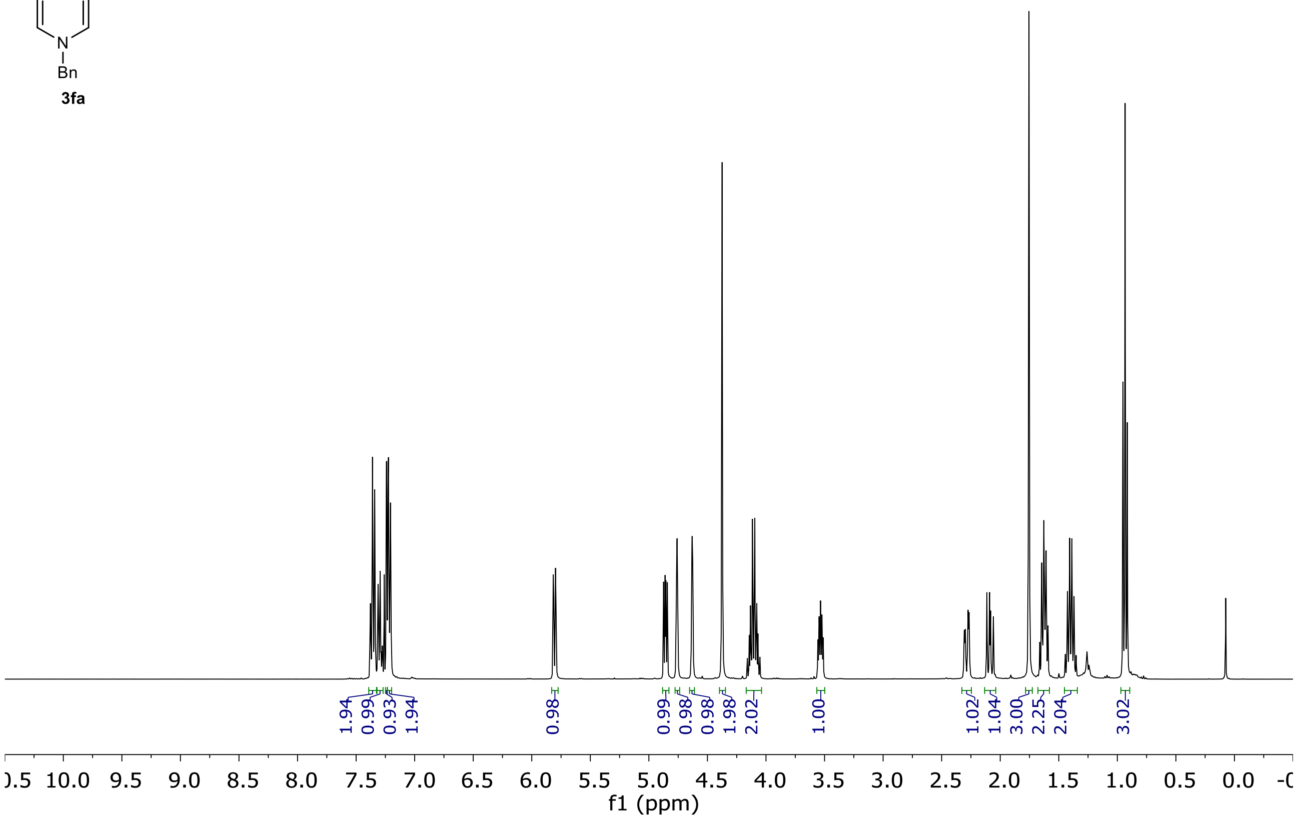
1.94
0.99
0.93
1.94
0.98
0.99
0.98
0.98
1.98
2.02
1.00
1.02
1.04
3.00
2.25
2.04
3.02
f1 (ppm)

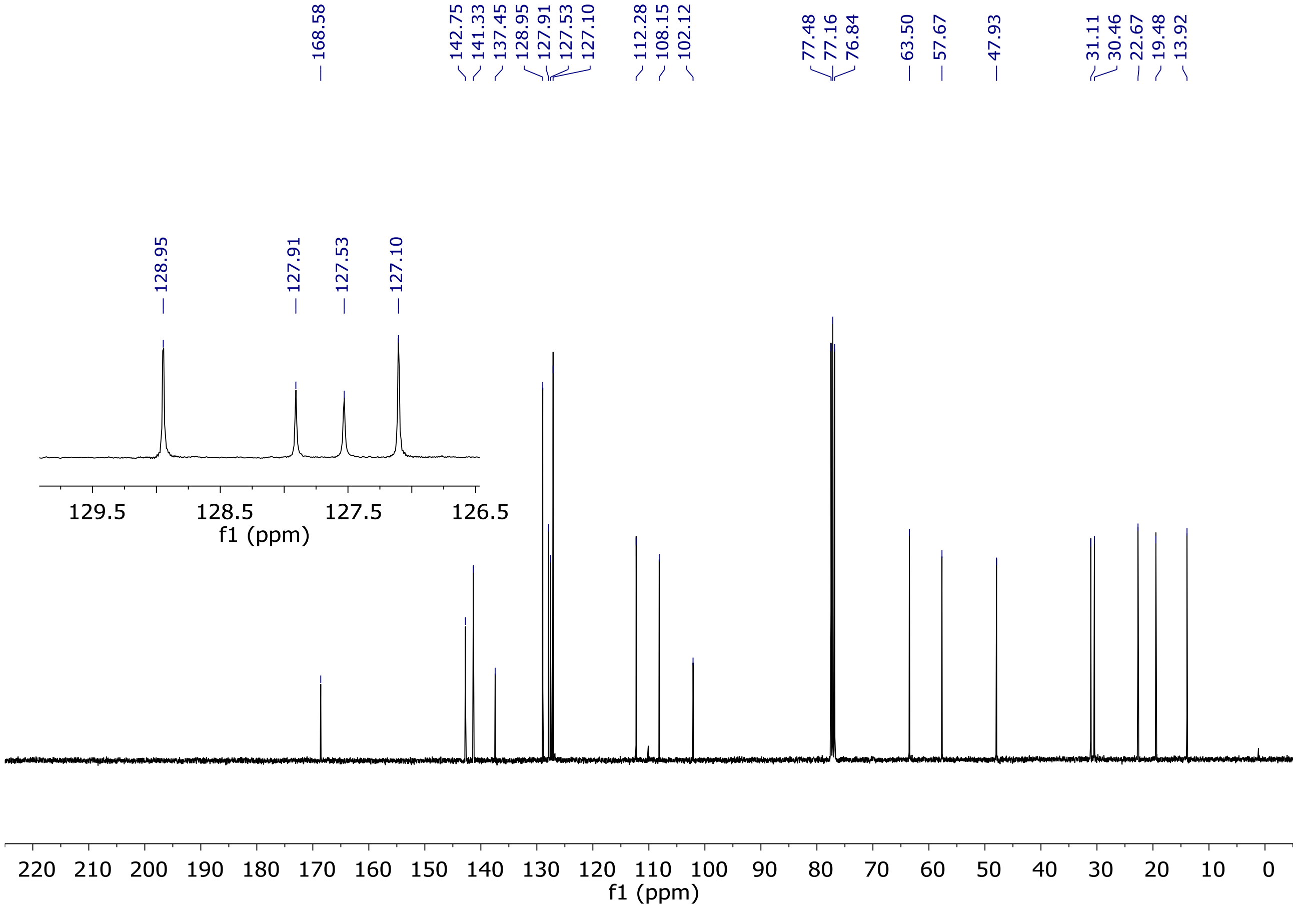
168.58
142.75
141.33
137.45
128.95
127.91
127.53
127.10
112.28
108.15
102.12
77.48
77.16
76.84
63.50
57.67
47.93
31.11
30.46
22.67
19.48
13.92
128.95
127.91
127.53
127.10
f1 (ppm)
f1 (ppm)

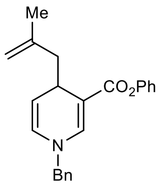
Me
CO_2Ph
N
Bn

3ga

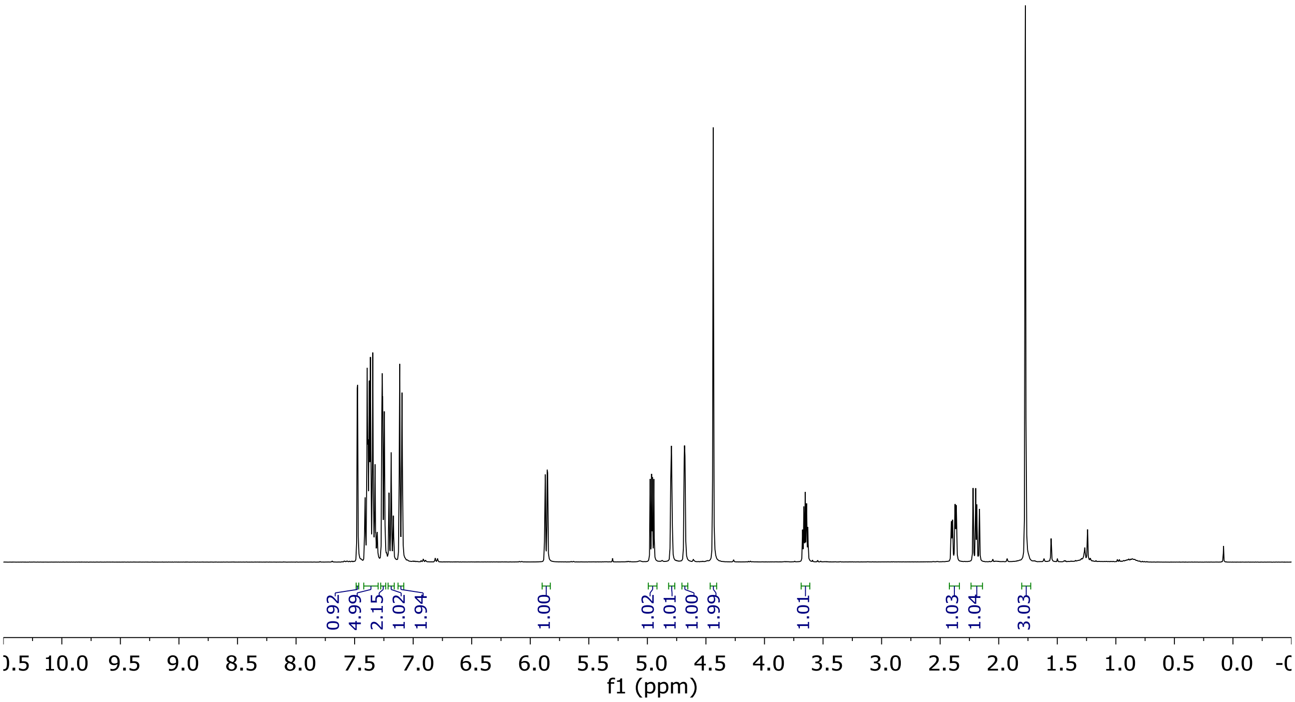
0.92
4.99
2.15
1.02
1.94
1.00
1.02
1.01
1.00
1.99
1.01
1.03
1.04
3.03
f1 (ppm)

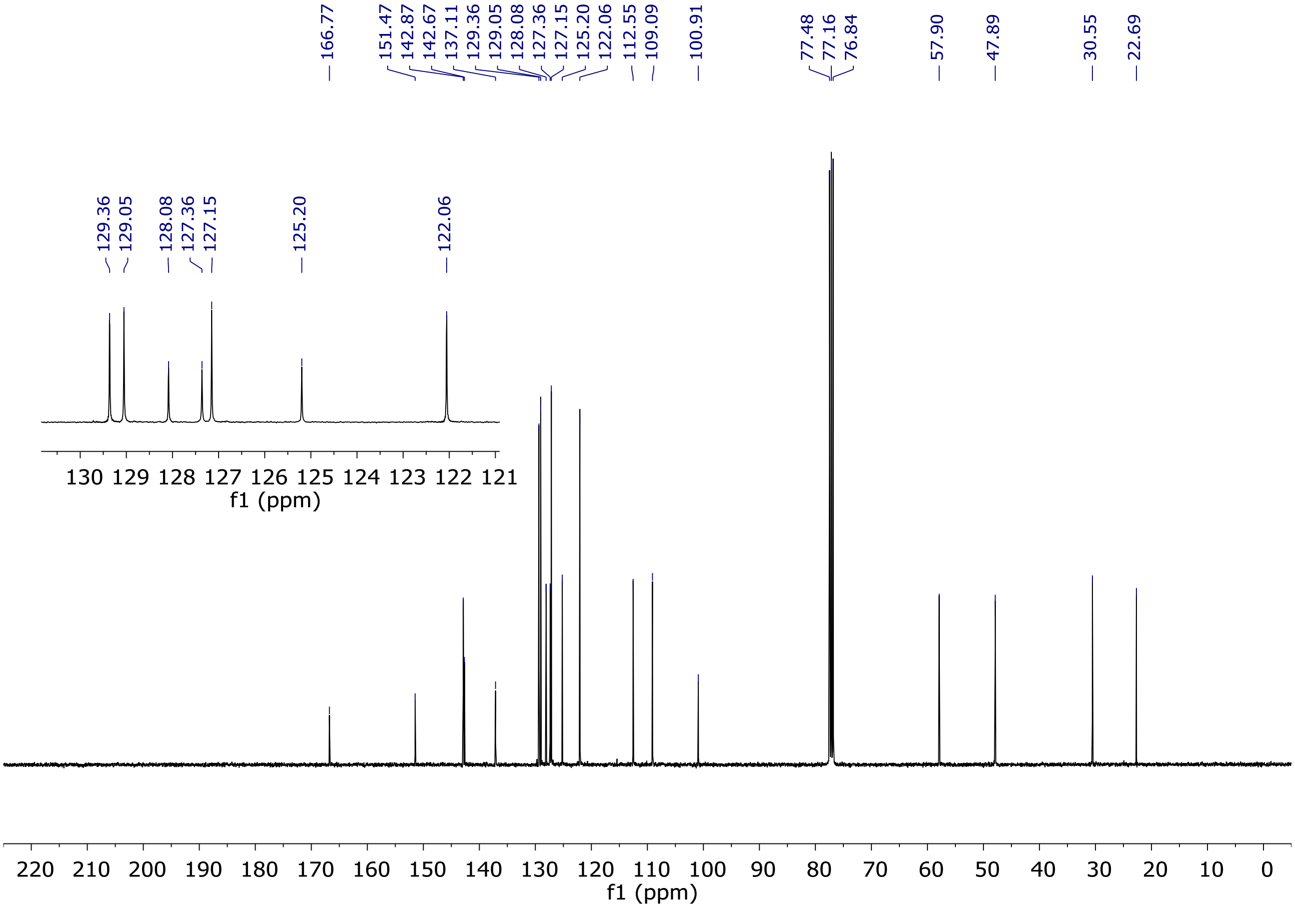
166.77
151.47
142.87
142.67
137.11
129.36
129.05
128.08
127.36
127.15
125.20
122.06
112.55
109.09
100.91
77.48
77.16
76.84
57.90
47.89
30.55
22.69
f1 (ppm)

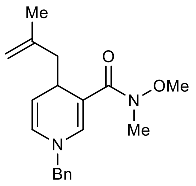
Me
O
OMe
N
Me
N
Bn

3ha

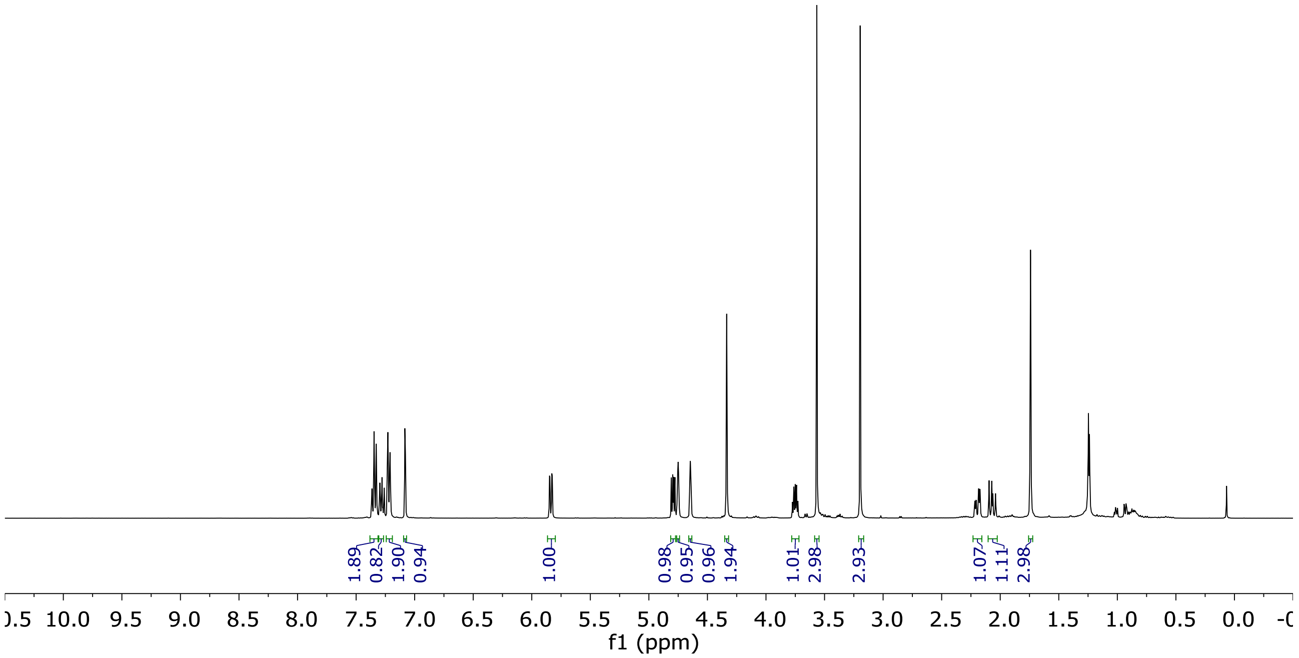
1.89
0.82
1.90
0.94
1.00
0.98
0.95
0.96
1.94
1.01
2.98
2.93
1.07
1.11
2.98
f1 (ppm)

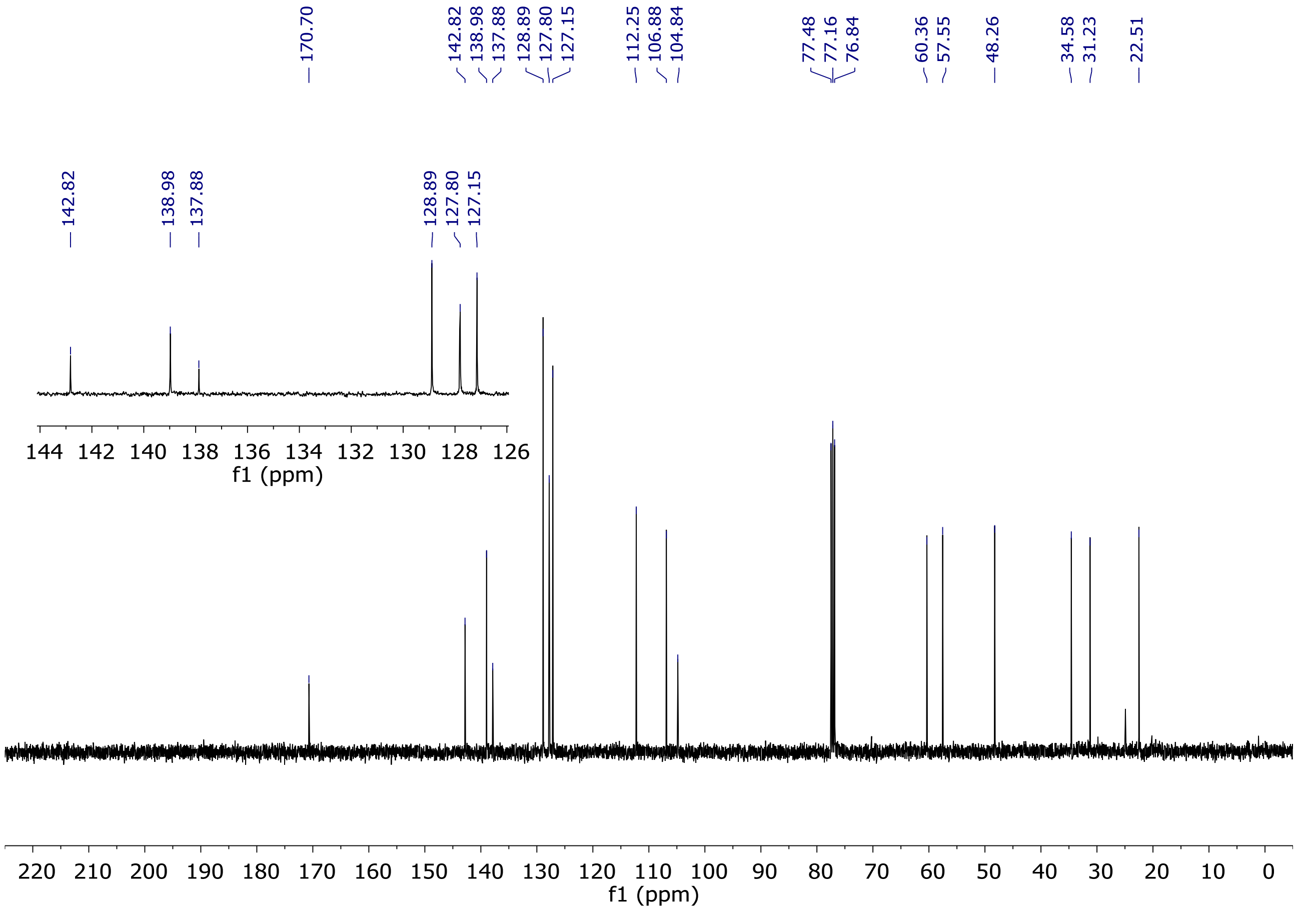
170.70
142.82
138.98
137.88
128.89
127.80
127.15
112.25
106.88
104.84
77.48
77.16
76.84
60.36
57.55
48.26
34.58
31.23
22.51
f1 (ppm)

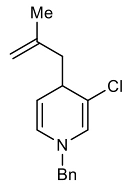
Me
Cl
N
Bn

3ia

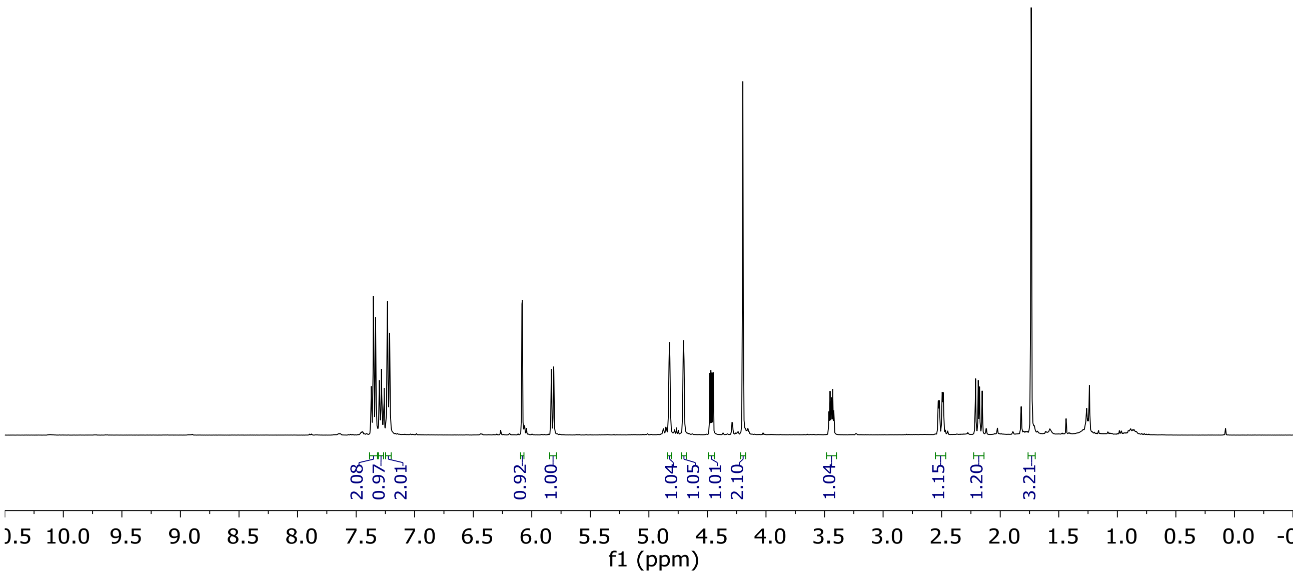
2.08
0.97
2.01
0.92
1.00
1.04
1.05
1.01
2.10
1.04
1.15
1.20
3.21
f1 (ppm)

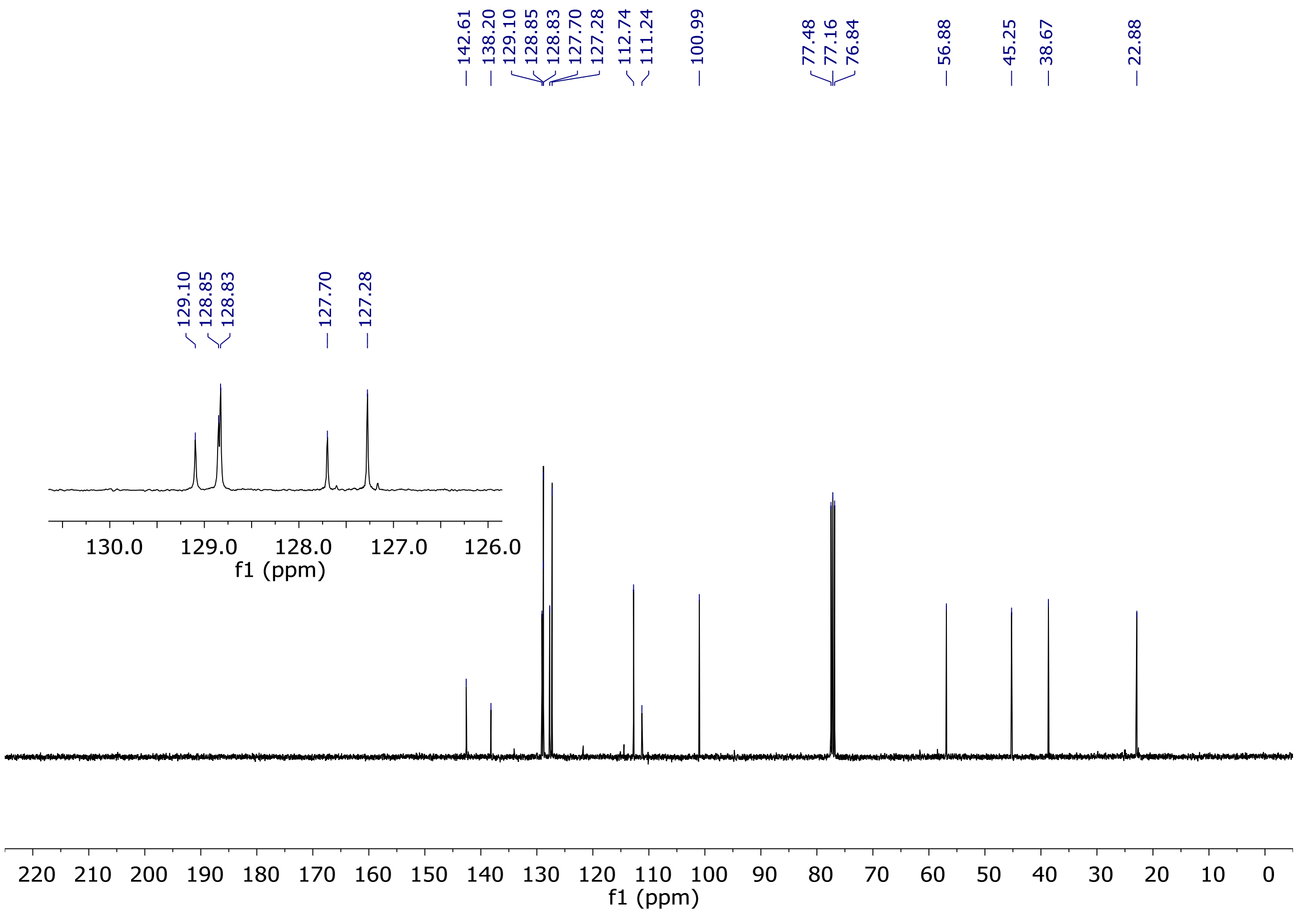
142.61
138.20
129.10
128.85
128.83
127.70
127.28
112.74
111.24
100.99
77.48
77.16
76.84
56.88
45.25
38.67
22.88
129.10
128.85
128.83
127.70
127.28
130.0
129.0
128.0
127.0
126.0
f1 (ppm)
f1 (ppm)

HMBC Spectrum

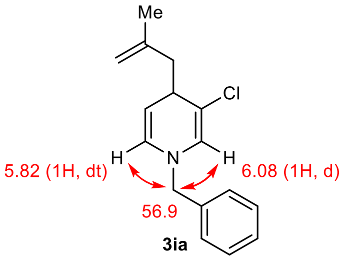

3ia

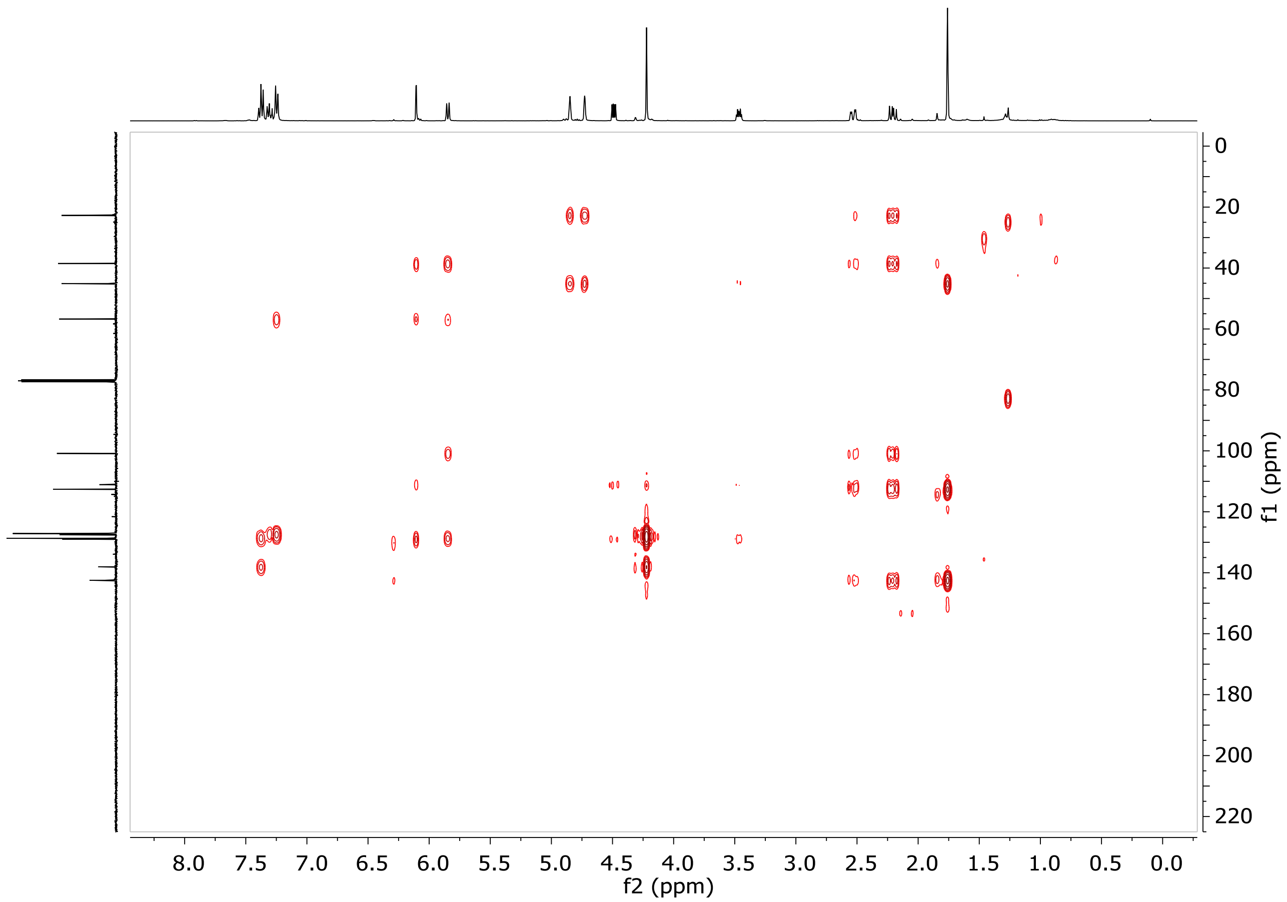

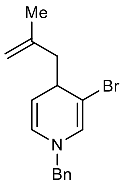
Me
Br
N
Bn

3ja

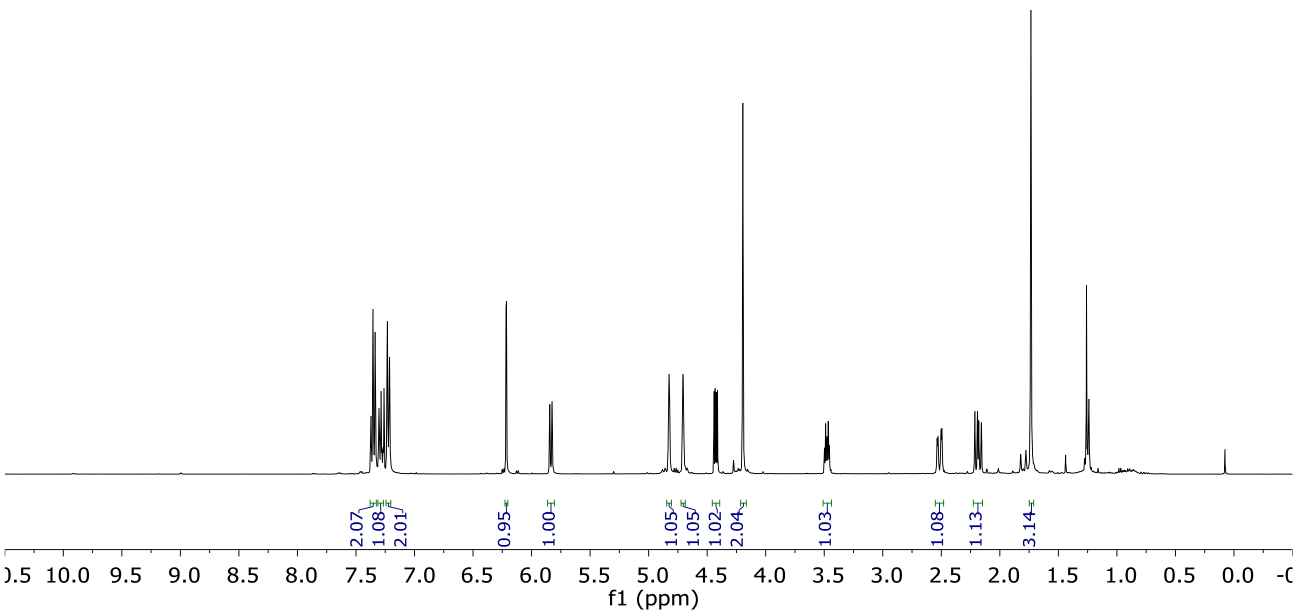
2.07
1.08
2.01
0.95
1.00
1.05
1.05
1.02
2.04
1.03
1.08
1.13
3.14
10.0 9.5 9.0 8.5 8.0 7.5 7.0 6.5 6.0 5.5 5.0 4.5 4.0 3.5 3.0 2.5 2.0 1.5 1.0 0.5 0.0
f1 (ppm)

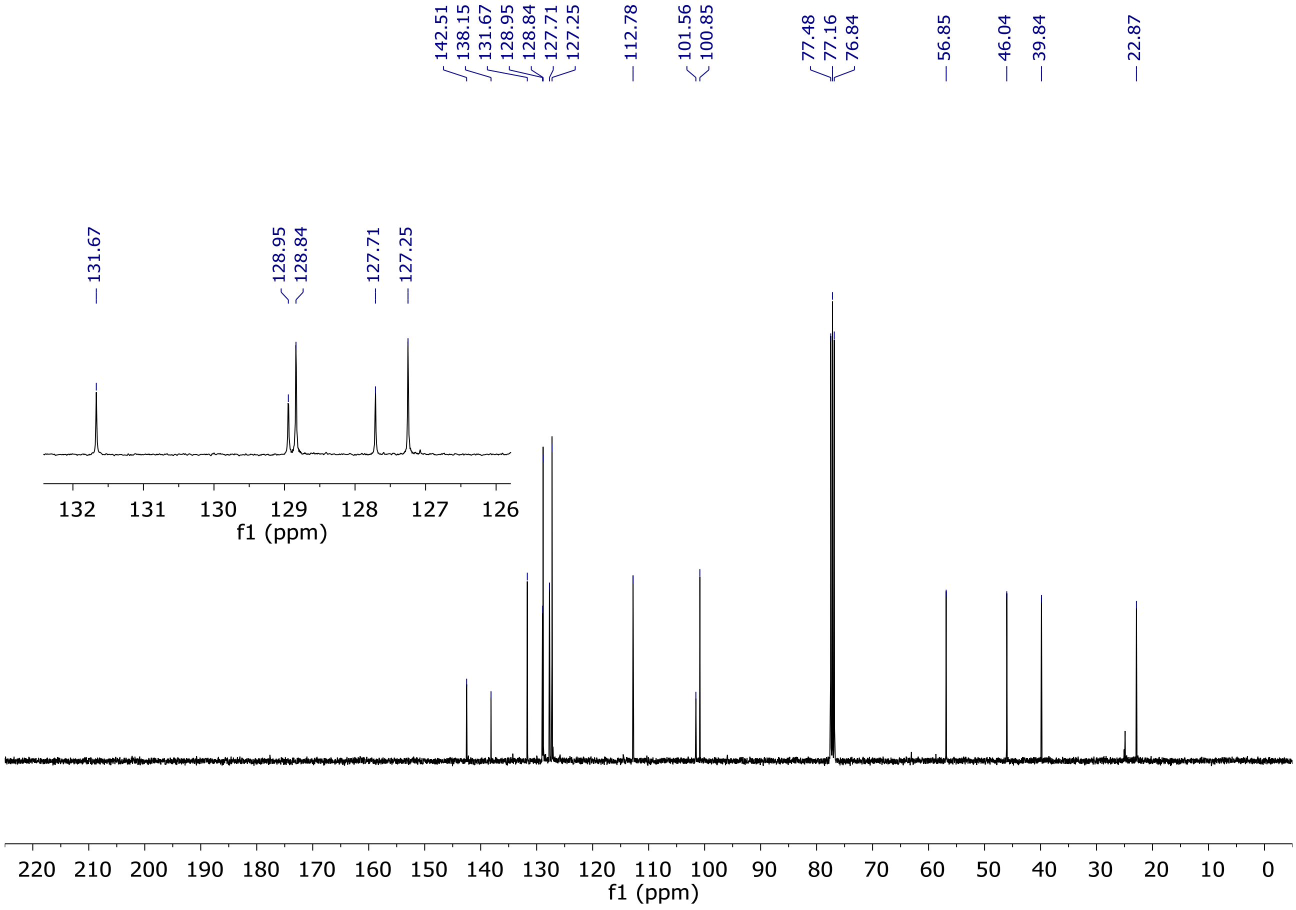
142.51
138.15
131.67
128.95
128.84
127.71
127.25
112.78
101.56
100.85
77.48
77.16
76.84
56.85
46.04
39.84
22.87
131.67
128.95
128.84
127.71
127.25
132 131 130 129 128 127 126
f1 (ppm)
220 210 200 190 180 170 160 150 140 130 120 110 100 90 80 70 60 50 40 30 20 10 0
f1 (ppm)

HMBC Spectrum

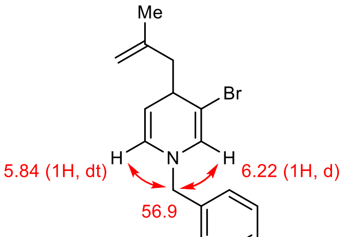

3ja

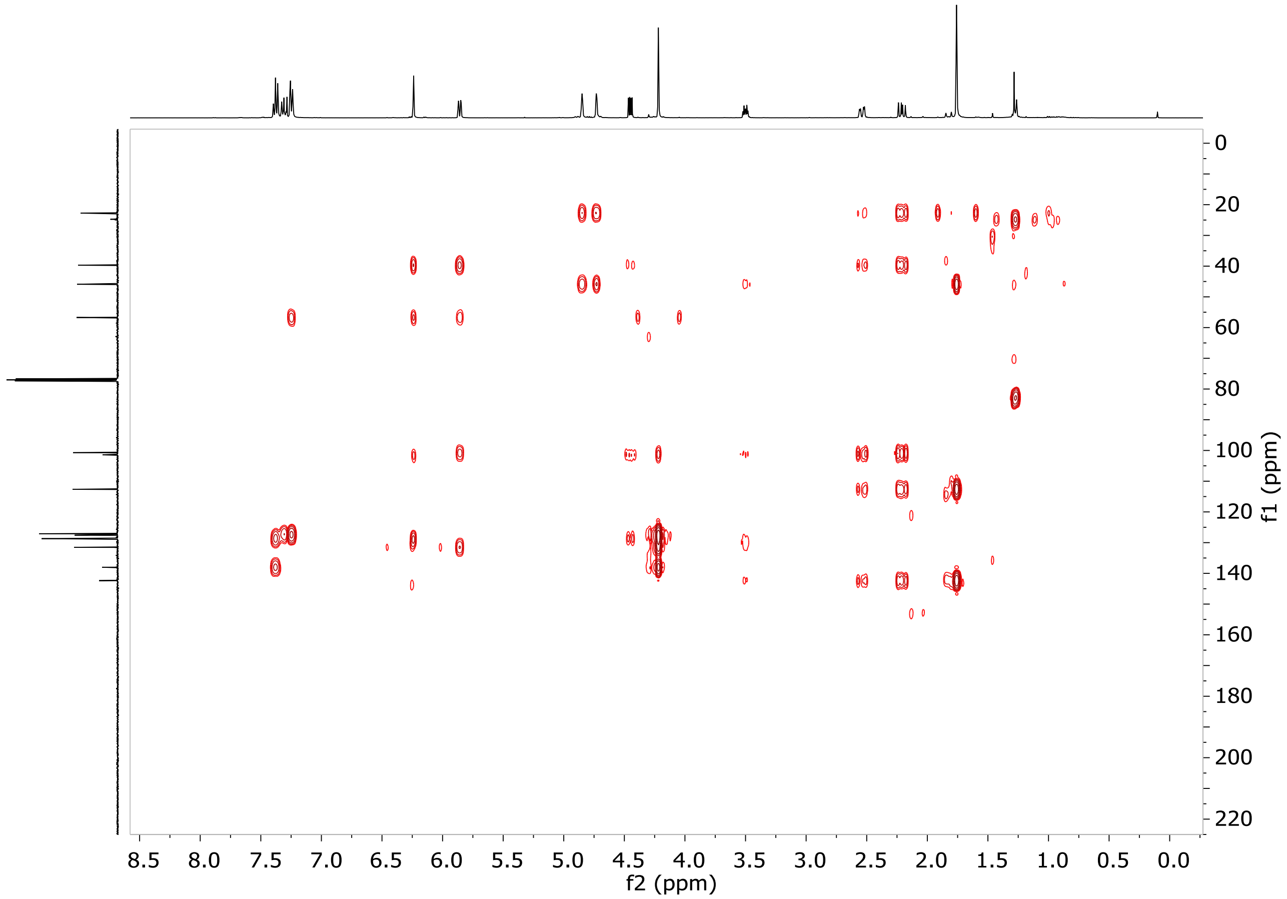

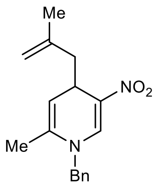
Me
NO2
Me
N
Bn

3ma

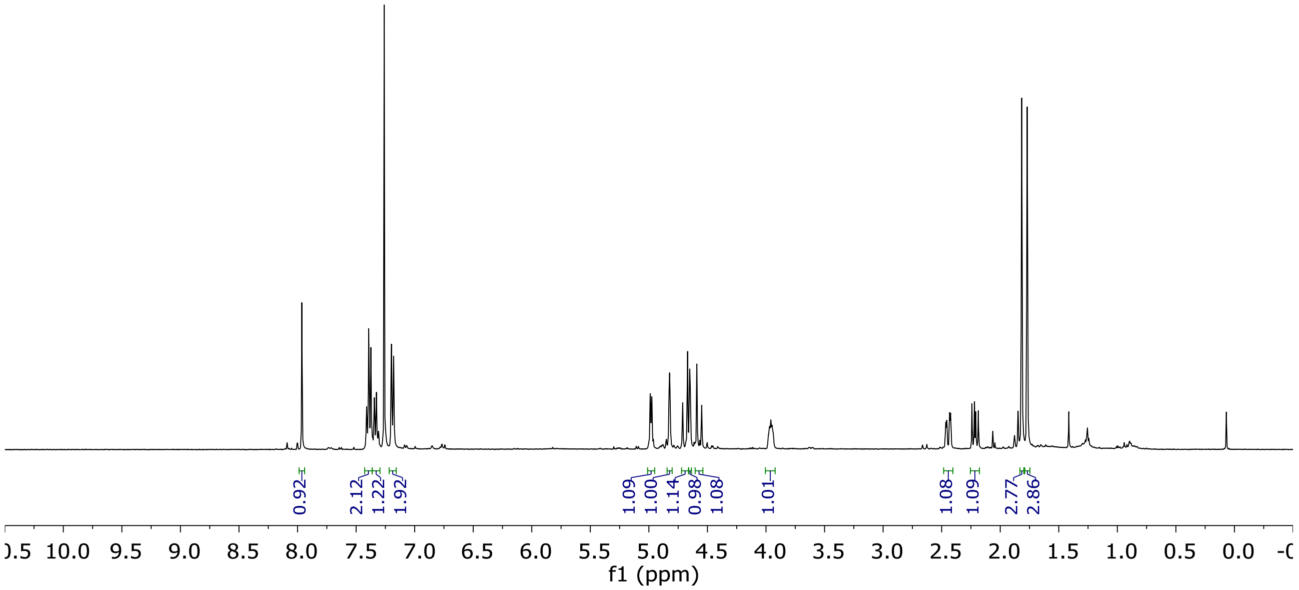
0.92
2.12
1.22
1.92
1.09
1.00
1.14
0.98
1.08
1.01
1.08
1.09
2.77
2.86
f1 (ppm)

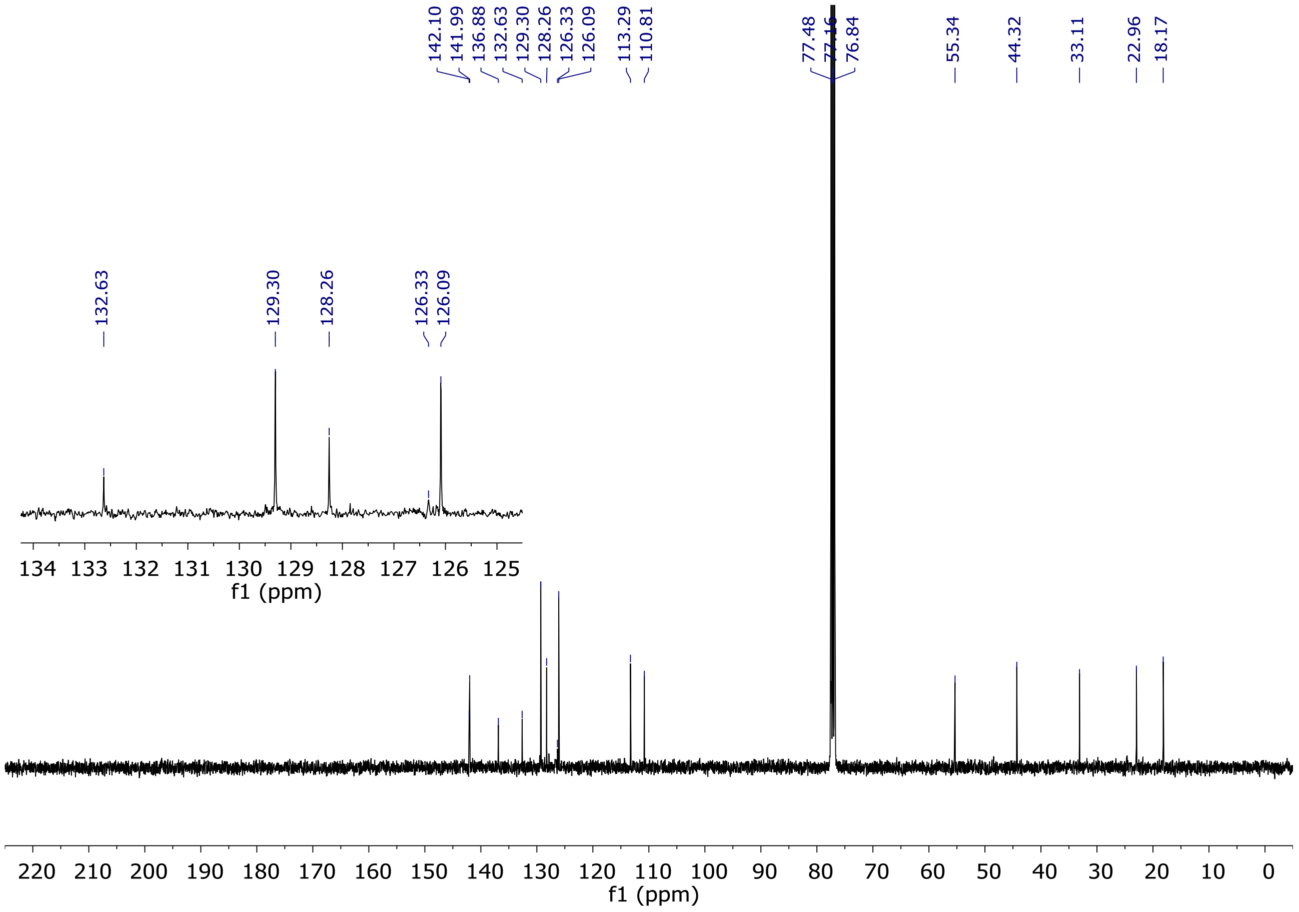
142.10
141.99
136.88
132.63
129.30
128.26
126.33
126.09
113.29
110.81
77.48
77.16
76.84
55.34
44.32
33.11
22.96
18.17
f1 (ppm)

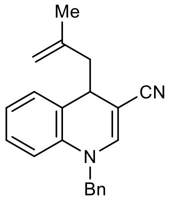
Me
CN
N
Bn

3na

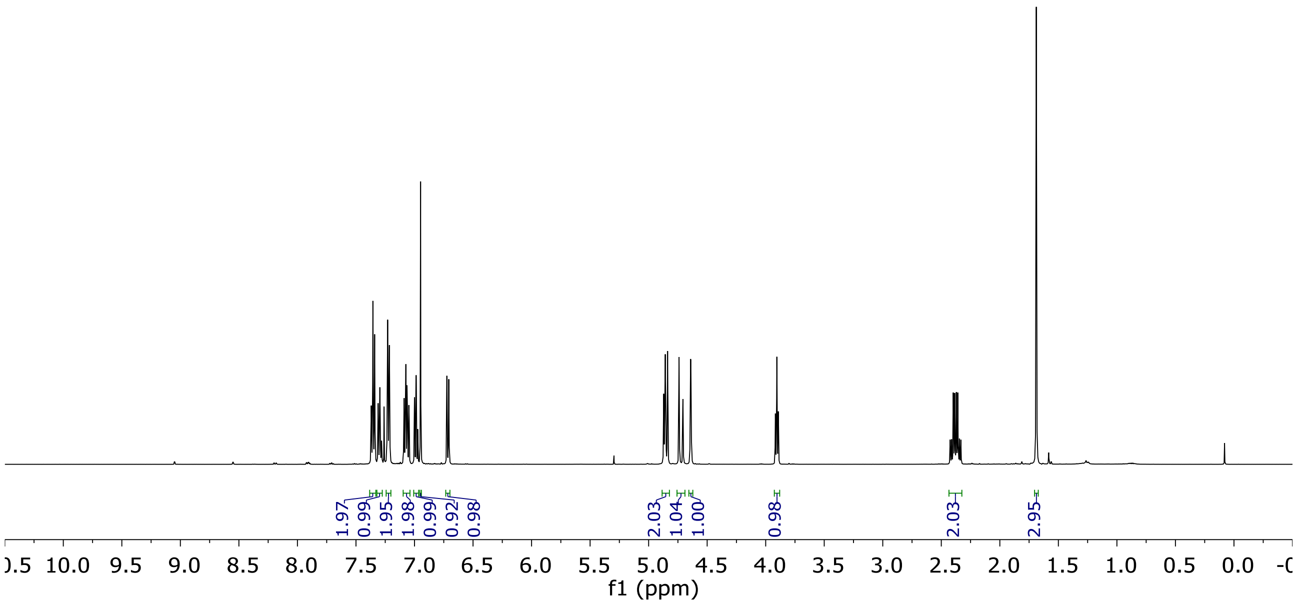
1.97 0.99 1.95 1.98 0.99 0.92 0.98
2.03 1.04 1.00
0.98
2.03
2.95
10.0 9.5 9.0 8.5 8.0 7.5 7.0 6.5 6.0 5.5 5.0 4.5 4.0 3.5 3.0 2.5 2.0 1.5 1.0 0.5 0.0
f1 (ppm)

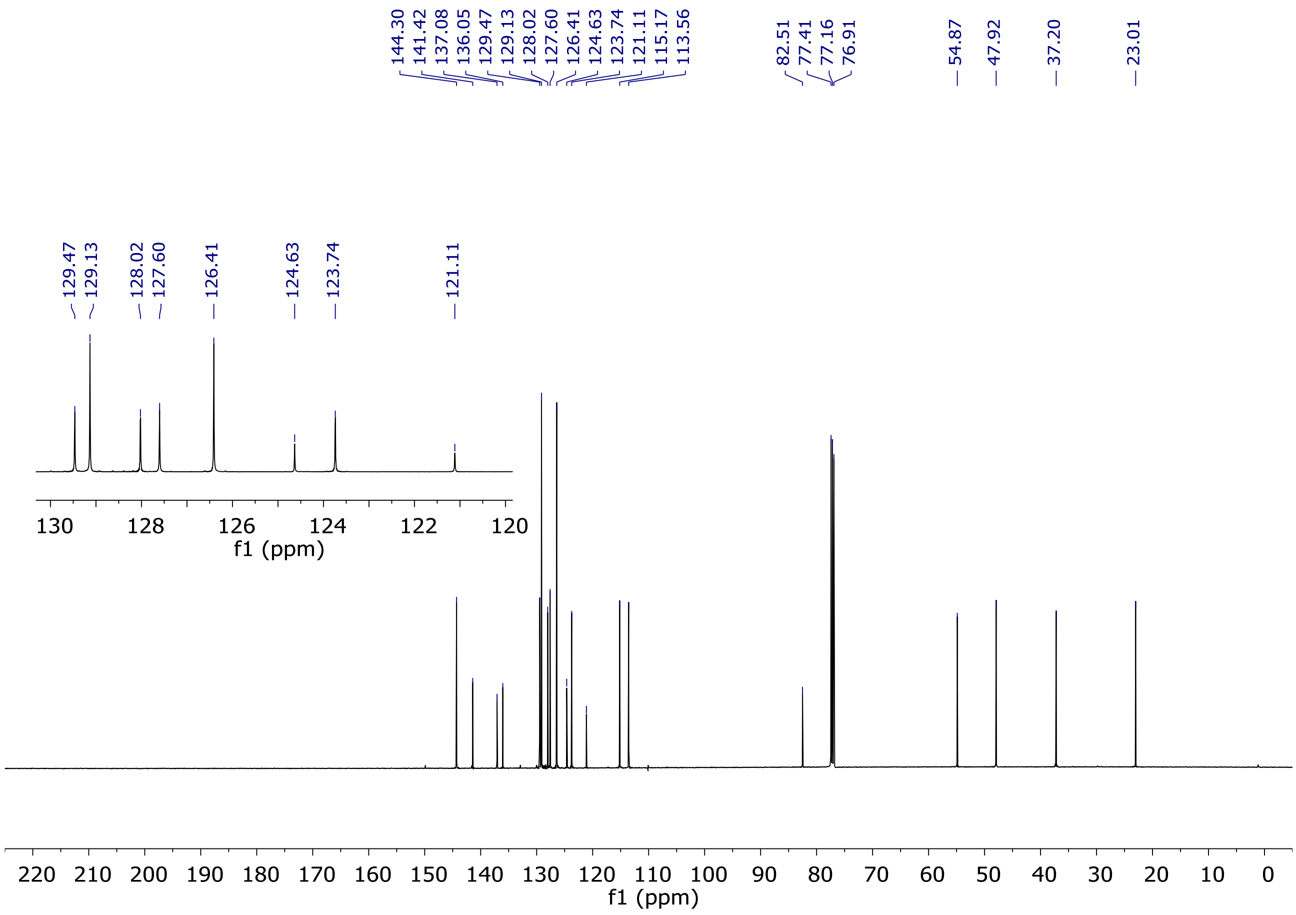
144.30 141.42 137.08 136.05 129.47 129.13 128.02 127.60 126.41 124.63 123.74 121.11 115.17 113.56
82.51 77.41 77.16 76.91
54.87
47.92
37.20
23.01
129.47 129.13 128.02 127.60 126.41 124.63 123.74 121.11
130 128 126 124 122 120
f1 (ppm)
220 210 200 190 180 170 160 150 140 130 120 110 100 90 80 70 60 50 40 30 20 10 0
f1 (ppm)

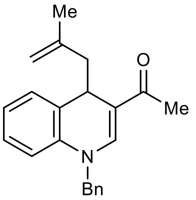
Me
O
Me
N
Bn

3oa

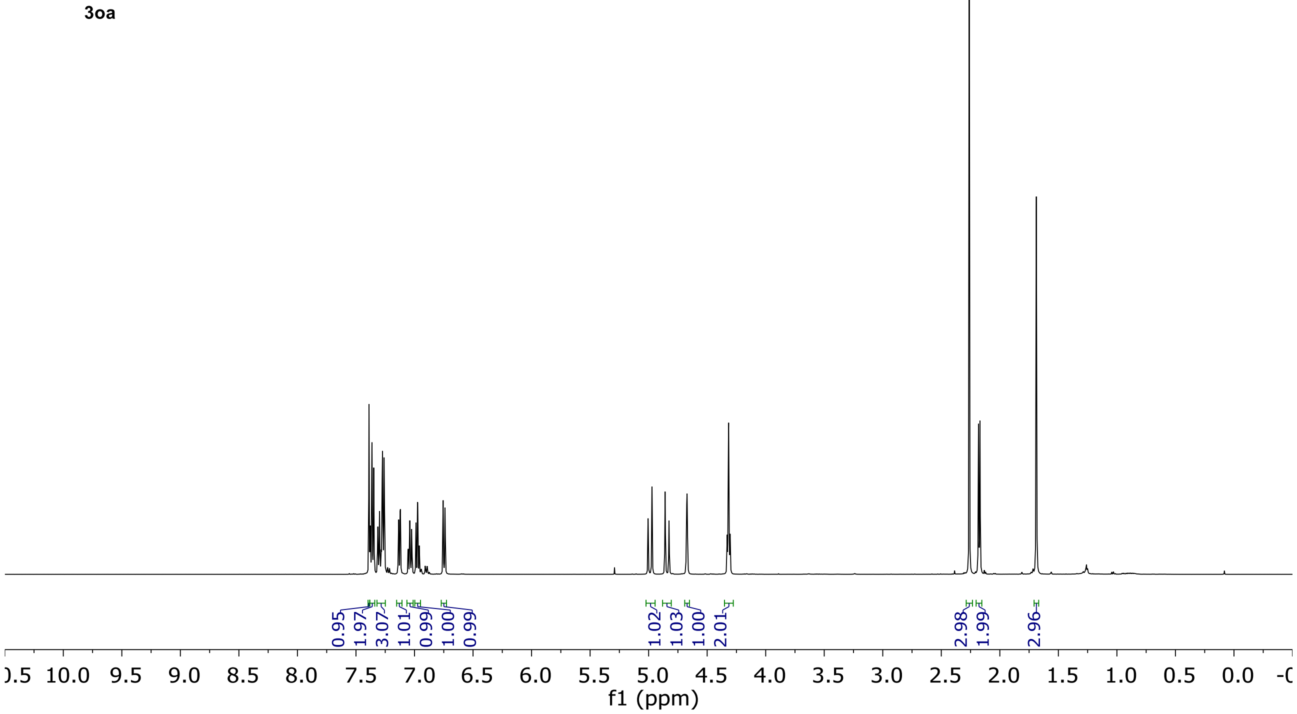
0.95
1.97
3.07
1.01
0.99
1.00
0.99
1.02
1.03
1.00
2.01
2.98
1.99
2.96
10.0 9.5 9.0 8.5 8.0 7.5 7.0 6.5 6.0 5.5 5.0 4.5 4.0 3.5 3.0 2.5 2.0 1.5 1.0 0.5 0.0
f1 (ppm)

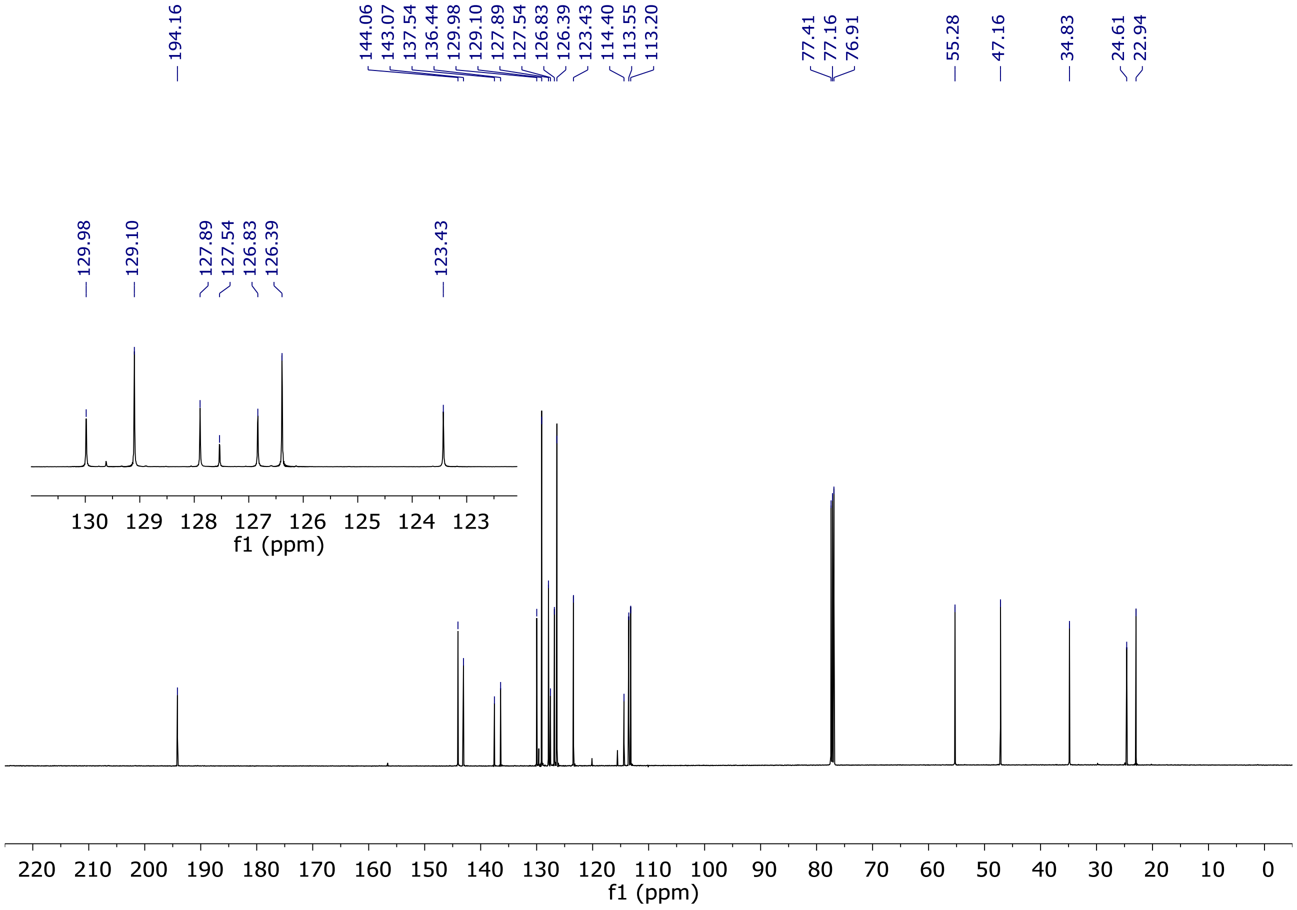
194.16
144.06
143.07
137.54
136.44
129.98
129.10
127.89
127.54
126.83
126.39
123.43
114.40
113.55
113.20
77.41
77.16
76.91
55.28
47.16
34.83
24.61
22.94
129.98
129.10
127.89
127.54
126.83
126.39
123.43
130 129 128 127 126 125 124 123
f1 (ppm)
220 210 200 190 180 170 160 150 140 130 120 110 100 90 80 70 60 50 40 30 20 10 0
f1 (ppm)

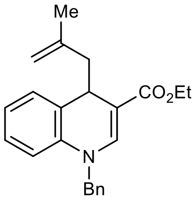
Me
CO2Et
N
Bn

3pa

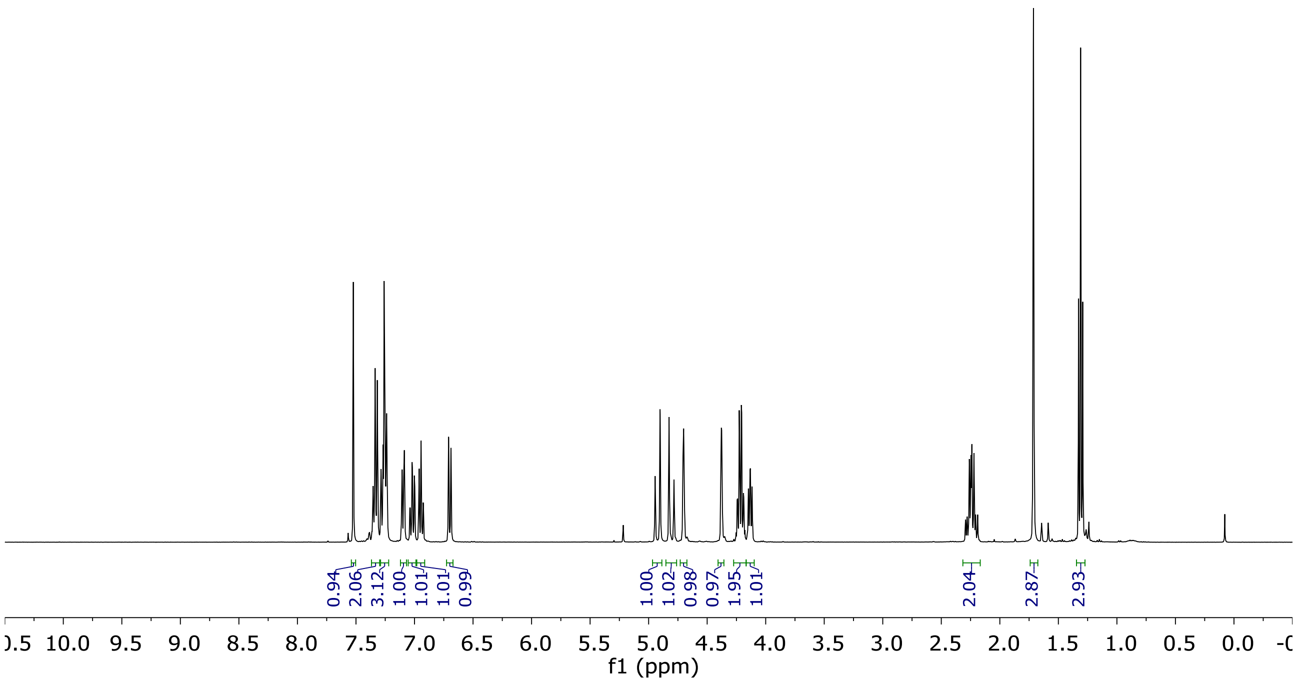
0.94
2.06
3.12
1.00
1.01
1.01
0.99
1.00
1.02
0.98
0.97
1.95
1.01
2.04
2.87
2.93
f1 (ppm)

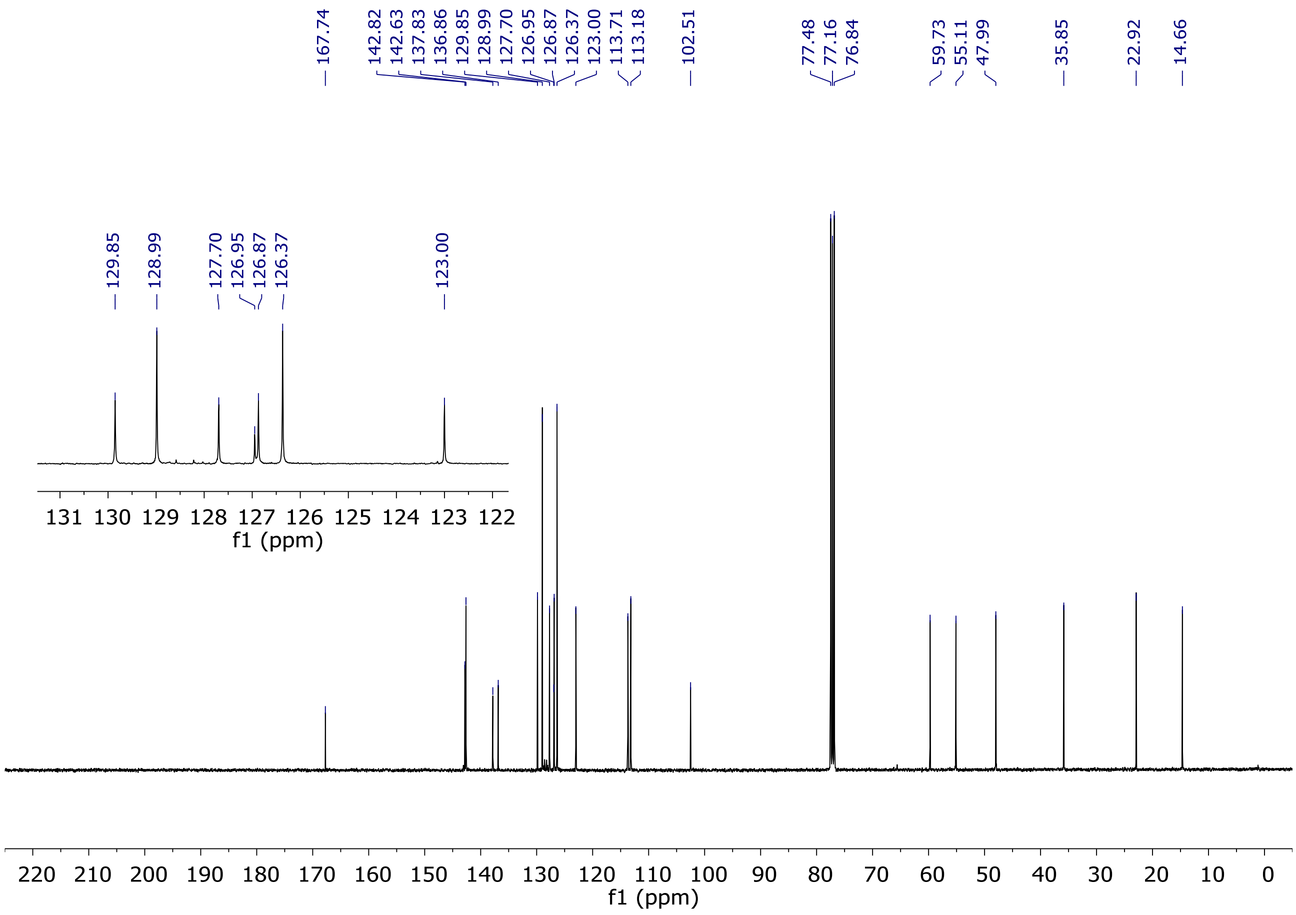
167.74
142.82
142.63
137.83
136.86
129.85
128.99
127.70
126.95
126.87
126.37
123.00
113.71
113.18
102.51
77.48
77.16
76.84
59.73
55.11
47.99
35.85
22.92
14.66
f1 (ppm)

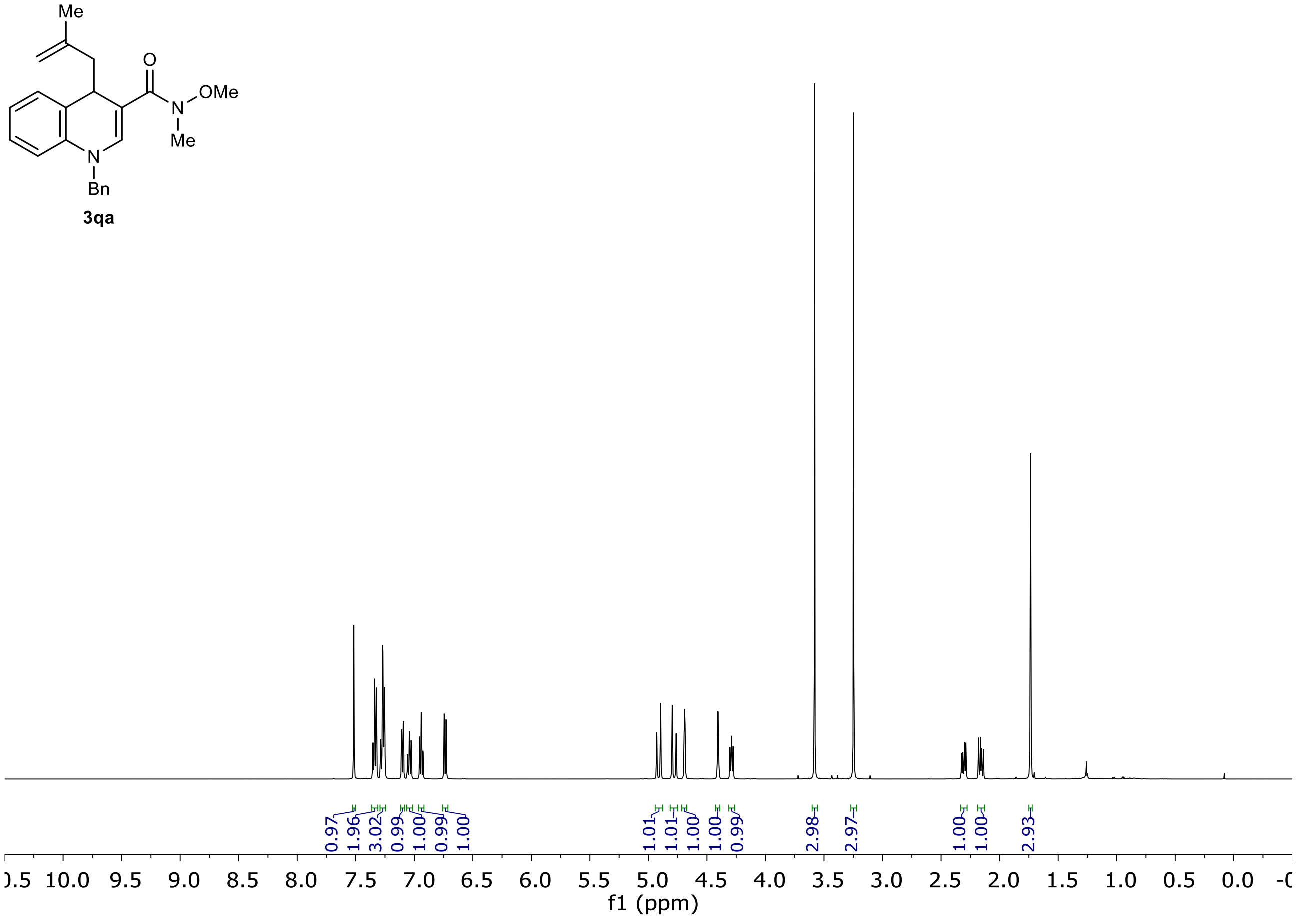
Me
O
OMe
N
Me
N
Bn
3qa
0.97
1.96
3.02
0.99
1.00
0.99
1.00
1.01
1.01
1.00
1.00
0.99
2.98
2.97
1.00
1.00
2.93
f1 (ppm)

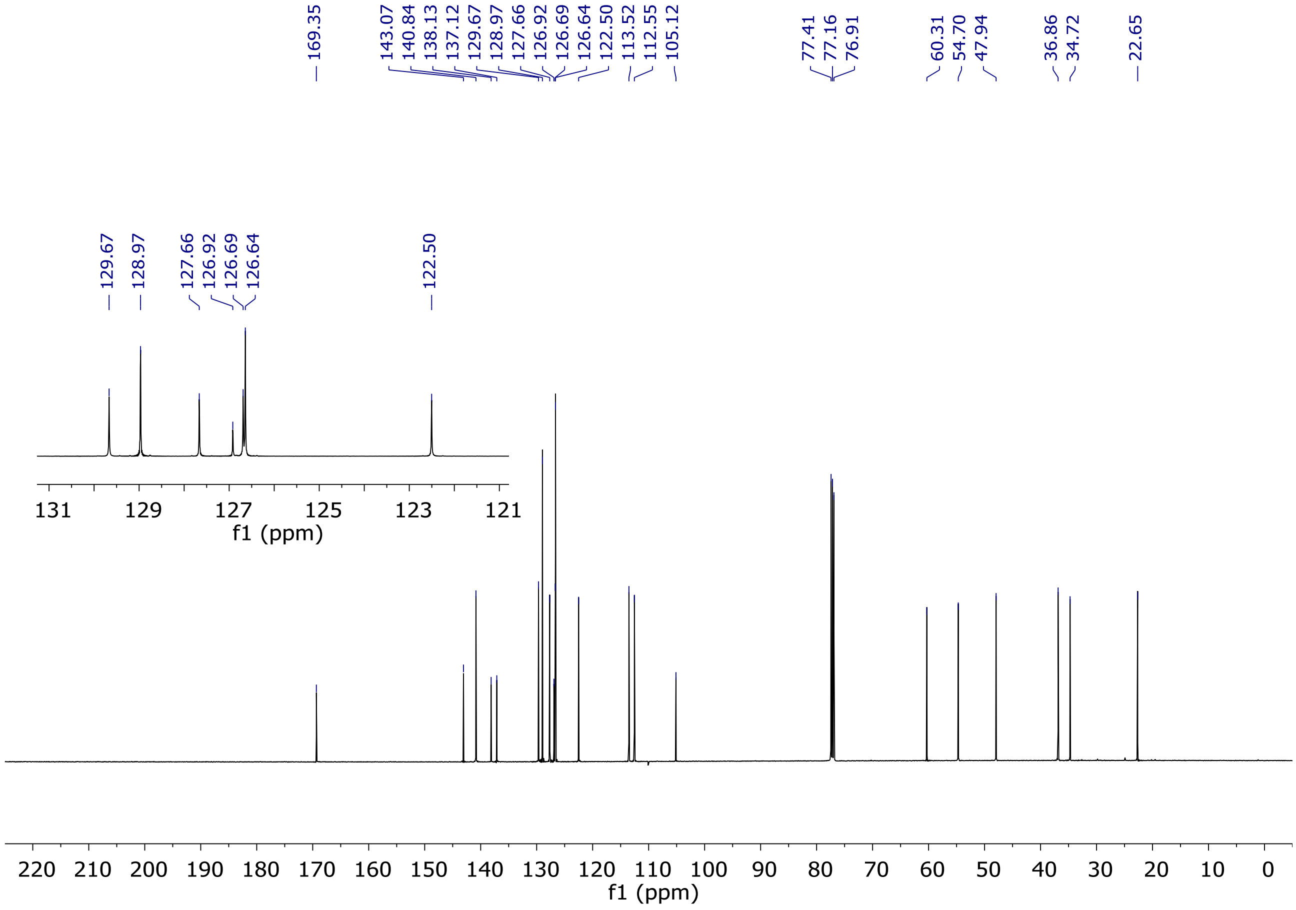
169.35
143.07
140.84
138.13
137.12
129.67
128.97
127.66
126.92
126.69
126.64
122.50
113.52
112.55
105.12
77.41
77.16
76.91
60.31
54.70
47.94
36.86
34.72
22.65
f1 (ppm)

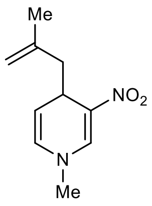
Me
NO2
N
Me

3ra

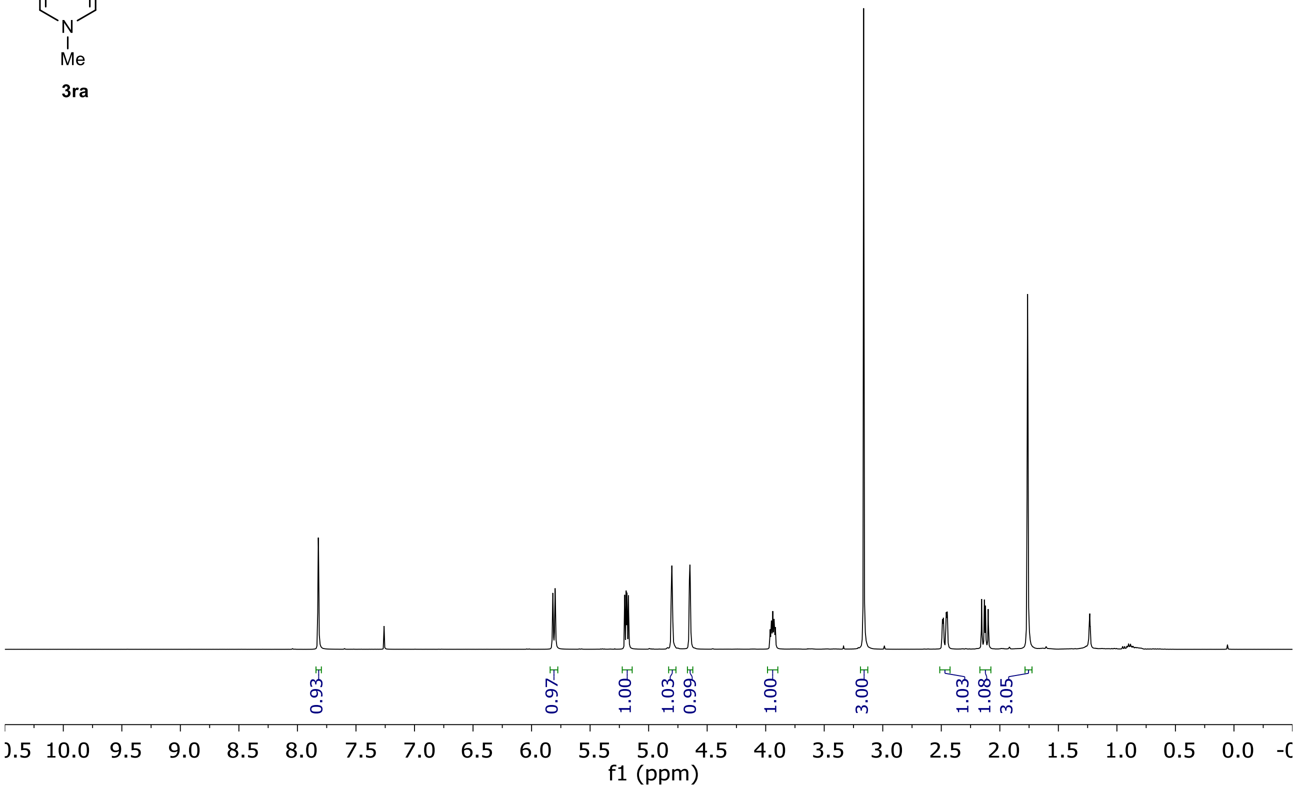
0.93
0.97
1.00
1.03
0.99
1.00
3.00
1.03
1.08
3.05
f1 (ppm)

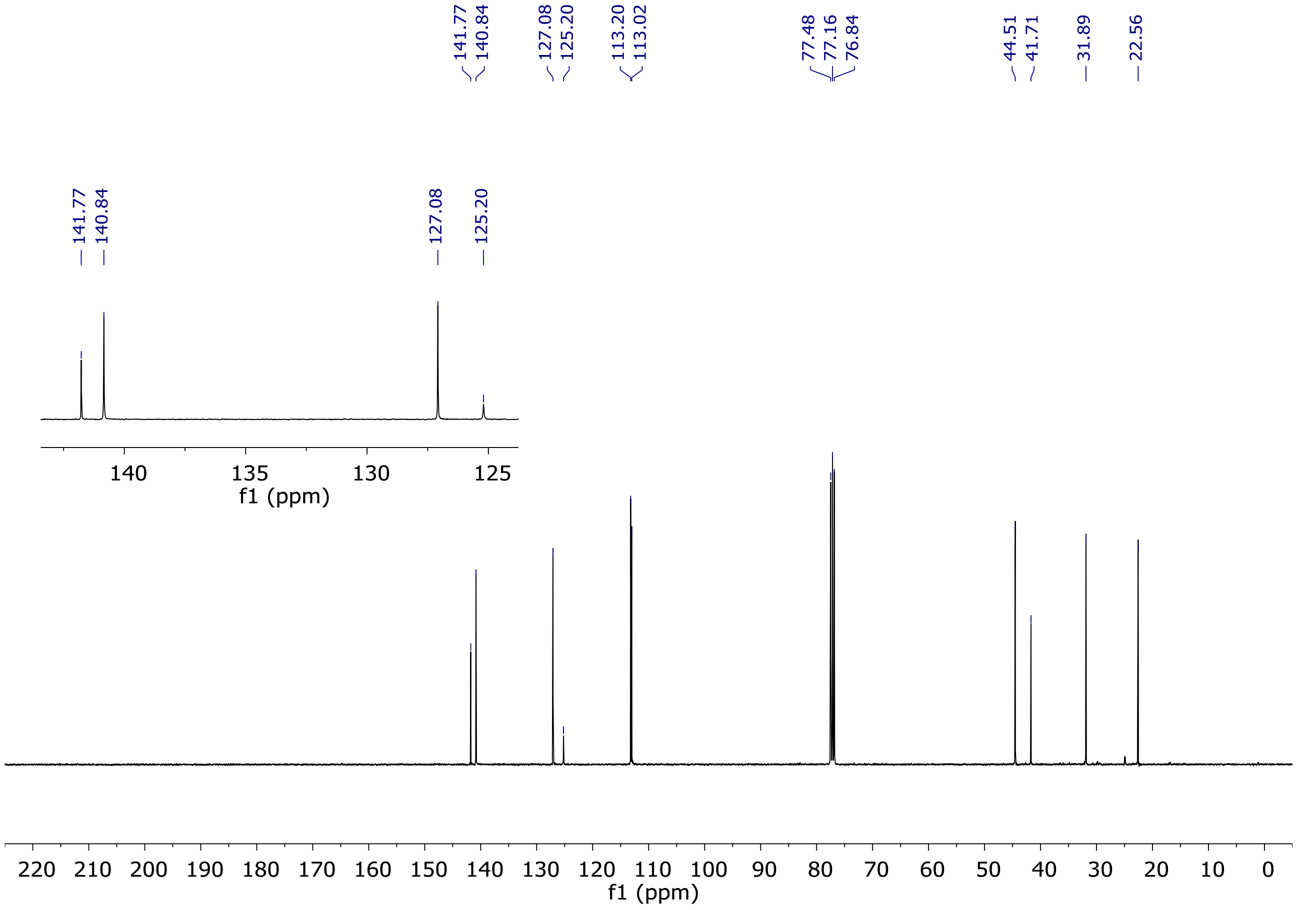
141.77
140.84
127.08
125.20
113.20
113.02
77.48
77.16
76.84
44.51
41.71
31.89
22.56
f1 (ppm)

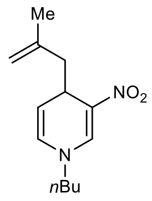
Me
NO2
N
nBu

3sa

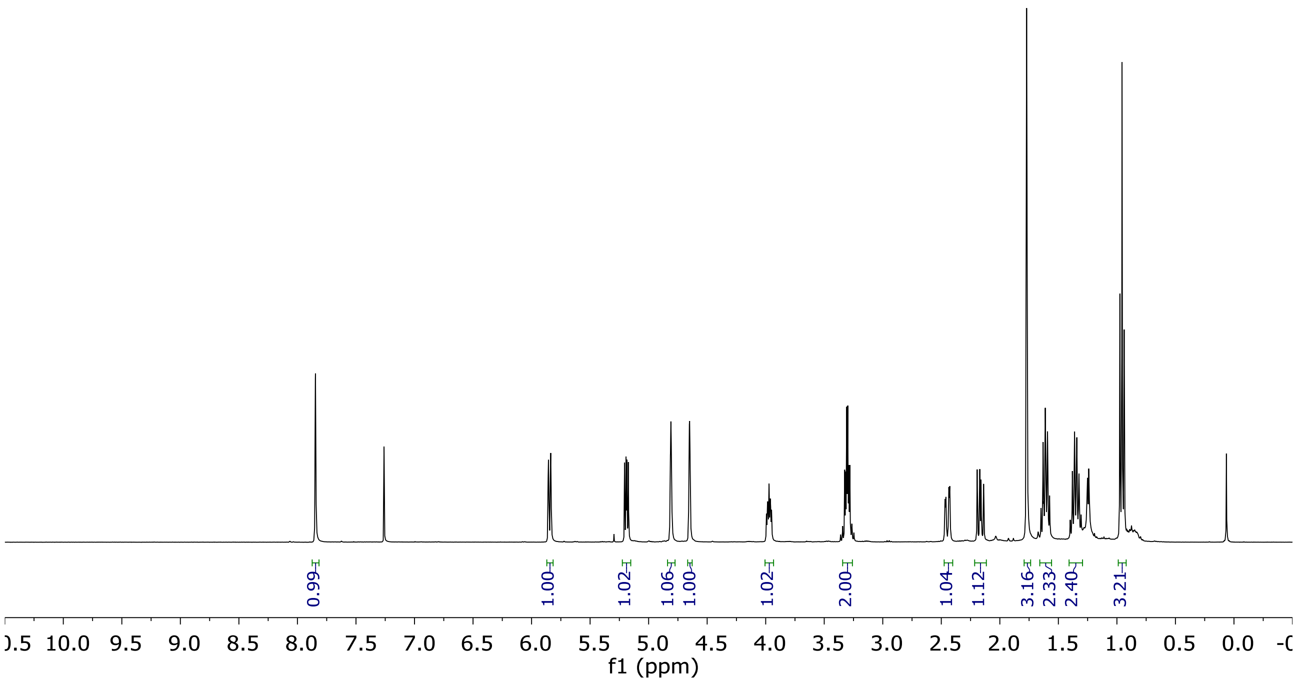
0.99
1.00
1.02
1.06
1.00
1.02
2.00
1.04
1.12
3.16
2.33
2.40
3.21
10.0 9.5 9.0 8.5 8.0 7.5 7.0 6.5 6.0 5.5 5.0 4.5 4.0 3.5 3.0 2.5 2.0 1.5 1.0 0.5 0.0
f1 (ppm)

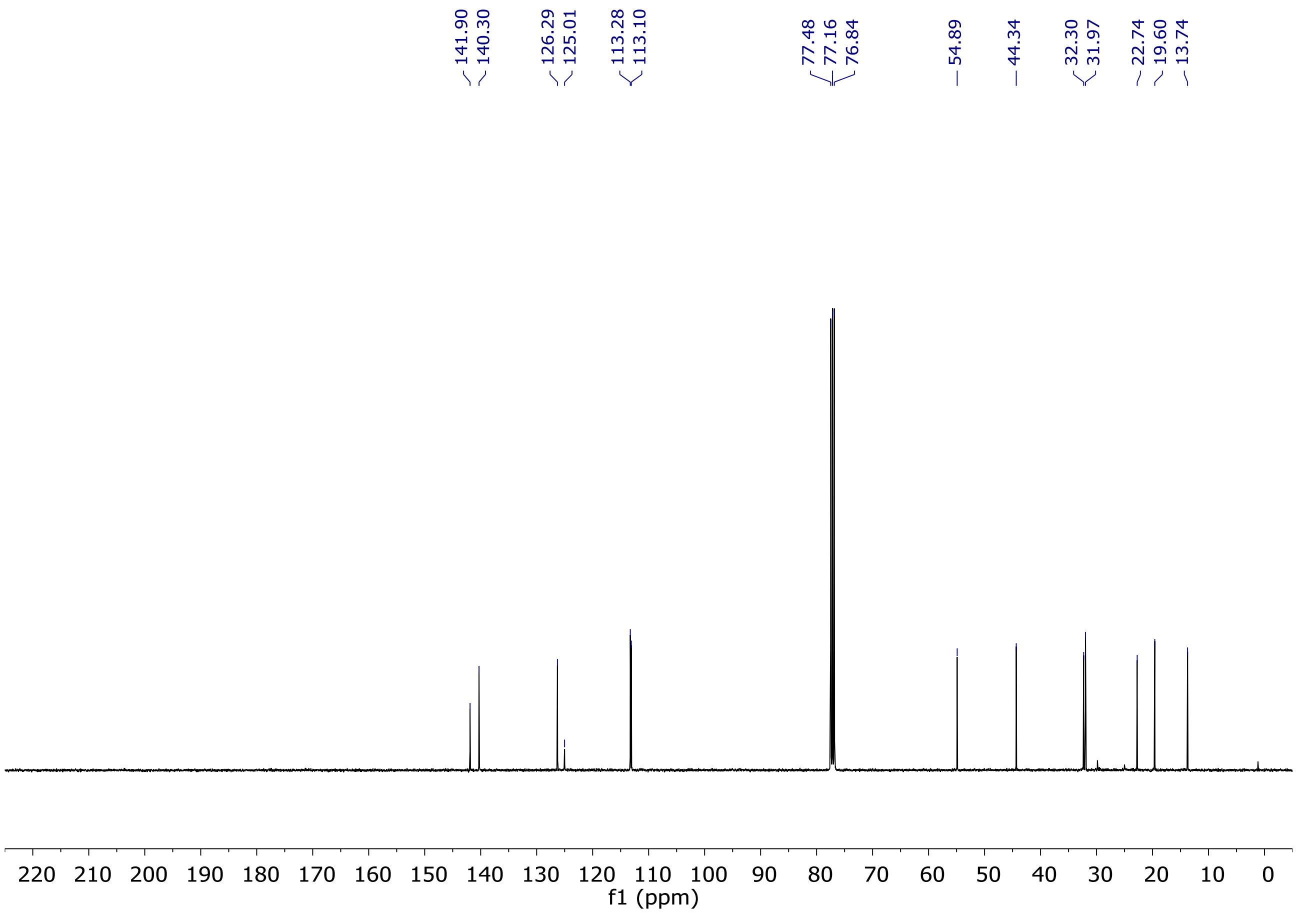
141.90
140.30
126.29
125.01
113.28
113.10
77.48
77.16
76.84
54.89
44.34
32.30
31.97
22.74
19.60
13.74
220 210 200 190 180 170 160 150 140 130 120 110 100 90 80 70 60 50 40 30 20 10 0
f1 (ppm)

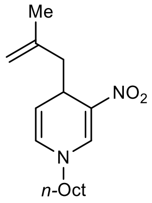
Me
NO2
N
n-Oct

3ta

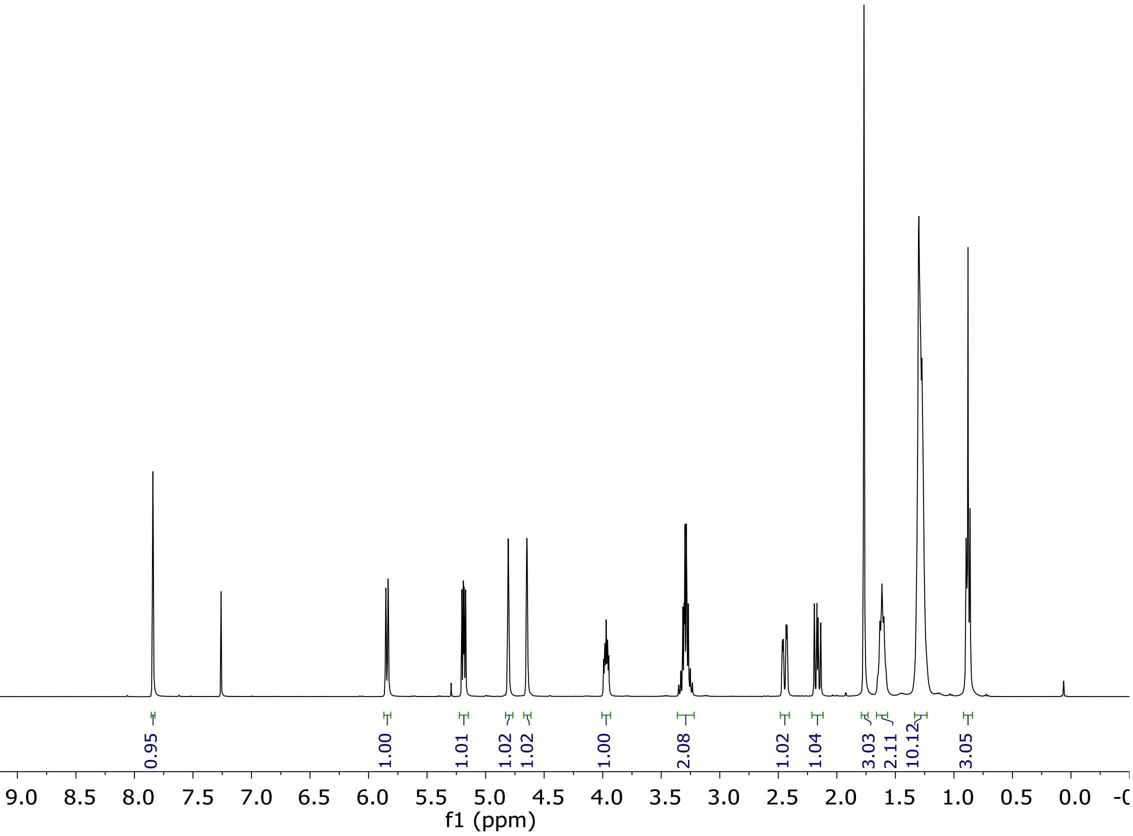
0.95
1.00
1.01
1.02
1.02
1.00
2.08
1.02
1.04
3.03
2.11
10.12
3.05
10.5 10.0 9.5 9.0 8.5 8.0 7.5 7.0 6.5 6.0 5.5 5.0 4.5 4.0 3.5 3.0 2.5 2.0 1.5 1.0 0.5 0.0
f1 (ppm)

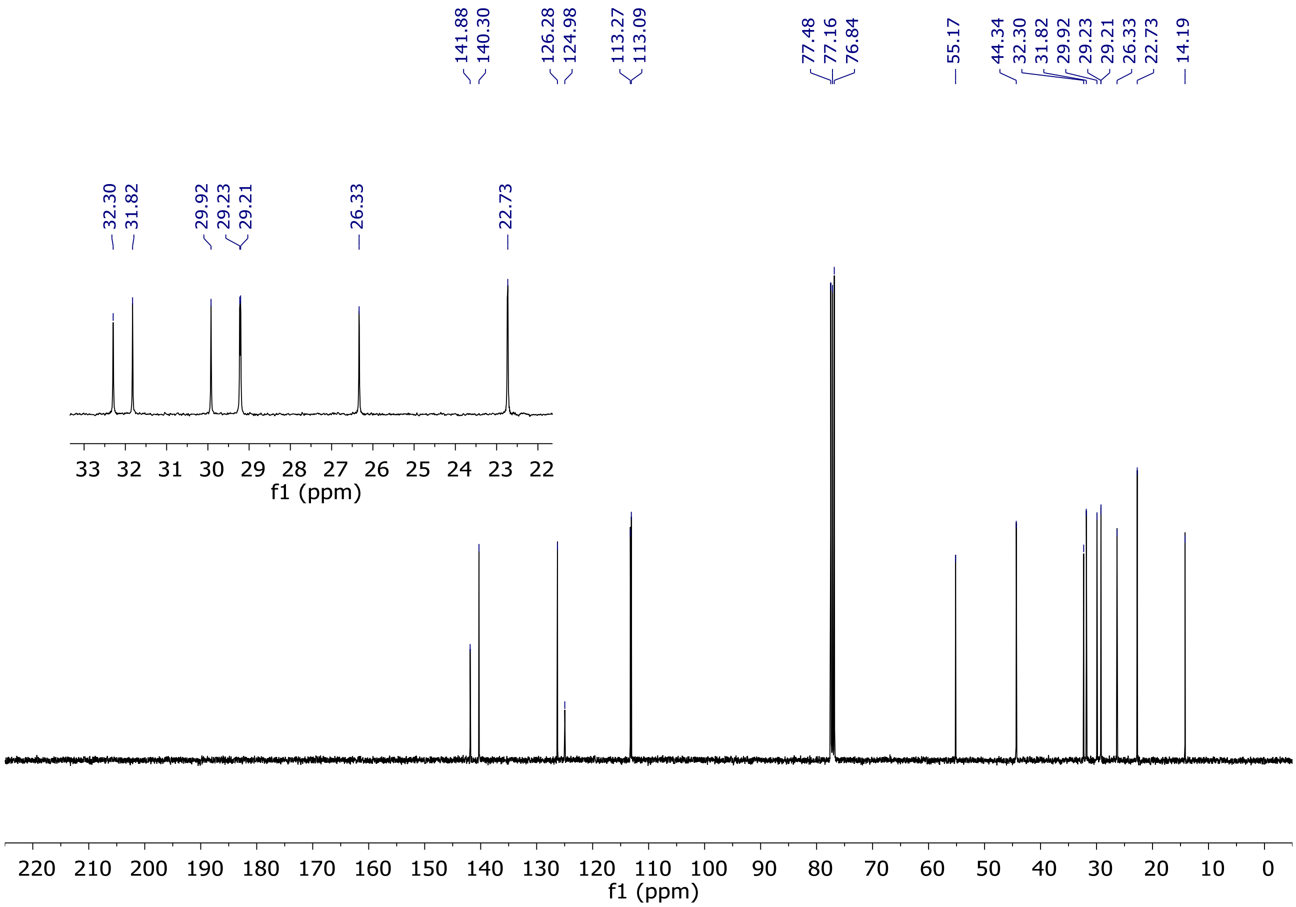
141.88
140.30
126.28
124.98
113.27
113.09
77.48
77.16
76.84
55.17
44.34
32.30
31.82
29.92
29.23
29.21
26.33
22.73
14.19
32.30
31.82
29.92
29.23
29.21
26.33
22.73
33 32 31 30 29 28 27 26 25 24 23 22
f1 (ppm)
220 210 200 190 180 170 160 150 140 130 120 110 100 90 80 70 60 50 40 30 20 10 0
f1 (ppm)

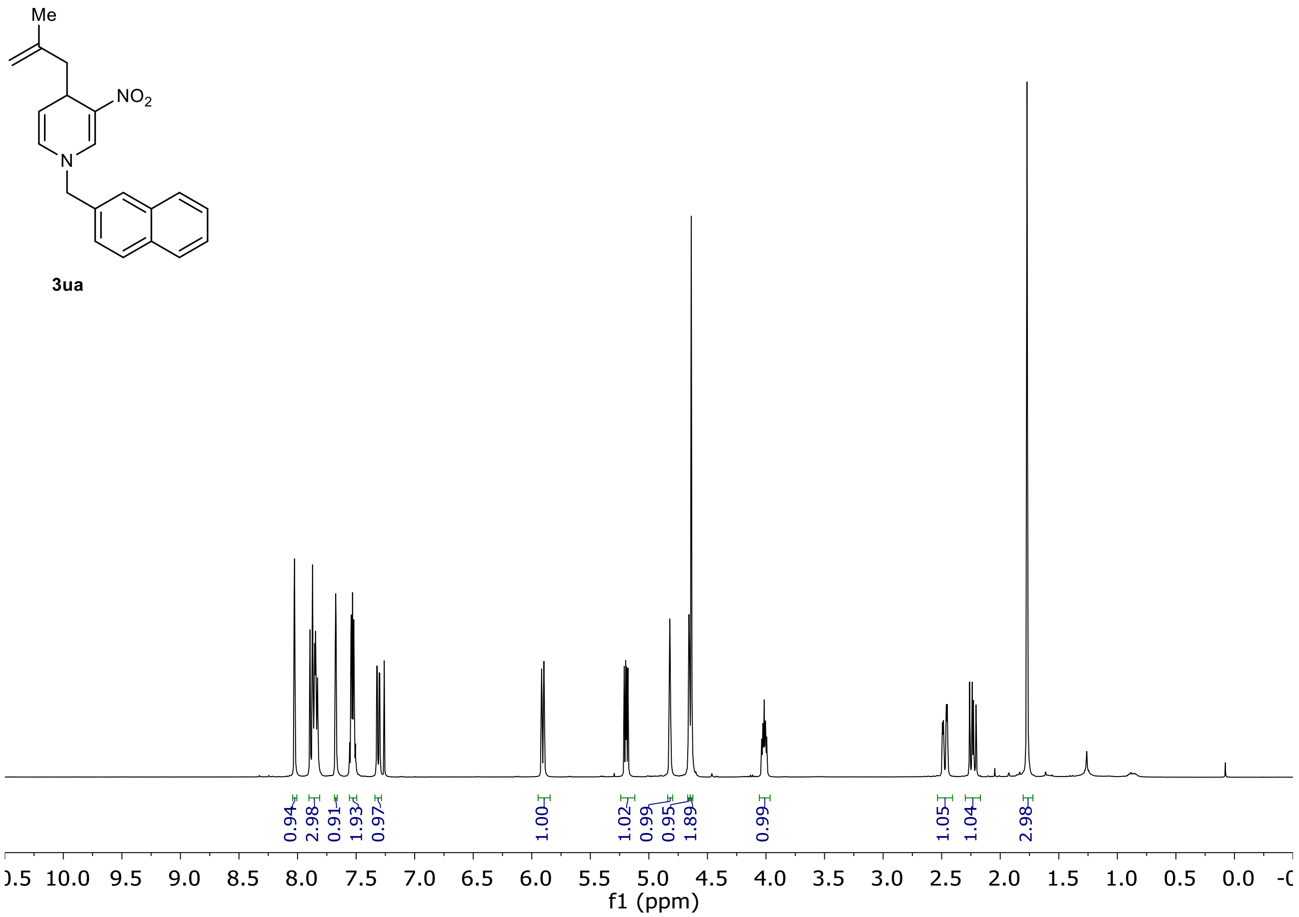
Me
NO2
N
3ua
0.94
2.98
0.91
1.93
0.97
1.00
1.02
0.99
0.95
1.89
0.99
1.05
1.04
2.98
10.0 9.5 9.0 8.5 8.0 7.5 7.0 6.5 6.0 5.5 5.0 4.5 4.0 3.5 3.0 2.5 2.0 1.5 1.0 0.5 0.0
f1 (ppm)

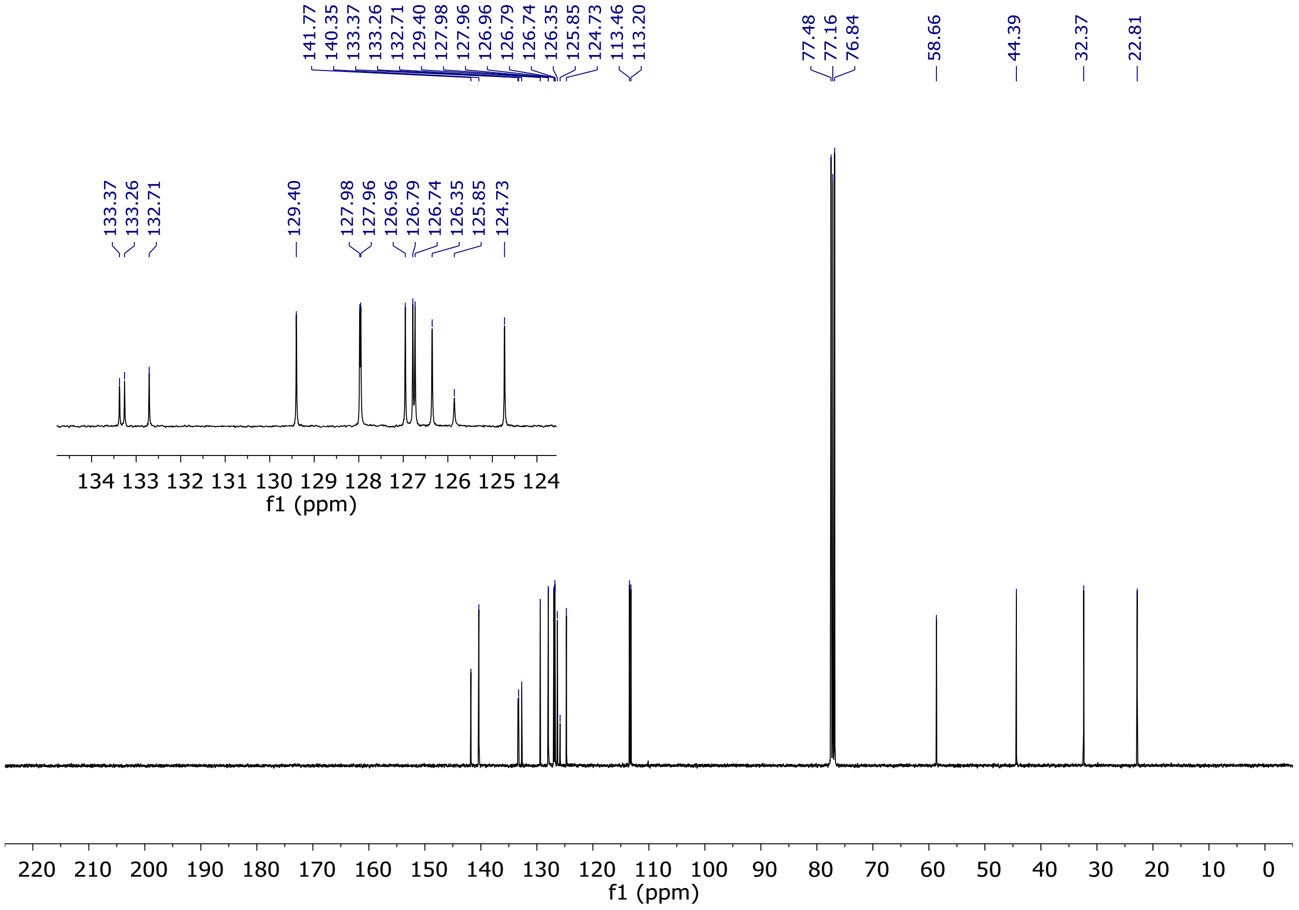
141.77
140.35
133.37
133.26
132.71
129.40
127.98
127.96
126.96
126.79
126.74
126.35
125.85
124.73
113.46
113.20
77.48
77.16
76.84
58.66
44.39
32.37
22.81
134 133 132 131 130 129 128 127 126 125 124
f1 (ppm)
220 210 200 190 180 170 160 150 140 130 120 110 100 90 80 70 60 50 40 30 20 10 0
f1 (ppm)

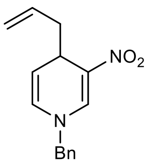
NO2
N
Bn

3ab

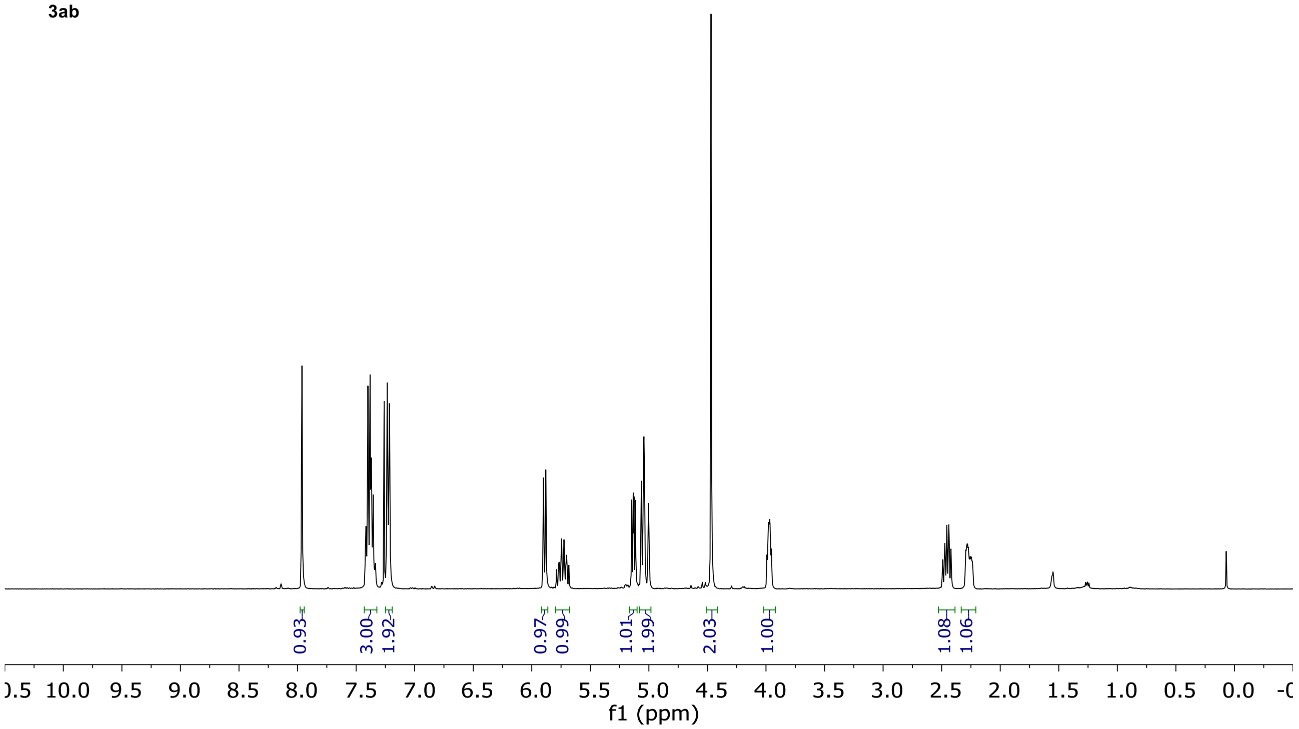
0.93
3.00
1.92
0.97
0.99
1.01
1.99
2.03
1.00
1.08
1.06
f1 (ppm)

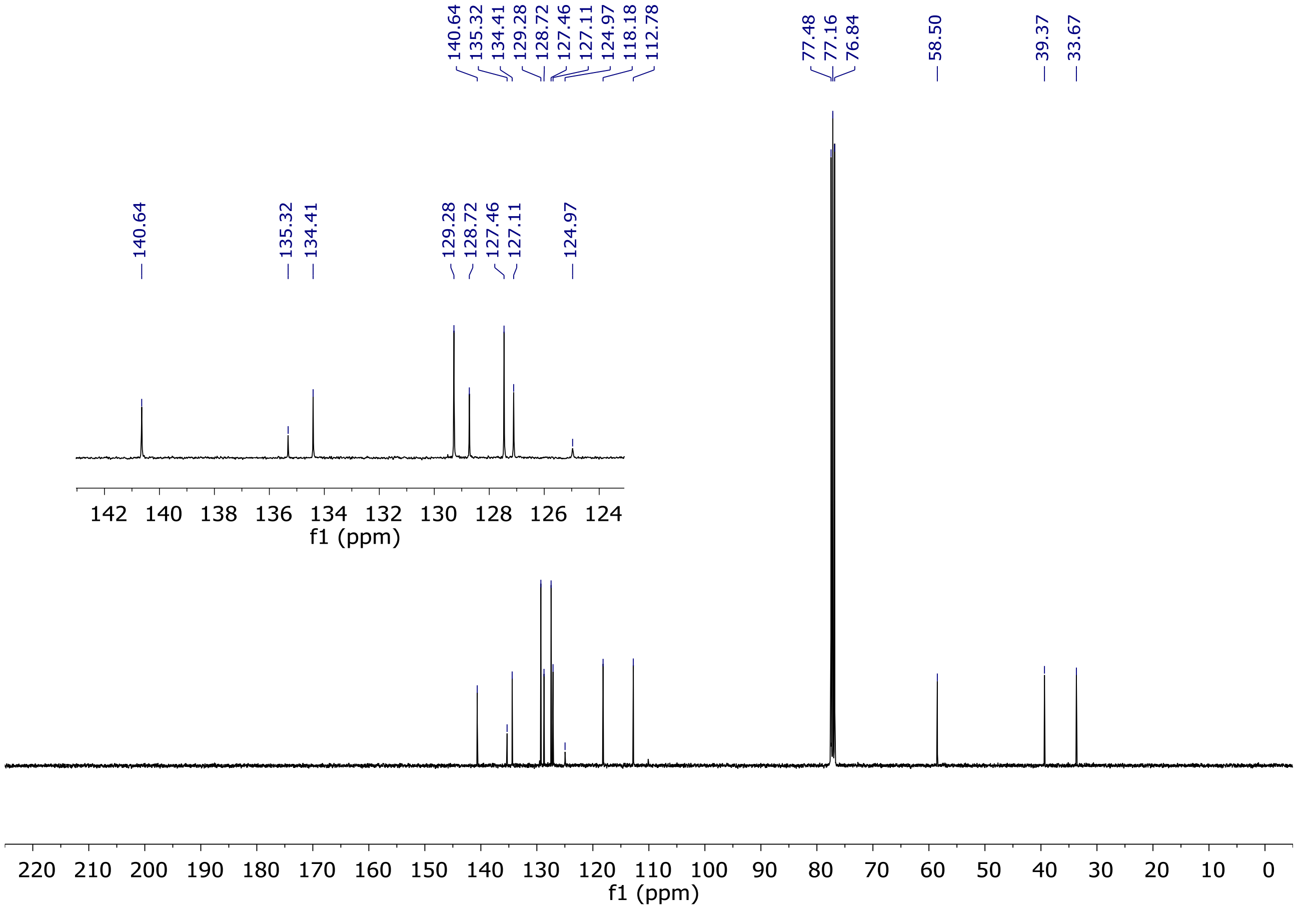
140.64
135.32
134.41
129.28
128.72
127.46
127.11
124.97
118.18
112.78
77.48
77.16
76.84
58.50
39.37
33.67
f1 (ppm)

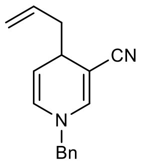
CN
N
Bn

3bb

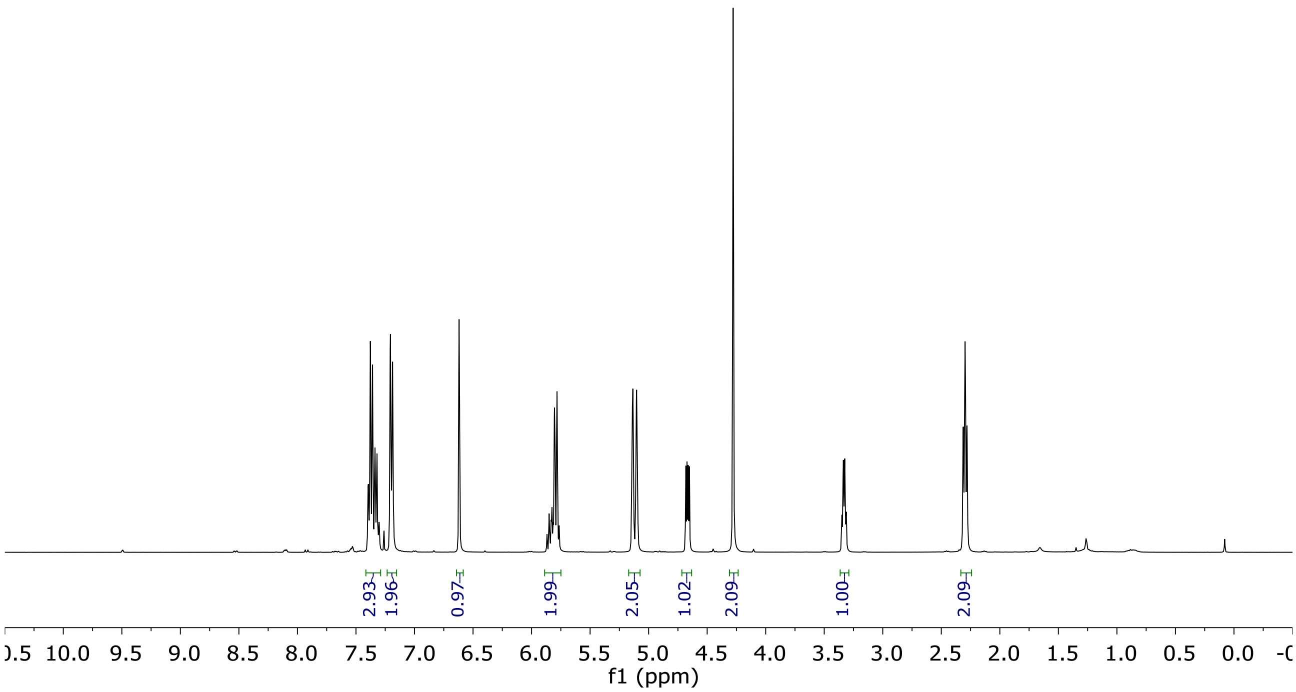
2.93
1.96
0.97
1.99
2.05
1.02
2.09
1.00
2.09
f1 (ppm)

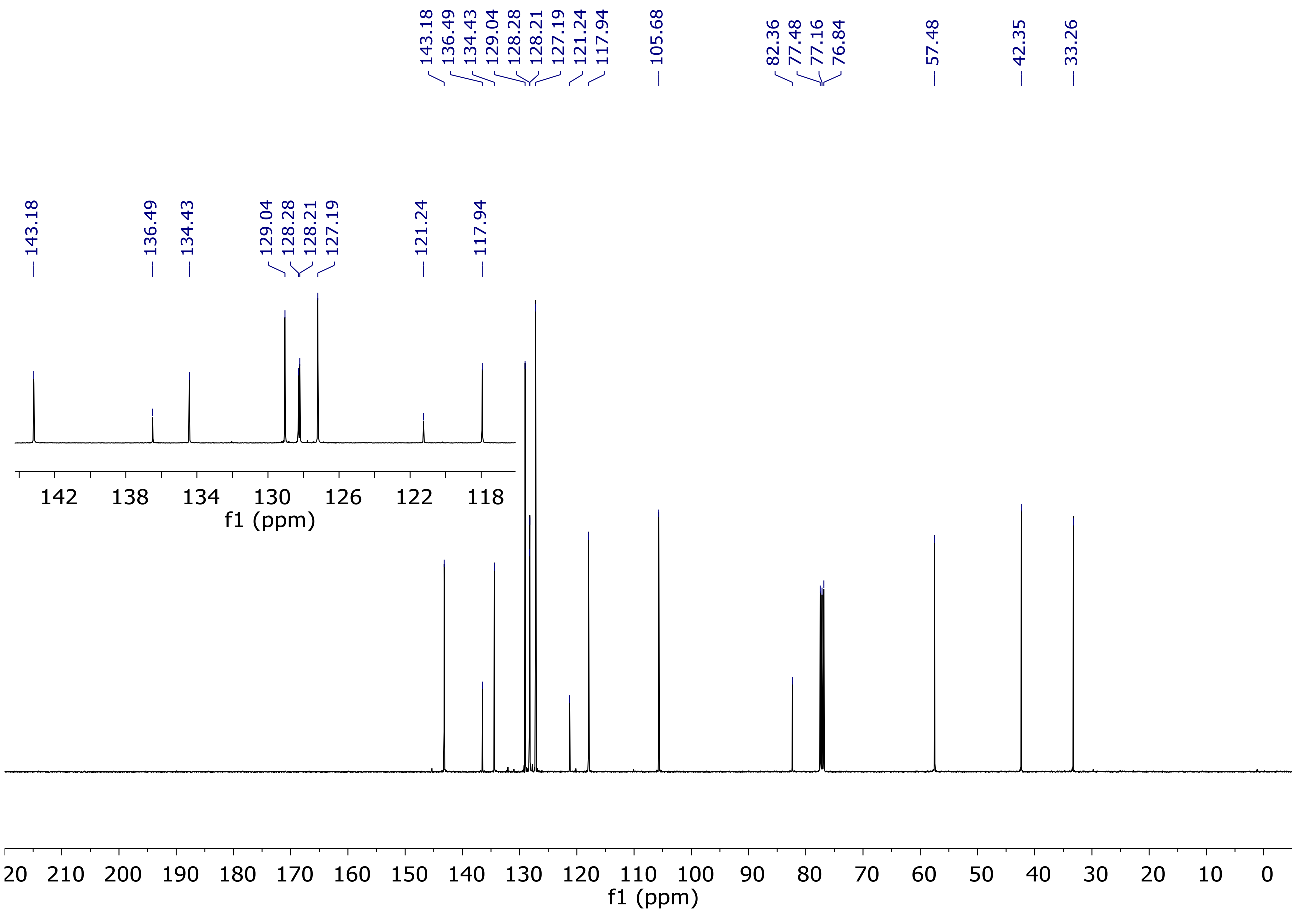
143.18
136.49
134.43
129.04
128.28
128.21
127.19
121.24
117.94
105.68
82.36
77.48
77.16
76.84
57.48
42.35
33.26
f1 (ppm)

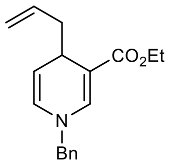
CO2Et
N
Bn

3eb

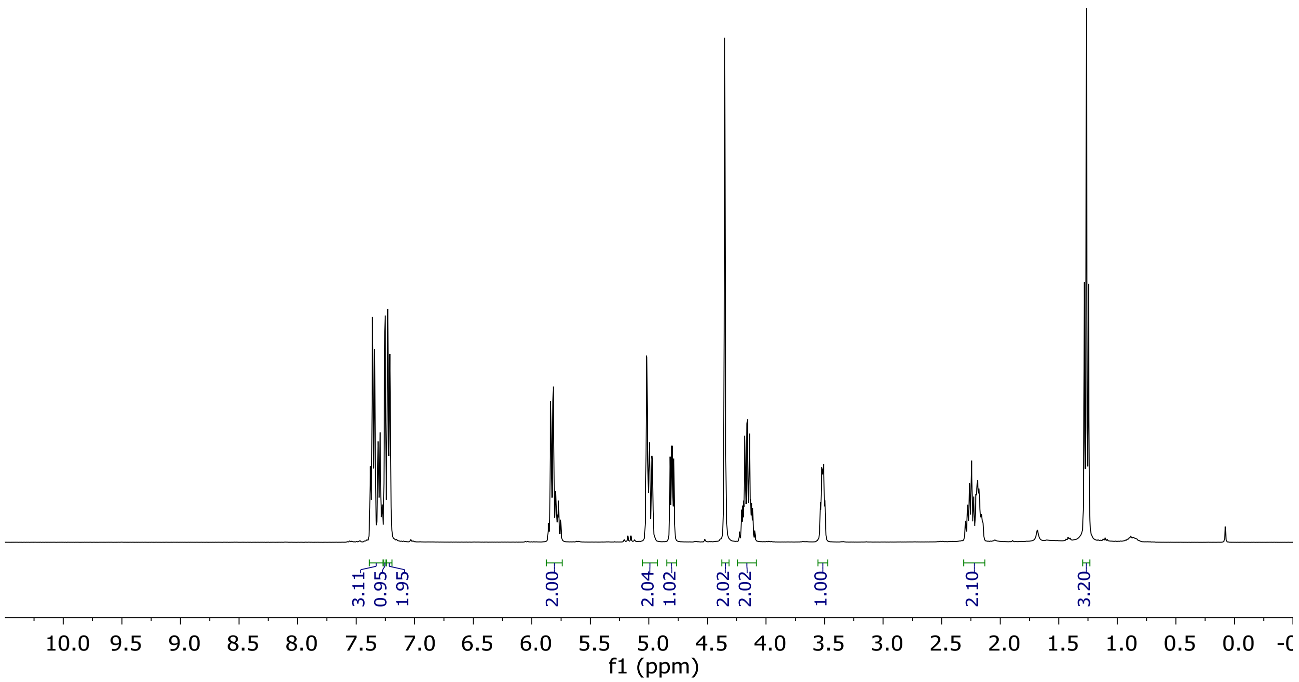
3.11
0.95
1.95
2.00
2.04
1.02
2.02
2.02
1.00
2.10
3.20
10.0 9.5 9.0 8.5 8.0 7.5 7.0 6.5 6.0 5.5 5.0 4.5 4.0 3.5 3.0 2.5 2.0 1.5 1.0 0.5 0.0
f1 (ppm)

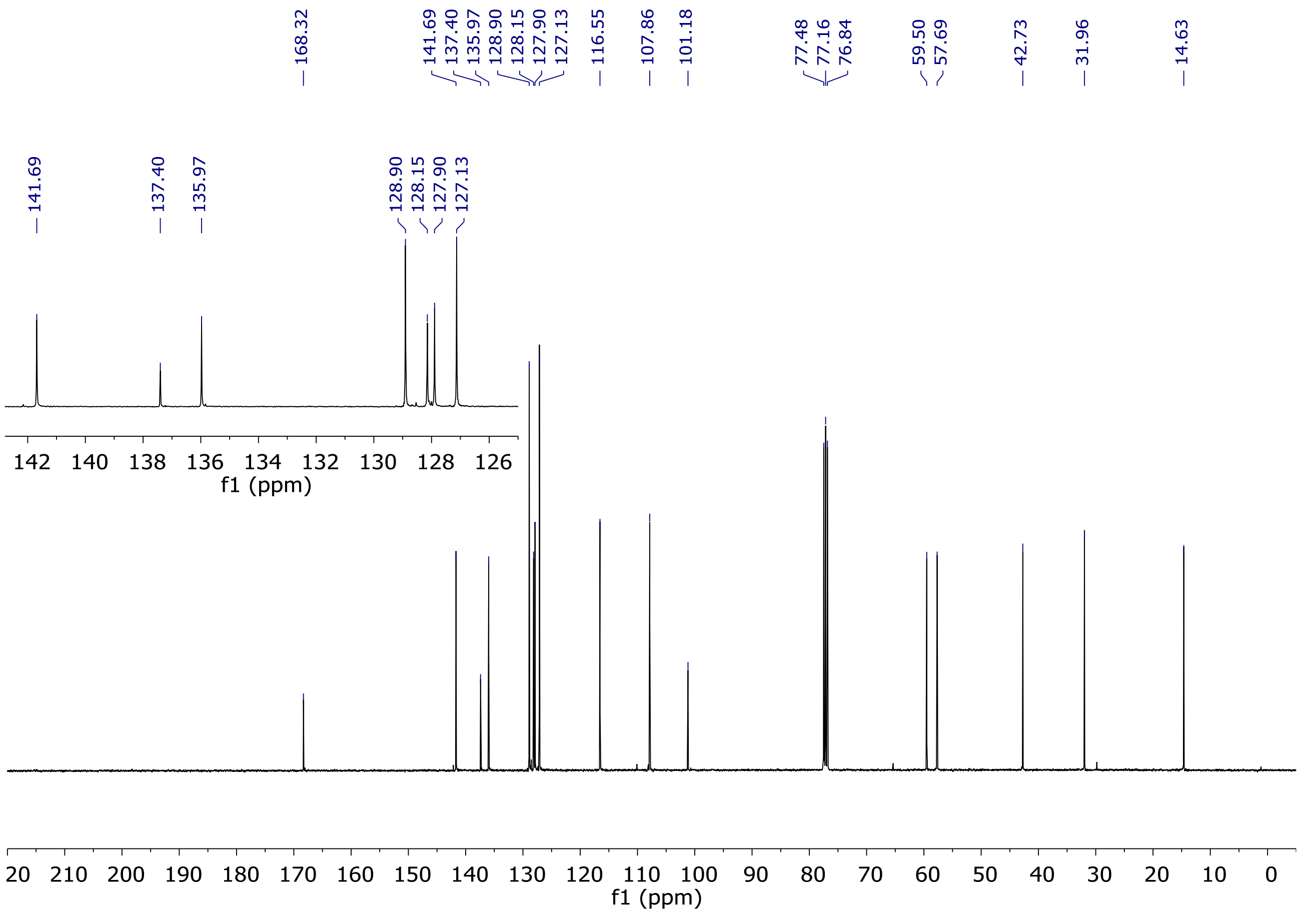
168.32
141.69
137.40
135.97
128.90
128.15
127.90
127.13
116.55
107.86
101.18
77.48
77.16
76.84
59.50
57.69
42.73
31.96
14.63
142 140 138 136 134 132 130 128 126
f1 (ppm)
210 200 190 180 170 160 150 140 130 120 110 100 90 80 70 60 50 40 30 20 10 0
f1 (ppm)

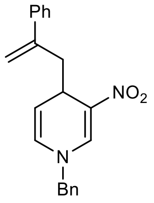
Ph
NO2
N
Bn

3ac

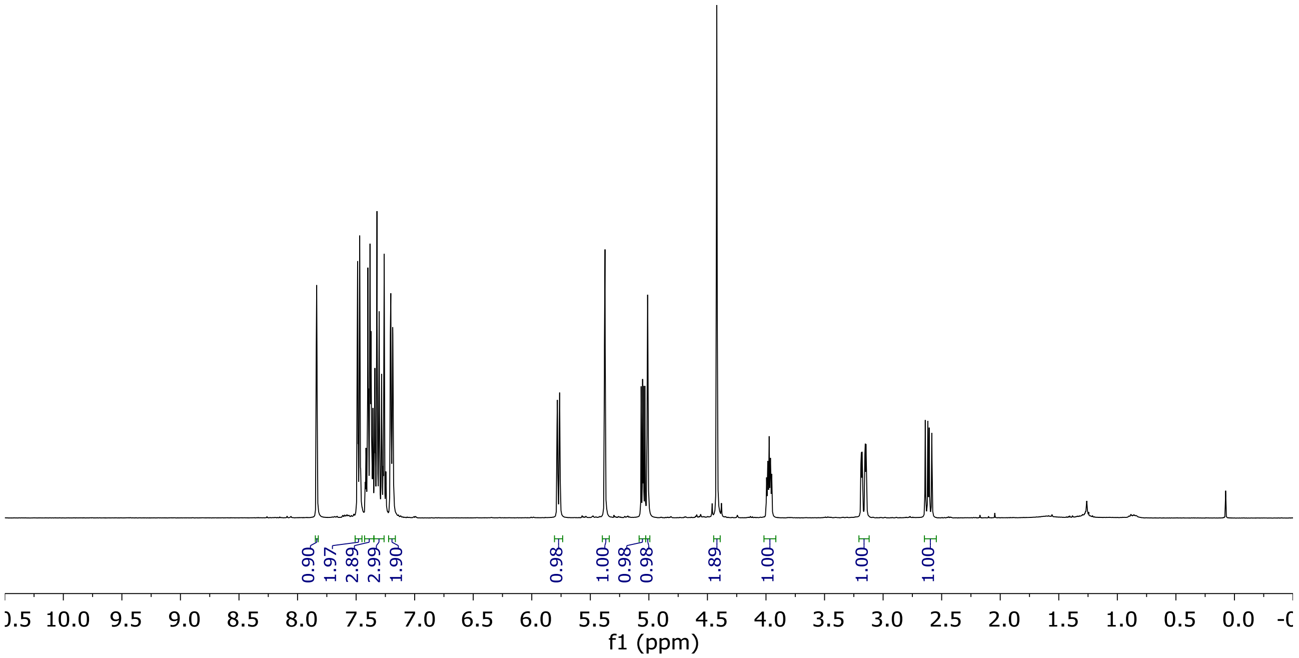
0.90
1.97
2.89
2.99
1.90
0.98
1.00
0.98
0.98
1.89
1.00
1.00
1.00
10.0 9.5 9.0 8.5 8.0 7.5 7.0 6.5 6.0 5.5 5.0 4.5 4.0 3.5 3.0 2.5 2.0 1.5 1.0 0.5 0.0
f1 (ppm)

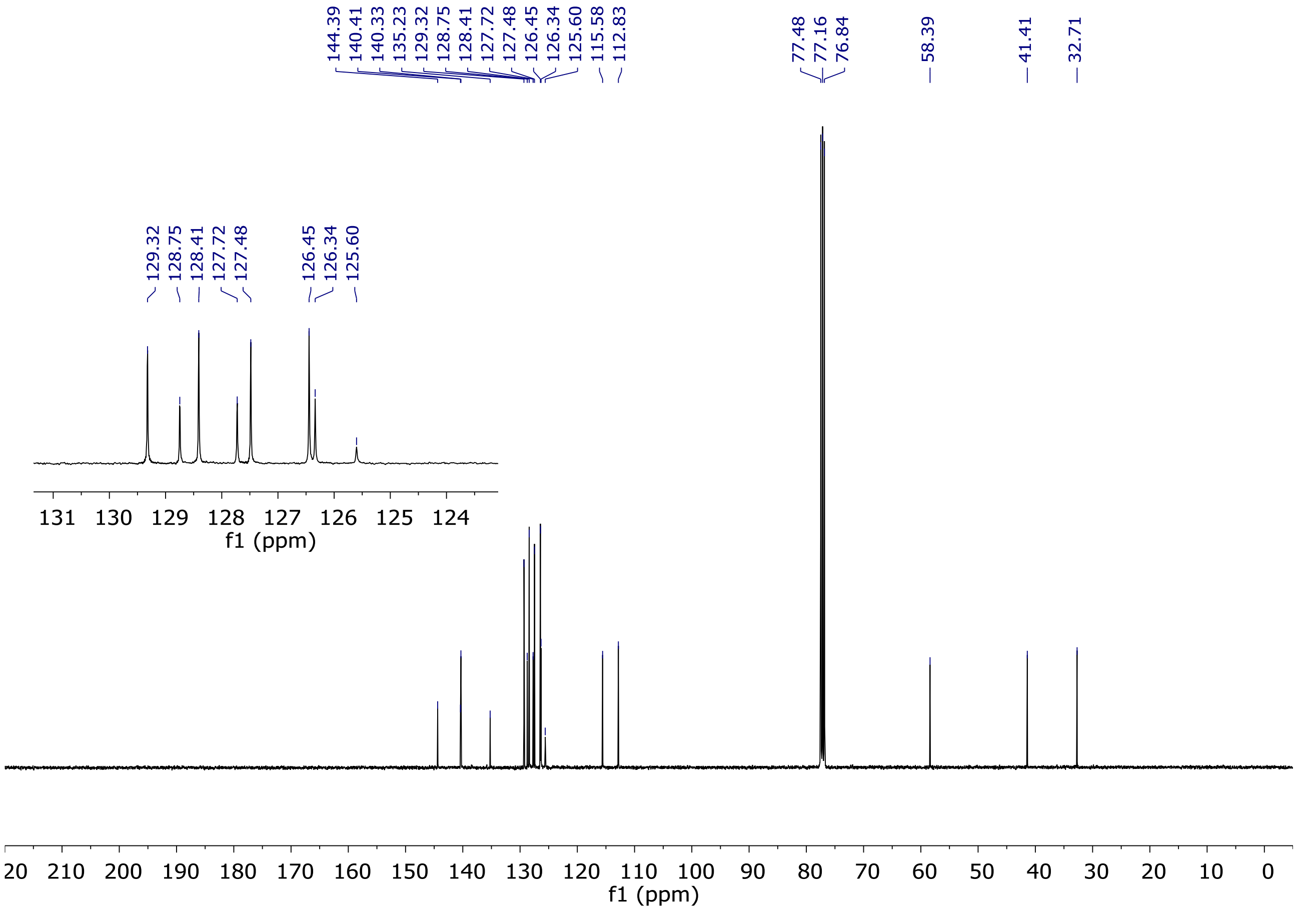
144.39
140.41
140.33
135.23
129.32
128.75
128.41
127.72
127.48
126.45
126.34
125.60
115.58
112.83
77.48
77.16
76.84
58.39
41.41
32.71
129.32
128.75
128.41
127.72
127.48
126.45
126.34
125.60
131 130 129 128 127 126 125 124
f1 (ppm)
210 200 190 180 170 160 150 140 130 120 110 100 90 80 70 60 50 40 30 20 10 0
f1 (ppm)

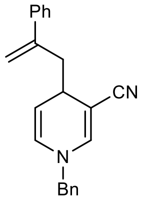
Ph
CN
N
Bn

3bc

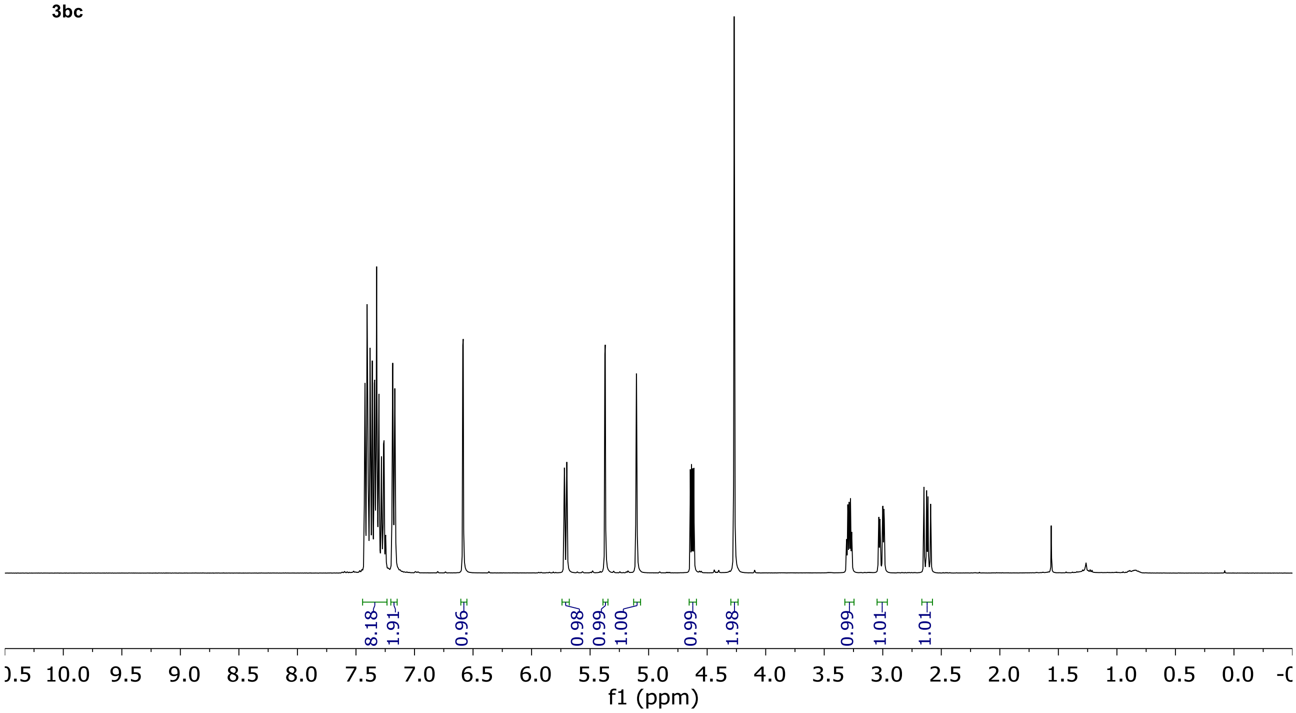
8.18
1.91
0.96
0.98
0.99
1.00
0.99
1.98
0.99
1.01
1.01
10.0 9.5 9.0 8.5 8.0 7.5 7.0 6.5 6.0 5.5 5.0 4.5 4.0 3.5 3.0 2.5 2.0 1.5 1.0 0.5 0.0
f1 (ppm)

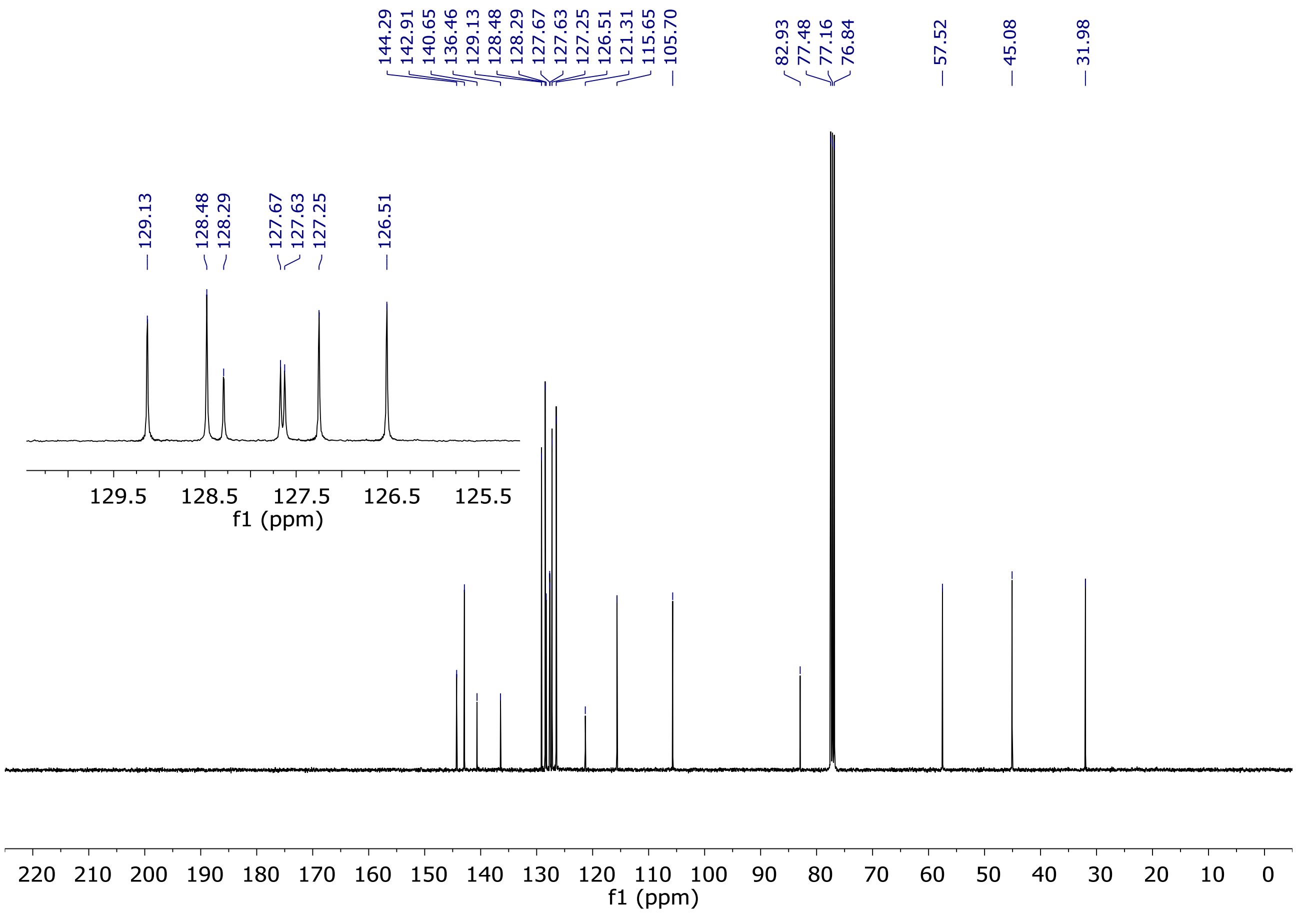
144.29
142.91
140.65
136.46
129.13
128.48
128.29
127.67
127.63
127.25
126.51
121.31
115.65
105.70
82.93
77.48
77.16
76.84
57.52
45.08
31.98
129.13
128.48
128.29
127.67
127.63
127.25
126.51
129.5 128.5 127.5 126.5 125.5
f1 (ppm)
220 210 200 190 180 170 160 150 140 130 120 110 100 90 80 70 60 50 40 30 20 10 0
f1 (ppm)

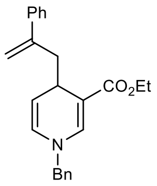
Ph
CO2Et
N
Bn

3ec

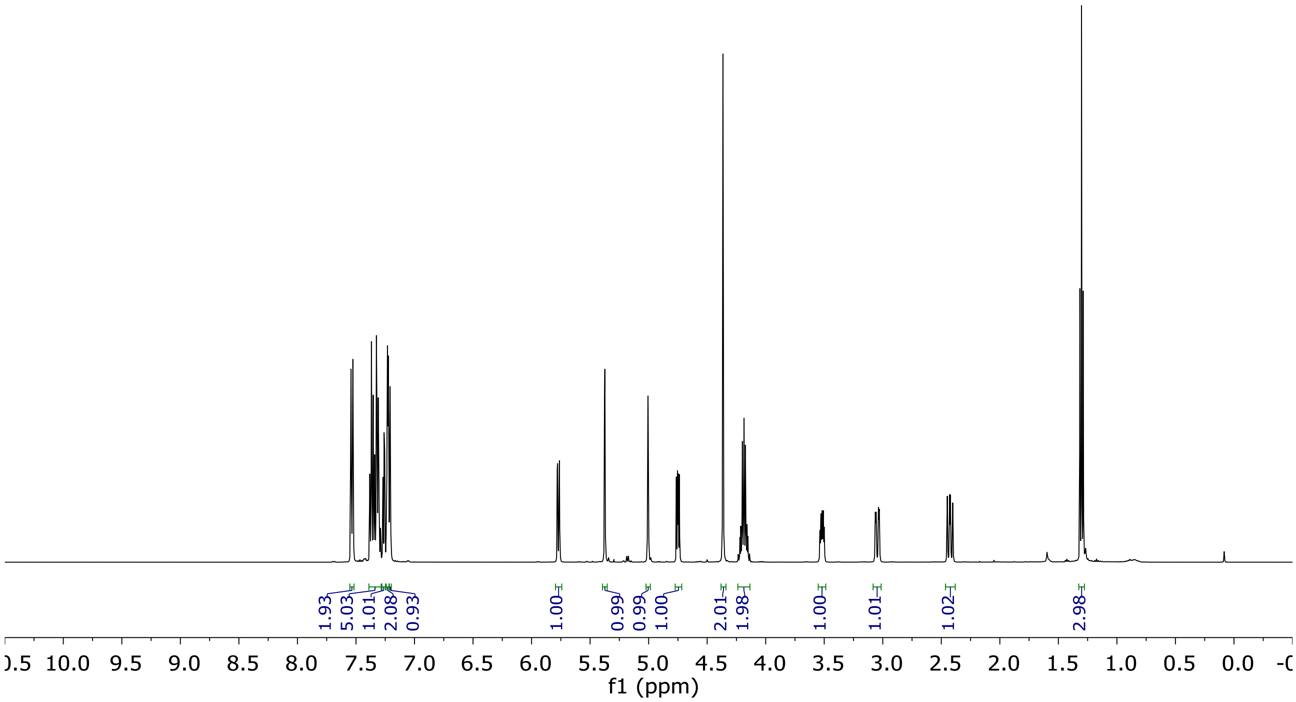
1.93
5.03
1.01
2.08
0.93
1.00
0.99
0.99
1.00
2.01
1.98
1.00
1.01
1.02
2.98
f1 (ppm)

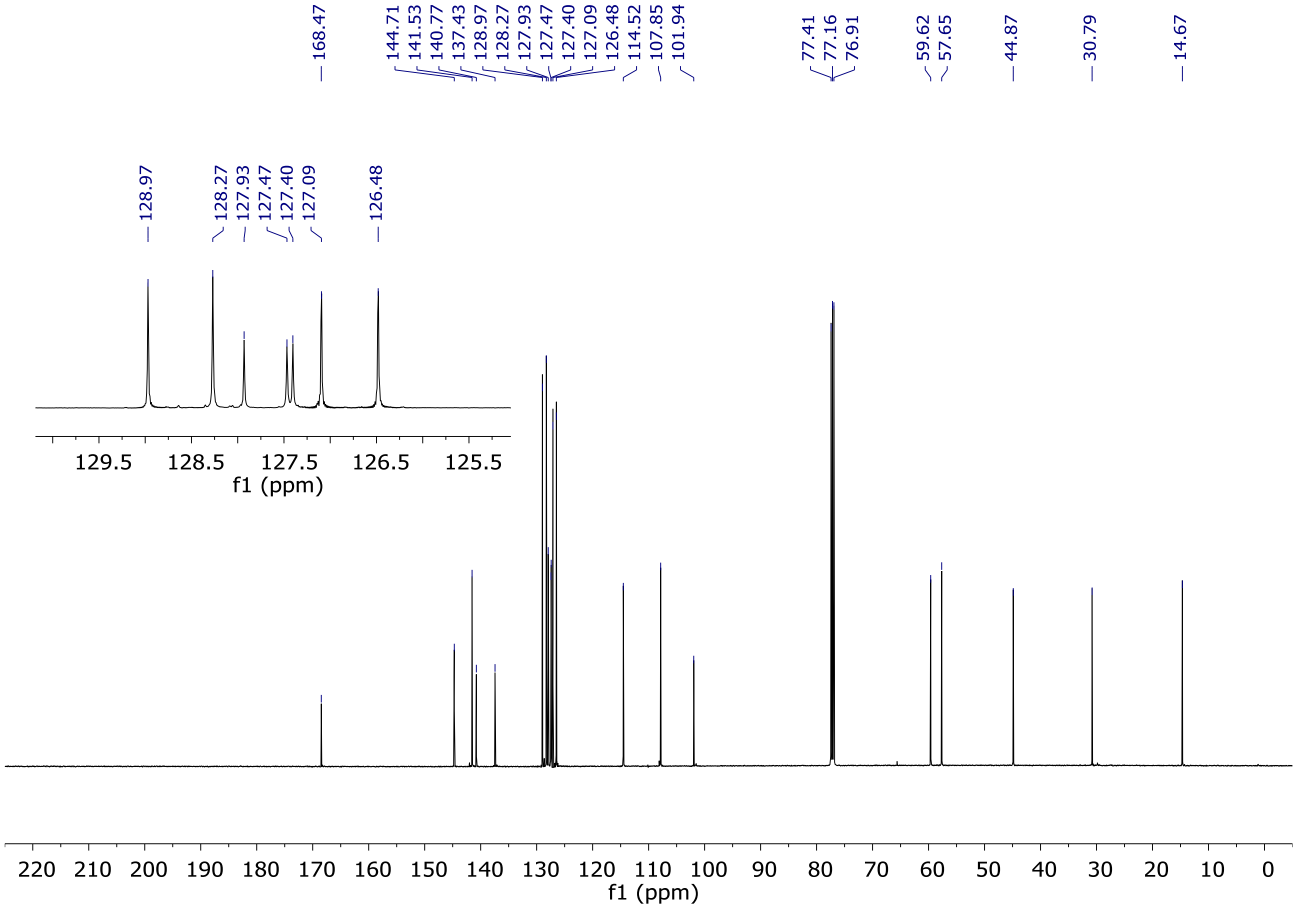
168.47
144.71
141.53
140.77
137.43
128.97
128.27
127.93
127.47
127.40
127.09
126.48
114.52
107.85
101.94
77.41
77.16
76.91
59.62
57.65
44.87
30.79
14.67
f1 (ppm)

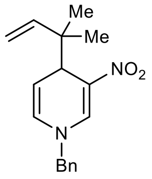
Me
Me
NO_2
N
Bn

3ad

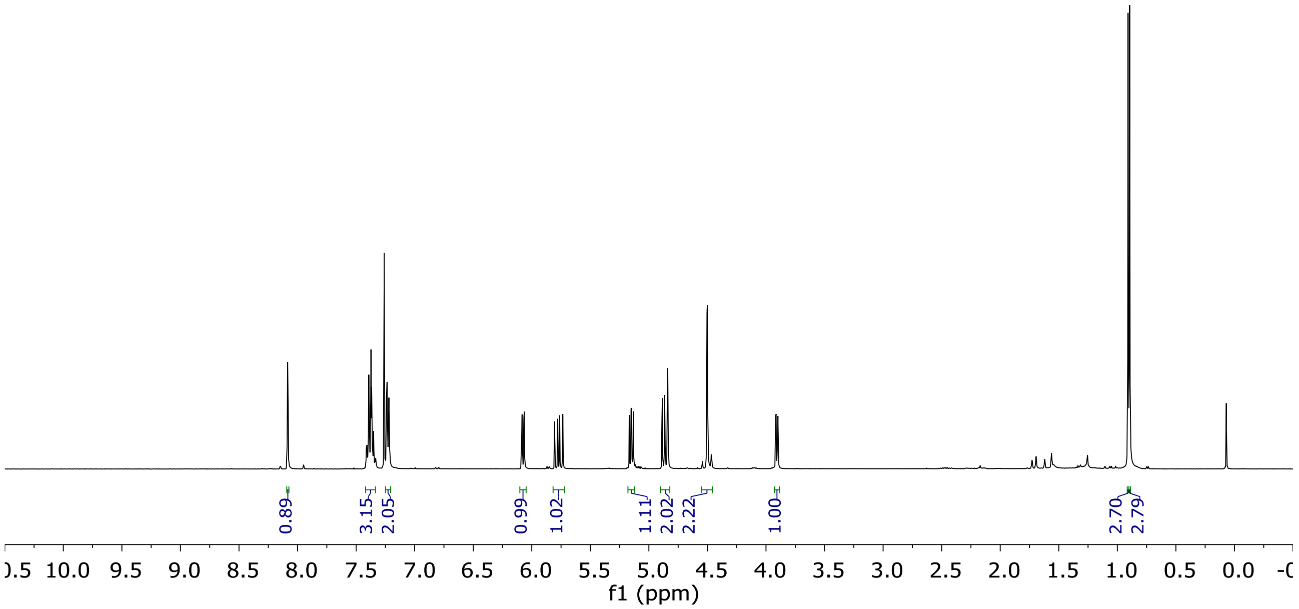
0.89
3.15
2.05
0.99
1.02
1.11
2.02
2.22
1.00
2.70
2.79
f1 (ppm)

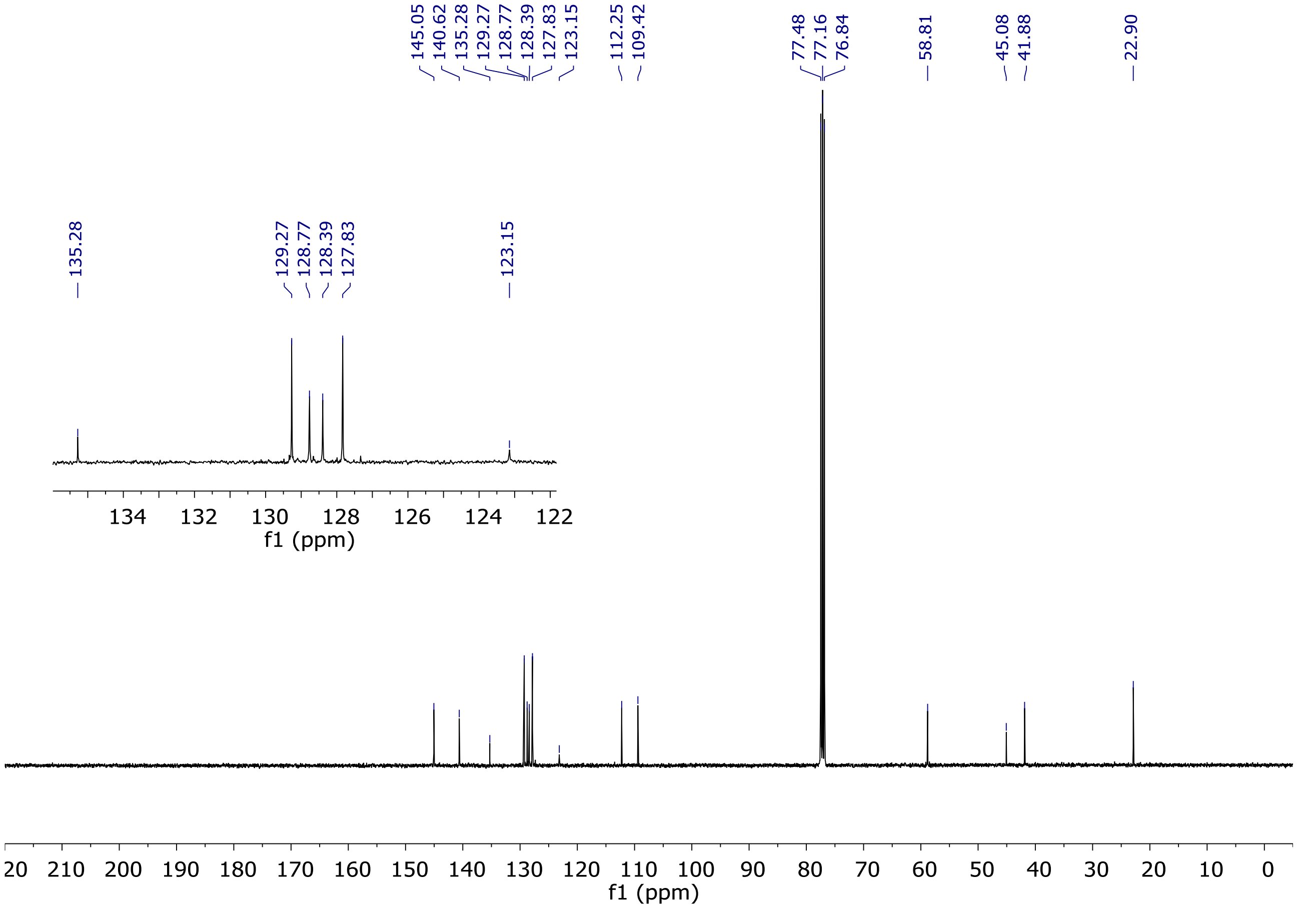
145.05
140.62
135.28
129.27
128.77
128.39
127.83
123.15
112.25
109.42
77.48
77.16
76.84
58.81
45.08
41.88
22.90
135.28
129.27
128.77
128.39
127.83
123.15
f1 (ppm)
f1 (ppm)

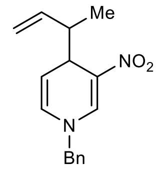

3ae
2:1 mixture of
diastereoisomers

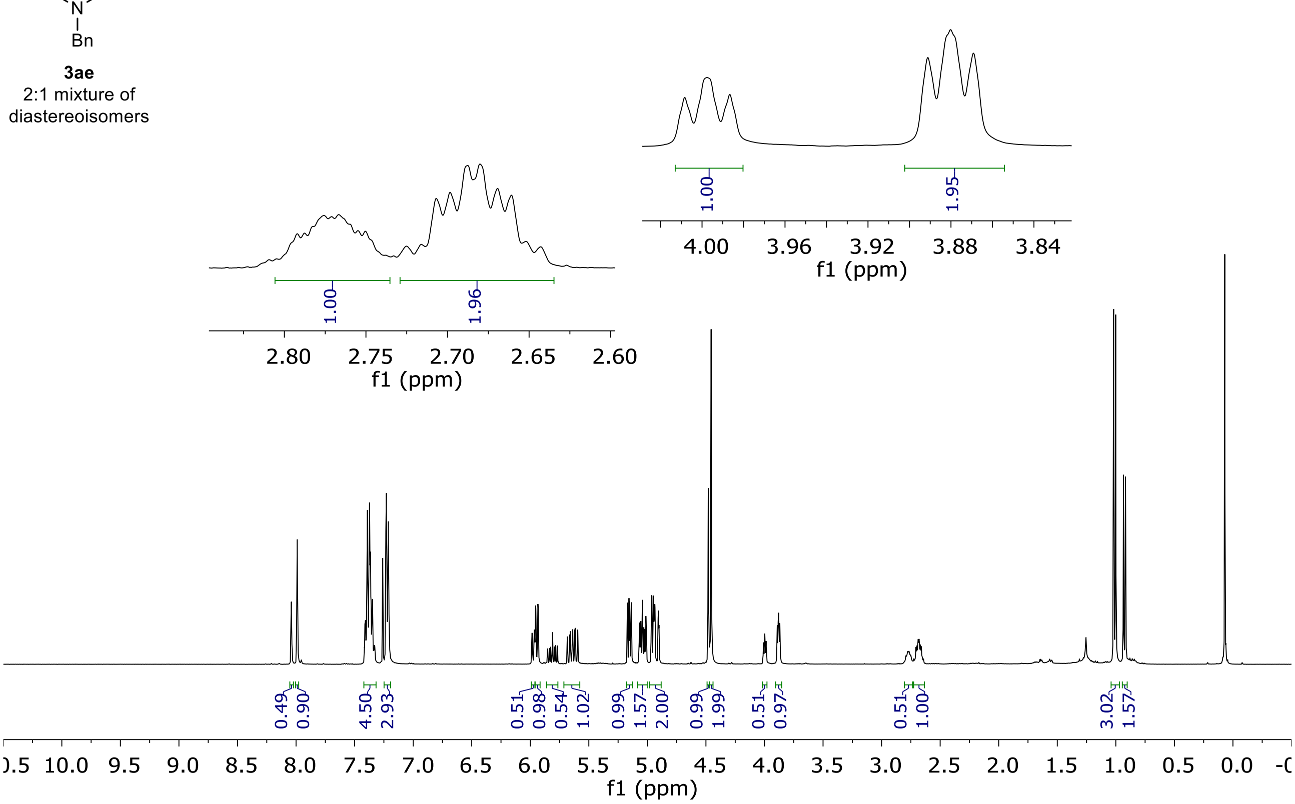

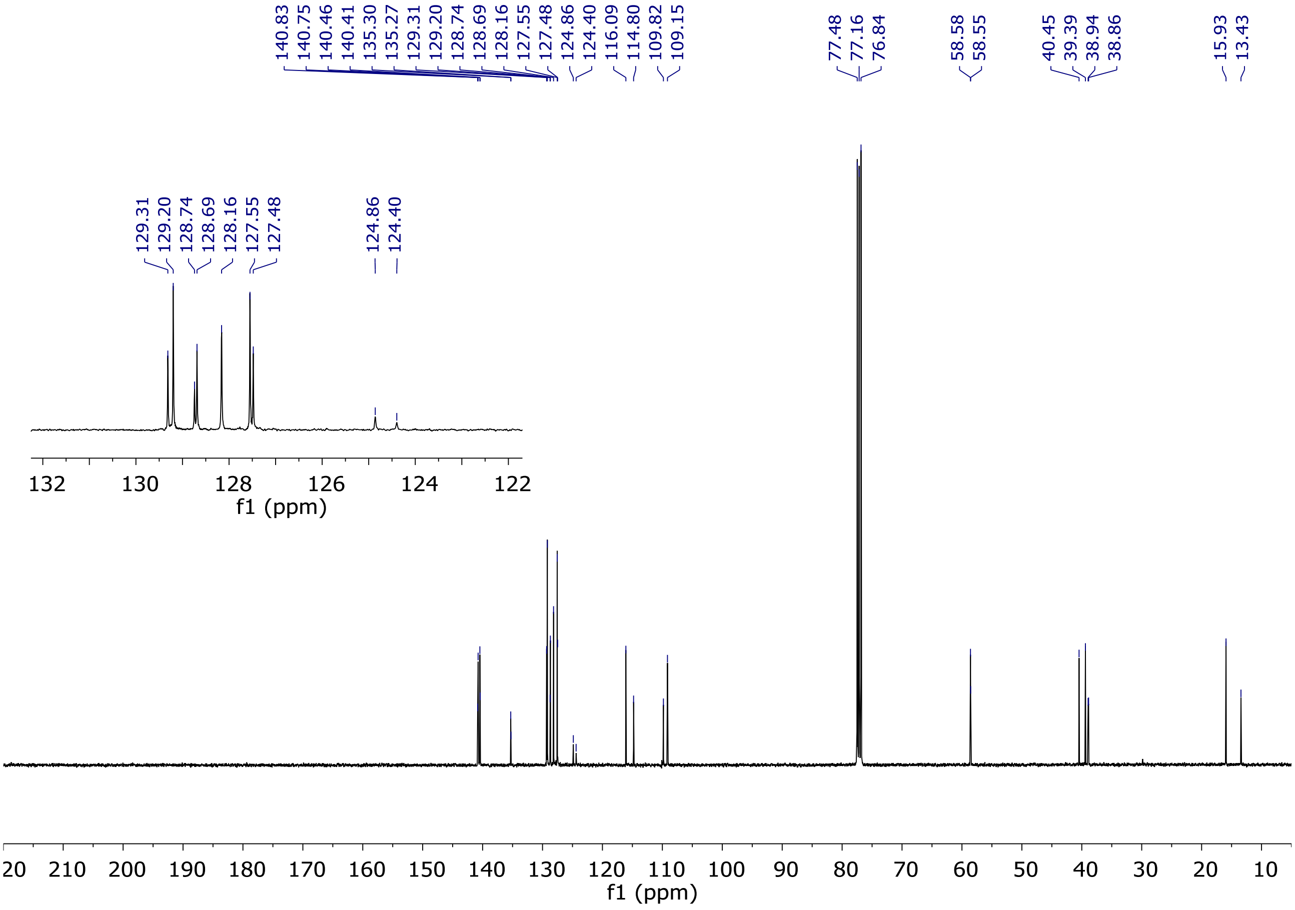

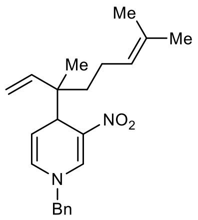

3af
2.1:1 mixture of
diastereoisomers

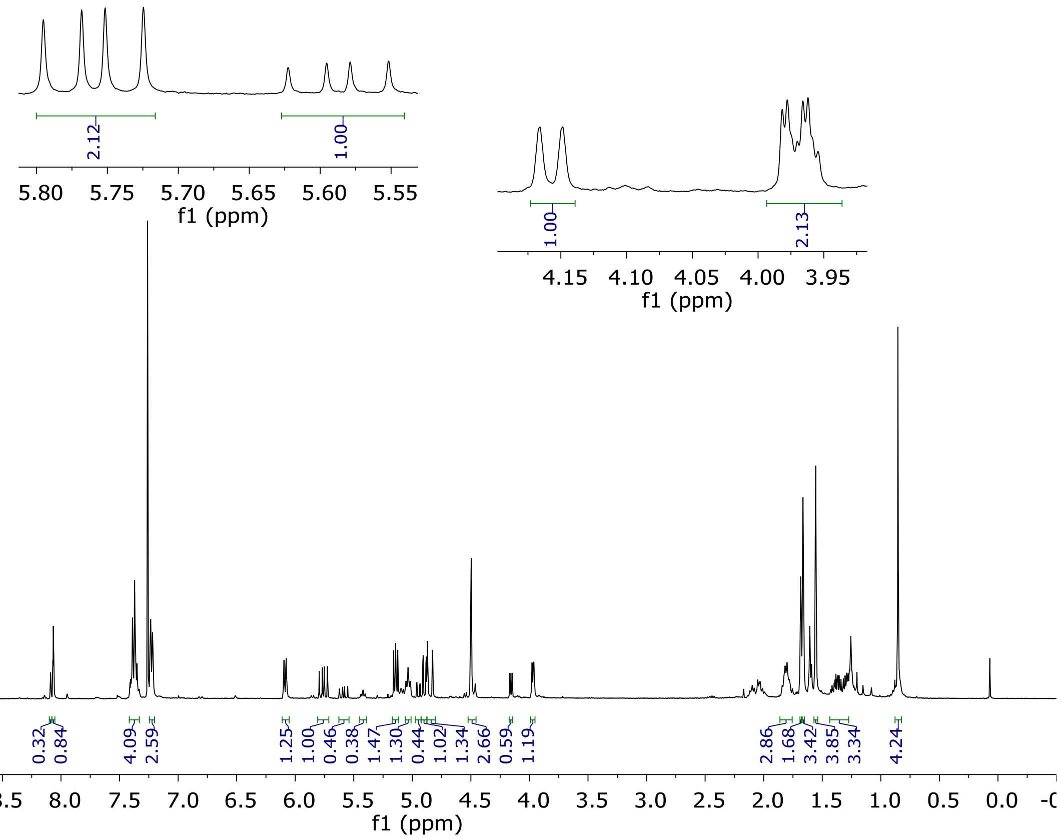

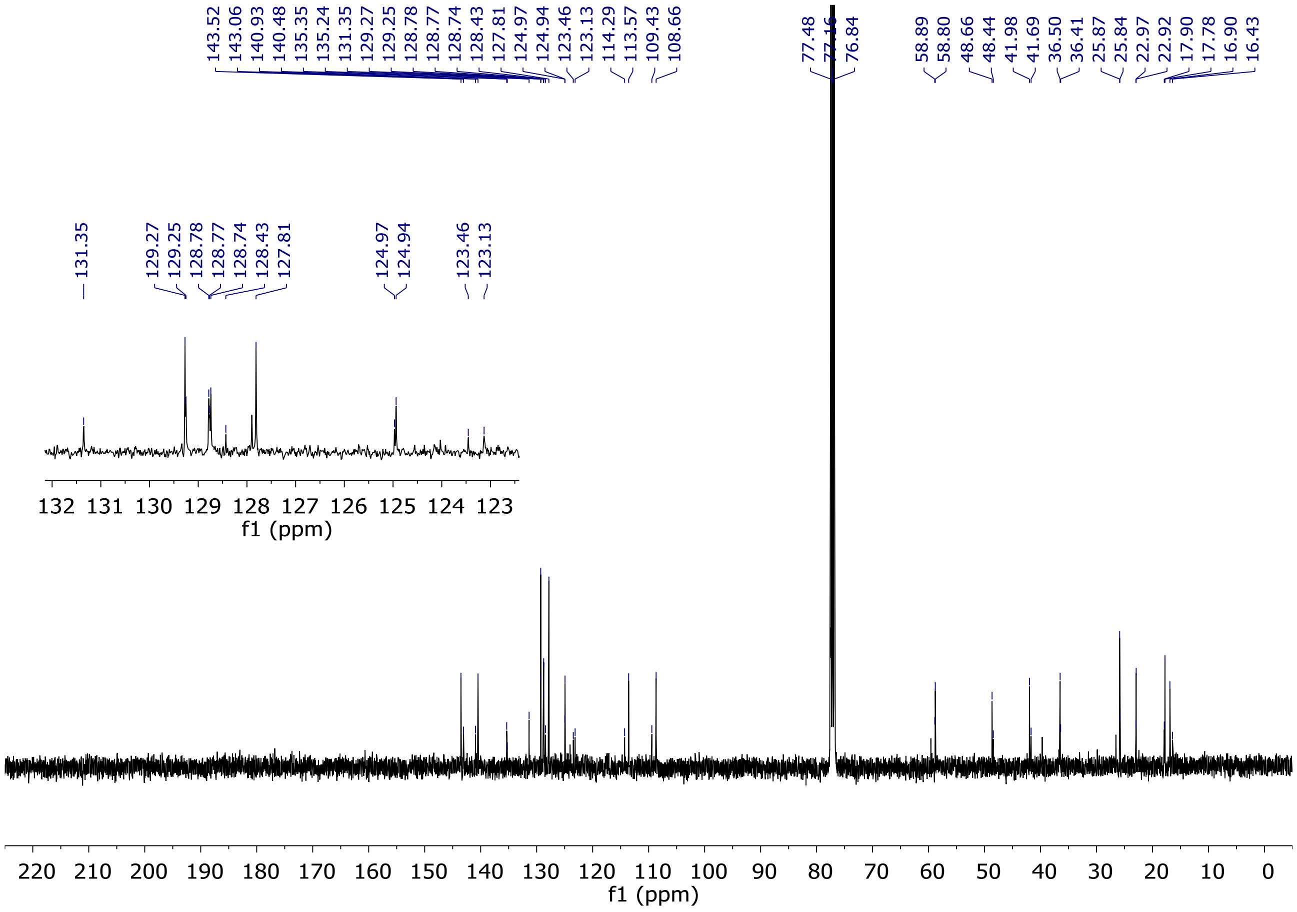

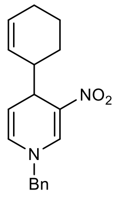

3ag
2.2:1 mixture of
diastereoisomers

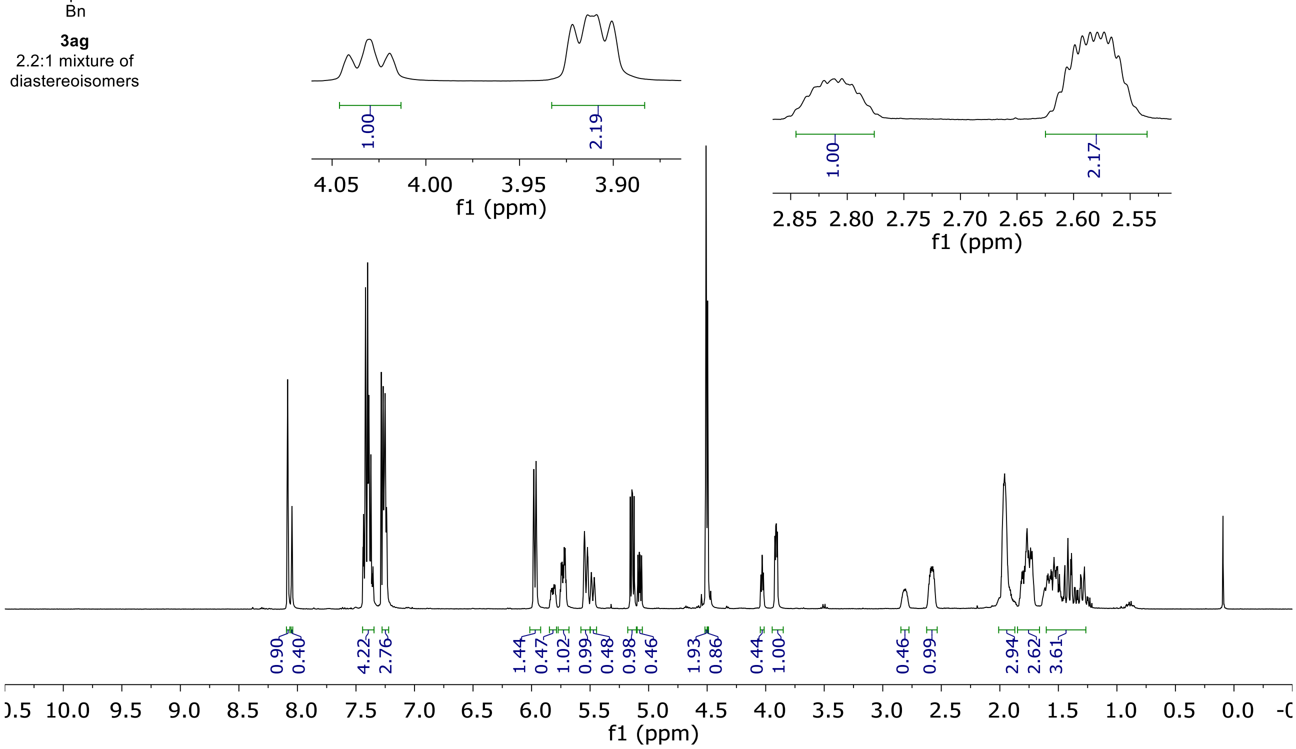

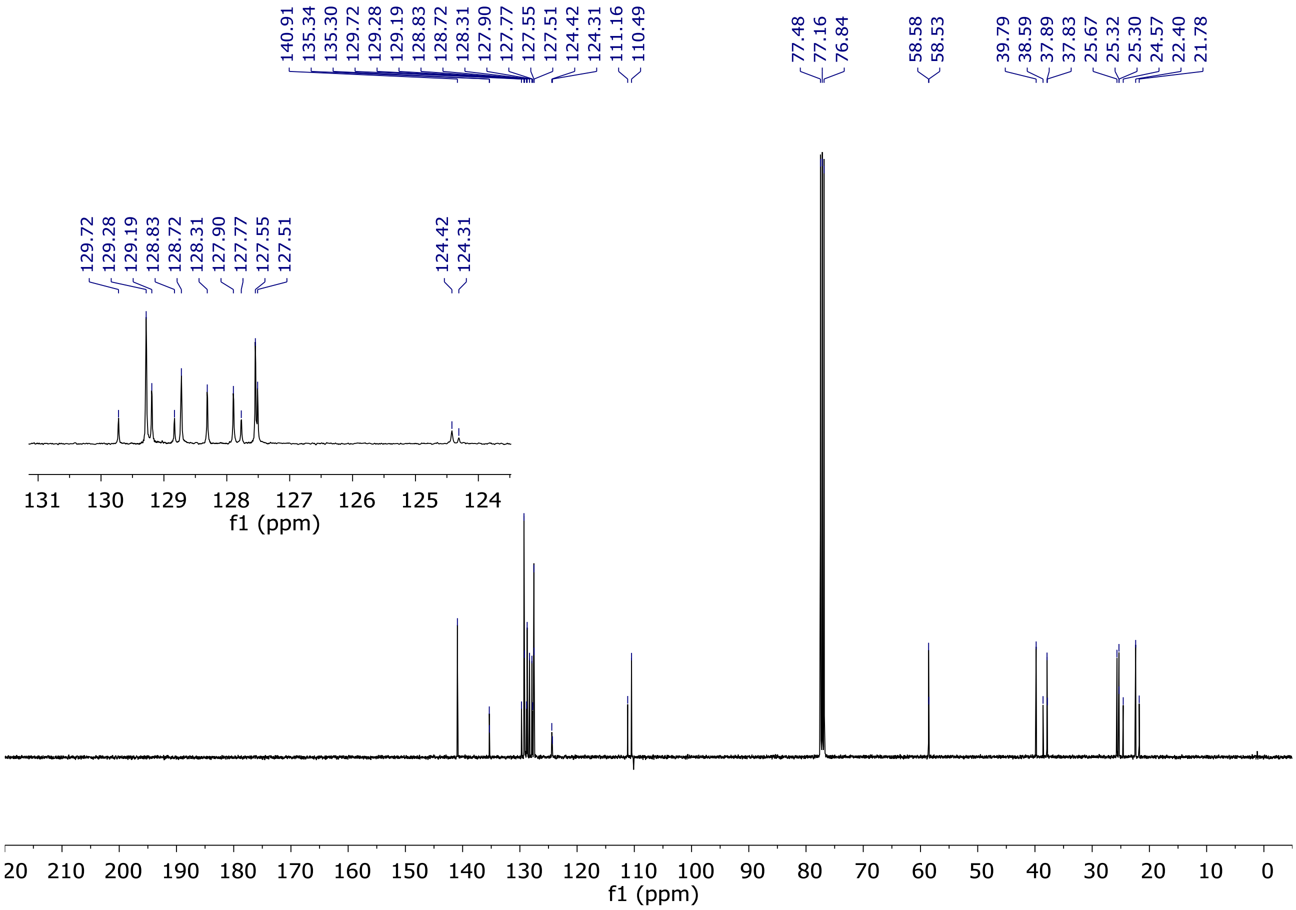

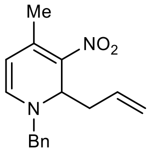
Me
NO2
N
Bn

3vba

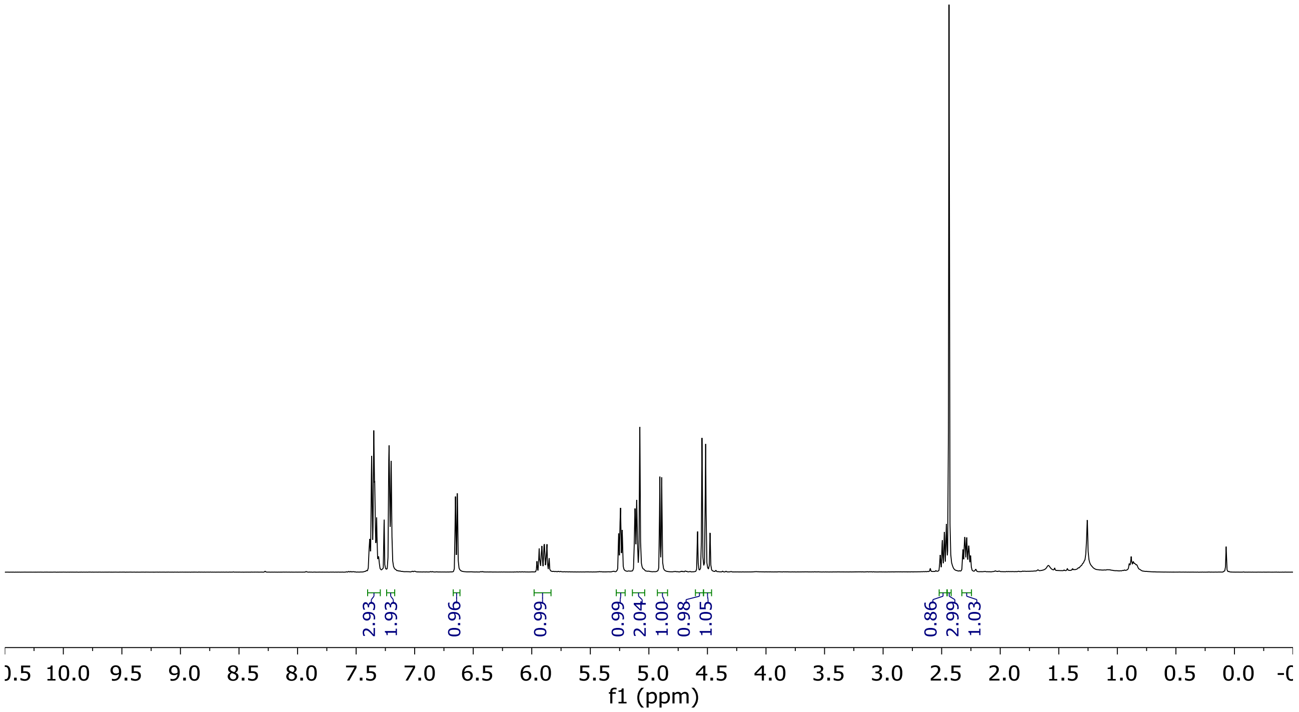
2.93
1.93
0.96
0.99
0.99
2.04
1.00
0.98
1.05
0.86
2.99
1.03
f1 (ppm)

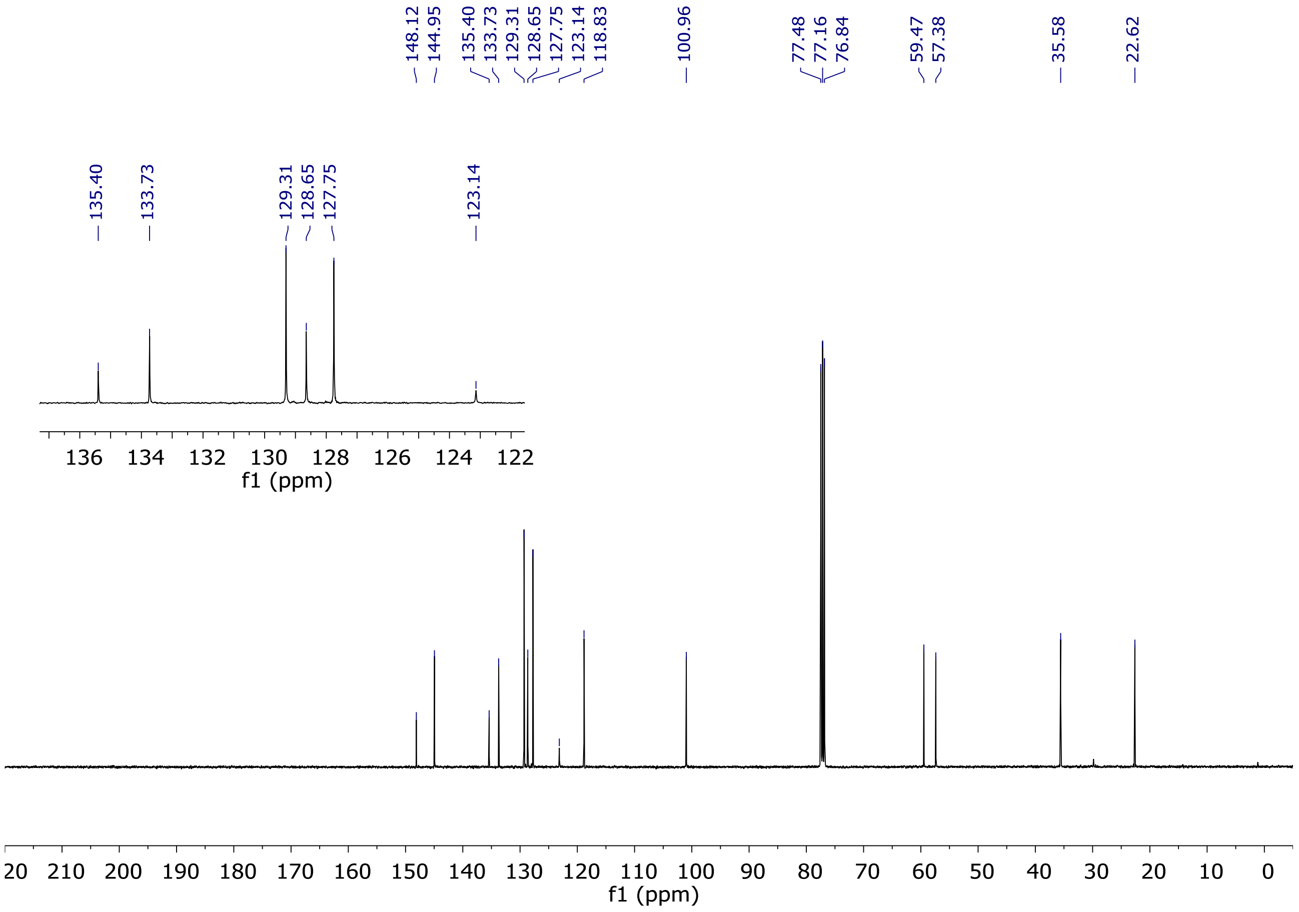
148.12
144.95
135.40
133.73
129.31
128.65
127.75
123.14
118.83
100.96
77.48
77.16
76.84
59.47
57.38
35.58
22.62
f1 (ppm)

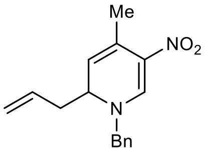
Me
NO_2
N
Bn

3vbb

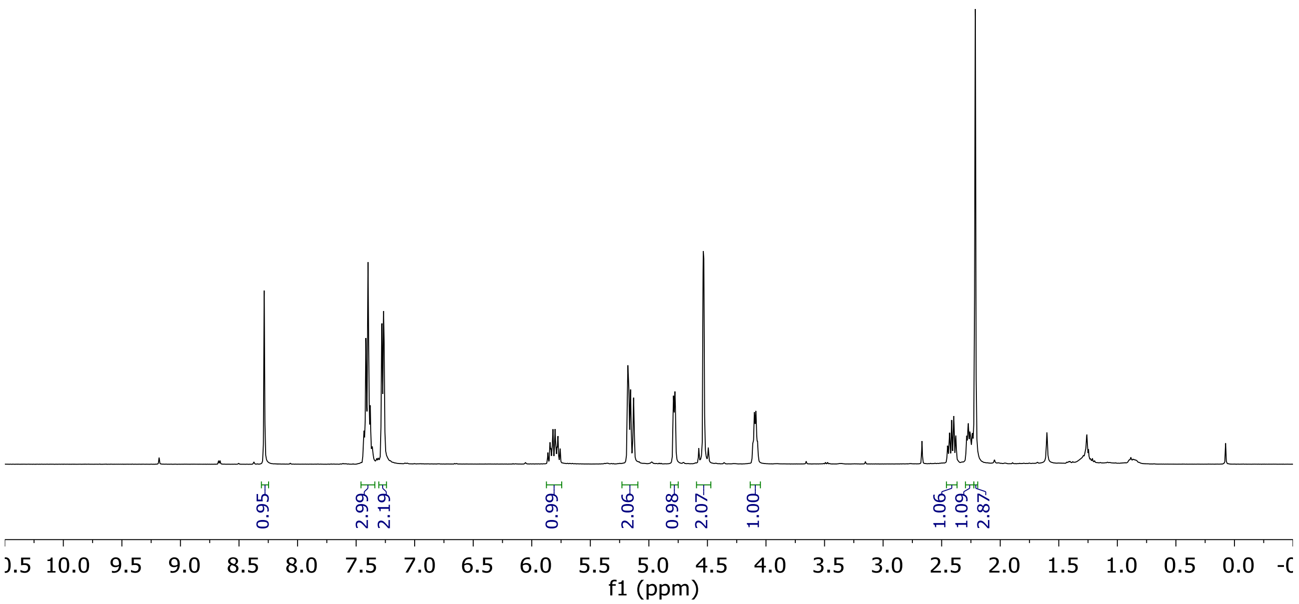
0.95
2.99
2.19
0.99
2.06
0.98
2.07
1.00
1.06
1.09
2.87
f1 (ppm)

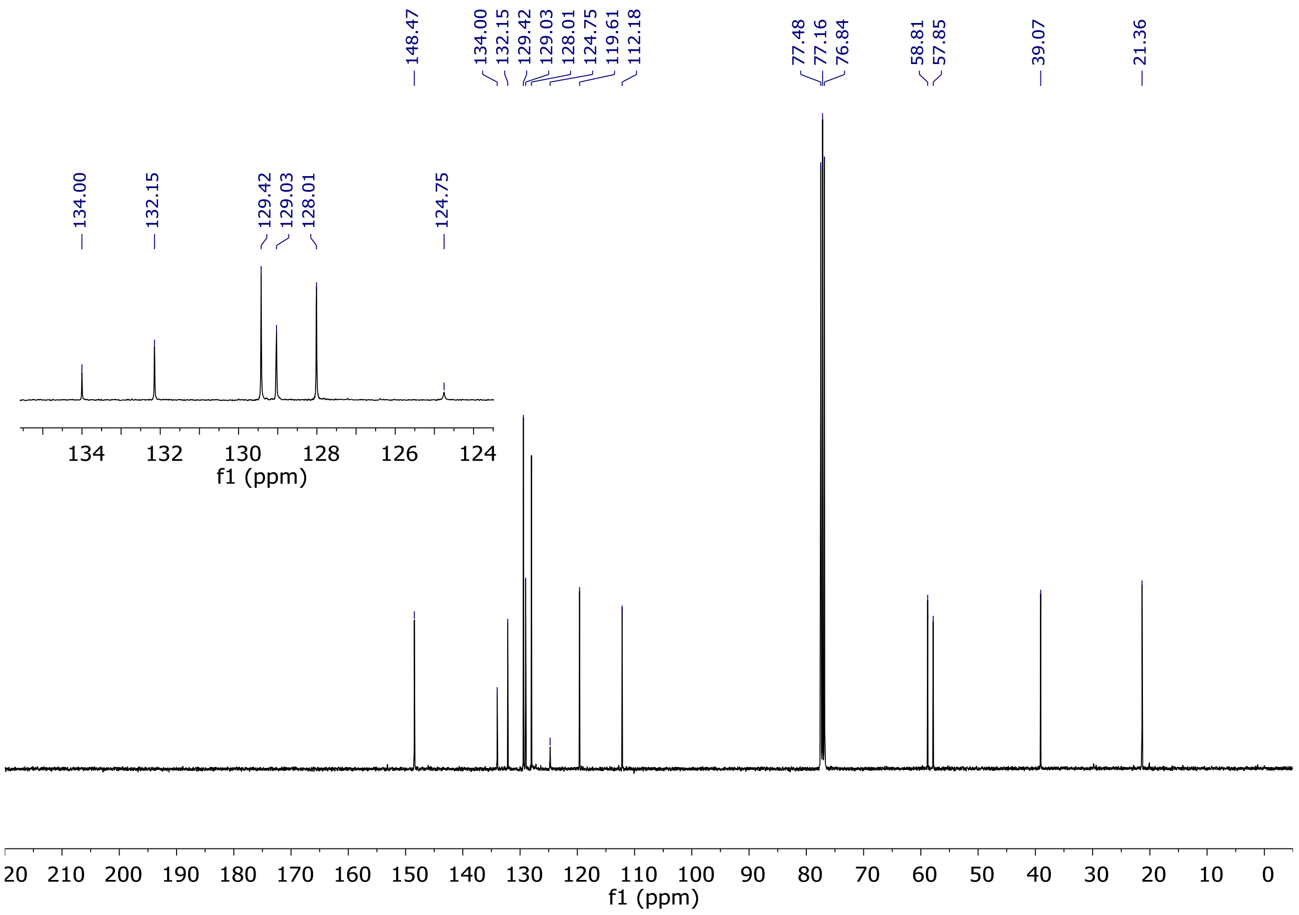
148.47
134.00
132.15
129.42
129.03
128.01
124.75
119.61
112.18
77.48
77.16
76.84
58.81
57.85
39.07
21.36
f1 (ppm)

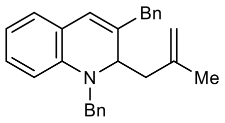
Bn
N
Bn
Me

3wa

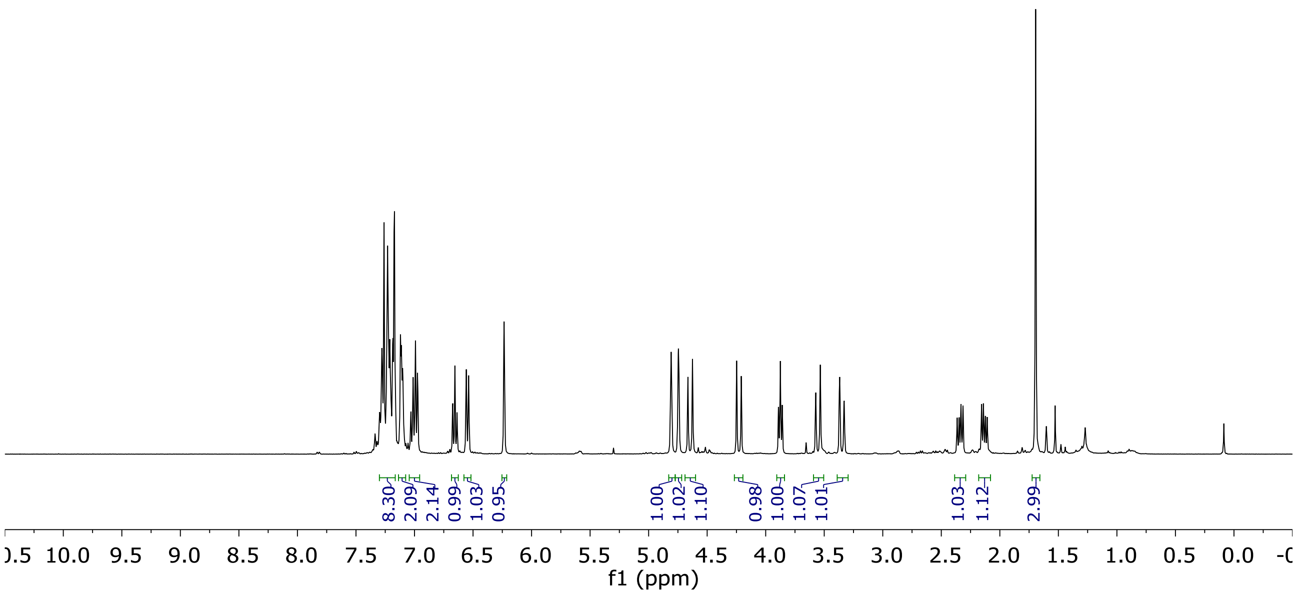
8.30
2.09
2.14
0.99
1.03
0.95
1.00
1.02
1.10
0.98
1.00
1.07
1.01
1.03
1.12
2.99
f1 (ppm)

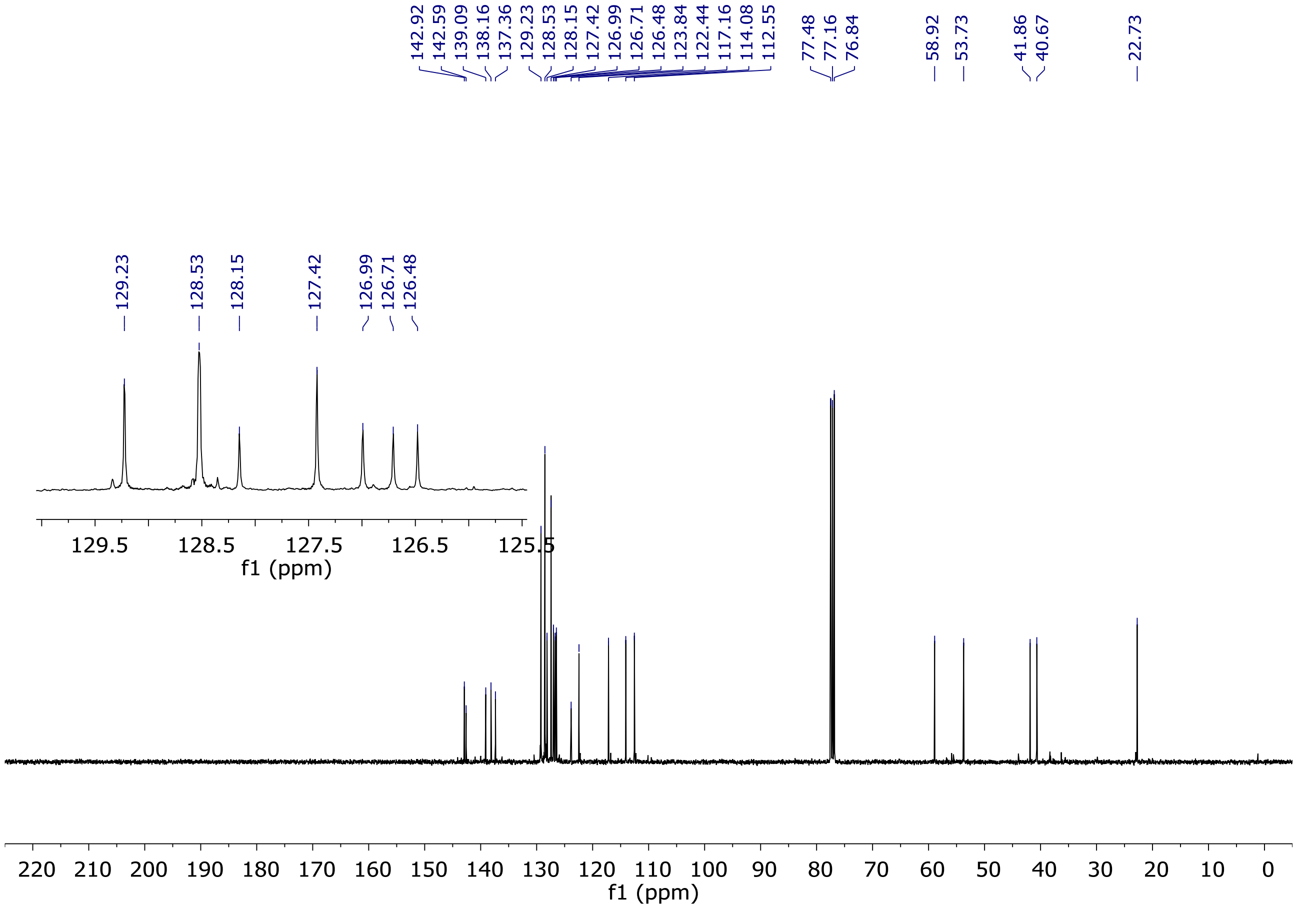
142.92
142.59
139.09
138.16
137.36
129.23
128.53
128.15
127.42
126.99
126.71
126.48
123.84
122.44
117.16
114.08
112.55
77.48
77.16
76.84
58.92
53.73
41.86
40.67
22.73
f1 (ppm)

HMBC Spectrum

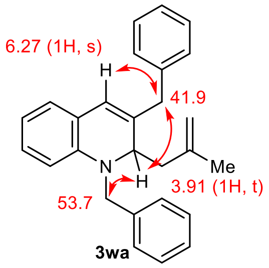

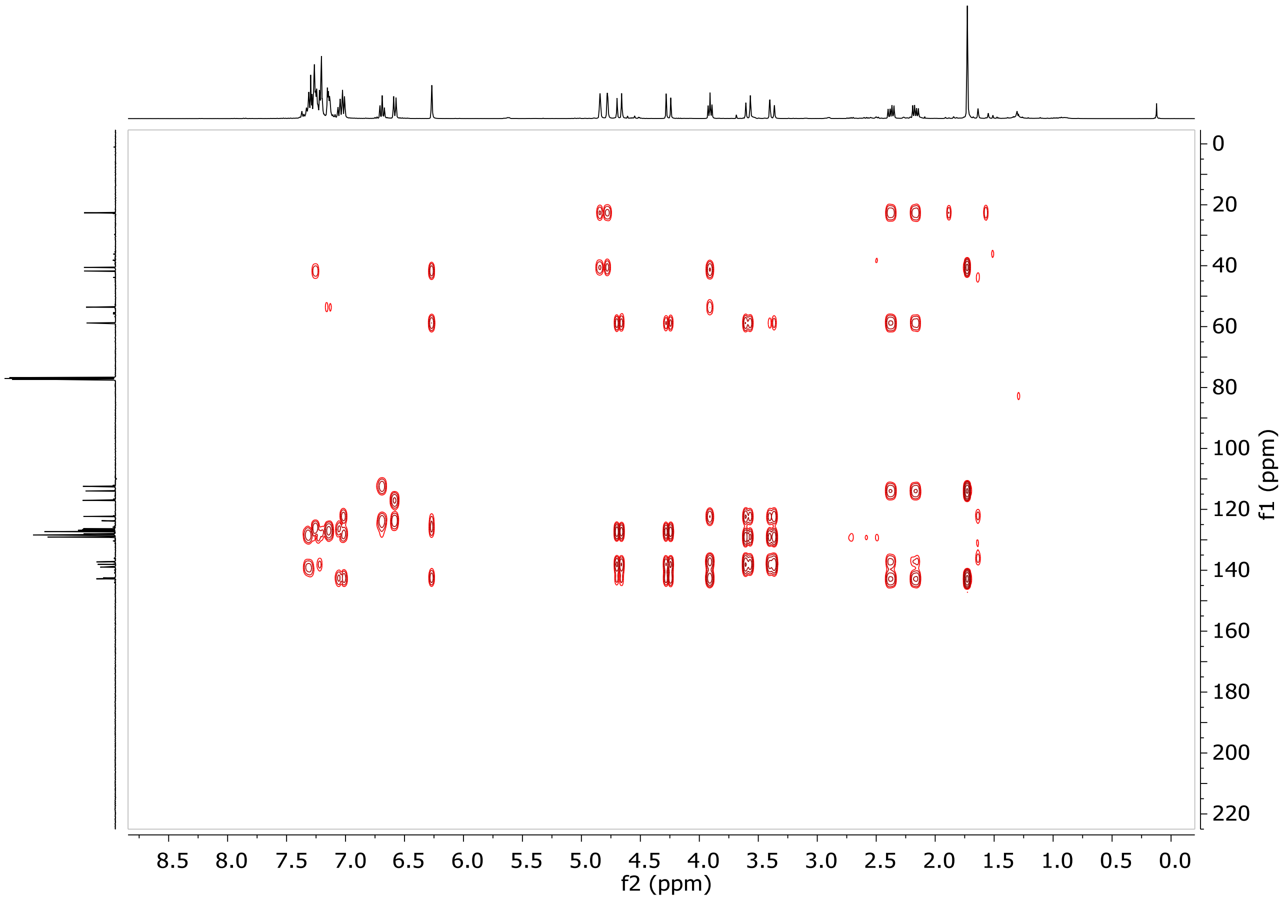

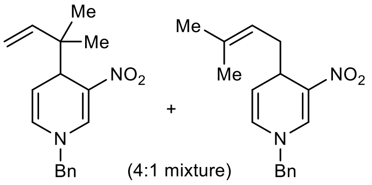
Me
Me
NO2
N
Bn
+
(4:1 mixture)
Me
Me
NO2
N
Bn

3ad **3ah**

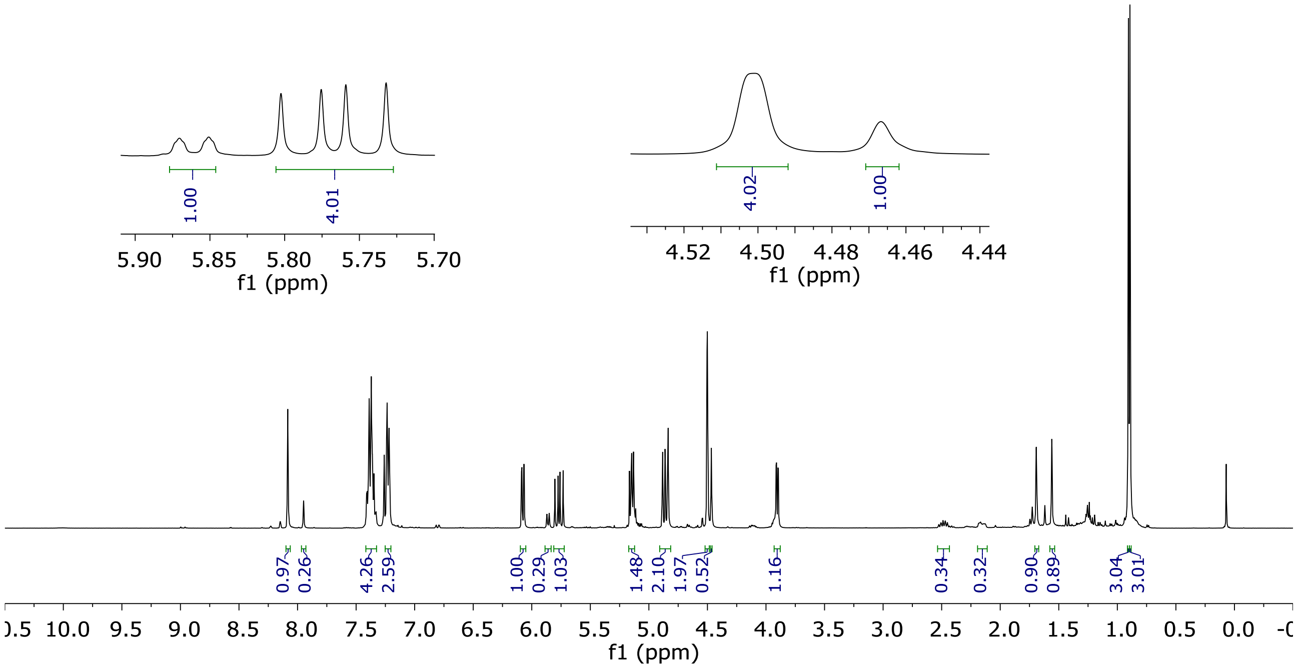
1.00
4.01
5.90 5.85 5.80 5.75 5.70
f1 (ppm)
4.02
1.00
4.52 4.50 4.48 4.46 4.44
f1 (ppm)
0.97
0.26
4.26
2.59
1.00
0.29
1.03
1.48
2.10
1.97
0.52
1.16
0.34
0.32
0.90
0.89
3.04
3.01
10.0 9.5 9.0 8.5 8.0 7.5 7.0 6.5 6.0 5.5 5.0 4.5 4.0 3.5 3.0 2.5 2.0 1.5 1.0 0.5 0.0
f1 (ppm)

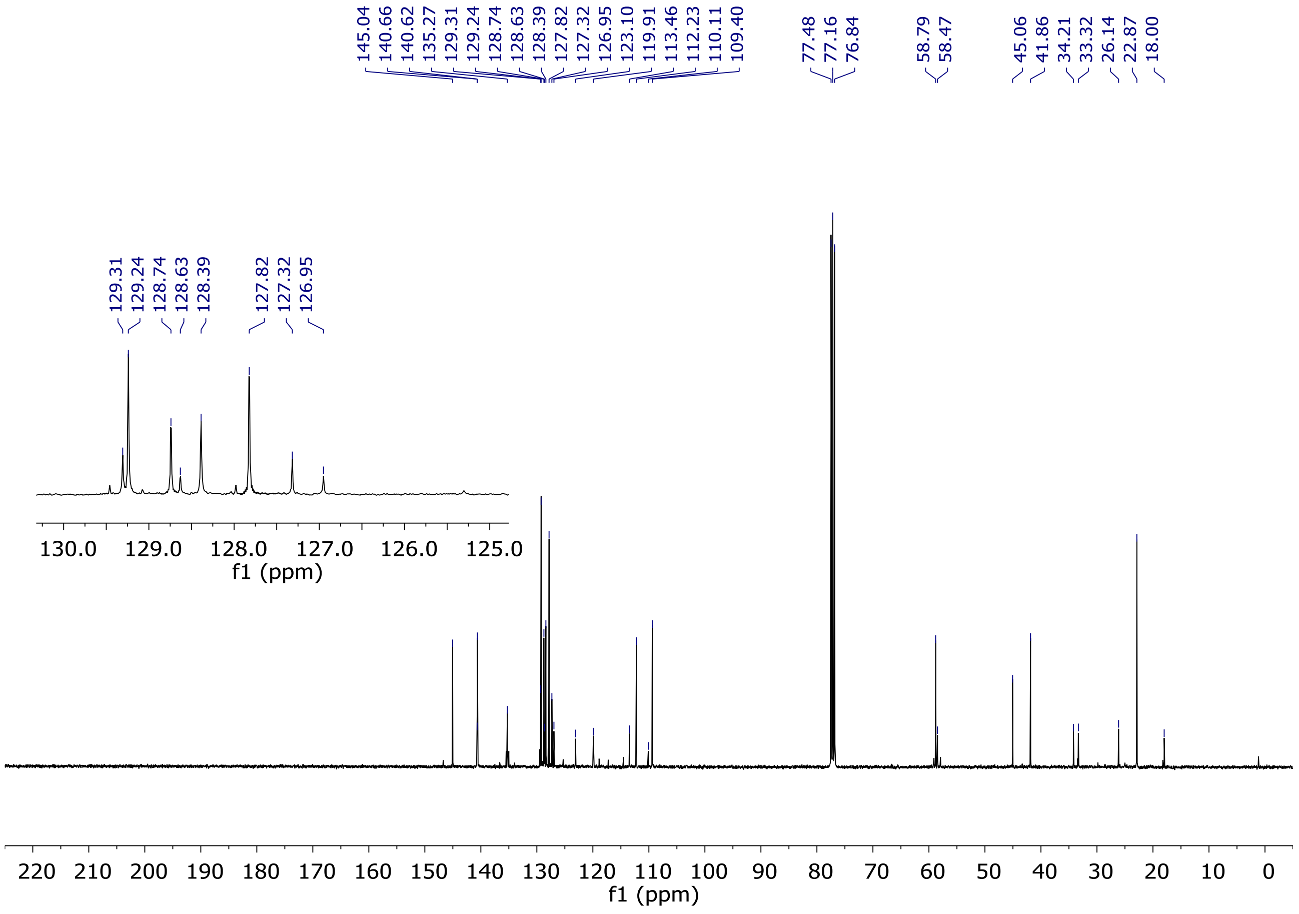
145.04
140.66
140.62
135.27
129.31
129.24
128.74
128.63
128.39
127.82
127.32
126.95
123.10
119.91
113.46
112.23
110.11
109.40
77.48
77.16
76.84
58.79
58.47
45.06
41.86
34.21
33.32
26.14
22.87
18.00
129.31
129.24
128.74
128.63
128.39
127.82
127.32
126.95
130.0 129.0 128.0 127.0 126.0 125.0
f1 (ppm)
220 210 200 190 180 170 160 150 140 130 120 110 100 90 80 70 60 50 40 30 20 10 0
f1 (ppm)

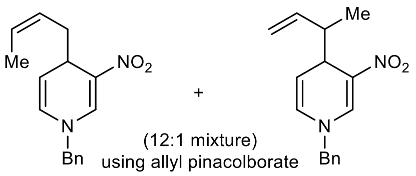

3ai

3ae

2:1 mixture of diastereoisomers

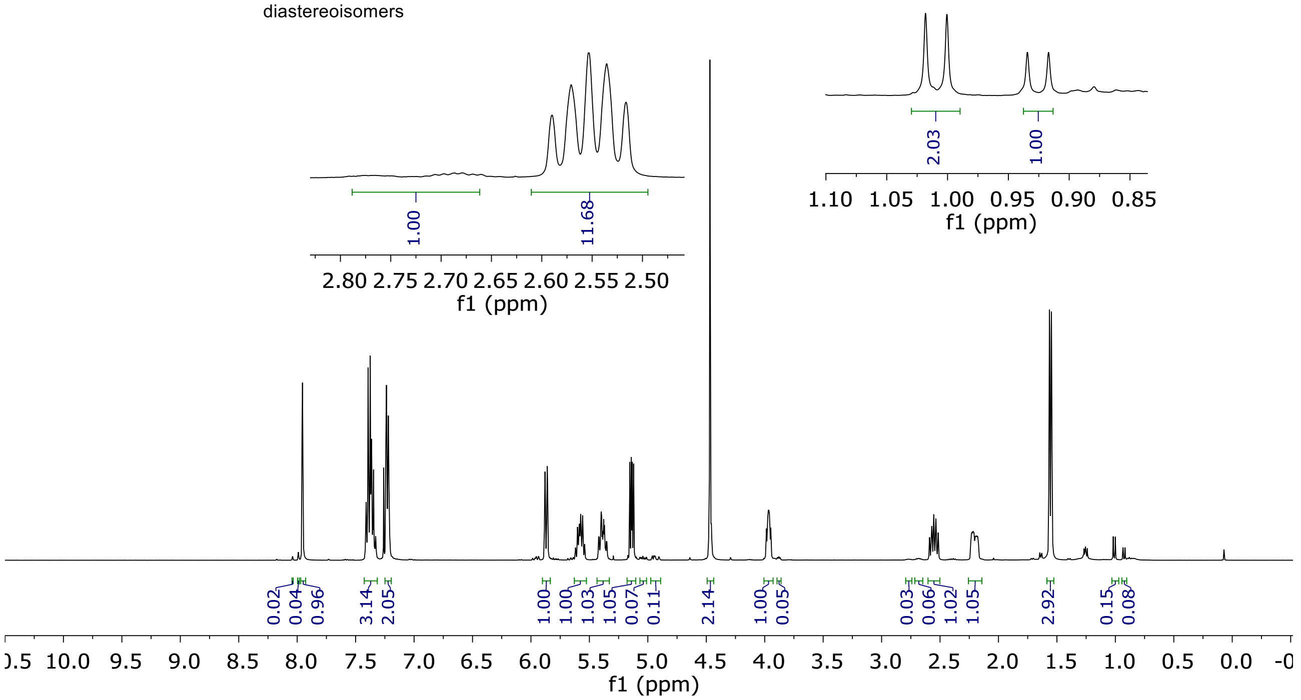

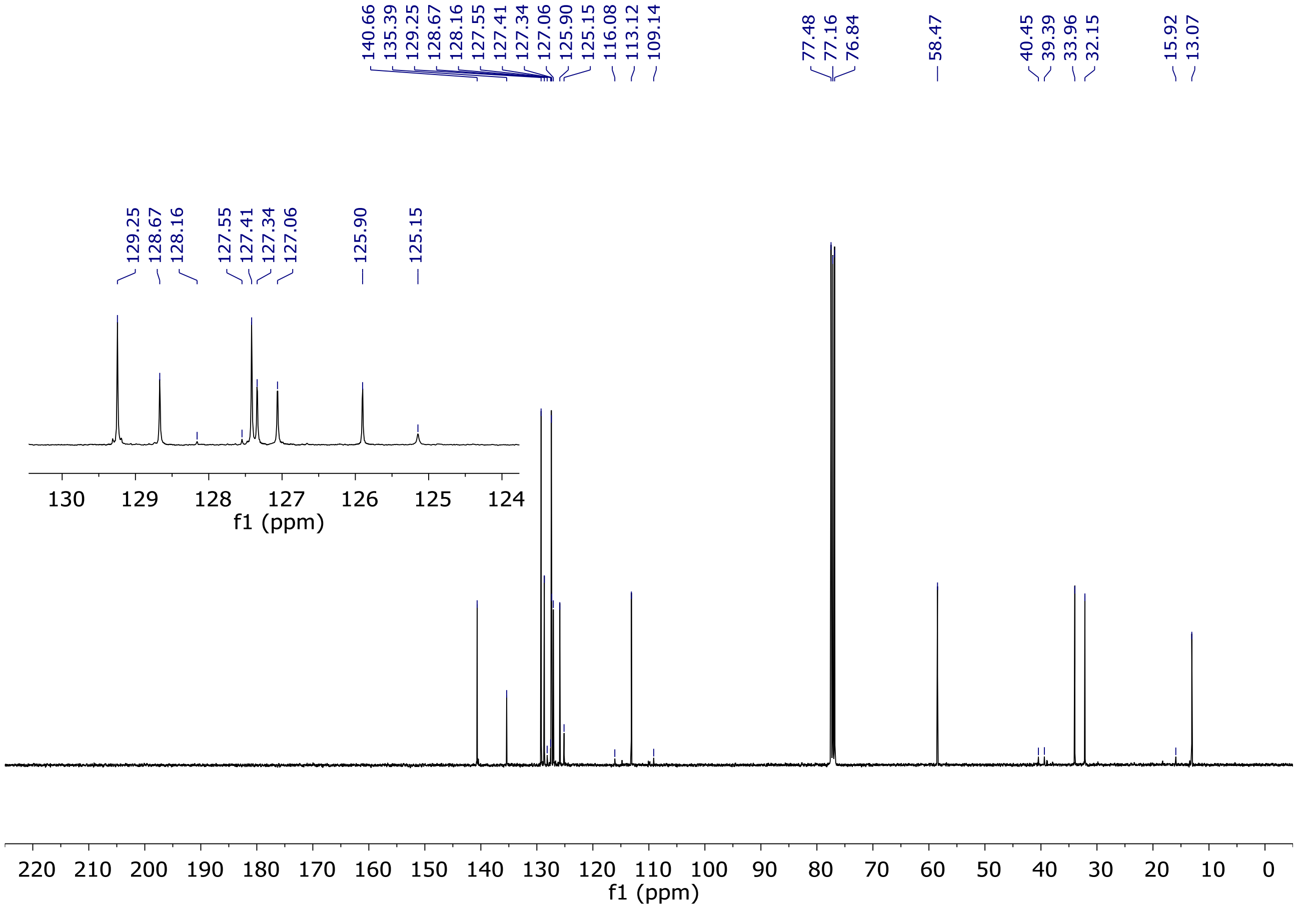

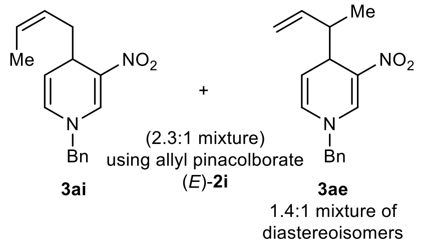

Me
NO2
N
Bn
3ai
+
(2.3:1 mixture)
using allyl pinacolborate
(E)-2i
Me
NO2
N
Bn
3ae
1.4:1 mixture of
diastereoisomers

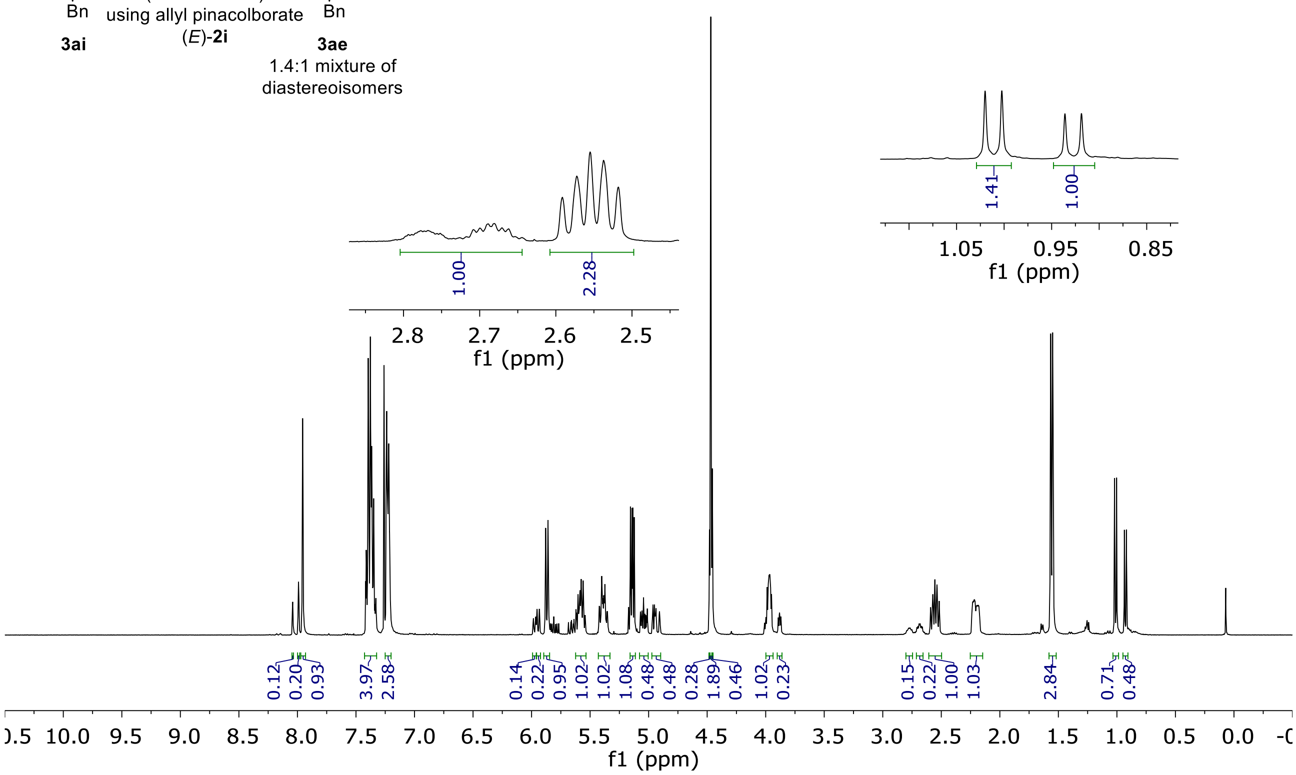

f1 (ppm)

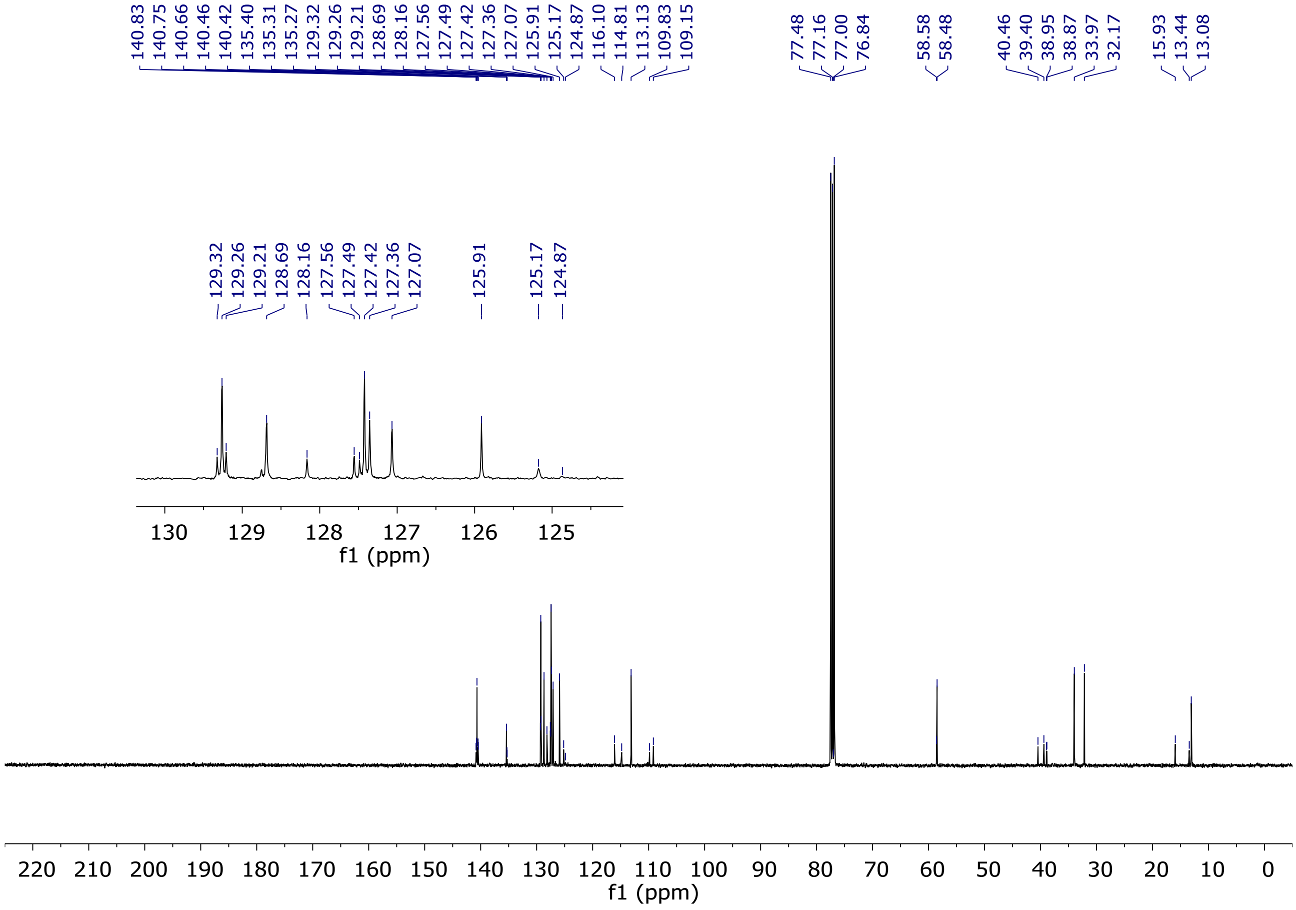

140.83
140.75
140.66
140.46
140.42
135.40
135.31
135.27
129.32
129.26
129.21
128.69
128.16
127.56
127.49
127.42
127.36
127.07
125.91
125.17
124.87
116.10
114.81
113.13
109.83
109.15
77.48
77.16
77.00
76.84
58.58
58.48
40.46
39.40
38.95
38.87
33.97
32.17
15.93
13.44
13.08
f1 (ppm)

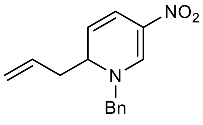
NO2
N
Bn

S6

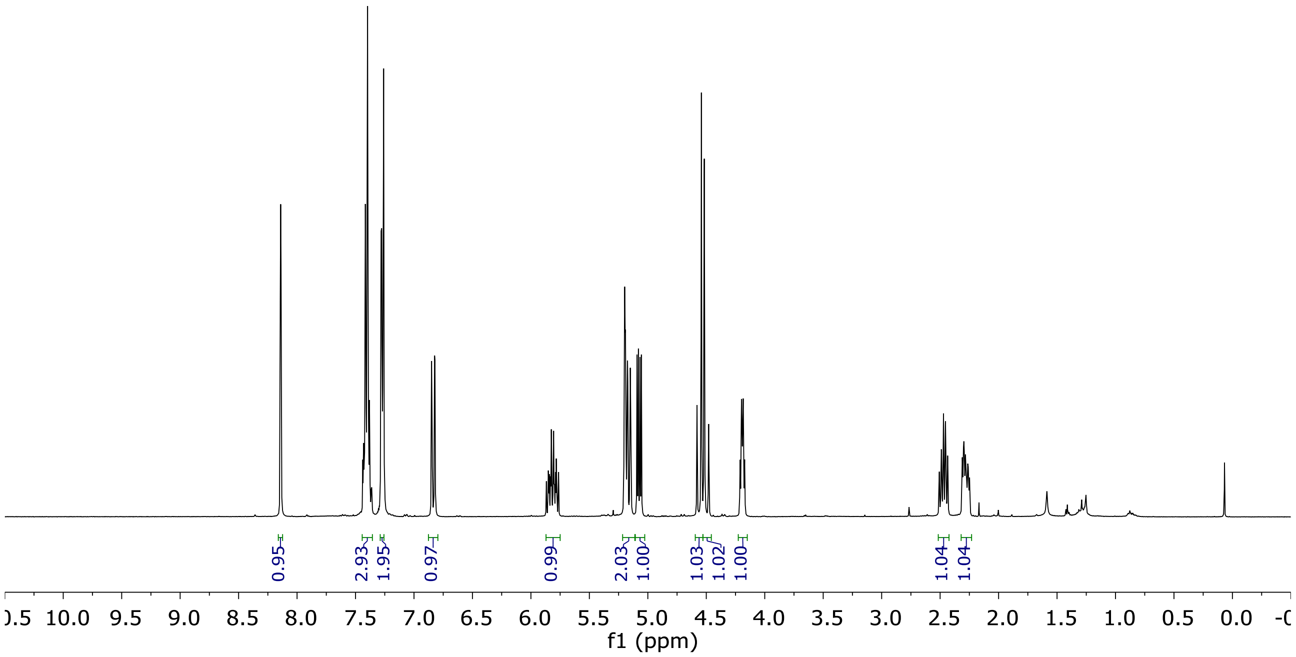
0.95
2.93
1.95
0.97
0.99
2.03
1.00
1.03
1.02
1.00
1.04
1.04
10.0 9.5 9.0 8.5 8.0 7.5 7.0 6.5 6.0 5.5 5.0 4.5 4.0 3.5 3.0 2.5 2.0 1.5 1.0 0.5 0.0
f1 (ppm)

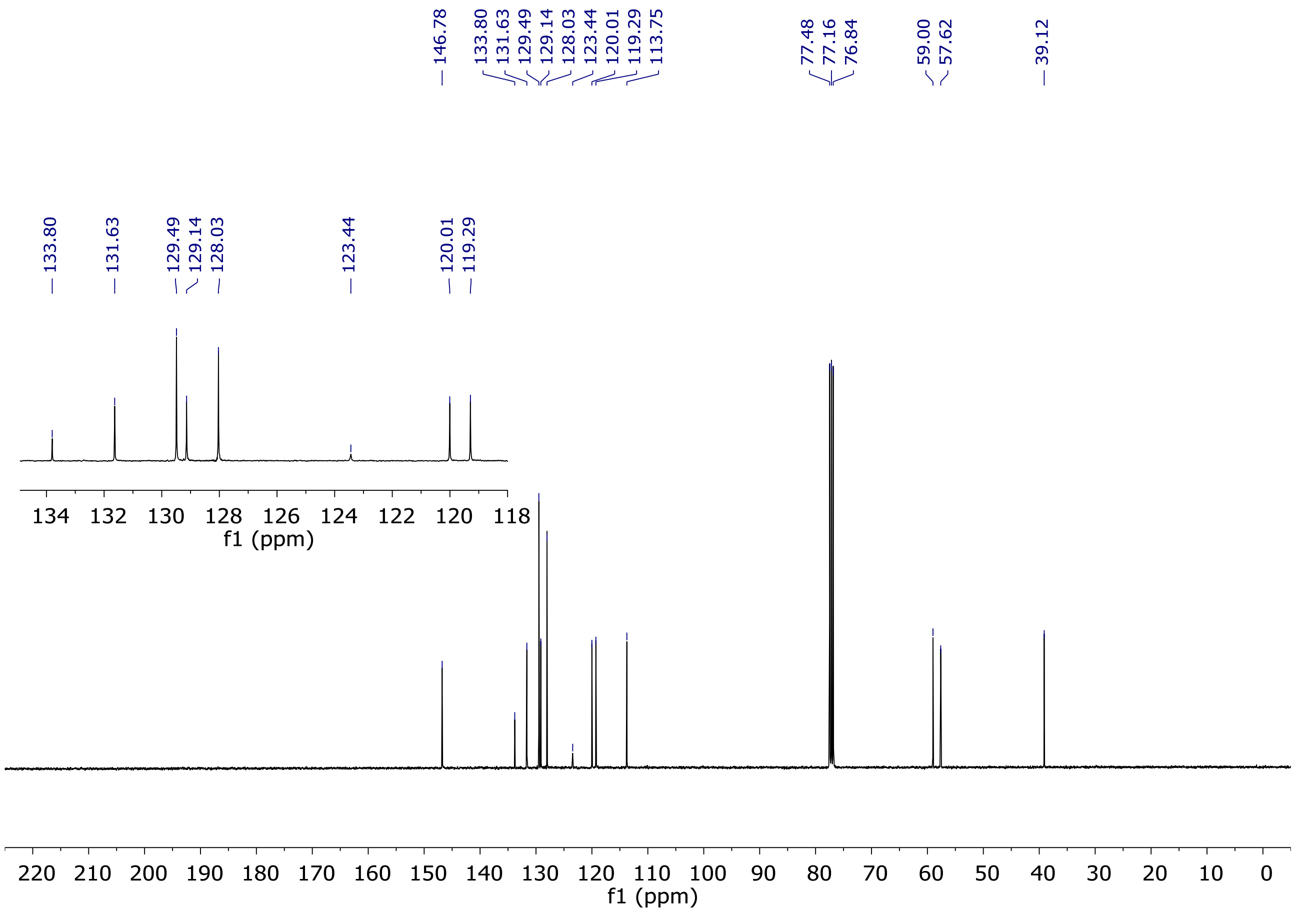
146.78
133.80
131.63
129.49
129.14
128.03
123.44
120.01
119.29
113.75
77.48
77.16
76.84
59.00
57.62
39.12
133.80
131.63
129.49
129.14
128.03
123.44
120.01
119.29
134 132 130 128 126 124 122 120 118
f1 (ppm)
220 210 200 190 180 170 160 150 140 130 120 110 100 90 80 70 60 50 40 30 20 10 0
f1 (ppm)

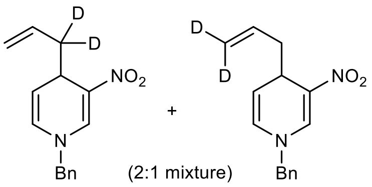

$[D]_2$-**3aba** $[D]_2$-**3abb**

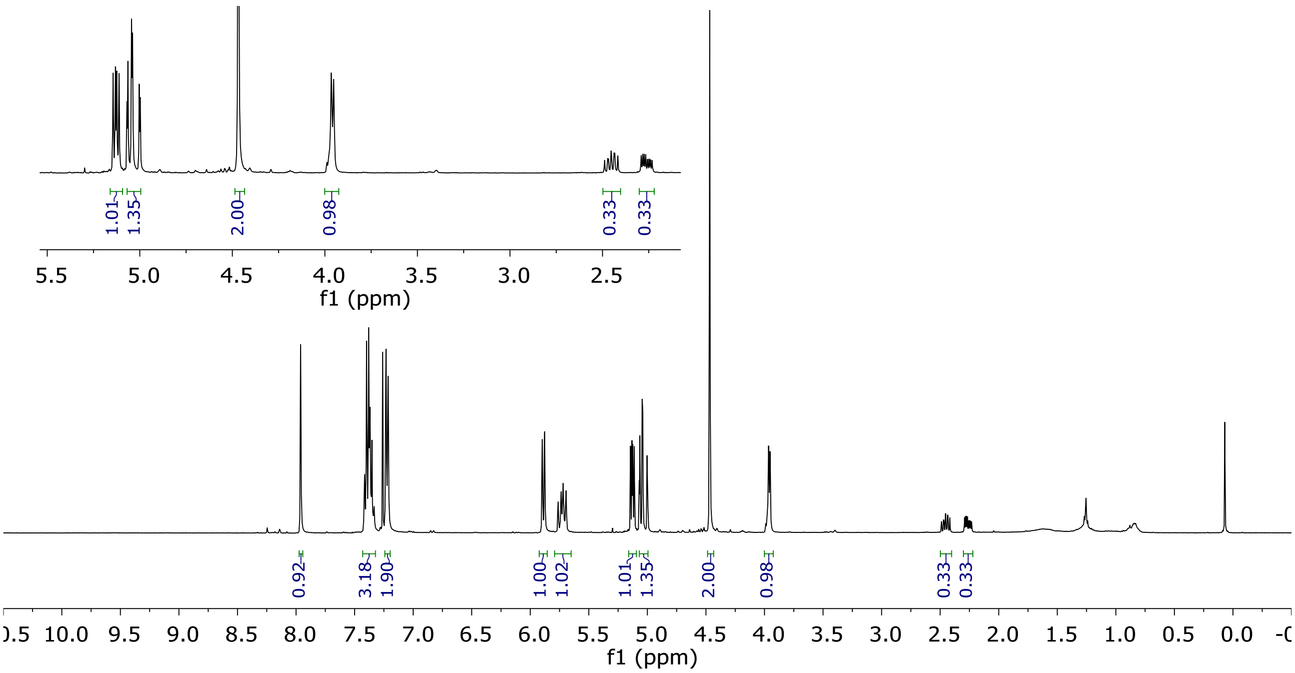

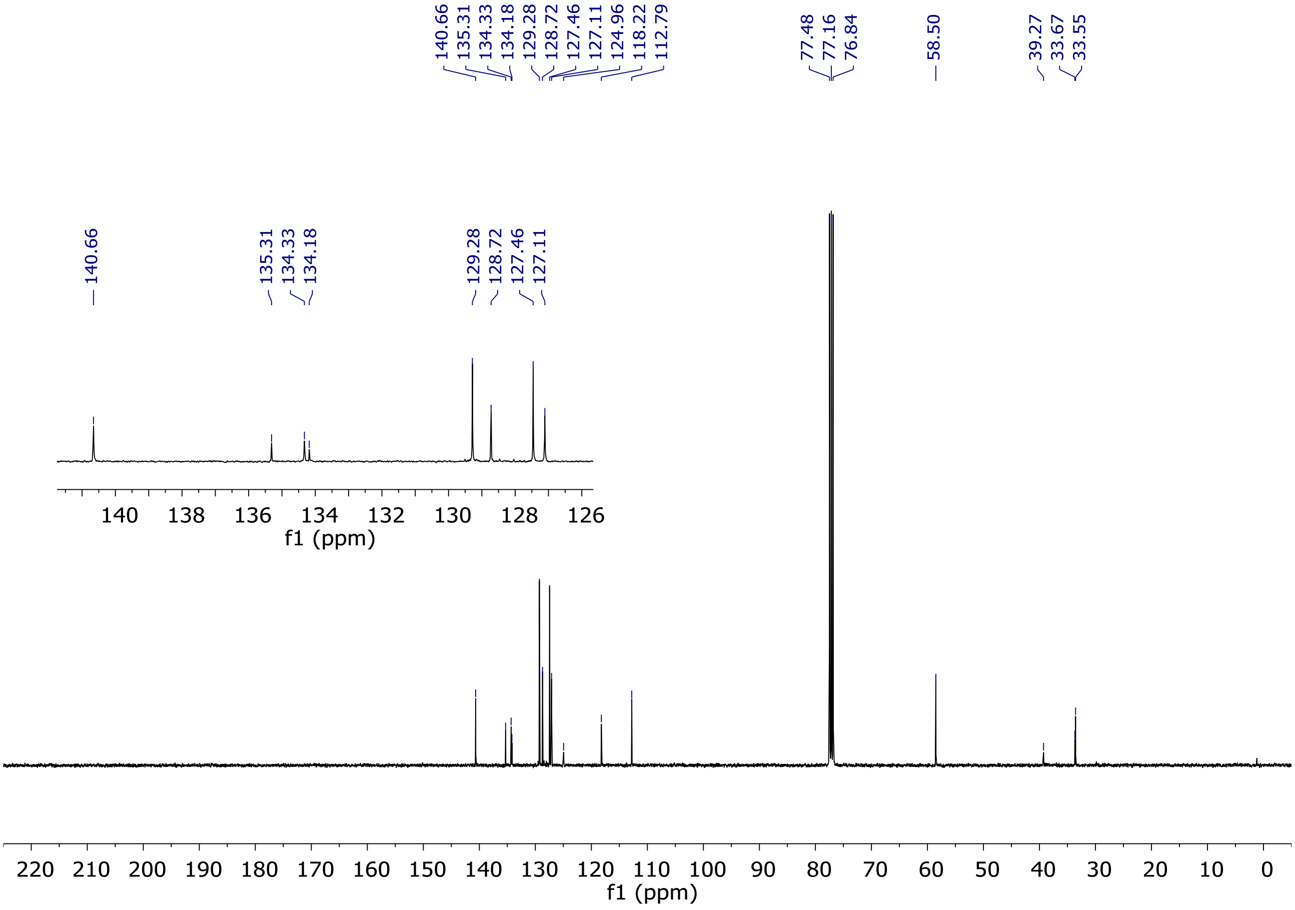

^{2}H NMR Spectrum

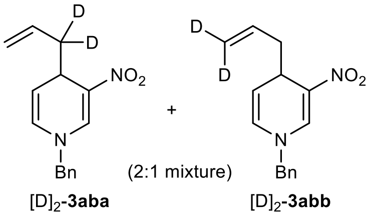

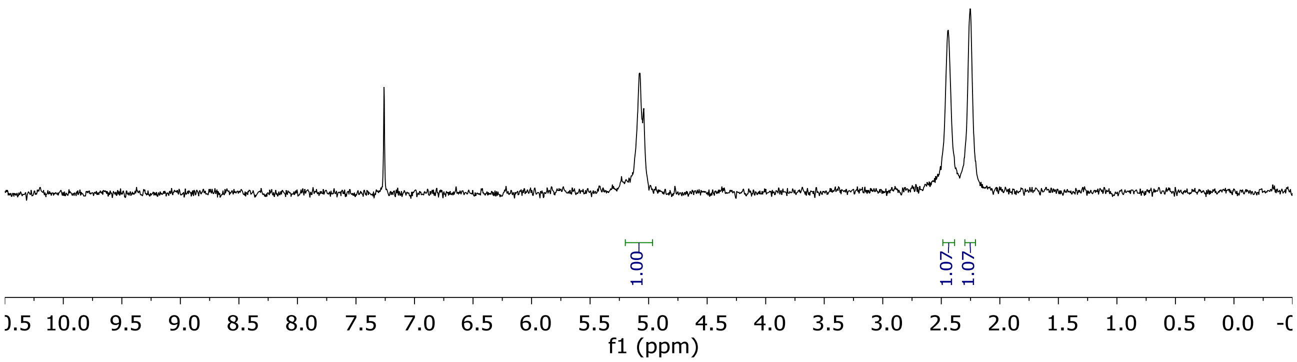

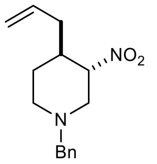

9
15:1 mixture of diastereomers

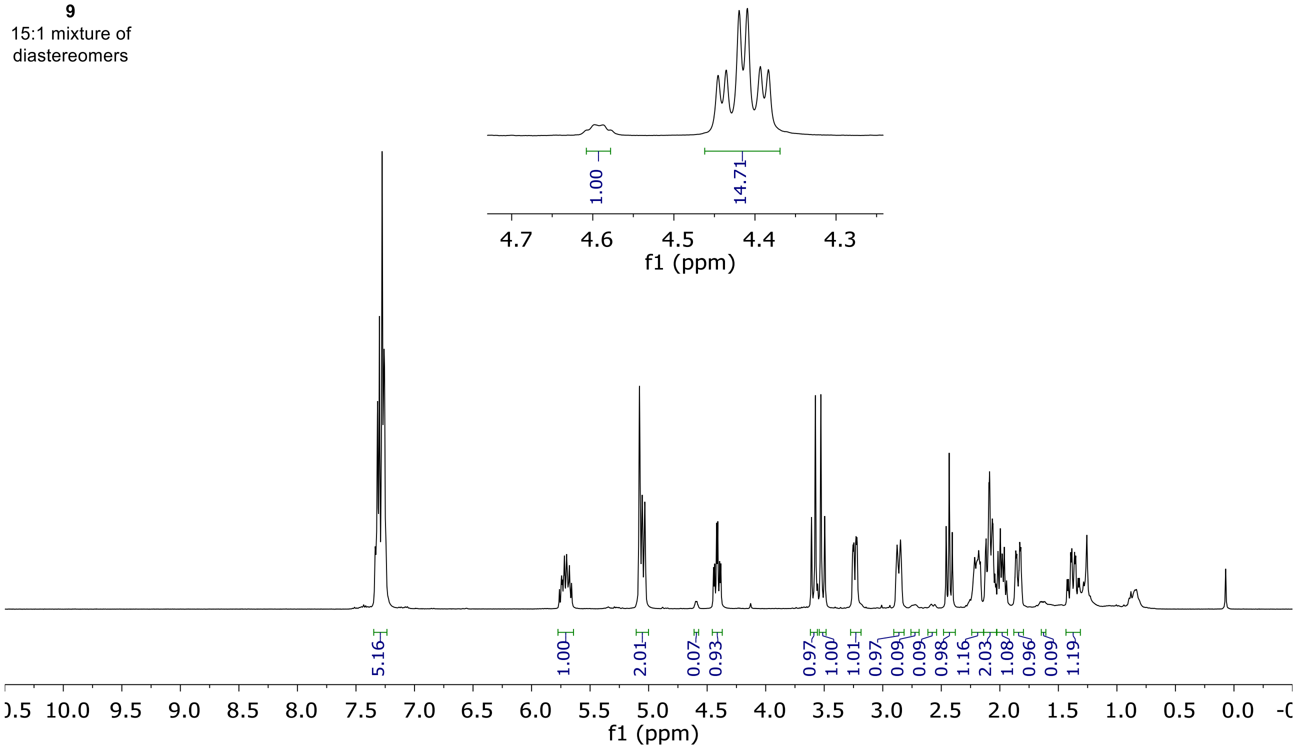

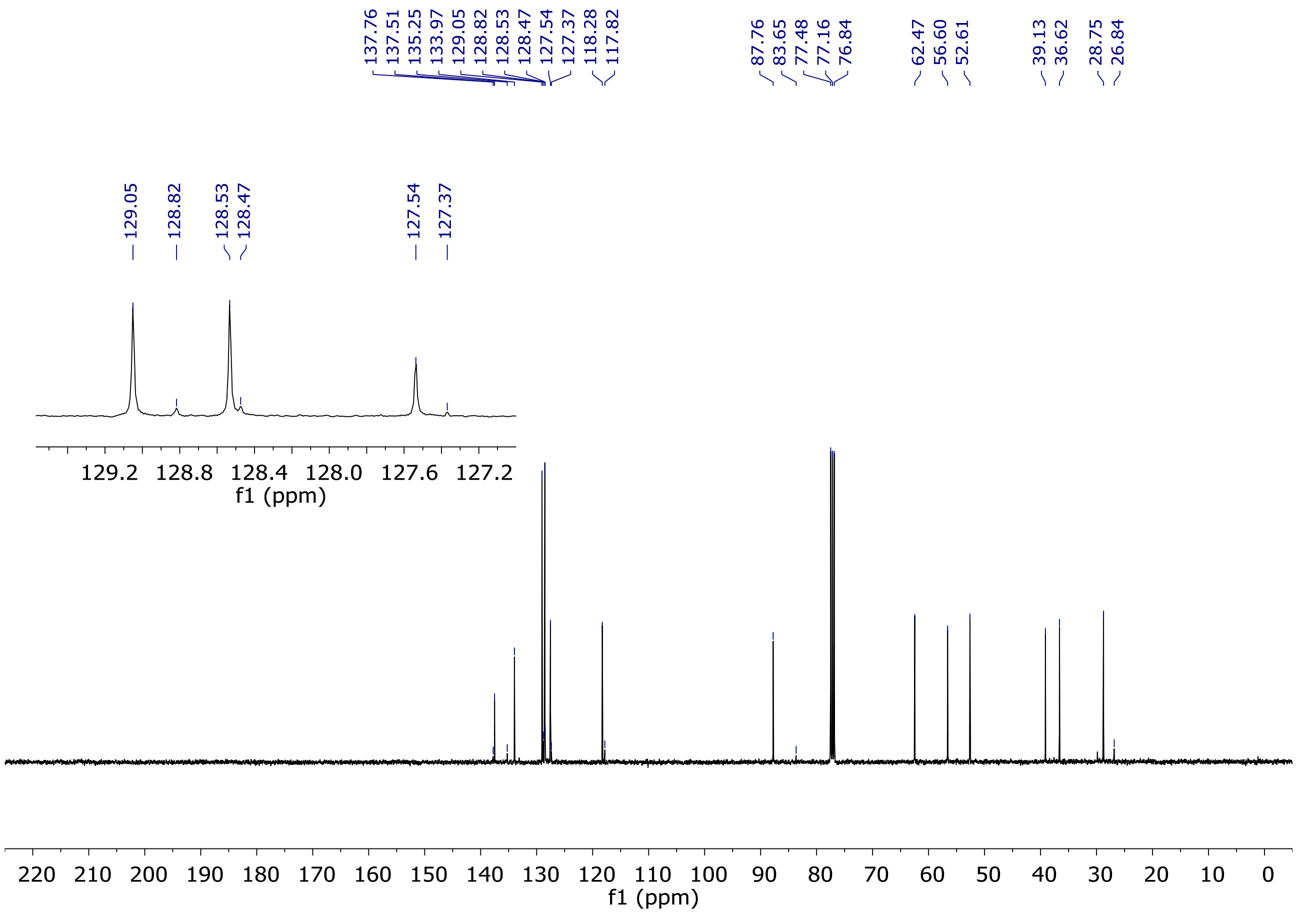

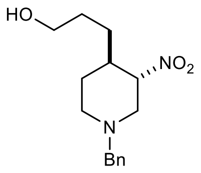

10
19:1 mixture of diastereomers

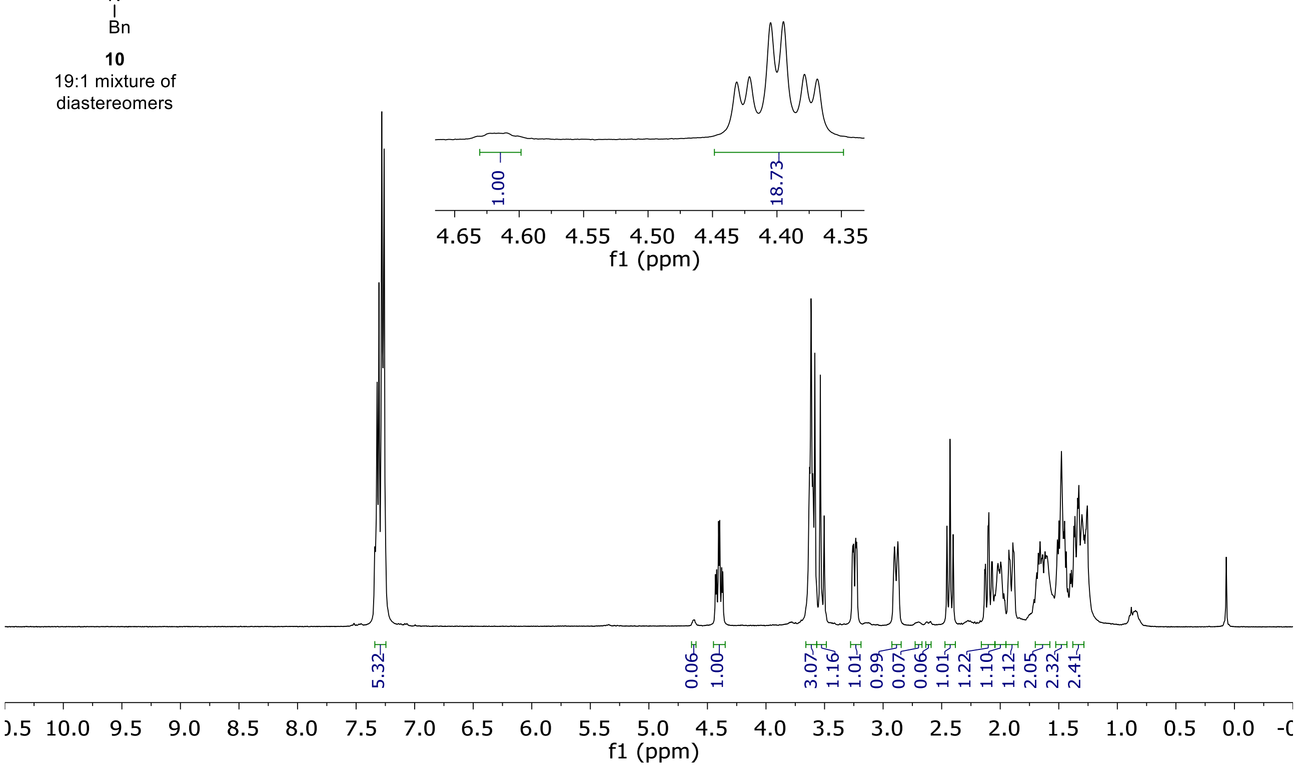

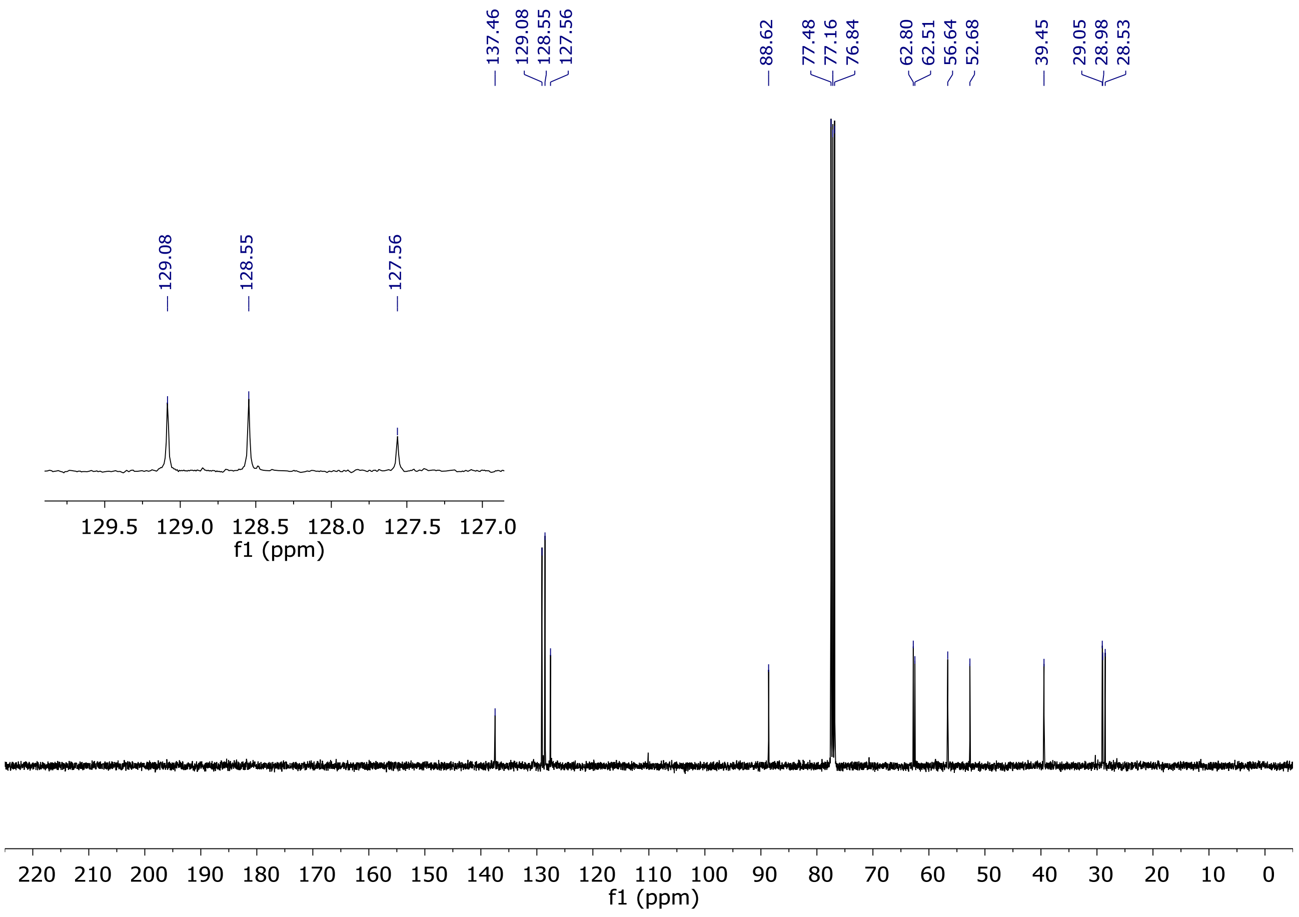

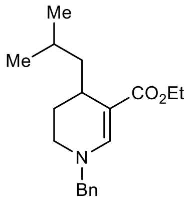
Me
Me
CO2Et
N
Bn

11

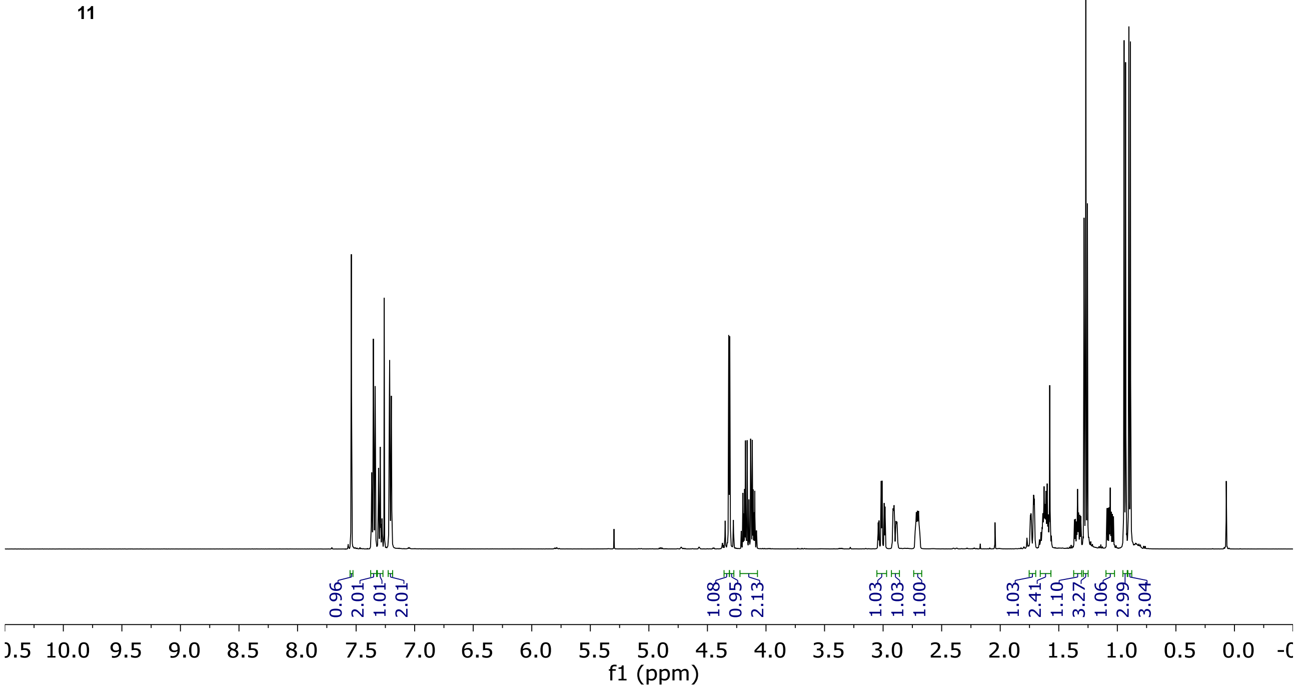
0.96
2.01
1.01
2.01
1.08
0.95
2.13
1.03
1.03
1.00
1.03
2.41
1.10
3.27
1.06
2.99
3.04
f1 (ppm)

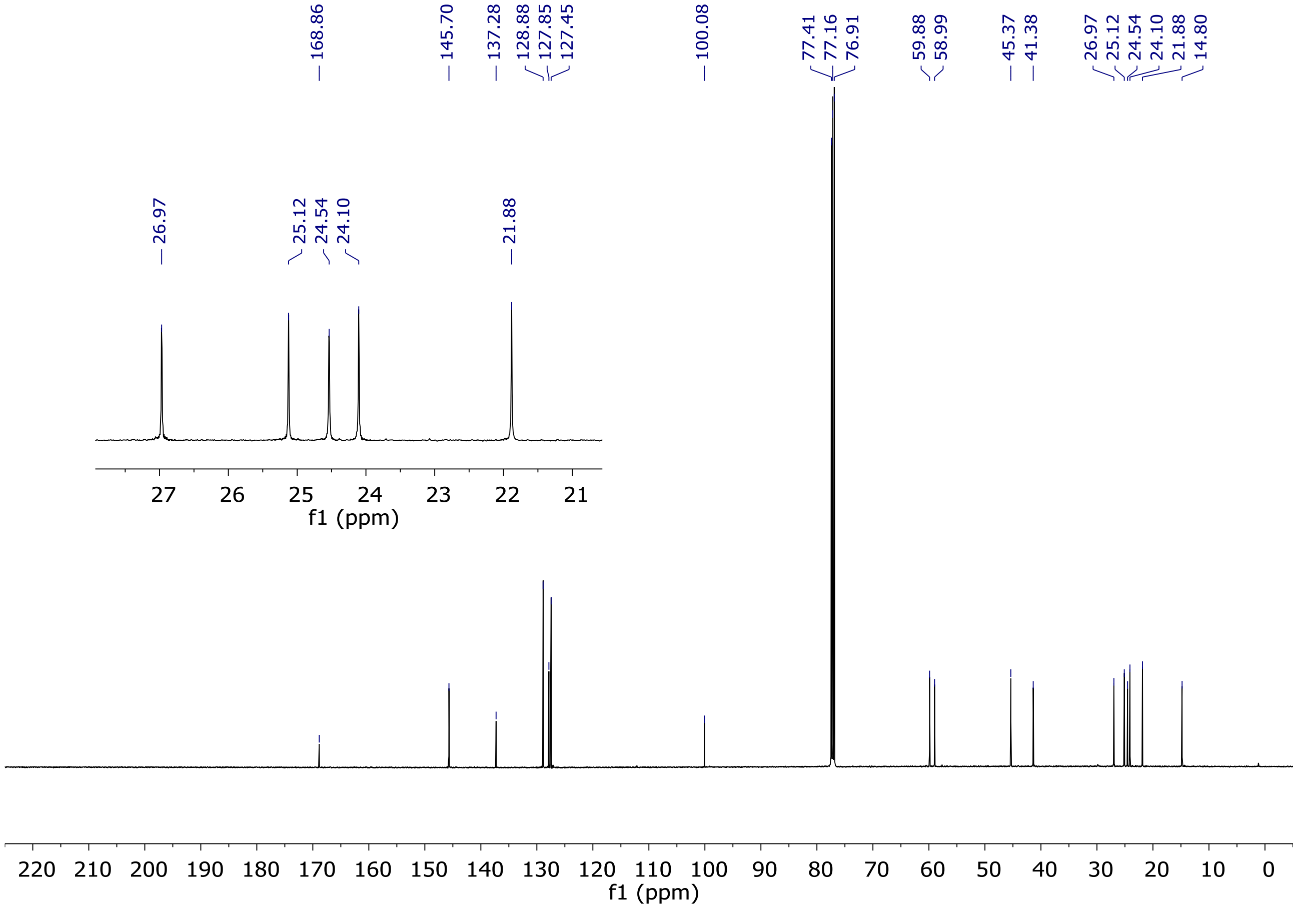
168.86
145.70
137.28
128.88
127.85
127.45
100.08
77.41
77.16
76.91
59.88
58.99
45.37
41.38
26.97
25.12
24.54
24.10
21.88
14.80
f1 (ppm)

12. References

1. J. W. Clary, T. J. Rettenmaier, R. Snelling, W. Bryks, J. Banwell, W. T. Wipke, B. Singaram, *J. Org. Chem.* **2011**, *76*, 9602.
2. H. B. Hepburn, H. W. Lam, *Angew. Chem., Int. Ed.* **2014**, *53*, 11605.
3. G. Dutheuil, N. Selander, K. J. Szabó, V. K. Aggarwal, *Synthesis* **2008**, 2293.
4. C. García-Ruiz, J. L. Y. Chen, C. Sandford, K. Feeney, P. Lorenzo, G. Berionni, H. Mayr, V. K. Aggarwal, *J. Am. Chem. Soc.* **2017**, *139*, 15324.
5. J. D. Sieber, J. P. Morken, *J. Am. Chem. Soc.* **2008**, *130*, 4978.
6. D. L. Silverio, S. Torker, T. Pilyugina, E. M. Vieira, M. L. Snapper, F. Haeffner, A. H. Hoveyda, *Nature* **2013**, *494*, 216.
7. W. R. Roush, A. E. Walts, L. K. Hoong, *J. Am. Chem. Soc.* **1985**, *107*, 8186.
8. H. V. Mierde, P. V. D. Voort, F. Verpoort, *Tetrahedron Lett.* **2009**, *50*, 201.
9. G. Bertuzzi, A. Sinisi, D. Pecorari, L. Caruana, A. Mazzanti, L. Bernardi, M. Fochi, *Org. Lett.* **2017**, *19*, 834.
10. C. E. Paul, S. Gargiulo, D. J. Opperman, I. Lavandera, V. Gotor-Fernández, V. Gotor, A. Taglieber, I. W. C. E. Arends, F. Hollmann, *Org. Lett.* **2013**, *15*, 180.
11. C. Nadeau, S. Aly, K. Belyk, *J. Am. Chem. Soc.* **2011**, *133*, 2878.
12. C. Baumert, M. Günthel, S. Krawczyk, M. Hemmer, T. Wersig, A. Langner, J. Molnár, H. Lage, A. Hilgeroth, *Bioorg. Med. Chem.* **2013**, *21*, 166.
13. T. Thanh Dang, A. Chen, A. Majeed Seayad, *RSC Adv.* **2014**, *4*, 30019.
14. J. Day, M. Uroos, R. A. Castledine, W. Lewis, B. McKeever-Abbas, J. Dowden, *Org. Biomol. Chem.* **2013**, *11*, 6502.
15. A. W. Jensen, J. M. Moore, M. V. Kimble, A. P. Ausmus, W. L. Dilling, *Tetrahedron Lett.* **2016**, *57*, 5636.
16. R. Krishnamoorthy, S. Q. Lam, C. M. Manley, R. J. Herr, *J. Org. Chem.* **2010**, *75*, 1251.
17. S. Khong, O. Kwon, *J. Org. Chem.* **2012**, *77*, 8257.
18. A. Monrose, H. Salembier, T. Bousquet, S. Pellegrini, L. Pélinski, *Adv. Synth. Catal.* **2017**, *359*, 2699.
19. A. Hilgeroth, C. Baumert, C. Coburger, M. Seifert, S. Krawczyk, C. Hempel, F. Neubauer, M. Krug, J. Molnar, H. Lage, *Med. Chem.* **2013**, *9*, 487.
20. G. Bertuzzi, A. Sinisi, L. Caruana, A. Mazzanti, M. Fochi, L. Bernardi, *ACS Catal.* **2016**, *6*, 6473.
21. Y. Yasuda, H. Ohmiya, M. Sawamura, *Angew. Chem., Int. Ed.* **2016**, *55*, 10816.
22. B. Qin, U. Schneider, *J. Am. Chem. Soc.* **2016**, *138*, 13119.
23. S. Shimada, A. S. Batsanov, J. A. K. Howard, T. B. Marder, *Angew. Chem., Int. Ed.* **2001**, *40*, 2168.
24. H. F. Bettinger, M. Filthaus, H. Bornemann, I. M. Oppel, *Angew. Chem., Int. Ed.* **2008**, *47*, 4744.
25. S. Cao, R. Christiansen, X. Peng, *Chem. Eur. J.* **2013**, *19*, 9050.
26. Gaussian 16, Revision A.01, Frisch, M. J.; Trucks, G. W.; Schlegel, H. B.; Scuseria, G. E.; Robb, M. A.; Cheeseman, J. R.; Scalmani, G.; Barone, V.; Petersson, G. A.; Nakatsuji, H.; Li, X.; Caricato, M.; Marenich, A. V.; Bloino, J.; Janesko, B. G.; Gomperts, R.; Mennucci, B.; Hratchian, H. P.; Ortiz, J. V.; Izmaylov, A. F.; Sonnenberg, J. L.; Williams-Young, D.; Ding, F.; Lipparini, F.; Egidi, F.; Goings, J.; Peng, B.; Petrone, A.; Henderson, T.; Ranasinghe, D.; Zakrzewski, V. G.; Gao, J.; Rega, N.; Zheng, G.; Liang, W.; Hada, M.; Ehara, M.; Toyota, K.; Fukuda, R.; Hasegawa, J.; Ishida, M.; Nakajima, T.; Honda, Y.; Kitao, O.; Nakai, H.; Vreven, T.; Throssell, K.; Montgomery, J. A., Jr.; Peralta, J. E.; Ogliaro, F.; Bearpark, M. J.; Heyd, J. J.; Brothers, E. N.; Kudin, K. N.; Staroverov, V. N.; Keith, T. A.; Kobayashi, R.;

Normand, J.; Raghavachari, K.; Rendell, A. P.; Burant, J. C.; Iyengar, S. S.; Tomasi, J.; Cossi, M.; Millam, J. M.; Klene, M.; Adamo, C.; Cammi, R.; Ochterski, J. W.; Martin, R. L.; Morokuma, K.; Farkas, O.; Foresman, J. B.; Fox, D. J. Gaussian, Inc., Wallingford CT, 2016.

27. A. V. Marenich, C. J. Cramer, D. G. Truhlar, *J. Phys. Chem. B* **2009**, *113*, 6378-6396.
28. C. Adamo, V. Barone, *J. Chem. Phys.* **1999**, *110*, 6158-6170.
29. (a) F. Weigend, R. Ahlrichs, *Phys. Chem. Chem. Phys.* **2005**, *7*, 3297-3305. (b) F. Weigend, *Phys. Chem. Chem. Phys.* **2006**, *8*, 1057-1065.
30. G. Luchini, J. Alegre-Requena, I. Funes-Ardoiz, R. Paton, *F1000Research* **2020**, *9*, 291.
